# Hydrogen bond stabilized *CO intermediate enables CO_2_ electroreduction to multi-electron products on silver catalysts

**DOI:** 10.1038/s41467-026-73970-9

**Published:** 2026-06-25

**Authors:** Weiluo Zhang, Yilei Zhang, Da Wan, Junrong Gao, Shuling Zheng, Jie He, Huizhu Cai, Xue Zhang, Qi Hu, Hengpan Yang, Chuanxin He

**Affiliations:** College of Chemistry and Environmental Engineering, Shenzhen University, Shenzhen, Guangdong China

**Keywords:** Electrocatalysis, Carbon capture and storage

## Abstract

Silver is an extensively investigated electrode material for electrochemical CO_2_ reduction owing to its high electrical conductivity and structural stability. However, a key limitation of silver catalysts is their weak adsorption of the *CO intermediate. This intrinsic constraint restricts the reaction primarily to two-electron pathways, hindering the formation of multi-electron products. Here we demonstrate that surface molecular modification can address this limitation. By anchoring bromothymol blue molecules onto the silver surface, we engineer a localized hydrogen-bonding network that stabilizes *CO intermediates via O···H–O interactions, prolonging their surface residence time. In situ spectroscopy and theoretical simulations reveal that this local microenvironment thermodynamically stabilizes *CO against desorption while kinetically facilitating its subsequent hydrogenation and C-C coupling. Consequently, the retained *CO undergoes deeper reduction pathways, generating CH_4_, C_2_H_4_, C_2_H_5_OH, and CH_3_COOH. At a current density of 400 mA cm^−2^, the Faradaic efficiency for multi-electron products reaches 24.2%. This work shifts the design paradigm from metal-centric electronic tuning to local microenvironment engineering, offering an alternative strategy for enabling multi-electron transfer on non-copper catalysts.

## Abstract (web summary)

Electrochemical CO_2_ reduction on silver is often limited to two-electron products due to weak intermediate adsorption. Here, the authors report a molecular modification strategy that uses hydrogen bonding to stabilize intermediates, enabling the formation of multi-electron products.

## Introduction

The electrochemical CO_2_ reduction reaction (CO_2_RR) has emerged as a pivotal technology to mitigate carbon emissions while enabling renewable energy storage through the conversion of CO_2_ into value-added chemicals^1^. Among the possible CO_2_RR products, multi-electron products (e.g., CH_4_, C_2_H_4_, C_2_H_5_OH, CH_3_COOH) are especially desirable due to their higher energy densities and established market demand^2–7^.

However, the selective formation of multi-electron products remains fundamentally challenging because it requires multiple proton-coupled electron-transfer (PCET) steps, stabilization of reactive intermediates, and precise control over competing reaction pathways. By comparison, while the formation of CO and HCOOH typically proceeds through 2e^−^ transfer steps, deeper reduction to CH_4_ (8e^−^) or CH_3_COOH (8e^−^), and especially to C_2_ products like C_2_H_4_ or C_2_H_5_OH (12e^−^), involves intricate proton-coupled multi-electron transfer sequences^8–11^. These complicated mechanisms require the competitive ability to stabilize a series of reaction intermediates during CO_2_RR. Among monometallic catalysts, Cu occupies a unique position owing to its ability to produce multi-carbon products with appreciable efficiency, a capability widely attributable to its favorable *CO adsorption energy. Such optimized adsorption energetics not only stabilize *CO sufficiently to facilitate its further hydrogenation but also promote the critical C-C coupling step, by maintaining an optimal surface coverage and configuration of *CO and related species^12,13^. Nevertheless, Cu-based catalysts are prone to restructuring or poisoning in CO_2_RR, leading to performance degradation and limited stability^14^.

Ag-based materials, owing to their high electrical conductivity and corrosion resistance, are widely regarded as attractive catalysts for electrochemical CO_2_ reduction. However, the key intermediate *CO exhibits weak adsorption on the Ag surface, which favors its rapid desorption as CO and suppresses its deeper reduction toward multi-electron products^15,16^. For deeper reduction to occur, the *CO intermediate must remain stably adsorbed on the surface instead of undergoing premature desorption. Sufficient residence time enables its subsequent hydrogenation to critical downstream intermediates such as *CHO or *COH, a step that is prohibitively unfavorable on Ag due to its suboptimal *CO binding strength^17^. Hence, by strategically prolonging the residence time of *CO, it is possible to unlock its intrinsic reactivity, thereby opening a viable pathway towards deeper reduction into multi-electron products.

Researchers have pursued diverse catalyst designs aimed at modulating *CO adsorption in order to promote multi-electron product formation on non-Cu catalysts^18–22^. For example, Zhang et al. demonstrated that imparting chirality to Ag can overcome this limitation via chirality-induced spin polarization of the reaction intermediate. They fabricated chiral nanostructured Ag films with helical lattice distortions that stabilize a triplet-state *OCCO intermediate and enable C-C coupling, thereby achieving a 4.7% FE for C_2+_ products at a current density of 22 mA cm^−2^ under 12.5 atm of CO_2_^23^. This spin-selective strategy highlights how tailored lattice distortions and spin polarization can modulate otherwise unfavorable reaction energetics, uncovering the feasibility of activating Ag-based catalysts toward C_2+_ synthesis.

Herein, we report a molecular modification strategy^24,25^ for Ag surfaces using bromothymol blue (BTB), a molecule whose hydroxyl groups form hydrogen bonds with *CO intermediates during CO_2_ electroreduction. In situ Fourier-transform infrared (FT-IR) and Raman spectroscopy reveal that this interfacial functionalization significantly prolongs the surface residence time of *CO, thereby facilitating hydrogenation and C-C coupling pathways. Density functional theory (DFT) calculations show that the Ag-BTB interface provides an optimal *CO binding energy while substantially lowering the energy barriers for *CO dimerization and *CO hydrogenation. Molecular dynamics (MD) simulations further indicate that the *CO intermediate exhibits prolonged interfacial residence, consistent with a kinetic trapping effect. Collectively, this multifaceted modulation, which combines thermodynamic stabilization of intermediates with kinetic trapping, synergistically promotes subsequent hydrogenation steps and *COH-*CO coupling, ultimately enhancing the selective formation of multi-electron products.

## Results

### Catalysts synthesis and characterization

**Fig. 1:**
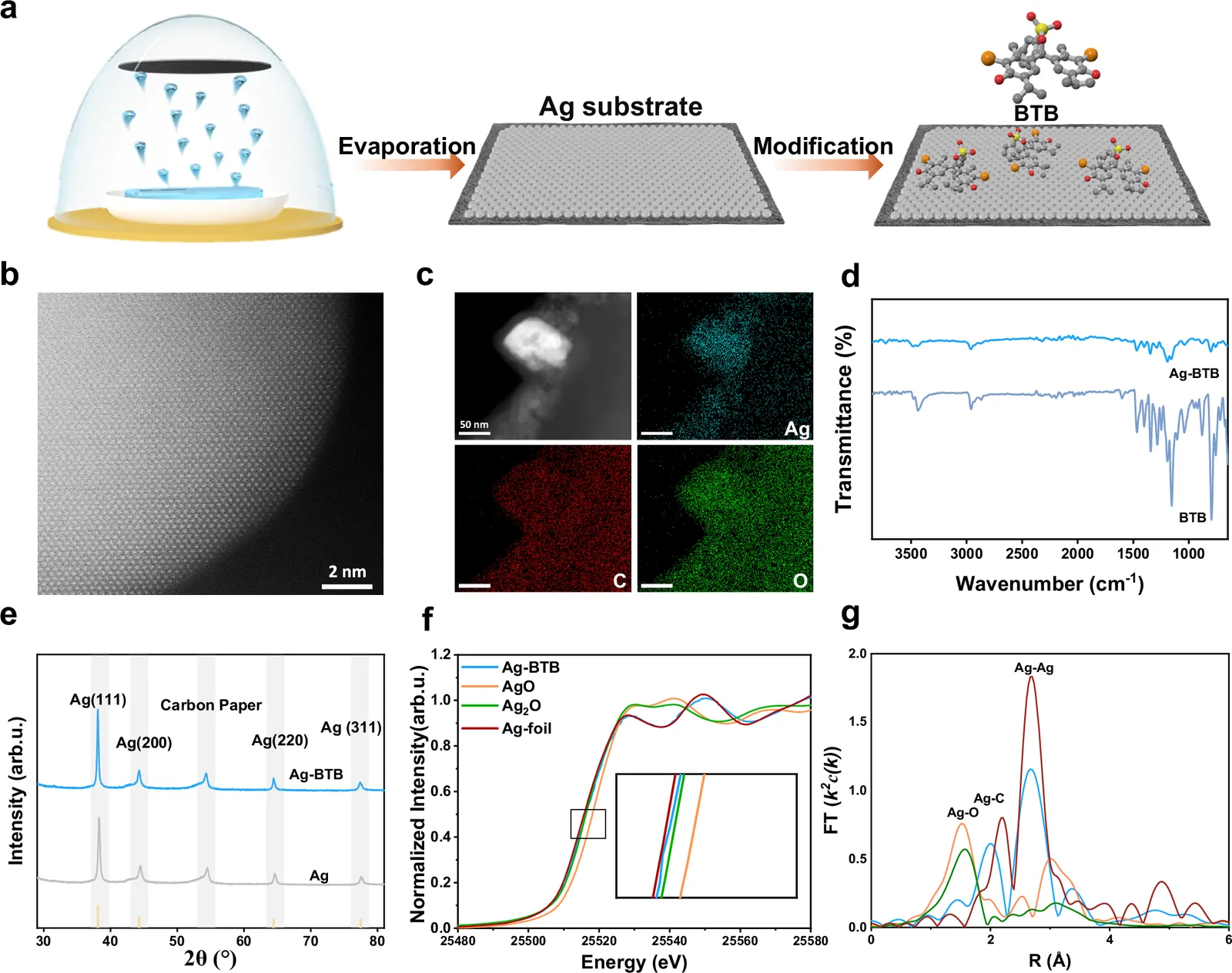
Synthesis, morphology, and structural characterization of the catalysts. **a** The synthesis scheme of Ag-BTB; (**b**) Spherical aberration-corrected HAADF-STEM image and (**c**) EDS elemental mapping of Ag-BTB catalyst; (**d**) FT-IR spectra and (**e**) XRD spectra of catalysts; (**f**) XANES and (**g**) FT-EXAFS spectra of Ag-BTB and the references. Source data are provided as a Source Data file.

The catalyst preparation procedure is schematically illustrated in Fig. 1a. Initially, a uniform Ag film was deposited onto commercial carbon paper via thermal evaporation. The substrate was subsequently modified with organic molecules, exemplified by bromothymol blue (BTB), to establish a generalized interfacial modification strategy for enhancing CO_2_RR activity. Full synthesis details are provided in the Methods section.

The morphological characteristics and elemental distribution of the as-prepared Ag and Ag-BTB catalysts were systematically characterized by scanning electron microscopy (SEM) coupled with energy-dispersive X-ray spectroscopy (EDS) (Supplementary Figs. S1, 2). SEM analysis reveals that the catalysts exhibit well-dispersed and distinct morphological features. EDS mapping further confirms the homogeneous distribution of key elements (Ag, C, and O) throughout the catalyst surfaces, demonstrating the successful and uniform modification of the organic molecules. Complementary transmission electron microscopy (TEM) and aberration-corrected electron microscopy (AC-TEM) analyses were additionally employed to provide higher-resolution morphological characterization, which further corroborated the well-defined nanostructures and uniform dispersion of the catalysts (Supplementary Figs. S3–5). High-angle annular dark-field scanning transmission electron microscopy (HAADF-STEM) images and corresponding energy-dispersive X-ray spectroscopy mapping (Fig. 1b, c) reveal that the Ag-BTB catalyst exhibits well-defined crystalline features.

Fourier-transform infrared (FT-IR) spectroscopy was employed to verify the successful functionalization of BTB molecules on the Ag surfaces (Fig. 1d). The spectra of Ag-BTB clearly retain the characteristic vibrational signatures of  its respective organic modifiers, demonstrating that the molecular structures of BTB remain intact following surface attachment. X-ray diffraction (XRD) analysis was performed to investigate the crystalline structure of the prepared catalysts. As shown in Fig. 1e, characteristic peaks are observed at ~ 38.1°, 44.2°, and 64.4°, which correspond to the (111), (200), and (220) crystallographic planes of Ag (PDF#04-0783), respectively, along with an additional peak at ~ 54.3° attributed to the carbon paper substrate. The dominant intensity of the (111) reflection suggests a preferential (111) orientation of the deposited Ag film. Static water contact angle (WCA) measurements (Supplementary Fig. S6) also confirm the successful introduction of polar functional groups (-OH) at the Ag interface.

X-ray photoelectron spectroscopy (XPS) analysis was conducted to investigate the electronic interactions between Ag and BTB (Supplementary Fig. S7). Comparative analysis reveals a negative shift in the Ag 3 *d* binding energy for Ag-BTB relative to pristine Ag, accompanied by a positive shift in the O 1 *s* binding energy (Supplementary Fig. S8). These systematic shifts suggest electron transfer from the hydroxyl oxygen of BTB to Ag atoms, consistent with increased electron density on the Ag surface. To elucidate the precise oxidation states and atomic-scale coordination environments of the silver species, we conducted comprehensive X-ray absorption fine structure (XAFS) spectroscopic investigations. The X-ray absorption near-edge structure spectra (XANES, Fig. 1f) demonstrate that Ag-BTB has an absorption edge position closer to that of Ag foil, suggesting that the dominant metallic state (Ag^0^) is maintained in the catalyst. Fourier-transformed extended X-ray absorption fine structure (FT-EXAFS, Fig. 1g, Supplementary Figs. S9, 10 and Supplementary Table S1) reveals that the main peak at ~ 2.87 Å corresponds to Ag-Ag metallic bonding, while the secondary peak at ~ 2.30 Å indicates the presence of Ag-O coordination, and a peak at ~ 2.18 Å is assigned to Ag-C coordination^26,27^. Furthermore, the wavelet transform analysis of Ag-BTB exhibits maximum intensity at the same frequency components as metallic Ag foil (Supplementary Fig. S11), providing additional confirmation of the dominant Ag-Ag metallic bonding. These results clearly demonstrate that despite the modification by BTB molecules, the Ag substrate still maintains its metallic state.

### CO_2_RR performance evaluation

**Fig. 2:**
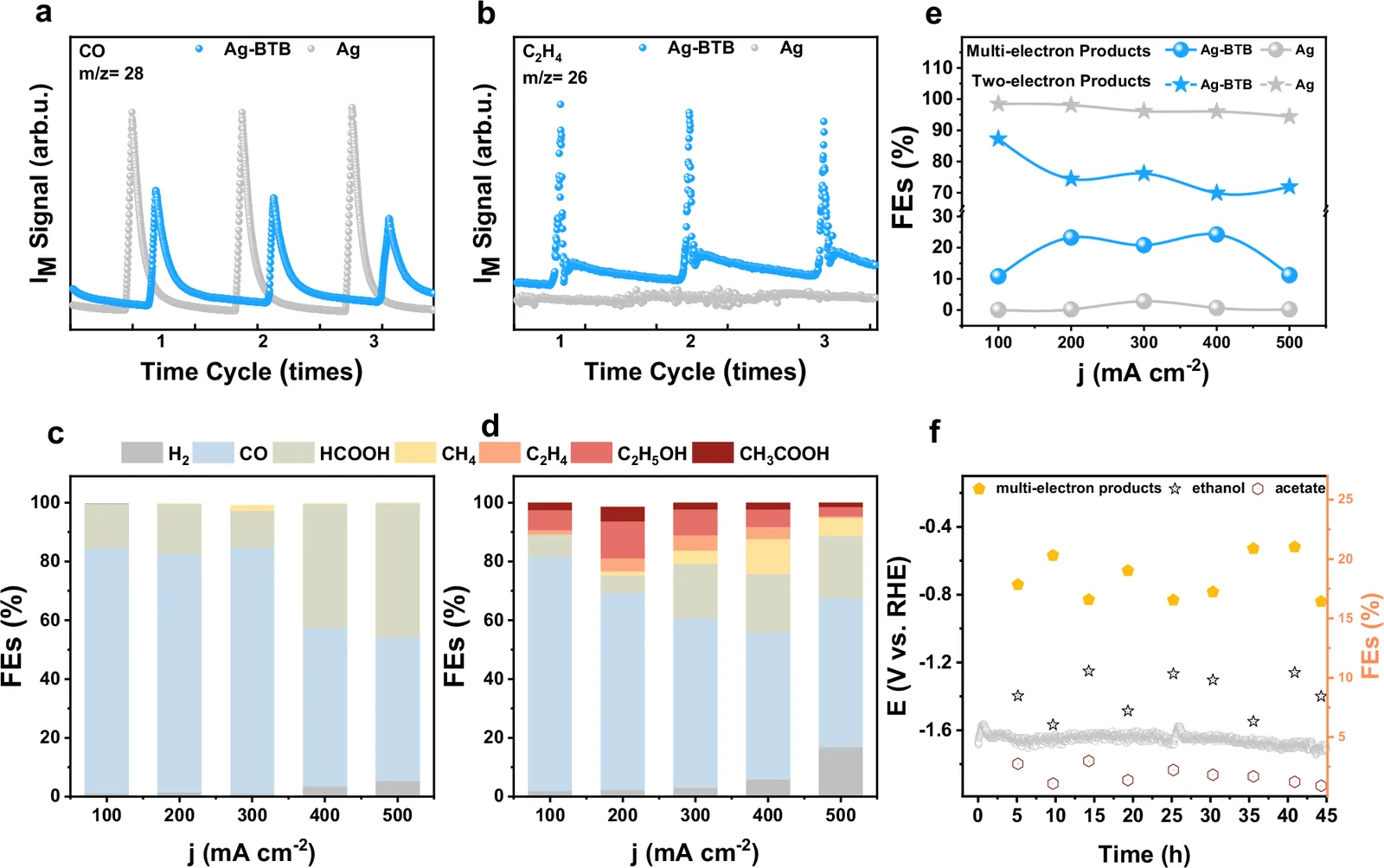
Electrocatalytic CO_2_ reduction performance and stability. **a**, **b** In situ DEMS data obtained during CO_2_RR on Ag and Ag-BTB catalysts, ionic currents of C_2_H_4_ (m/z = 26 for the fragment of C_2_H_2_^+^), and CO (m/z = 28). **c**, **d** Comparison of the FE product distributions for Ag and Ag-BTB in a flow cell. **e** Comparison of two-electron and multi-electron products at current densities of 100–500 mA cm^−2^. **f** Long-term tests on Ag-BTB at 300 mA cm^−2^ in a flow cell. All reported potentials were not IR corrected. Source data are provided as a Source Data file.

Linear sweep voltammetry measurements (LSV, Supplementary Fig. S12) reveal distinct catalytic behavior between the modified and unmodified Ag catalysts. When comparing responses in Ar-purged versus CO_2_-saturated electrolytes, Ag-BTB demonstrates markedly enhanced current density under a CO_2_ atmosphere relative to pristine Ag, indicating improved CO_2_ activation capacity and optimized interfacial mass transport properties. In situ differential electrochemical mass spectrometry (DEMS) analysis during CO_2_ electroreduction reveals distinct catalytic behaviors between Ag and Ag-BTB (Fig. 2a, b and Supplementary Fig. S13). Compared to pristine Ag, the mass spectral response on the Ag-BTB catalyst exhibits a significantly attenuated CO (m/z = 28) signal. This pronounced suppression of CO gas evolution is consistent with enhanced stabilization of *CO induced by the BTB modifier. This robust *CO stabilization acts as a prerequisite for deeper reduction, as evidenced by the appearance of detectable C_2_H_4_ (tracked via the C_2_H_2_^+^ fragment at m/z = 26) on the Ag-BTB electrode. The altered product distribution is consistent with increased surface retention of *CO intermediates on Ag-BTB. Concurrently, the enhanced CH_4_ signal (tracked via the CH_3_^+^ fragment at m/z = 15) indicates highly favorable *CO sequential protonation pathways. Collectively, these in situ observations demonstrate that BTB functionalization inherently alters the reaction mechanism by simultaneously anchoring the *CO intermediate against premature desorption and accelerating its subsequent coupling and hydrogenation toward value-added multi-electron products.

The electrochemical CO_2_ reduction performance was thoroughly evaluated using a commercially customized flow cell system, with 1.0 M KOH (pH = 13.9 ± 0.1) serving as the electrolyte. The measurements were conducted under galvanostatic conditions across a current density range of 100–500 mA cm^−2^ to systematically assess the catalytic behavior under varying reaction intensities. Product analysis was performed using a combination of online gas chromatography for gaseous products and nuclear magnetic resonance (NMR) spectroscopy for liquid phase products (Supplementary Figs. S14, 15). Comparative studies of the pristine Ag, Ag-BTB (at the optimized modification loading, Supplementary Fig. S16), and various strictly controlled samples reveal striking differences in their electrocatalytic behaviors (Fig. 2c–e and Supplementary Fig. S17). Remarkably, the optimally functionalized Ag-BTB catalyst demonstrates a strong tendency to drive deep reduction toward multi-electron products, including CH_4_, C_2_H_4_, CH_3_COOH, and C_2_H_5_OH. The total Faradaic efficiency (FE) for these value-added multi-electron products reaches up to 24.2% at a high total current density of 400 mA cm^−2^, delivering a corresponding partial current density of 96.8 mA cm^−2^. To elucidate the origin of this competitive activity, two sets of critical control experiments were conducted. First, pristine Ag predominantly yielded two-electron products (CO and HCOOH) along with H_2_, and only trace amounts of methane  were detected. Second, to pinpoint the specific structural motifs within the BTB molecule responsible for this catalytic behavior, we first examined Ag electrodes modified with triphenylmethane (Ag-TPM) and phenyl 4-bromobenzenesulfonate (Ag-PBBS). Under identical reaction conditions, neither of these electrodes afforded any detectable multi-electron products. We subsequently evaluated electrodes functionalized with polyethylene glycol (Ag-PEG) and phenolphthalein (Ag-PP). The Ag-PEG system exhibited no measurable activity toward multi-electron products, whereas Ag-PP produced only trace quantities. Notably, even the limited multi-electron product generation observed on Ag-PP was markedly inferior to that achieved with Ag-BTB. The product distribution of Ag-BTB reveals an optimal balance between *CO surface coverage and *H availability required for efficient multi-electron product production^28–30^. To evaluate the long-term performance of the Ag-BTB catalyst, we conducted chronopotentiometric measurements at a fixed current density of 300 mA cm^−2^ over 45 hours of continuous operation (Fig. 2f). The catalyst maintained stable catalytic performance over extended electrolysis, exhibiting a consistent multi-electron product FE of ~ 18.4% throughout the durability test, with no significant degradation in catalytic activity observed. Post-electrolysis FT-IR and XPS analyses confirmed that the BTB modifier retained its structural integrity and chemical environment after the reaction (Supplementary Figs. S18–20).

To explicitly trace the carbon origin of the multi-electron products and rule out any potential carbon contamination from the degradation of the BTB modifier, isotopic labeling experiments were conducted using ^13^CO_2_ as the reactant under electrocatalytic conditions. Gas-phase products were continuously monitored via in situ Differential Electrochemical Mass Spectrometry. As illustrated in Supplementary Fig. S21, the mass-to-charge ratio (m/z) signals exhibited clear upward shifts corresponding to the ^13^CO_2_ -labeled species. Specifically, ^13^CH_4_ was detected at m/z = 17 (compared to m/z = 15 for ^12^CH_3_^+^); ^13^CO was detected at m/z = 29 (compared to m/z = 28 for ^12^CO); ^13^C_2_H_4_ was detected at m/z = 30 (compared to m/z = 26 for ^12^C_2_H_2_^+^), confirming that the gaseous carbon products originate entirely from the reactant ^13^CO_2_ (m/z = 45). For the liquid-phase products, the electrolyte was comprehensively analyzed using both ^1^H and ^13^C Nuclear Magnetic Resonance spectroscopy (Supplementary Figs. S22, 23). Notably, in the ^1^H NMR spectra, the characteristic peaks of the liquid products evolved from standard singlets into distinct multiplets. This peak splitting is a direct consequence of the robust heteronuclear spin-spin coupling (*J*-coupling) between the ^13^CO_2_ nuclei (spin *I* = 1/2) and adjacent protons (^1^H), providing unequivocal evidence of ^13^C incorporation into the molecular skeletons. This was further corroborated by the ^13^C NMR spectra, which clearly resolved the characteristic chemical shifts of the ^13^C-labeled oxygenates: bicarbonate at ~ 161 ppm; formate at ~ 171 ppm; acetate at ~ 181 ppm (carboxyl carbon) and ~ 23 ppm (methyl carbon); and ethanol at ~ 58 ppm (methylene carbon) and ~18 ppm (methyl carbon). Mechanistically, these isotopic labeling results provide rigorous analytical closure. They explicitly confirm that the highly valuable multi-electron products are synthesized de novo via the C–C coupling of CO_2_-derived intermediates.

### Mechanistic research

**Fig. 3:**
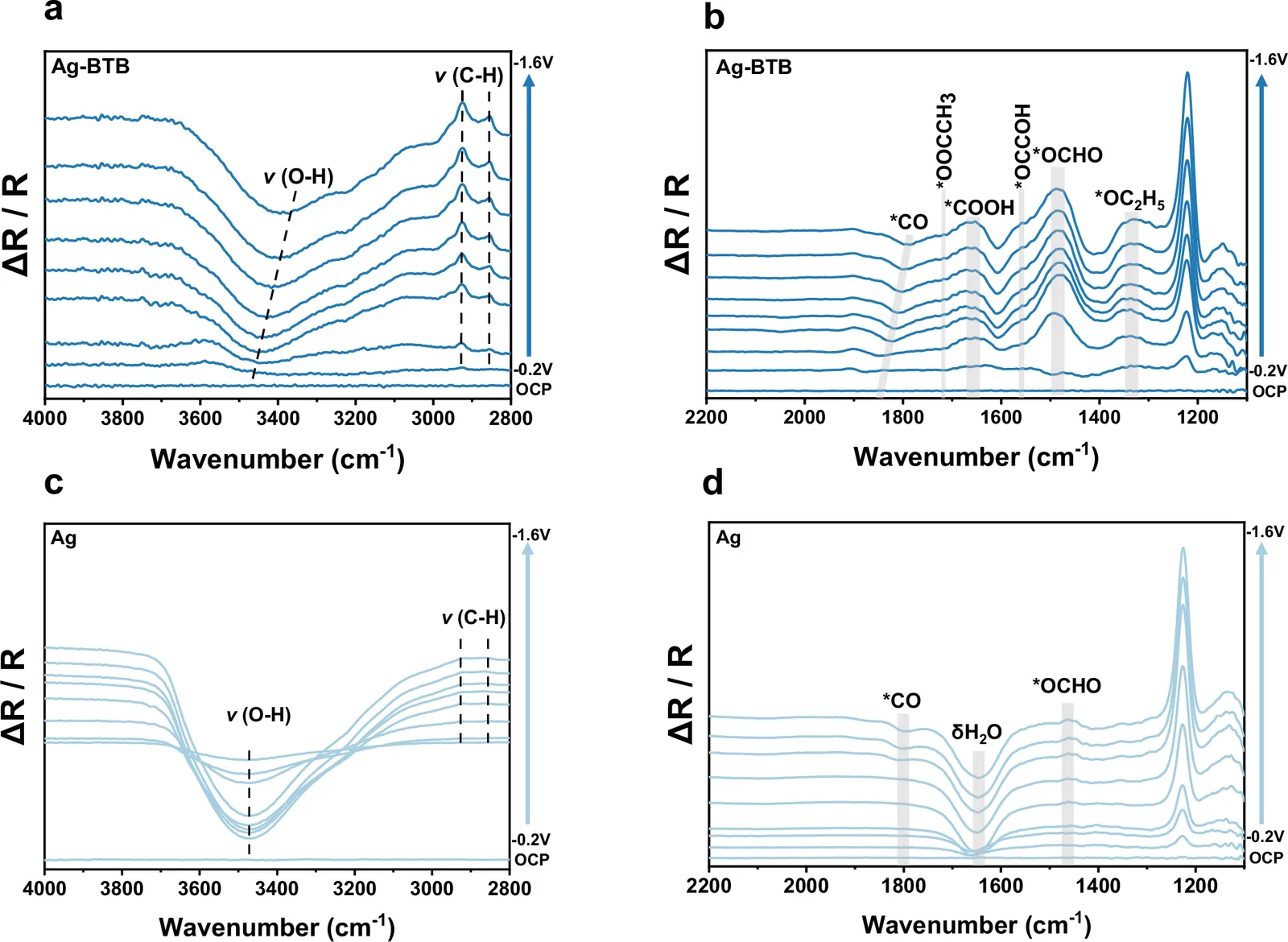
In situ FT-IR Spectroscopic Investigation. In situ FT-IR spectra were collected for both Ag-BTB (**a**, **b**) and the pristine Ag catalyst (**c**, **d**). The spectra display infrared signals in the ranges of 2800–4000 cm^−1^ and 1100–2200 cm^−1^, respectively. All reported potentials were not IR corrected. Source data are provided as a Source Data file.

In situ Fourier-transform infrared (FT-IR) characterization reveals distinct reaction pathways at the Ag-BTB interface compared to a pristine Ag surface (Fig. 3a–d and Supplementary Fig. S24). The unmodified Ag surface exhibits a characteristic O-H stretching vibration of interfacial water at 3480 cm^−1^, along with a signature peak at 1460 cm^−1^ corresponding to the *OCHO intermediate, indicating the HCOOH production pathway^31,32^. In contrast, the BTB-modified surface displays markedly different vibrational features. For example, potential-dependent FT-IR spectroscopy reveals that the broad O-H stretching band at the Ag-BTB interface (~ 3450 cm^−1^) not only intensifies but also continuously red-shifts with increasing cathodic potential. The band is broad because of a distribution of hydrogen-bond strengths, and it is strong because of the enhanced polarity of the O-H bond. The observed red shift occurs because the strengthened hydrogen bonds at more negative potentials further elongate and weaken the O-H covalent bonds, which lowers their vibrational frequency and signals a reorganization of the interfacial network. Importantly, an obvious C-H stretching vibration emerges around 2900 cm^−1^ on the BTB-modified surface, providing direct spectroscopic evidence of ongoing *CO hydrogenation processes^33^.

Furthermore, the BTB-modified catalyst exhibits additional bands associated with *CO intermediate. For example, a feature at 1650 cm^−1^ is assigned to the *COOH intermediate (a precursor to *CO formation), and an intense band at 1840cm^−1^ corresponds to adsorbed *CO^34,35^. Notably, the *CO band at 1840 cm⁻¹ exhibits distinct broadening, a red-shift, and enhanced intensity compared to pristine Ag. Strong interfacial hydrogen-bonding interactions induce significant polarization of the *CO adsorbate, localizing excess electron density at the highly nucleophilic oxygen terminus. This electronic polarization modifies the vibrational potential well of the C–O bond, thereby lowering its vibrational frequency (red-shift) while simultaneously preserving its structural stability. The band broadening further reflects a statistical distribution of dynamic hydrogen-bond strengths within this highly heterogeneous microenvironment. Concurrently, the intensity increase signifies a higher surface coverage of *CO and an augmented transition dipole moment, both facilitated by the stabilizing effect of interfacial hydrogen bonding^36^.

These observations suggest that the BTB-functionalized interface facilitates *CO formation and retention, thereby creating favorable conditions for the subsequent C-C coupling process. Indeed, an *OCCOH intermediate is observed at 1558 cm^−1^, providing a definitive spectroscopic signature of C-C bond formation. Bands at 1718 cm^−1^ are assigned to *OOCCH_3_, a route to CH_3_COOH formation; similarly, features at 1330 cm^−1^ correspond to *OC_2_H_5_, a key intermediate in C_2_H_5_OH formation^37–39^. These spectroscopic observations demonstrate that BTB modification not only stabilizes *CO intermediate against desorption but also creates an interfacial environment conducive to their sequential hydrogenation and coupling. This dual effect enables the efficient formation of multi-electron products through well-defined reaction pathways^40^.

**Fig. 4:**
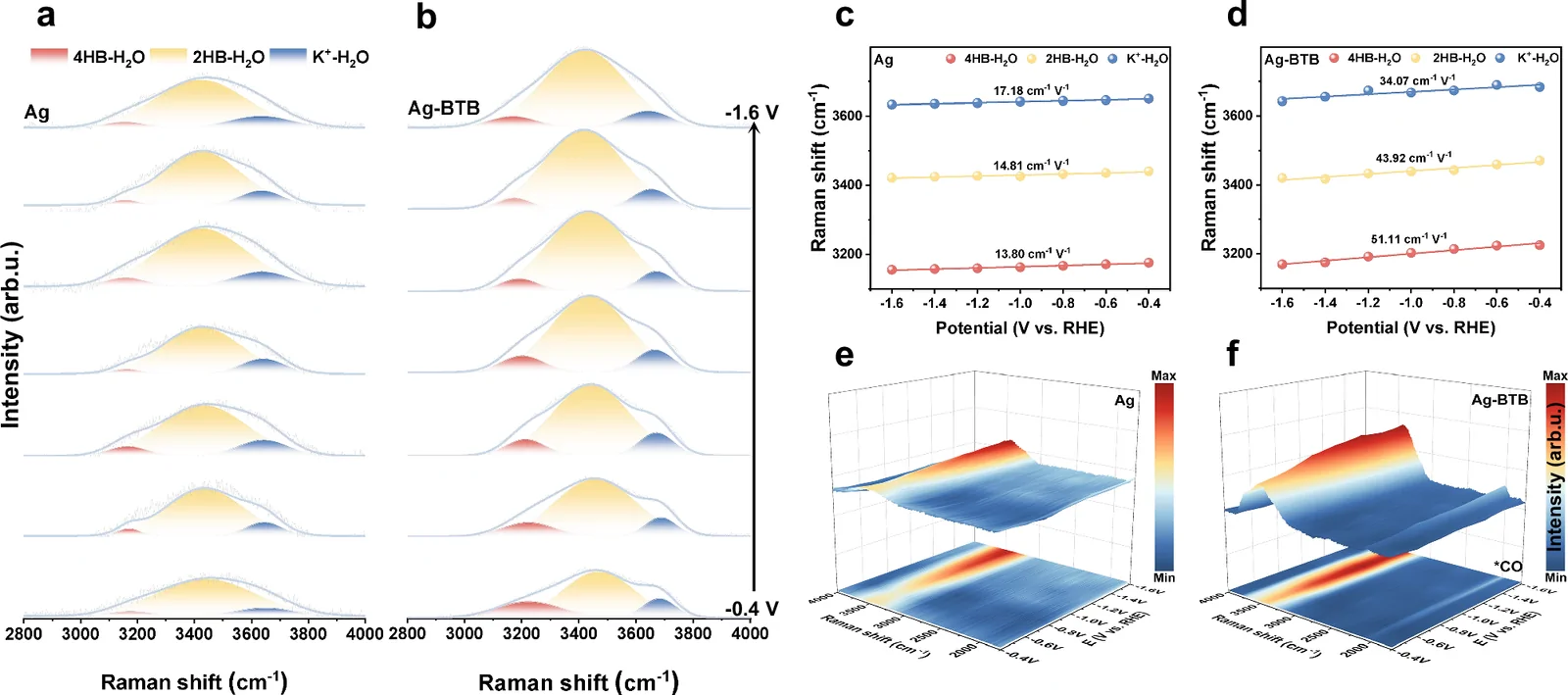
In situ Raman Spectroscopic Investigation. Peak fitting of the water peaks for (**a**) Ag and (**b**) Ag-BTB at various potentials; corresponding Stark slope analysis for (**c**) Ag and (**d**) Ag-BTB; In situ Raman spectra for (**e**) Ag and (**f**) Ag-BTB in the range of 1800–4000 cm^−1^. All reported potentials were not IR corrected. Source data are provided as a Source Data file.

To elucidate the reaction intermediates and the interfacial water microenvironment during CO_2_ electroreduction, in situ Raman spectroscopy was performed on both pristine Ag and Ag-BTB catalysts. Focusing on the O-H stretching region around 3440 cm^−1^, both catalysts exhibit a broad water band. As shown in Fig. 4a, b, Gaussian deconvolution of this region reveals three distinct water configurations: four-coordinated hydrogen-bonded water (4-HB·H_2_O, ~ 3200 cm^−1^), two-coordinated hydrogen-bonded water (2-HB·H_2_O, ~ 3450 cm^−1^), and K^+^-hydrated water (K^+^-H_2_O, ~ 3650 cm^−1^)^41,42^. Statistical analysis of these peak populations at various applied potentials (Supplementary Fig. S25) indicates that while 2-HB·H_2_O dominates on both surfaces, the Ag-BTB interface possesses a slightly higher proportion of 4-HB·H_2_O compared to pristine Ag. The mechanistic impact of this interfacial restructuring is further illuminated by the Stark tuning diagrams (Fig. 4c, d). A comparison of the Stark slopes reveals a marked difference between the two electrodes. For the pristine Ag electrode, the Stark slopes for K^+^-H_2_O, 2-HB·H_2_O, and 4-HB·H_2_O are 17.18, 14.81, and 13.8 cm^−1^ V^−1^, respectively. These moderate tuning rates suggest that water molecules on pristine Ag experience a relatively weak average local electric field, indicating that the Raman signal likely contains significant contributions from the less-polarized diffuse layer or bulk water. In striking contrast, the corresponding Stark slopes on the Ag-BTB electrode exhibit enhancement, reaching 34.07, 43.92, and 51.11 cm^−1^ V^−1^, respectively. Since the vibrational Stark tuning rate is directly proportional to the local interfacial electric field strength, these elevated values demonstrate that BTB functionalization generates a highly concentrated and robust local electric field. This enhanced local electric field drives the reorganization of the interfacial water network, wherein optimally aligned water dipoles and hydrogen-bonding interactions synergistically stabilize key reaction intermediates^25,43^. This stabilization mechanism is directly corroborated by the detection of carbonaceous intermediates (Fig. 4e, f). The Ag-BTB catalyst exhibits a pronounced vibrational band at 2070 cm^−1^, assigned to a stabilized *CO intermediate^44^, which is completely absent on the unmodified Ag surface^45^. This observation confirms enhanced stabilization through strong interactions with the BTB-modified interface.

**Fig. 5:**
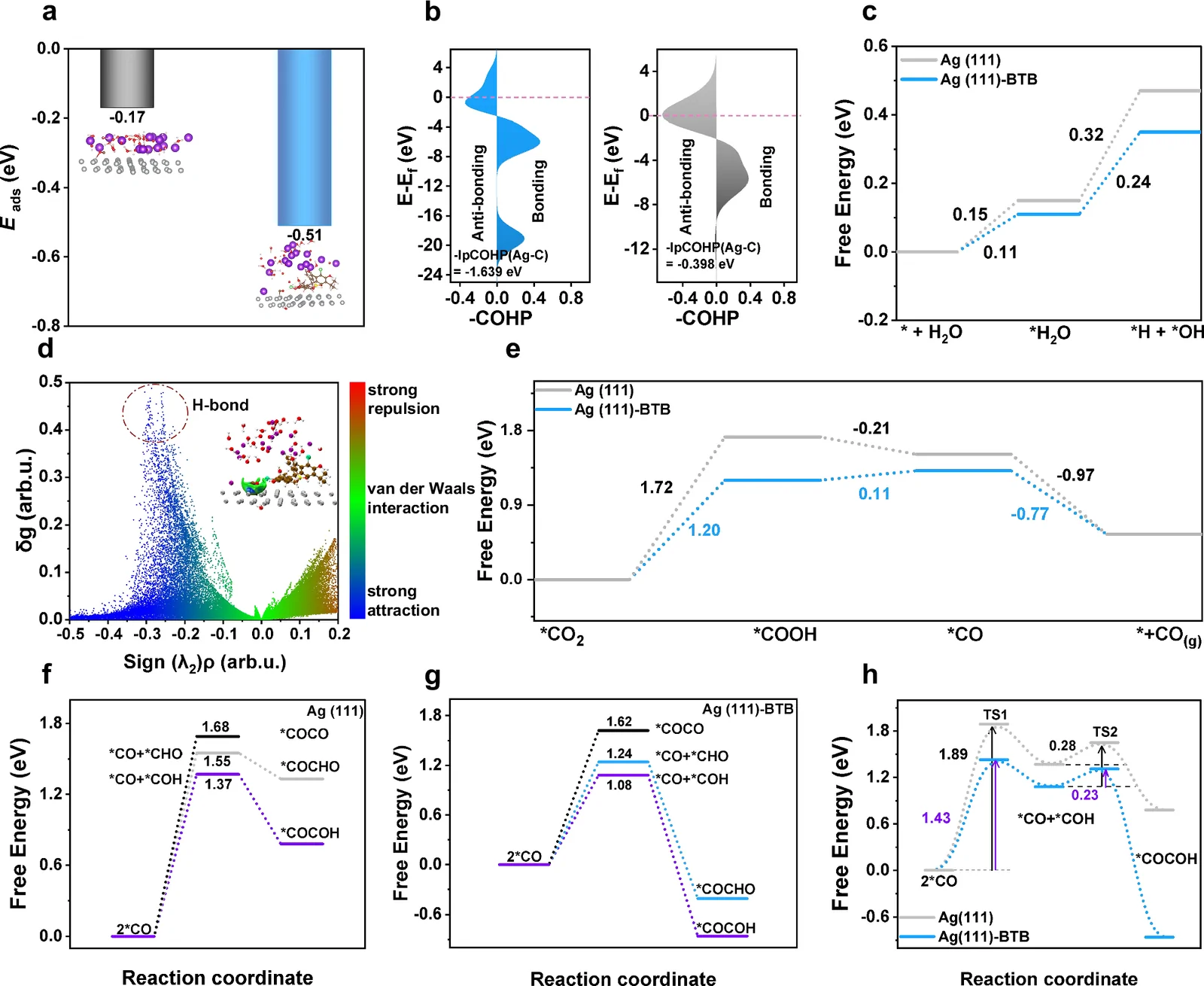
Theoretical calculations of the reaction mechanism, thermodynamics, and kinetics. **a** The *E*_ads_ of *CO on the electrode surfaces. **b** COHP curves for the Ag-C bonds in *CO on electrode surfaces. **c** Free-energy diagram of H_2_O dissociation on Ag (111) and Ag (111)-BTB. **d** Independent gradient model based on Hirshfeld partition (IGMH) analysis of the interaction between Ag-*CO and BTB. **e** Gibbs free energy diagrams for *CO_2_ electroreduction to CO on Ag (111) and Ag (111)-BTB. Free energy profiles of the *CO hydrogenation and coupling steps on (**f**) Ag (111) and (**g**) Ag (111)-BTB. **h** Kinetic analysis of the transition states associated with *CO hydrogenation and C-C coupling. Light gray, brown, red, yellow, green, light pink, and purple balls correspond to Ag, C, O, S, Br, H, and K atoms, respectively. Source data are provided as a Source Data file.

Density functional theory calculations were performed to elucidate the mechanistic role of BTB modification in promoting CO_2_ reduction to multi-electron products. The electronic interactions at the modified interface were systematically investigated using Ag (111) and BTB-modified Ag (111) as model systems (Supplementary Data 1). An optimized configuration of BTB interacting with *CO was constructed on the Ag (111) surface, revealing a direct interaction between *CO and the hydroxyl group of BTB, with an intermolecular distance of *d* = 2.01 Å (Supplementary Fig. S26). This optimized interfacial configuration suggests favorable electronic coupling between BTB and the key *CO intermediate within the simplified computational model. This electronic redistribution is also reflected in the enhanced *CO adsorption energy on Ag (111)-BTB (− 0.51 eV) compared to pristine Ag (111) (− 0.17 eV, Fig. 5a), suggesting enhanced interaction between *CO and Ag-BTB.

To comprehensively elucidate the stabilization and activation mechanism of the *CO intermediate, we performed a multi-dimensional electronic structure analysis. The density of states (DOS) of Ag 3*d* orbitals near the Fermi level (Supplementary Fig. S27) and the projected DOS (PDOS) analysis of the O 2*p* and C 2*p* orbitals of *CO (Supplementary Fig. S28), which demonstrate greater orbital overlap in the Ag (111)-BTB system, corresponding to strengthened C-O bonding and significantly enhanced interfacial electronic coupling on the Ag-BTB surface. Quantitatively, Bader charge analysis (Supplementary Figs. S29, 30) shows that electron injection from the substrate into *CO increases from 0.31|e| (pristine Ag) to 0.54|e| (Ag-BTB). Correspondingly, the projected crystal orbital Hamiltonian population (pCOHP) (Fig. 5b) for the Ag–C interaction exhibits a more negative integrated value, suggesting stronger interfacial anchoring of *CO, consistent with suppressed *CO desorption. Interestingly, despite the increased electron transfer, both PDOS orbital overlap and C–O pCOHP (Supplementary Fig. S31) analysis indicate a reinforced C–O bond. This apparent electronic behavior is further elucidated by differential charge density analysis (Supplementary Figs. S29, 30), which suggests a characteristic “push-pull” electronic effect. The Ag substrate donates electrons into the adsorbate, while the interfacial hydrogen-bonding network (BTB-OH···*CO) redistributes and localizes this density onto the oxygen terminus. This spatial redistribution is associated with reduced population of C–O antibonding orbitals, thereby structurally stabilizing the C–O framework. Simultaneously, the resulting polarization transforms the oxygen terminus into a nucleophilic reactive center, which could facilitate the subsequent proton-coupled hydrogenation. Furthermore, we investigated the water dissociation process as it governs the supply of active hydrogen for *CO hydrogenation, a critical step in CO_2_RR. The calculations suggest that BTB modification reduces the energy barrier for H_2_O dissociation (Fig. 5c and Supplementary Figs. S32, 33), thereby potentially increasing the surface coverage of active *H species and accelerating subsequent *CO hydrogenation and coupling process.

To explicitly visualize the intermolecular interactions driving the interfacial polarization, independent gradient model based on Hirshfeld partition (IGMH)^46^ analysis was performed (Fig. 5d). In this method, the interaction type and strength are mapped by the sign(λ_2_)ρ function, where blue, green, and red regions signify strong attraction (e.g., hydrogen bonding), weak van der Waals interactions, and strong steric repulsion, respectively. As illustrated in the scatter plot, a prominent spike emerges in the intensely blue region (sign(λ_2_)ρ < 0), indicative of strong attractive forces. Coupled with the localized blue 3D isosurface situated between the *CO intermediate and the BTB, these results are consistent with the formation of interfacial hydrogen-bonding interactions between BTB and *CO.

To gain deeper mechanistic insights, we systematically computed the free energy landscapes for CO_2_ electroreduction. As shown in the free energy profiles from *CO_2_ to CO with corresponding structural models in Supplementary Figs. S34–37, Ag-BTB exhibits a clear advantage in initial *CO_2_ activation over pristine Ag. More importantly, the desorption of *CO to form gaseous CO is significantly more energetically unfavorable on Ag-BTB than on Ag (111) (Fig. 5e). This indicates that the BTB-modified surface effectively stabilizes the *CO intermediate, thereby driving its further deep reduction. While numerous possibilities exist for the initial C–C coupling (e.g., *OCCO, *OCCHO, and *OCCOH)^47^, to elucidate the fundamental origin of this selectivity, we systematically evaluated the reaction thermodynamics for these three potential pathways on the Ag-BTB interface and on the pristine Ag surface (Fig. 5f, g). The computational results reveal a distinct thermodynamic preference: the free energy required to form the precursors for the *OCCOH pathway (*CO + *COH) is merely 1.08 eV. This is significantly lower than the energies required for the alternative *CO + *CHO pathway (1.24 eV, leading to *OCCHO) and the direct *CO dimerization pathway (1.62 eV, leading to *OCCO). These calculated thermodynamic trends suggest the *COCOH route as the most favorable pathway, in qualitative agreement with our in situ FTIR observations. Guided by these thermodynamic findings, we further investigated the kinetic activation barriers along this optimal *COCOH pathway (Fig. 5h). The first proton-coupled electron transfer (PCET) to form *COH typically serves as the rate-determining step. On the pristine Ag (111) surface, this step exhibits a formidable kinetic barrier of 1.89 eV (TS1). The BTB-induced interfacial polarization transforms the *CO oxygen terminus into a nucleophilic center as discussed above. As a result, this critical TS1 barrier on the Ag-BTB surface is calculated to decrease to 1.43 eV. Following this accelerated protonation, the *COH species undergoes asymmetric C–C coupling with an adjacent *CO monomer (*CO + *COH → *OCCOH), navigating a low kinetic barrier (TS2) of only 0.23 eV. Therefore, both thermodynamic and kinetic analyses comprehensively demonstrate that the BTB functionalization not only intrinsically dictates the regioselectivity of the initial coupling but also significantly accelerates the *COCOH-mediated pathway toward multi-carbon products.

**Fig. 6:**
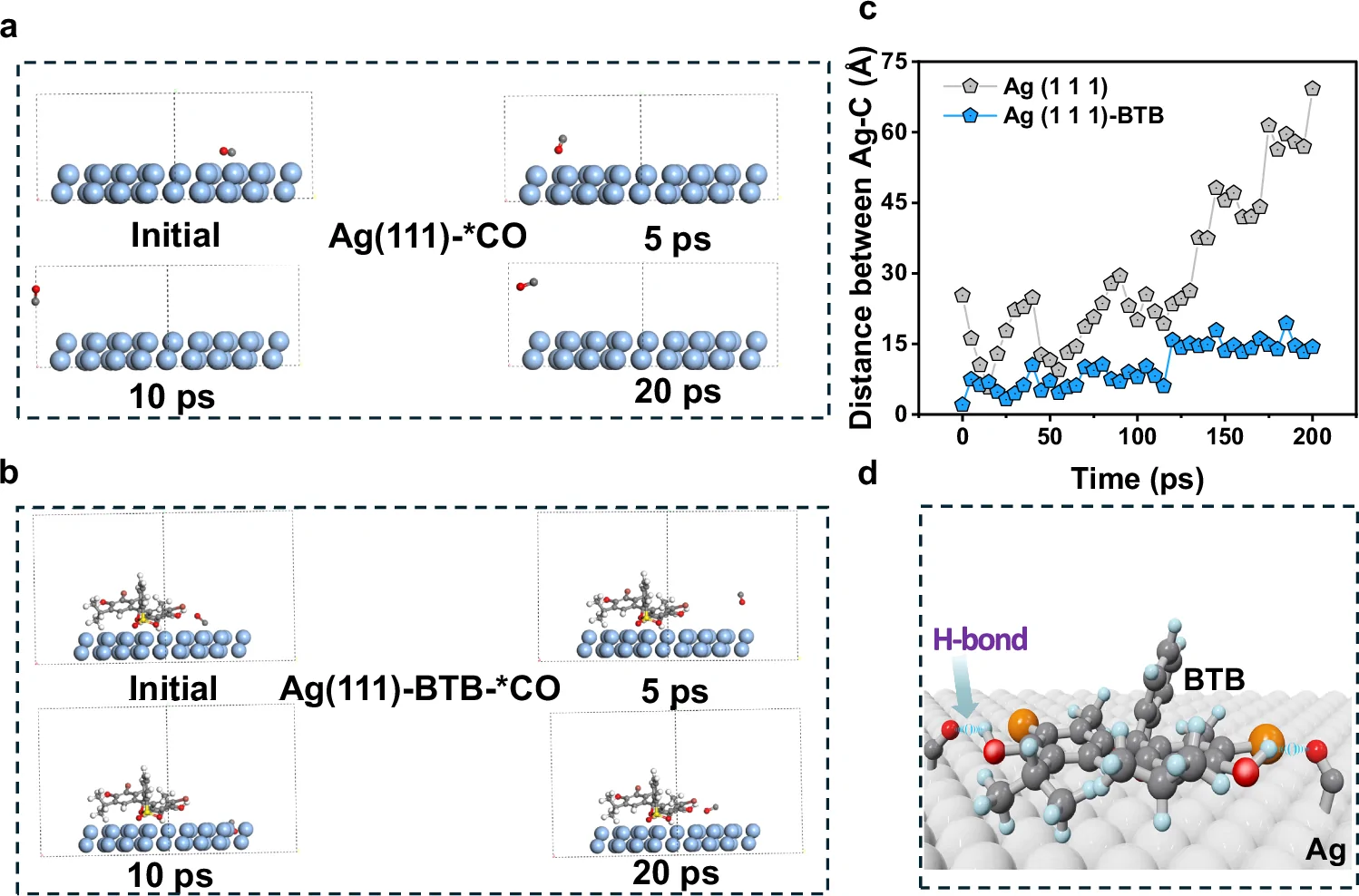
Molecular Dynamics (MD) Simulations. **a**, **b** Snapshot of spatial distribution of *CO intermediate for Ag (111) and Ag (111)-BTB systems obtained from MD simulations; Light blue, gray, red, yellow, reddish-brown, and white balls represent Ag, C, O, S, Br, and H atoms, respectively. **c** Statistical distribution of the interfacial distance between the C atom and the Ag (111), Ag (111)-BTB surfaces. **d** Schematic illustration for hydrogen-bond formation between *CO intermediate and BTB molecule. Source data are provided as a Source Data file.

Molecular dynamics simulations under constant volume and temperature (NVT) conditions reveal distinct stabilization behaviors of the *CO intermediate on pristine Ag (111) versus BTB-modified surfaces. To rigorously evaluate the dynamic stability of the adsorbed *CO, we tracked the vertical distances of both its C and O atoms relative to the Ag surface throughout the molecular dynamics (MD) trajectory (Fig. 6a–c and Supplementary Figs. S38, 39). The distance evolutions for both atoms exhibit highly consistent trends. Notably, the trajectories reveal that the *CO intermediate at the Ag-BTB interface is relatively confined within the interfacial microenvironment, remaining significantly closer to the electrode surface compared to pristine Ag. These geometric trends demonstrate that the BTB-modification strategy appears to suppress *CO desorption through non-covalent interactions. The prolonged surface residence time of *CO, as evidenced by these comparative simulations, allows for the subsequent hydrogenation and dimerization processes essential for multi‑electron product formation. Ultimately, these MD results provide atomic-scale visualization of how surface molecular functionalization alters interfacial dynamics. They suggest that hydrogen-bonding interactions extend the *CO adsorption lifetime, providing the kinetic stabilization required to drive the sequential elementary steps for hydrogenation and C-C coupling. These simulations provide molecular-level insights complementary to our spectroscopic observations and are consistent with direct interactions between the BTB modifier and the *CO intermediate. Such interfacial interactions are associated with changes in both the thermodynamic landscape (e.g., enhanced *CO adsorption energy) and kinetic behavior (e.g., prolonged *CO residence time) at the interface. It should be noted that the present DFT and MD simulations are based on idealized surface models and do not explicitly account for the full structural complexity of the catalyst/electrolyte interface under operando electrochemical conditions, including dynamic solvent effects, surface reconstruction, electric-field effects, and potential-dependent adsorbate coverage. Accordingly, these calculations primarily provide qualitative mechanistic insights into the interfacial electronic interactions and reaction trends. Collectively, these observations suggest reduced kinetic barriers for the subsequent reduction of *CO, in a manner consistent with pathways toward multi-electron product formation (Fig. 6d).

## Discussion

In summary, this study demonstrates a surface molecular functionalization strategy to reconstruct the interfacial hydrogen-bonding network on silver catalysts. By anchoring BTB molecules onto the Ag surface, the introduced hydroxyl groups establish hydrogen bonds with the transient *CO intermediate. This interaction significantly suppresses *CO desorption and diffusion, facilitating subsequent hydrogenation and C-C coupling steps. Consequently, this interfacial engineering approach successfully unlocks the capacity of Ag to generate multi-electron products during electrochemical CO_2_ reduction, achieving a  FE of 24.2% at a current density of 400 mA cm^−2^. These findings show that Ag-based materials are promising for deep CO_2_ reduction, and that microenvironment engineering (beyond electronic tuning) offers a viable path to efficient non-Cu catalysts.

## Methods

### Chemicals and materials

Silver slugs (2 mm diameter/5 mm length, 99.999%) were purchased from PrMat Co., Ltd. Carbon paper (30 T) was purchased from Suzhou Sinero Technology Co., Ltd. Bromothymol blue (BTB), Phenolphthalein (PP), phenyl 4-bromobenzenesulfonate (PBBS), polyethylene glycol (PEG), and triphenylmethane (TPM) were all purchased from Aladdin Reagent (Shanghai) Co., Ltd. All materials were used directly without further purification.

### Synthesis of catalysts

The catalyst substrate was prepared using commercial carbon paper (Toray 30 T) as the base material. Initially, a uniform thin silver (Ag) film was deposited onto the carbon paper via thermal evaporation in a high-vacuum deposition system. Prior to evaporation, the chamber was evacuated to a base pressure below 10 Pa to minimize potential contamination. High-purity silver (99.999%) was thermally evaporated from a tungsten boat at a strictly controlled deposition rate of 1 Å s^−1^ to ensure the formation of a homogeneous Ag film with a targeted thickness of 100 nm.

Following the physical deposition process, the Ag-coated carbon paper was further functionalized with molecular modifiers to enhance its electrocatalytic performance. For the preparation of the BTB-functionalized electrodes (denoted as Ag-BTB), a series of aqueous BTB solutions with varying concentrations of 0.1, 0.19, and 0.38% (w/v) were prepared and subjected to ultrasonication to ensure complete dissolution. The pristine Ag substrates were then immersed in these specific BTB solutions and kept undisturbed for 10 min at room temperature (25 °C). This precisely controlled immersion step ensures sufficient and uniform molecular adsorption onto the catalyst surface. Subsequently, the freshly functionalized electrodes were carefully removed and completely dried under an infrared lamp to obtain the final Ag-BTB catalysts.

For comparative structure-activity studies, a series of control catalysts modified with different organic molecules were synthesized strictly following the identical protocol. These molecules include phenyl 4-bromobenzenesulfonate (Ag-PBBS), polyethylene glycol (Ag-PEG), phenolphthalein (Ag-PP), and triphenylmethane (Ag-TPM). They were prepared using the same standard mass/volume concentration (w/v) under the exact same immersion time, temperature, and drying conditions to ensure a standardized molecular loading across all experimental groups.

### Characterizations

The surface morphologies were examined by field emission scanning electron microscopy (FE-SEM, JSM-7800F) and transmission electron microscopy (TEM, JEM-F200). Spherical aberration-corrected high-angle annular dark-field scanning transmission electron microscopy (AC HAADF-STEM, Titan Cubed Themis G2 300) was further performed to obtain atomic structural characterization. X-ray diffraction (XRD) patterns were recorded on the X-ray diffractometer of PANalytical Empyrean with Cu Kα radiation (λ = 1.54178 Å). X-ray photoelectron spectra analysis (XPS) was  performed on ThermoVG Scientific ESCALAB 250. Infrared spectra were achieved by a Fourier transform infrared spectrometer (FT-IR, SHIMADZU). The X-ray absorption spectra (XAS) were collected at the beamline BL14W1 station of the Shanghai Synchrotron Radiation Facility.

In situ differential electrochemical mass spectrometry (DEMS) measurements were conducted using a commercial Linglu Instruments QAS100 system coupled with a CHI660E electrochemical workstation. The customized three-electrode electrochemical cell (Supplementary Fig. S13) employed a hydrophobic carbon paper coated with the catalysts as the working electrode, a platinum wire as the counter electrode, and a saturated Ag/AgCl as the reference electrode. The electrolyte was 1.0 M KOH (pH = 13.9 ± 0.1). A Polytetrafluoroethylene (PTFE) membrane (pore size ≤ 20 nm, thickness 40 µm, Shanghai Maohang Biotechnology Co., Ltd.) was utilized to separate the liquid electrolyte from the mass spectrometer vacuum chamber. For isotope experiments, ^13^CO_2_ was used as the feed gas. Gaseous products generated on the electrode surface during the test were directed into a mass spectrometer via a vacuum pump and detected under a specific mass/charge ratio. The ion currents are plotted directly without any deconvolution.

In situ FT-IR spectra measurements were performed with Bruker INVENIO-S. Testing was conducted in a homemade in situ cell, with Ag/AgCl used as a reference. All spectra were presented as ΔR/R = (Es-E_R_)/E_R_, where Es and E_R_ represent the sample and background spectra, respectively. In situ Raman measurements were conducted in a custom-made electrochemical flow-cell with a three-electrode configuration. During the In situ Raman test process, to enhance signal intensity, an excitation laser of 532 nm was used as the light source. The spectrum was collected after each potential (from − 0.4 V _RHE_ to − 1.6 V _RHE_).

### Electrochemical measurements

The CO_2_ electro-reduction measurements were conducted in a custom-designed flow cell with an electrochemical workstation (CHI1140D). An anion exchange membrane was used to separate the catholyte and anolyte, facilitating the transfer of ions while attenuating product crossover. The prepared gas diffusion electrode, nickel foam, and calibrated Ag/AgCl (saturated KCl) served as the working electrode, counter electrode, and reference electrode, respectively. During the tests, 1.0 M KOH (pH = 13.9 ± 0.1) was pumped into the anodic and cathodic chambers via a peristaltic pump under a constant flow rate of 8 mL min^−1^. CO_2_ gas with a flow rate of 20 mL min^−1^ was fed into gas chamber by a mass flowmeter. No IR compensation was employed for all electrochemical tests. All the potentials used were based on the reversible hydrogen electrode (RHE) using the following equation:

1 $${E}_{{RHE}}={E}_{{Ag}/{AgCl}}+0.197+0.0591\times {pH}$$

### Product analysis

The gas products were monitored by an online gas chromatograph (Shimadzu, GC-2014) equipped with a thermal conductivity detector (TCD) and a flame ionization detector (FID). The signal of H_2_ was obtained from TCD, while the CO, CH_4_, and other alkane contents were detected by FID with a methane reformer. The Faradaic efficiency of gas products was calculated as the following formula:

2 $${FE}\left(\%\right)=\frac{C\times u\times z\times F}{{V}_{m}\times I}\times 100\%$$

where *C* is the concentration of gas products (ppm), *u* is the flow rate of CO_2_ (mL min^−1^), *z* is the number of transferred electrons, *F* is the Faradaic constant (96485 C mol^–1^), *V*_*m*_ is the molar volume of gas, and *I* is the applied current.

The liquid products were determined by ^1^H NMR (Bruker AVANCE III 500 MHz) with a water suppression program. In detail, 100 μL of D_2_O and 100 μL of DMSO (served as an internal standard) were added into 400 μL of catholyte for the quantification of liquid products. Similarly, the calculation of Faradaic efficiency for liquid products was given as follows:

3 $${FE}\left(\%\right)=\frac{n\times z\times F}{I\times t}\times 100\%$$

where *n* is the mole number, *z* is the number of transferred electrons, *F* is the Faradaic constant, *I* is the applied current, and *t* is the electrolysis time.

### DFT calculation

All first-principles density functional theory (DFT) calculations were performed using the Vienna Ab initio Simulation Package (VASP) within the *MedeA VASP* platform (VASP developed at the Universität Wien)^48–50^. The electron exchange-correlation interactions were treated within the generalized gradient approximation using the Perdew-Burke-Ernzerhof functional (GGA-PBE) with Grimme’s D3 dispersion correction, while ion-electron interactions were described by the projector augmented wave (PAW) method. We employed a plane-wave basis set with a kinetic energy cutoff of 450 eV. For Brillouin zone integration, a 3 × 3 × 1 Monkhorst-Pack k-point mesh was used during structural optimization. Structural optimizations were performed until the energy change between two electronic self-consistent steps was less than 10^−5^ eV, and the Hellmann-Feynman forces on all atoms were converged to below 0.02 eV Å^−1^.

The adsorption energy (${E}_{{ads}}$)was defined according to the equation:

4 $${E}_{{\mbox{ads}}}={E}_{{\mbox{substrate}}+{\mbox{adsorbate}}}-{E}_{{\mbox{substrate}}}-{E}_{{\mbox{adsorbate}}}$$

Where ${E}_{{\mbox{substrate}}+{\mbox{adsorbate}}}$ is the total energy of the slab with the adsorbate, ${{{{\rm{E}}}}}_{{{{\rm{substrate}}}}}$ is the energy of the clean optimized Ag or Ag-BTB slab, and ${E}_{{\mbox{adsorbate}}}$ is the energy of the isolated adsorbate molecule in the gas phase. By this definition, a more negative *E*_*ads*_ value indicates a stronger binding interaction. The transition state (TS) structures and reaction pathways were determined using the climbing image nudged elastic band (CI-NEB) method.

To investigate the electrochemical reaction pathways, the free energy change (Δ*G*) for each elementary step involving coupled proton-electron transfer was calculated within the framework of the computational hydrogen electrode (CHE) model. In this model, the chemical potential of the (H^+^ + e^−^) pair is referenced to half of the chemical potential of gas-phase H_2_ at standard conditions (pH = 0, U = 0 V, T = 298.15 K). Accordingly, the free energy was calculated as:

5 $$\Delta G=\Delta E+\Delta {E}_{{ZPE}}-T\Delta S+\Delta {G}_{{pH}}+\Delta {G}_{U}$$

Where Δ*E* denotes the electronic energy difference obtained from DFT calculations. Δ*G*_pH_ = −k_B_Tln_10 _× pH accounts for the pH dependence, and ΔG_U_ = −ne*U* accounts for the effect of the electrode potential *U*. In this work, Δ*E*_*ZPE*_ and Δ*S* were derived from vibrational frequency calculations and standard thermodynamic data, respectively. The temperature T was set to 298.15 K for all reaction systems.

The charge density differences were analyzed by the VASPKIT code. In order to quantitatively analyze the bonding properties of CO adsorbed on Ag-BTB and characterization of charge transfer, the charge densities of CO in the corresponding compounds were extracted from the corresponding charge densities in the Ag-BTB substrates to assess the differences in the charge densities of CO adsorbed on the constructed Ag-BTB models. The level of charge transfer between CO and Ag-BTB was calculated using a Bader charge analysis program.

6 $$\Delta \rho=\rho \left({CO}-{Ag}-{BTB}\right)-\rho \left({Ag}-{{{\rm{BTB}}}}\right)-\rho \left({CO}\right)$$

Independent gradient model based on Hirshfeld partition (IGMH)^51^ simulations were performed with the Multiwfn program to investigate the type of interaction force. H-bonding interactions between CO and Ag-BTB are revealed  by negative sign(λ_2_)ρ values.

### Molecular dynamics (MD) simulations

Molecular dynamics (MD) simulations were performed using the Forcite module with the COMPASS force field. The simulation systems comprised a periodic Ag(111) slab model, a layer of explicitly modeled BTB molecules (for the modified system), and a CO molecule. After geometric optimization, the systems were equilibrated under the NPT ensemble for 200 ps. Subsequently, the production runs were performed under the NVT ensemble for 200 ps to collect the dynamic trajectory data. Temperature was regulated using a Nosé-Hoover thermostat. Electrostatic and van der Waals (vdW) interactions were treated using the Ewald summation and an atom-based cutoff method (12.5 Å radius), respectively. The vertical trajectory distance between the carbon atom of CO and the uppermost layer of the Ag(111) surface was systematically extracted to evaluate dynamic spatial confinement.

## Supplementary information

Supplementary Information

Description of Additional Supplementary Files

Supplementary Data 1

Transparent Peer Review file

## Source data

Source Data

## Data Availability

The authors declare that all data supporting the findings of this study are available within the paper and its supplementary information files. Source data are provided in this paper.
