# Supplementary material for: Hydrogen bond stabilized *CO intermediate enables CO2 electroreduction to multi-electron products on silver catalysts
Source: Nat Commun. 2026 Jun 25;17:8700. doi: 10.1038/s41467-026-73970-9 (PMC13490397; doi:10.1038/s41467-026-73970-9)
Supplement: Supplementary file 1 [file 41467_2026_73970_MOESM1_ESM.pdf]

Supplementary Information for

Hydrogen Bond Stabilized *CO Intermediate Enables CO_2 Electroreduction to Multi-Electron Products on Silver Catalysts

Weiluo Zhang, Yilei Zhang, Da Wan, Junrong Gao, Shuling Zheng, Jie He, Huizhu Cai, Xue Zhang, Qi Hu, Hengpan Yang*, Chuanxin He*

College of Chemistry and Environmental Engineering, Shenzhen University, Shenzhen, Guangdong, 518060, China

* Corresponding Authors:

E-mail: hpyang@szu.edu.cn (H.P. Y.); hecx@szu.edu.cn (C.X. H.)

Contents

Figures S1-S39 and Table S1

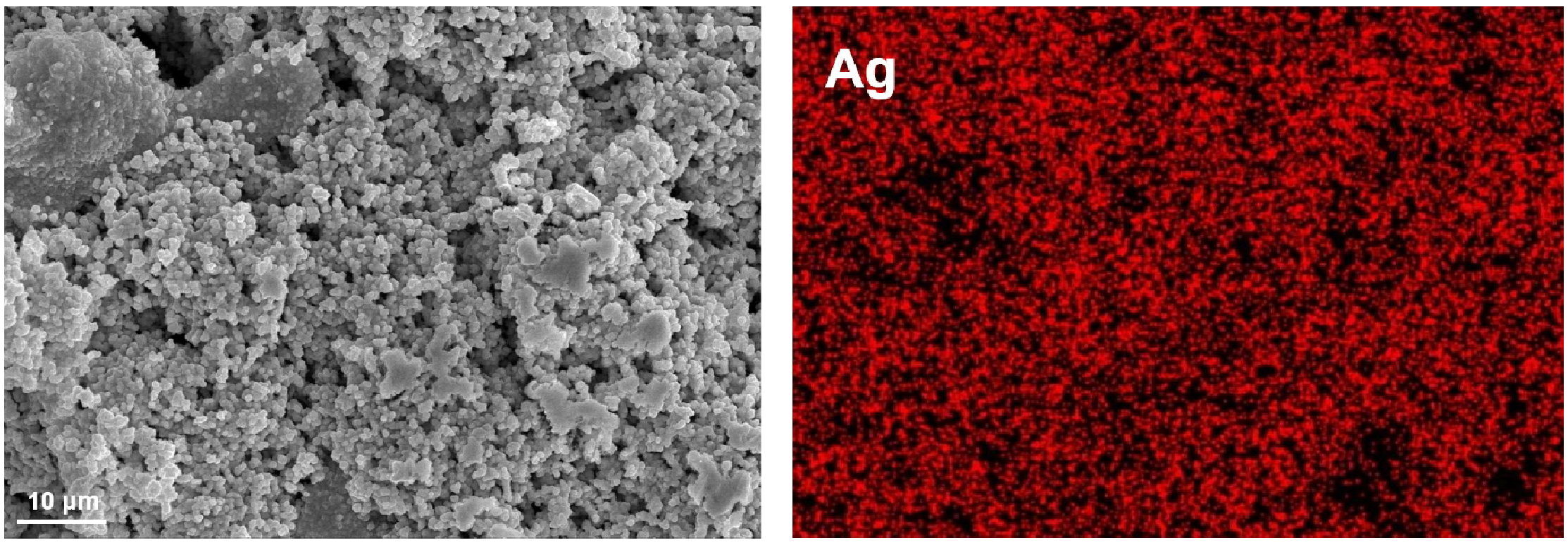

Figure S1. SEM images and the EDS mappings of Ag.

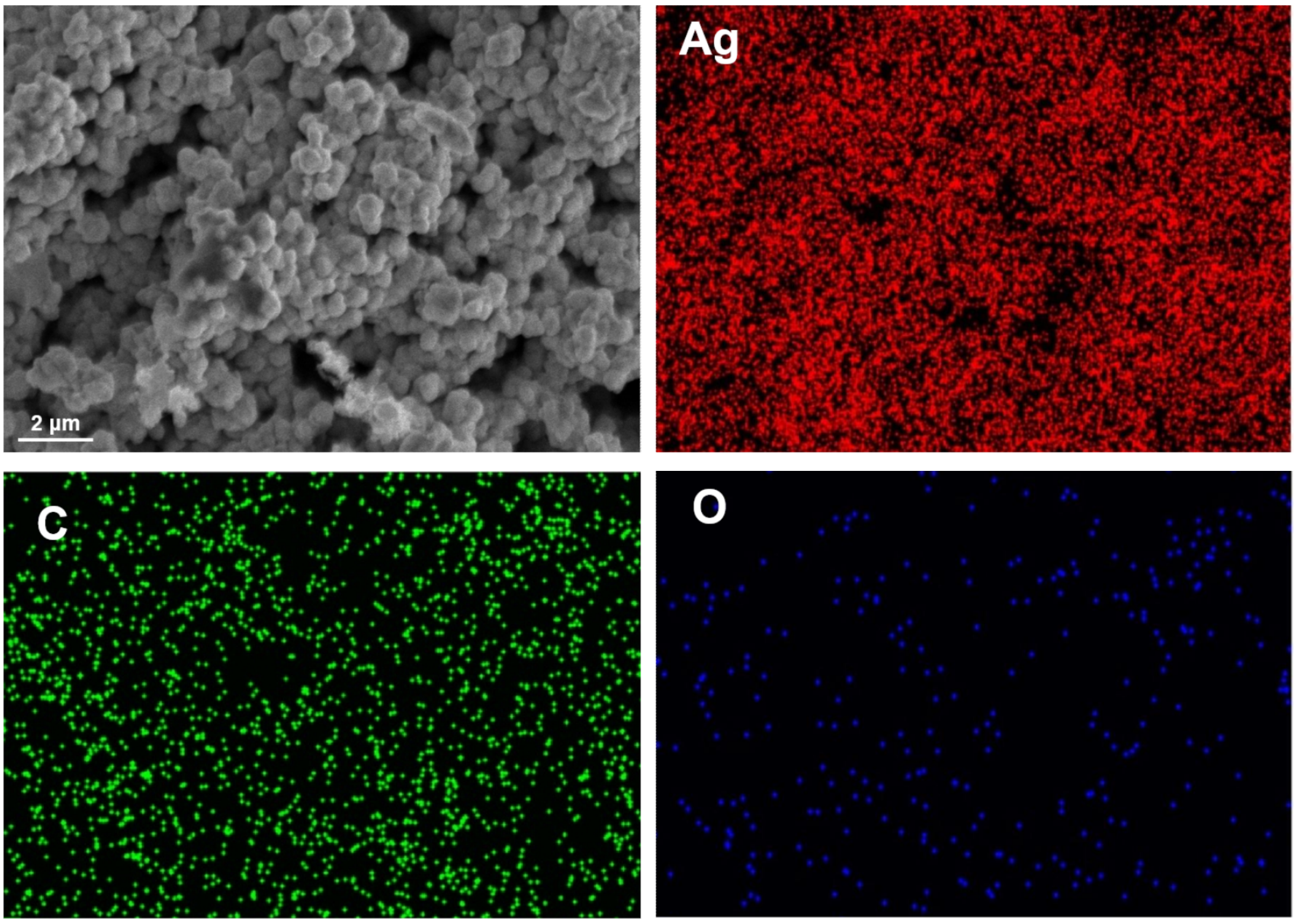

Figure S2. SEM images and the EDS mappings of Ag-BTB.

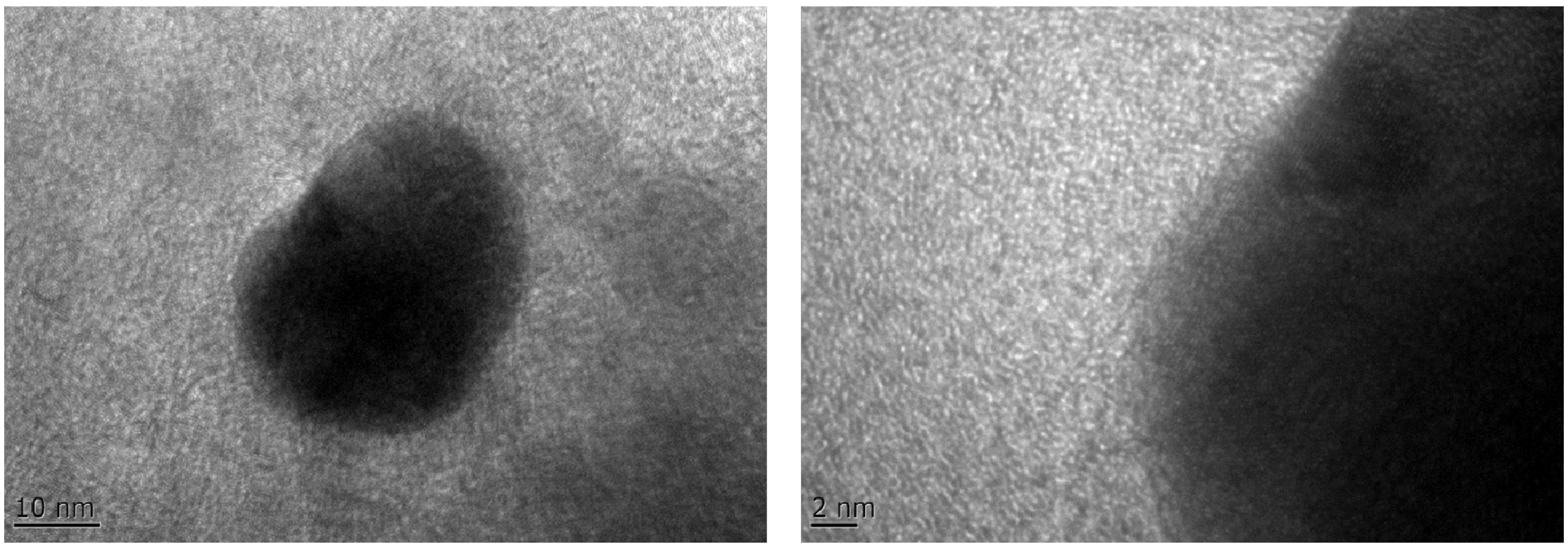

Figure S3. TEM images of Ag.

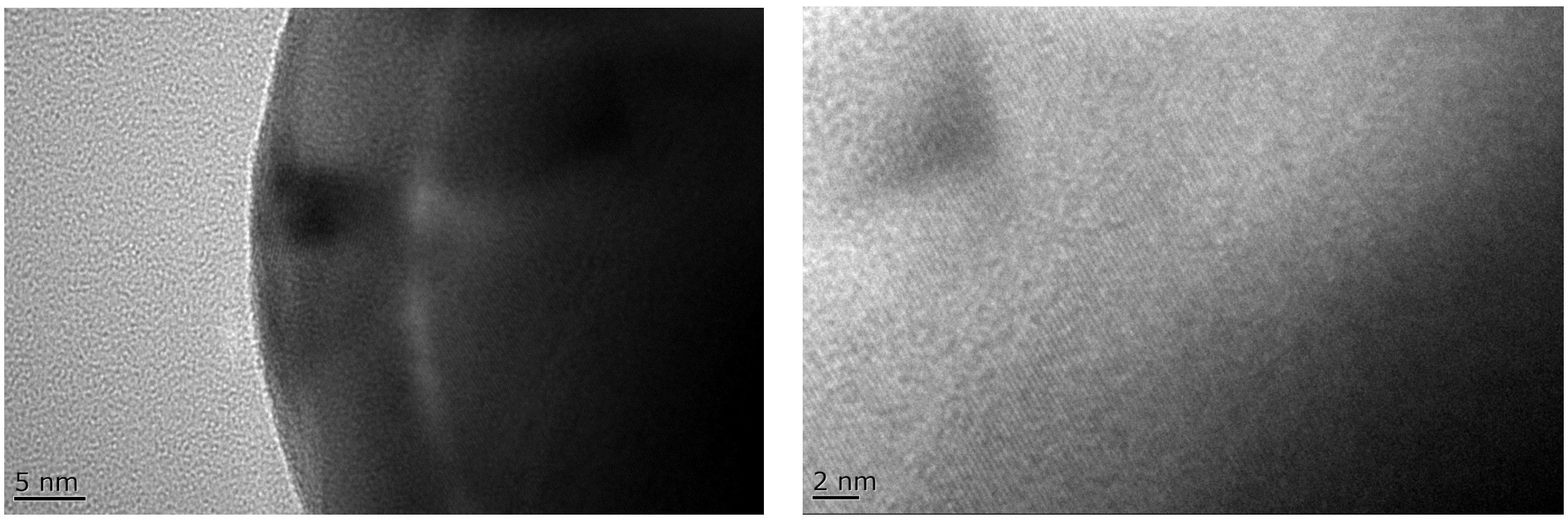

Figure S4. TEM images of Ag-BTB.

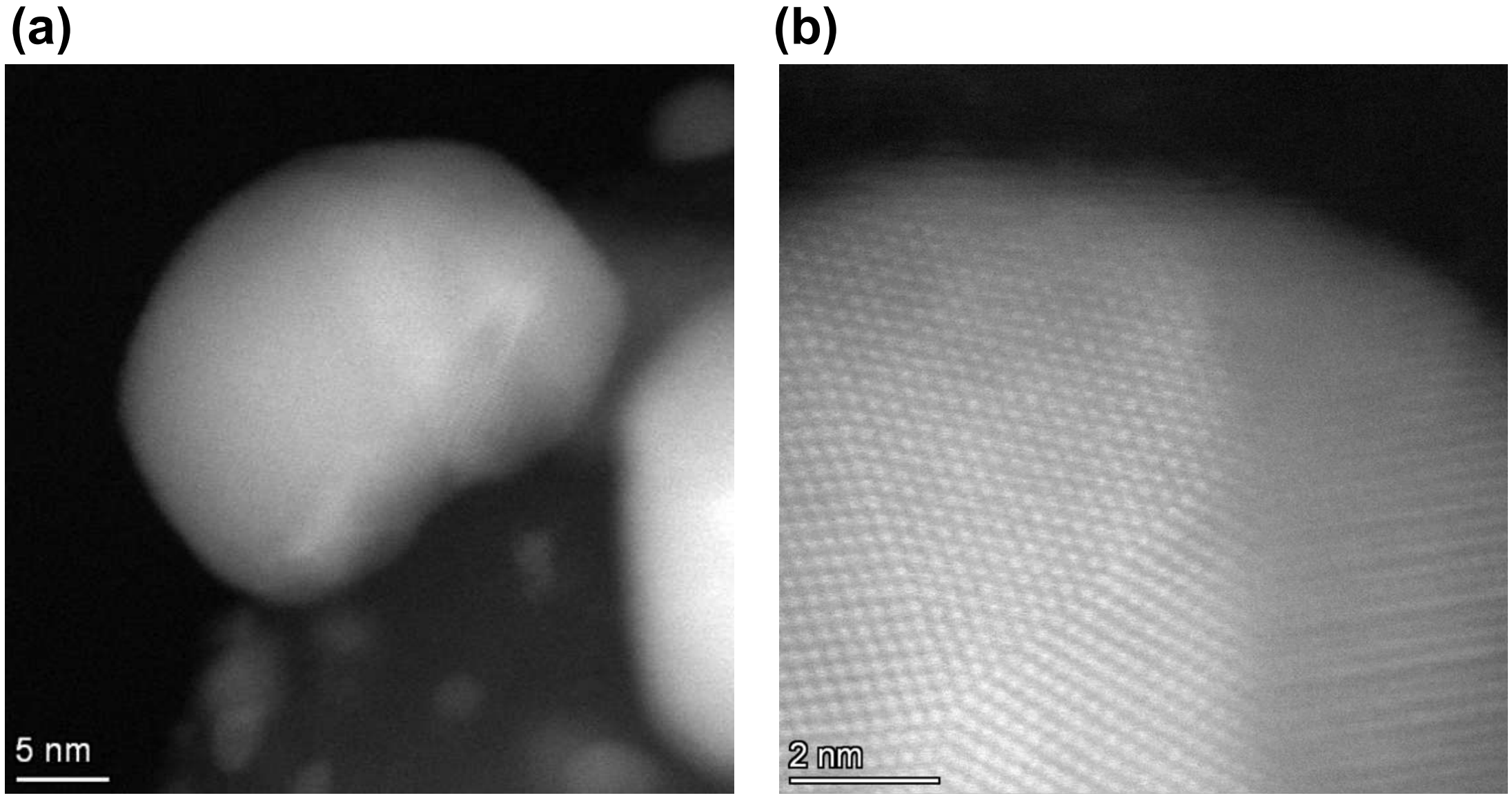

Figure S5. Aberration-Corrected HAADF-STEM images of Ag-BTB.

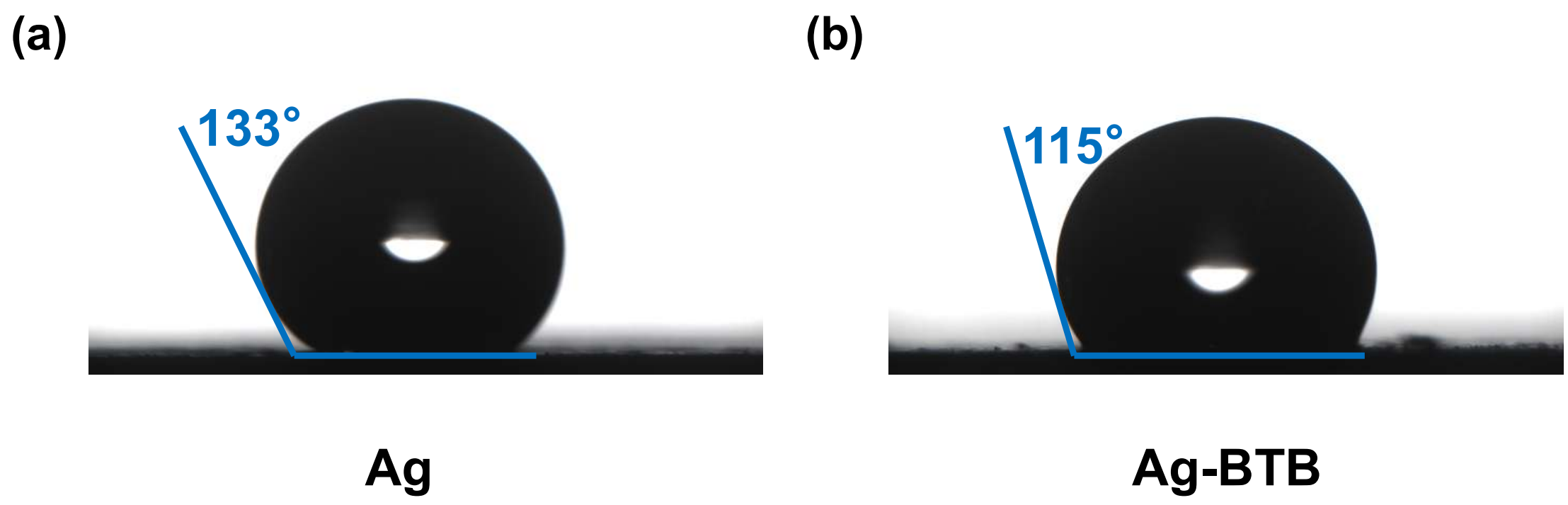

Figure S6. Contact angle measurement of (a) Ag and (b) Ag-BTB.

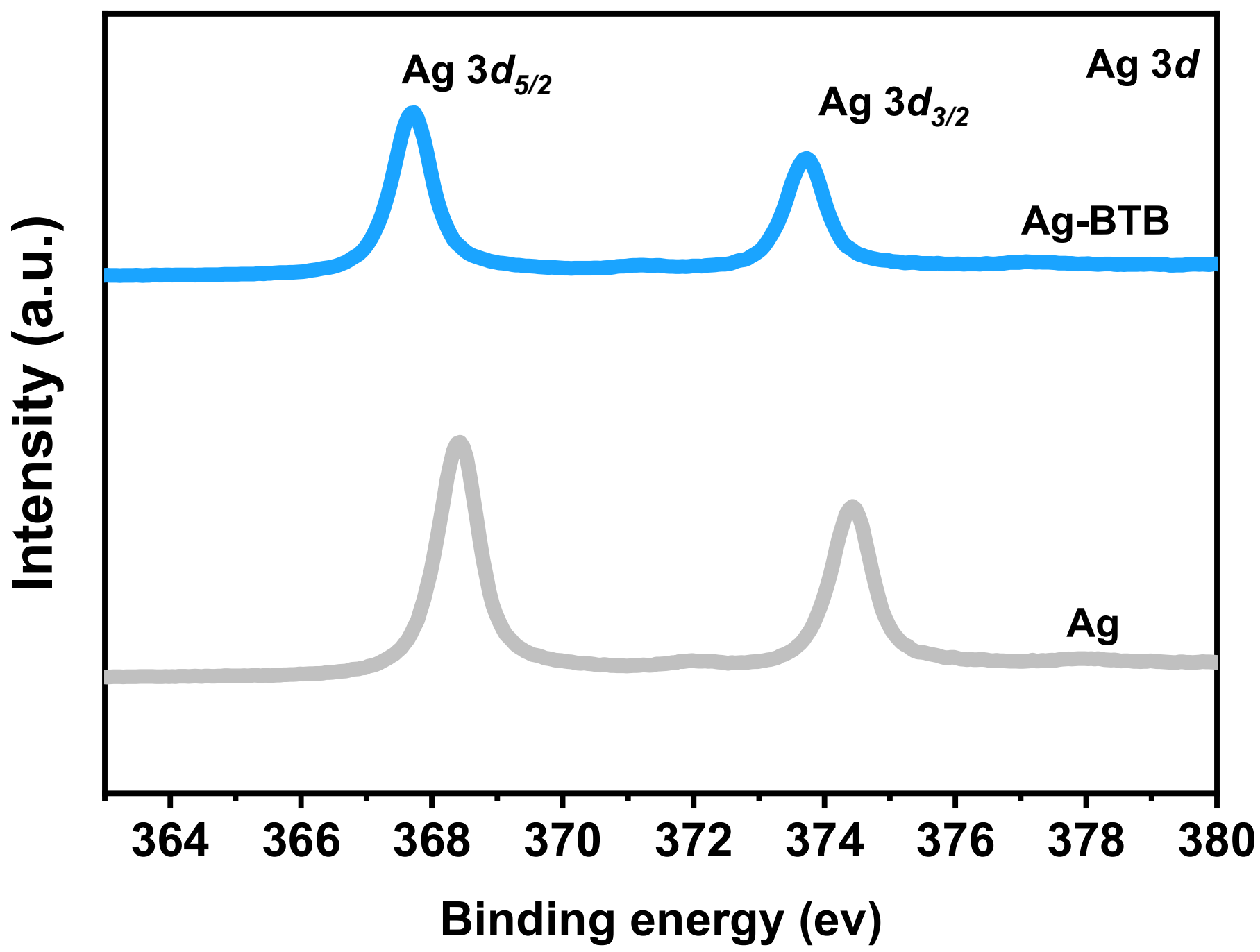

Figure S7. Ag 3*d* XPS spectra of Ag and Ag-BTB.

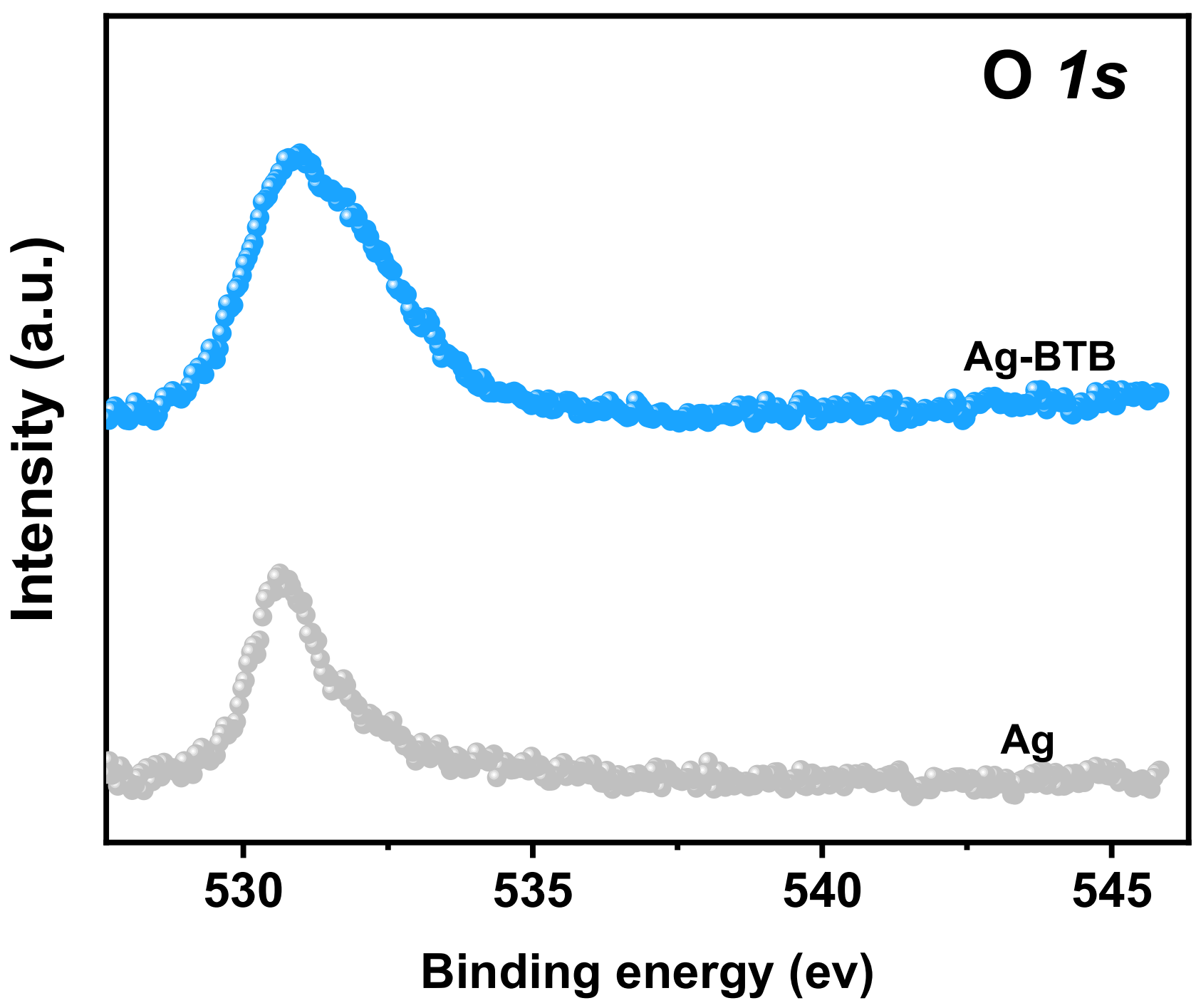

Figure S8. O 1*s* XPS spectra of Ag and Ag-BTB.

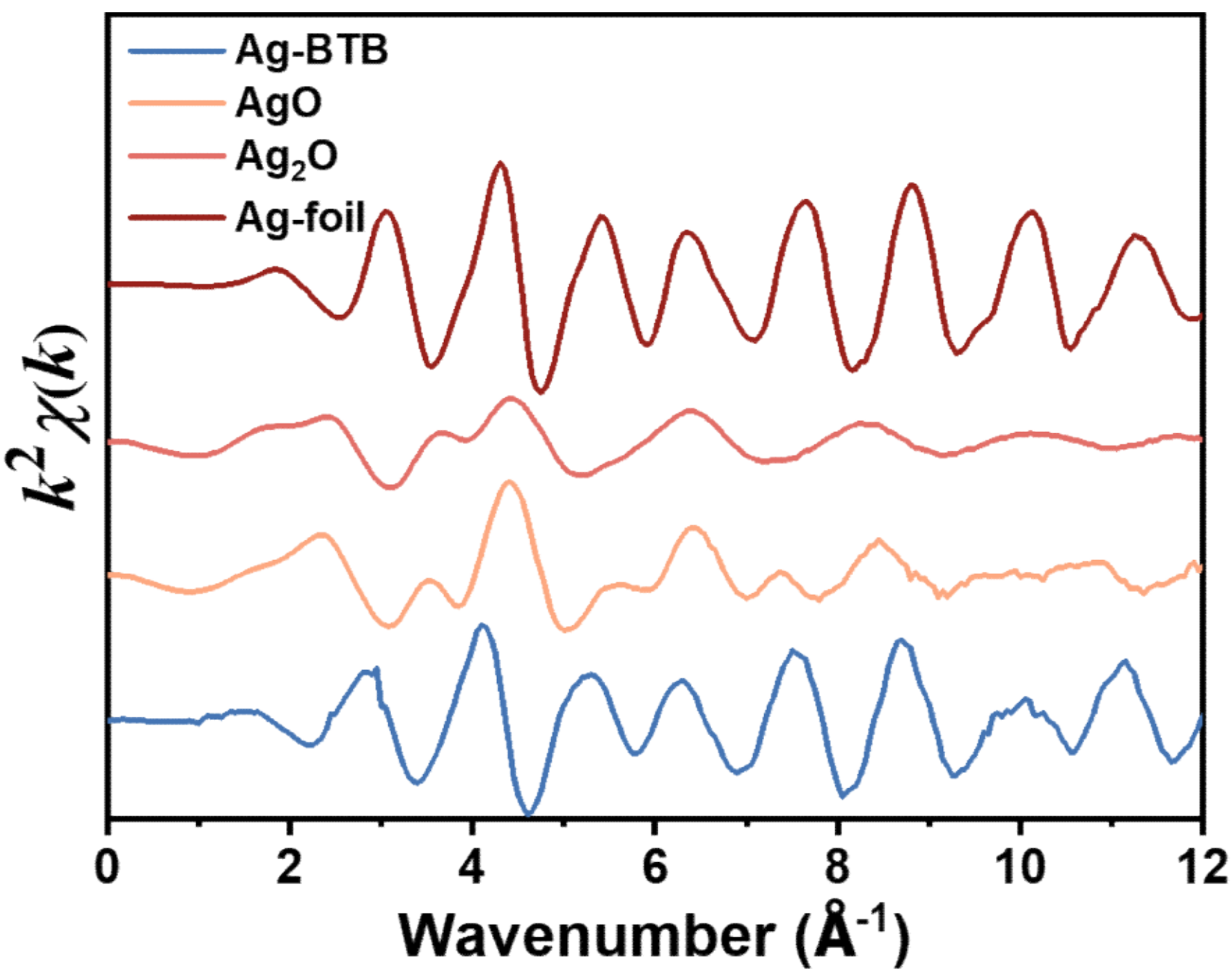

Figure S9. (a) FT-EXAFS spectra in the *K* space of Ag-BTB and reference Ag foil, AgO and Ag_2O.

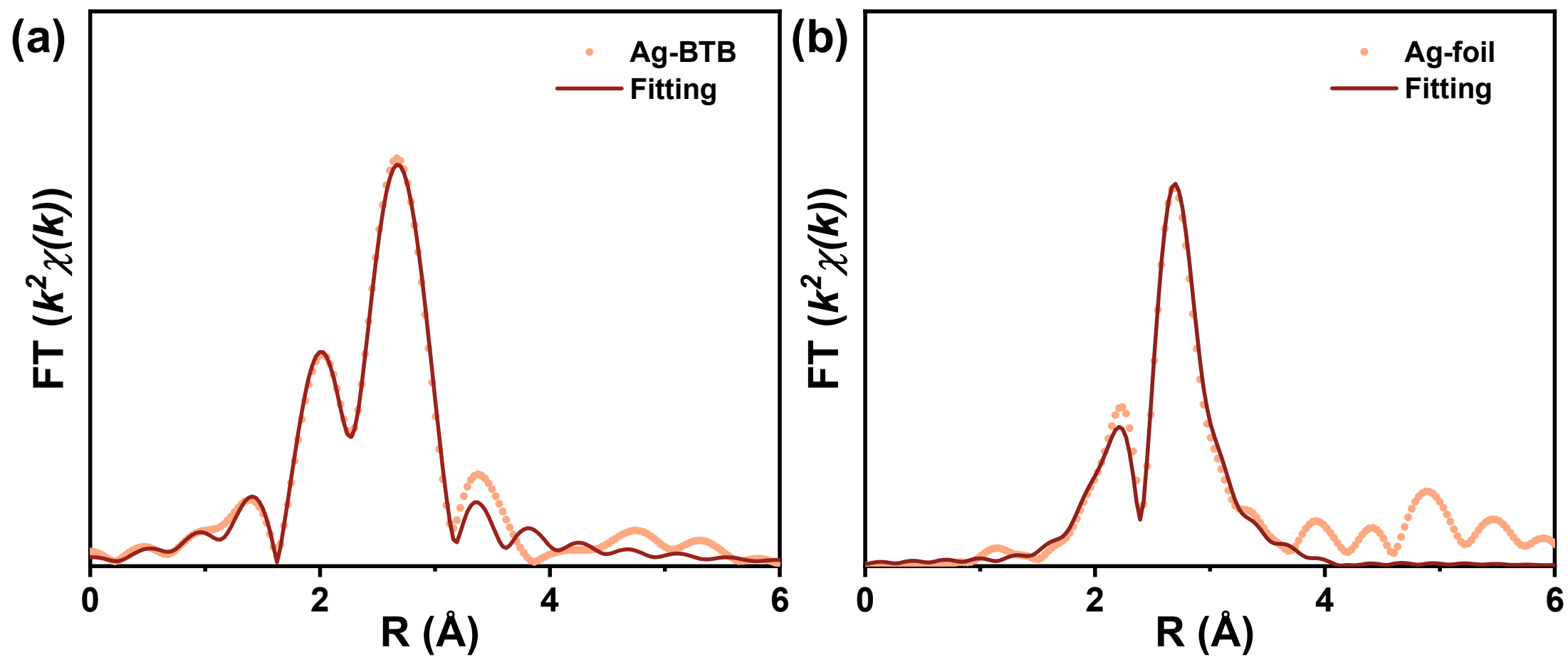

Figure S10. FT-EXAFS fitting curves in the *R* space of (a) Ag-BTB and (b) Ag-foil

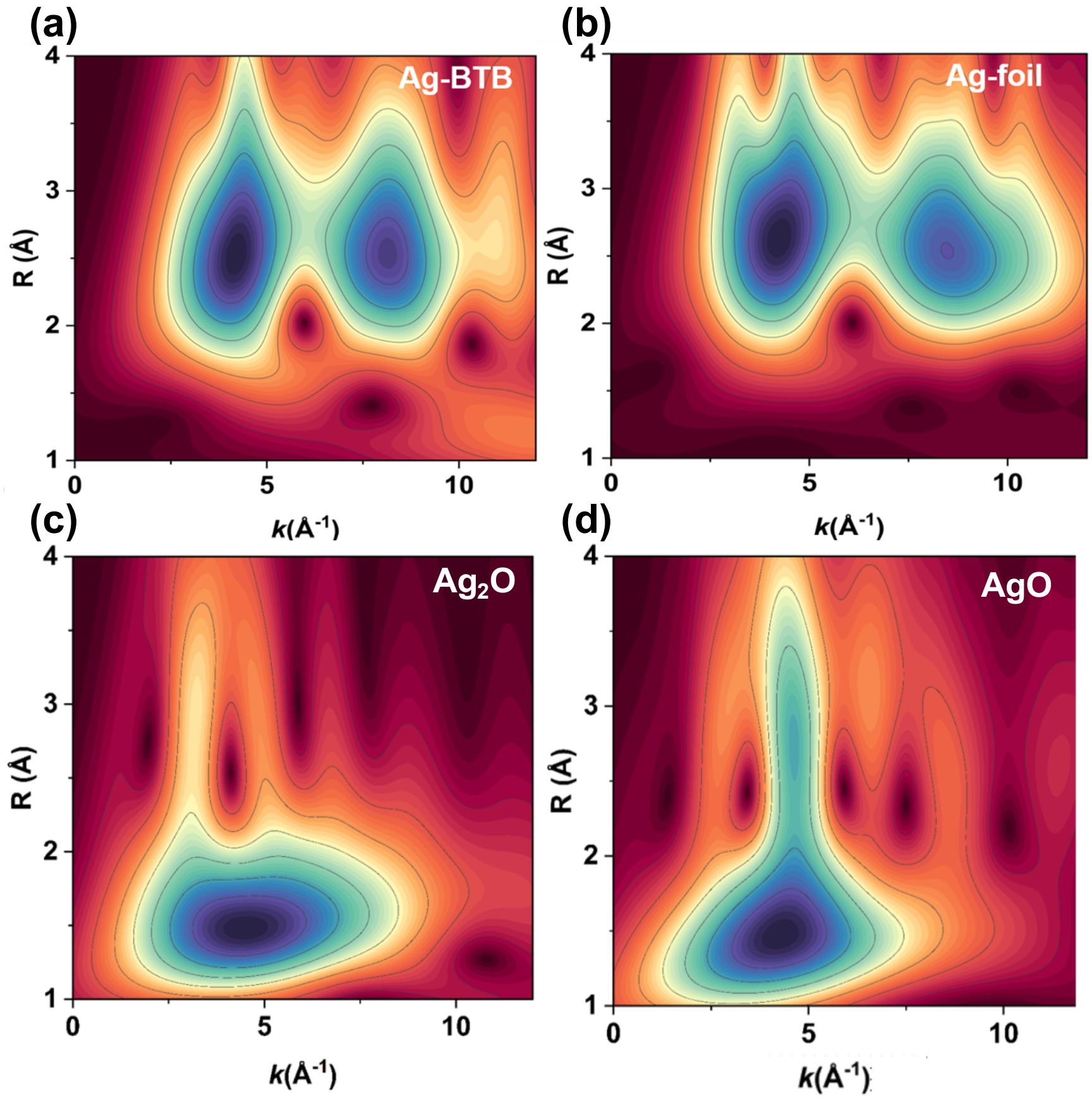

Figure S11. Wavelet transforms of the EXAFS signals for (a) Ag-BTB, (b) Ag foil, (c) Ag_2O and (d) AgO.

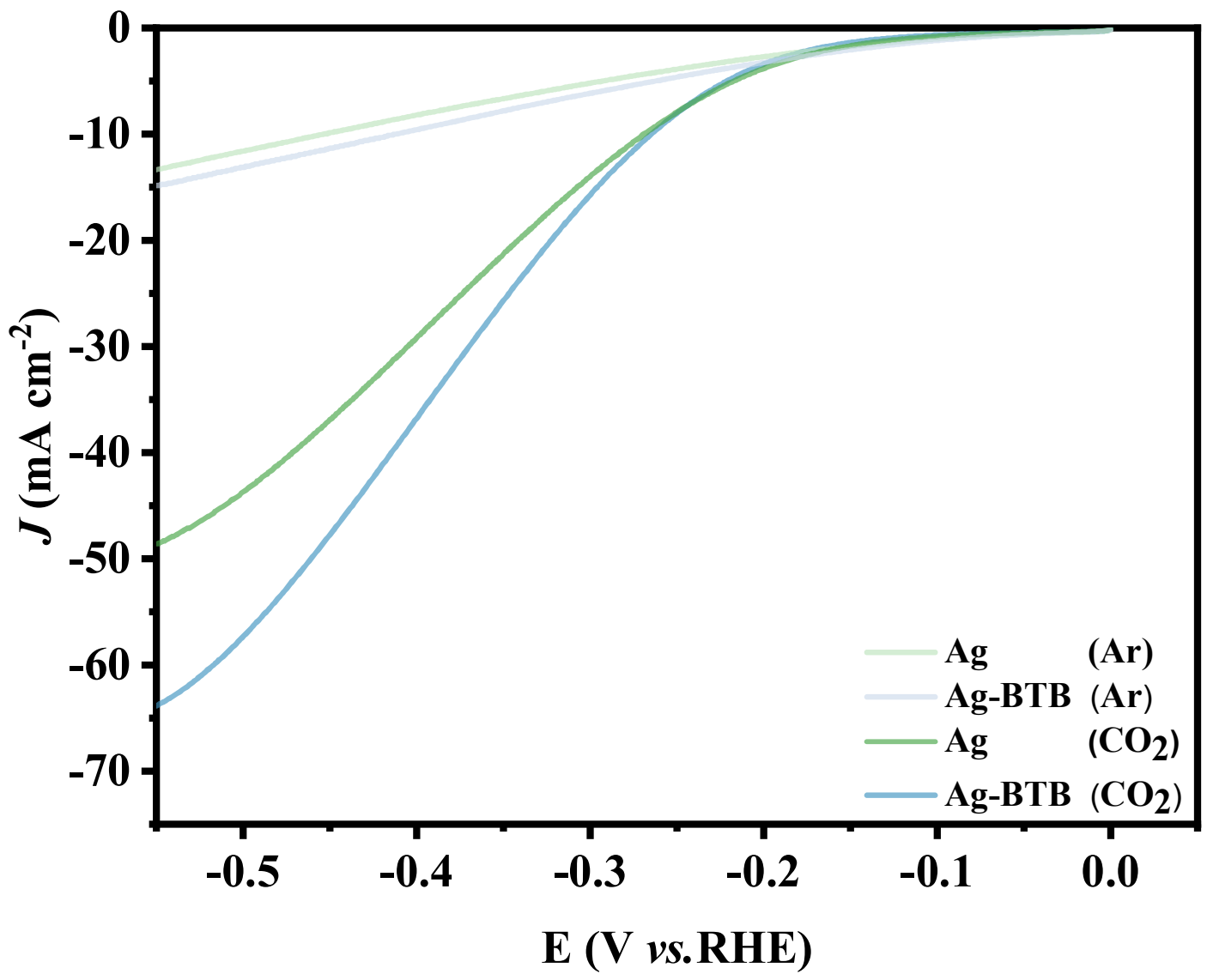

Figure S12. LSV curves of Ag and Ag-BTB tested in flow cell saturated with Ar and CO_2 respectively.

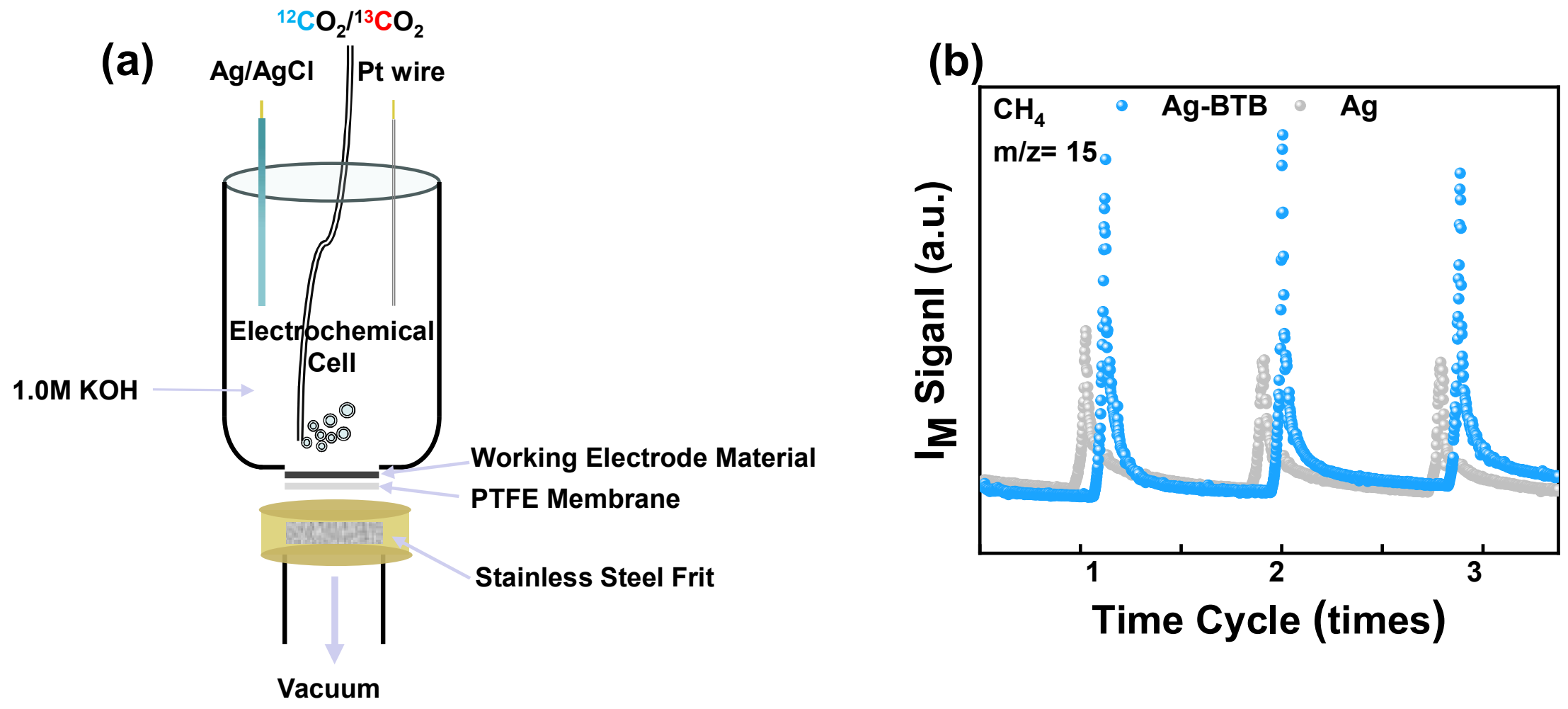

Figure S13. (a) Schematic illustration of the in situ Differential Electrochemical Mass Spectrometry (DEMS) cell configuration. (b) In-situ DEMS data obtained during CO_2RR on Ag and Ag-BTB catalysts, ionic currents of CH_3^+.

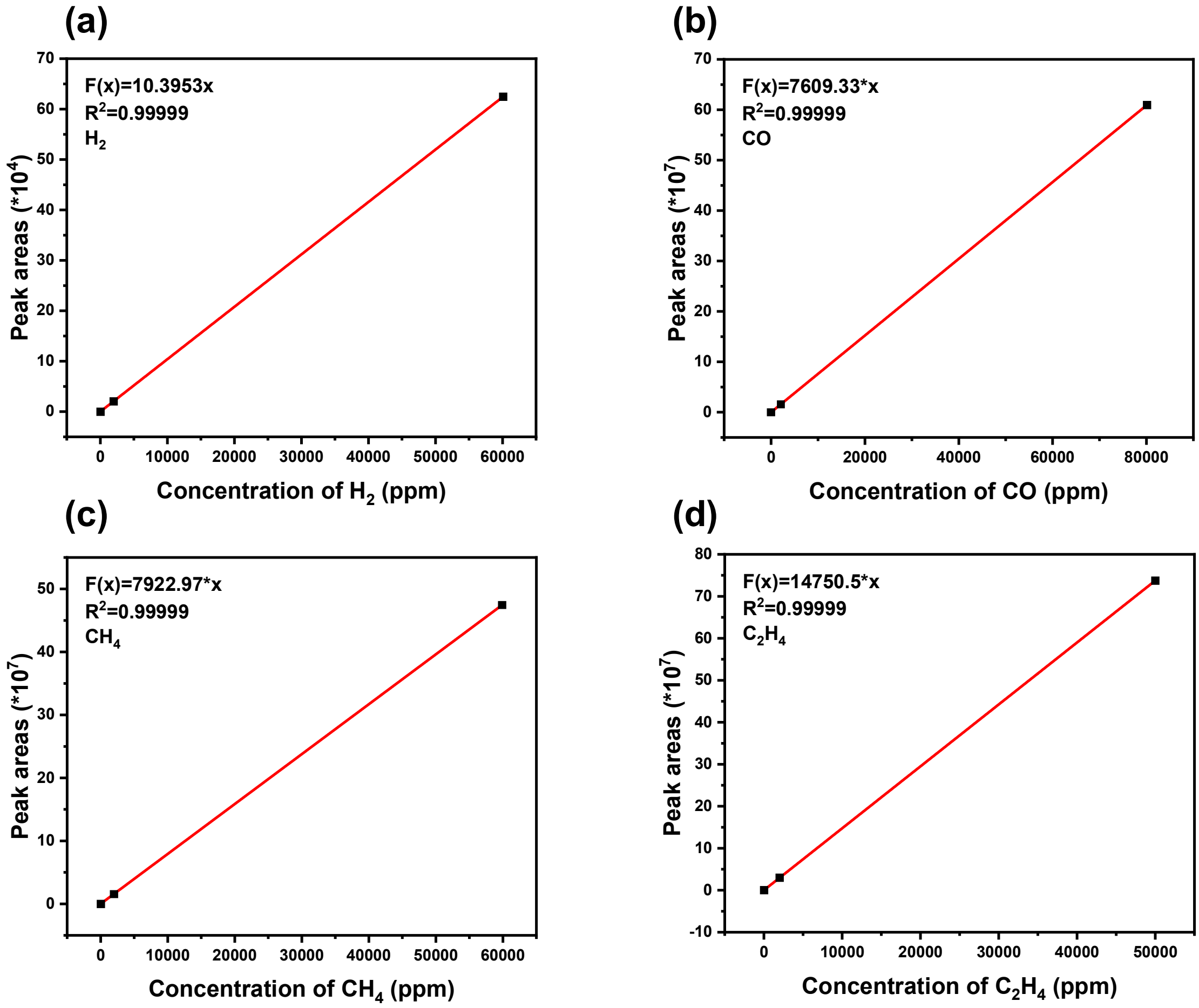

Figure S14. The method for quantitative analysis of gas products. The corresponding calibration curves for H_2(a), CO (b), CH_4(c), and C_2H_4(d) components were determined by gas chromatography.

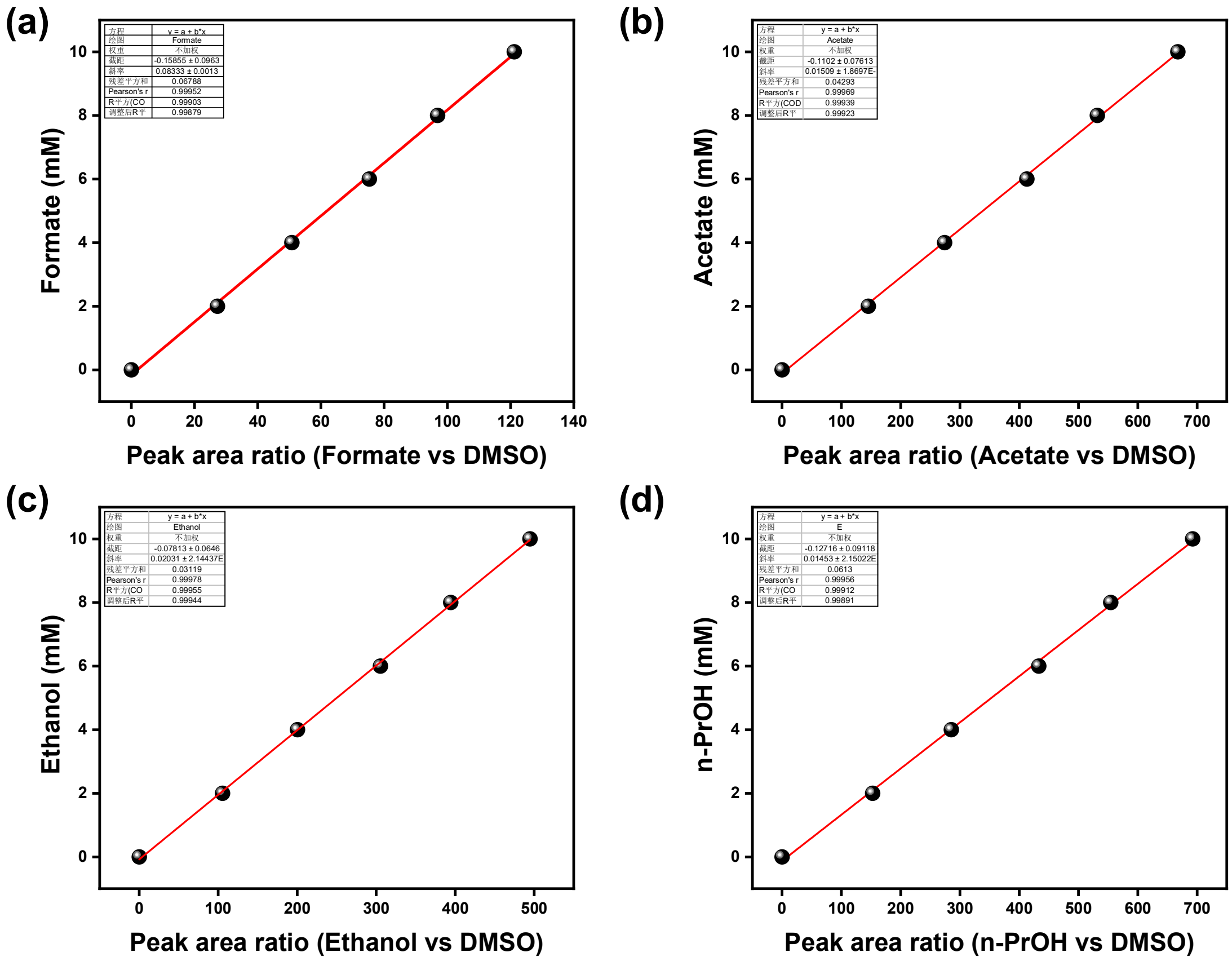

Figure S15. The method for quantitative analysis of liquid products. The standard curves for formate (a), acetate (b), ethanol (c), and n-propanol (d) ingredients were confirmed by means of ^{1}H NMR tests.

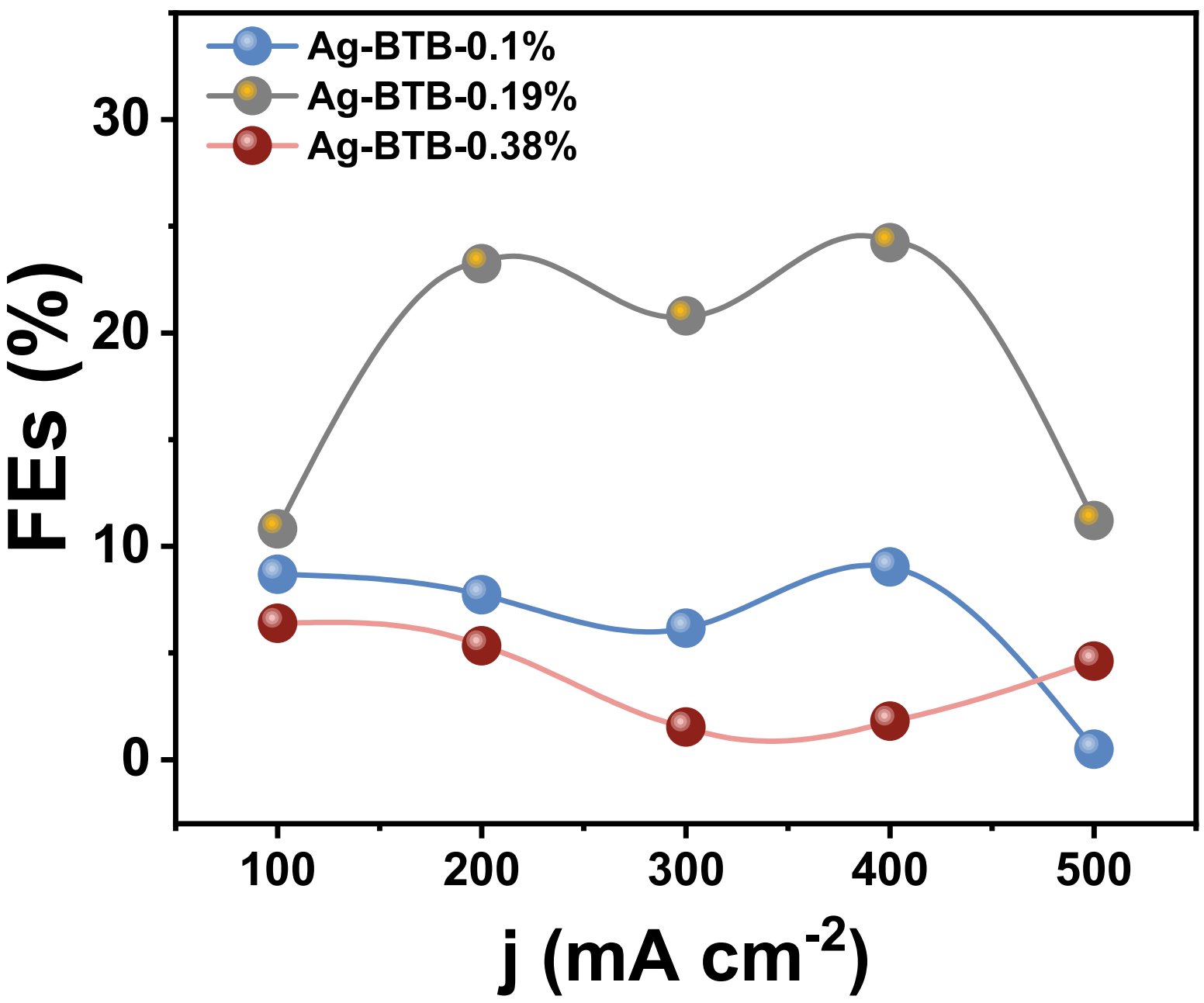

Figure S16. Comparison of Faradaic efficiencies for Ag modified with different contents of BTB.

(a)

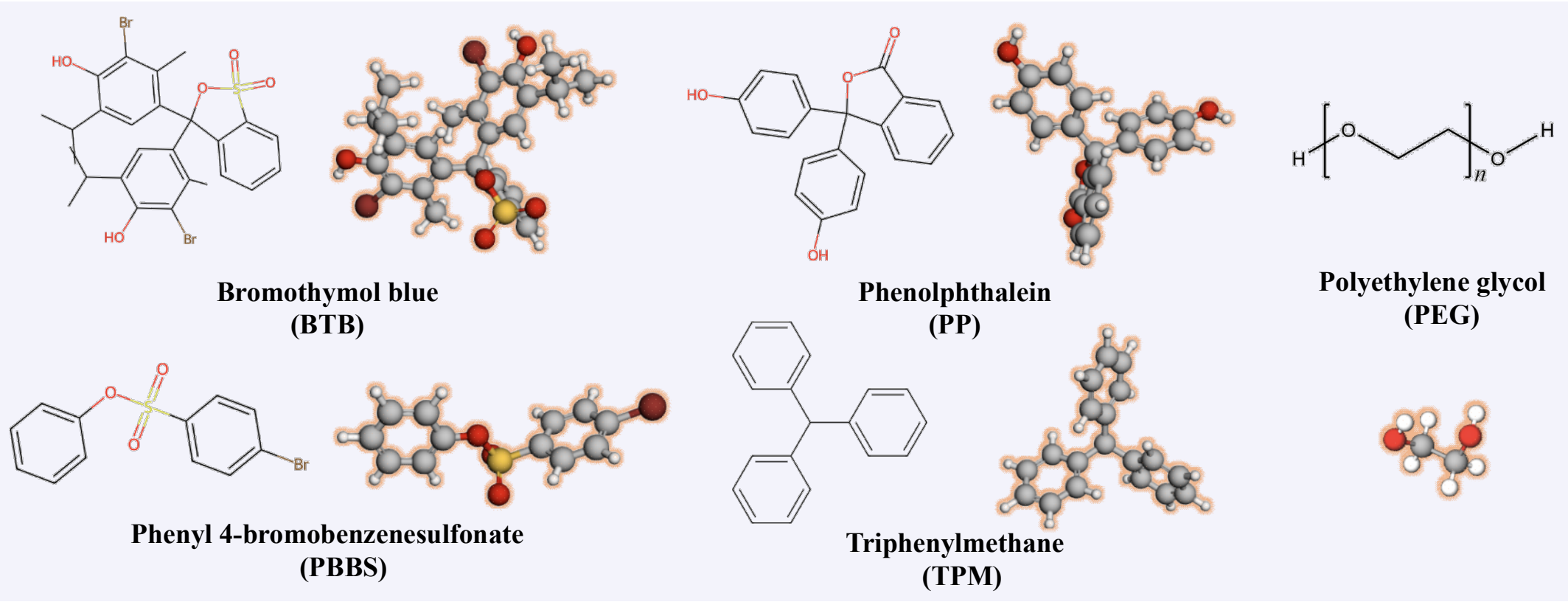

(b)

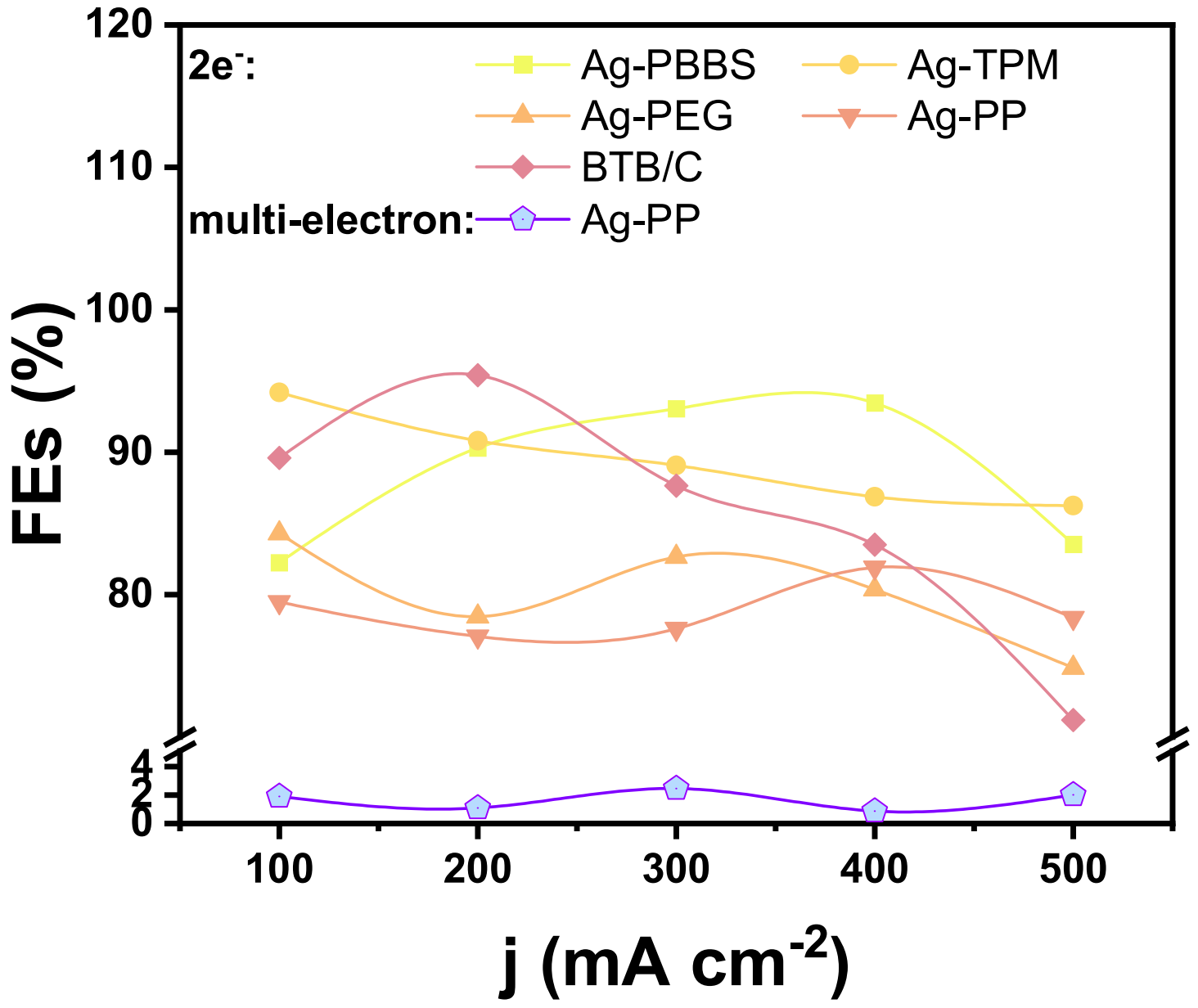

Figure S17. (a) Schematic illustration of different molecular structures. (b) Faradaic efficiencies of the corresponding molecules at current densities ranging from 100 to 500 mA cm^{-2} in a flow cell.

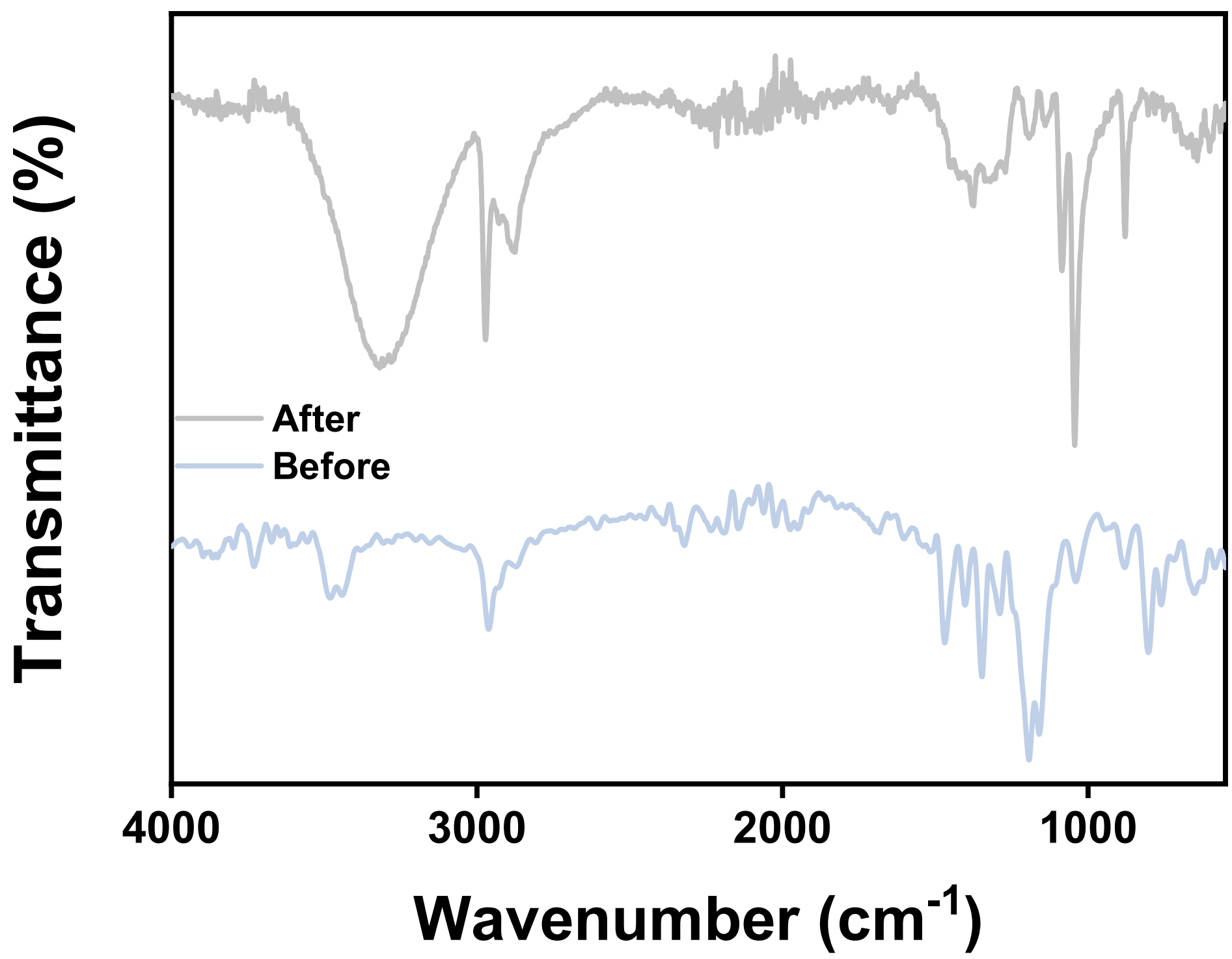

Figure S18. Post-stability FTIR characterization of Ag-BTB.

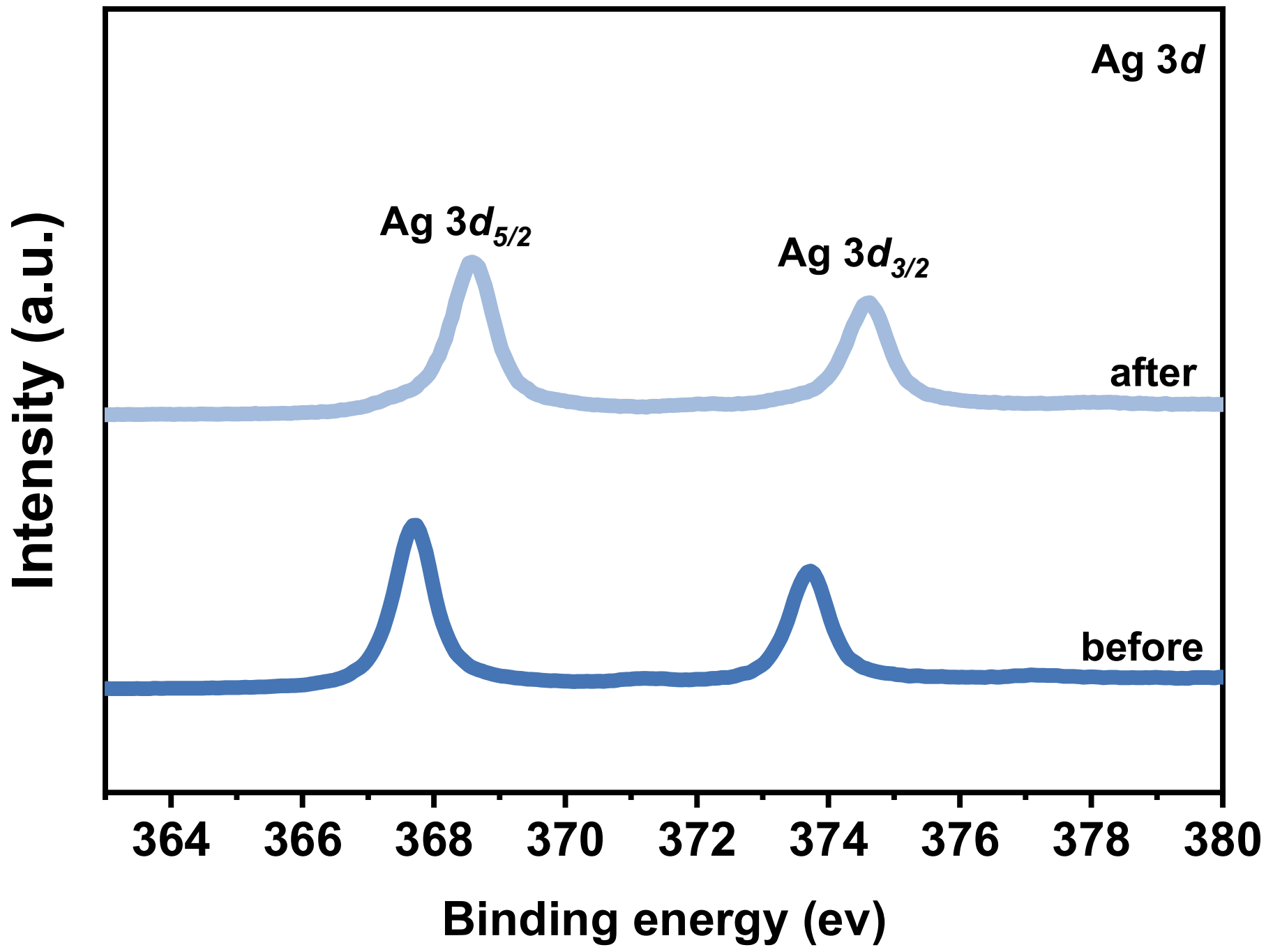

Figure S19. Post-stability XPS characterization of Ag 3*d* for Ag-BTB.

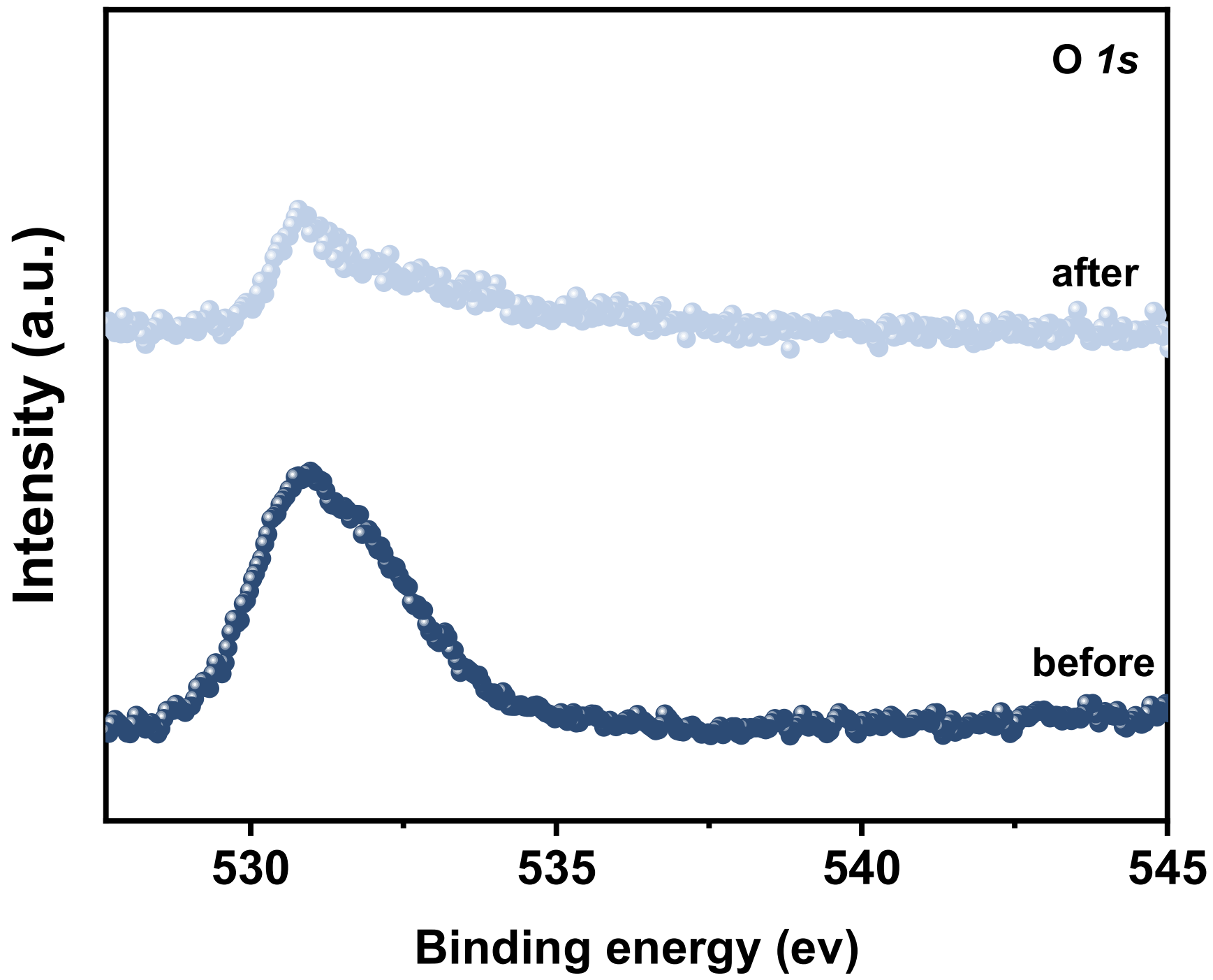

Figure S20. Post-stability XPS characterization of O 1*s* for Ag-BTB.

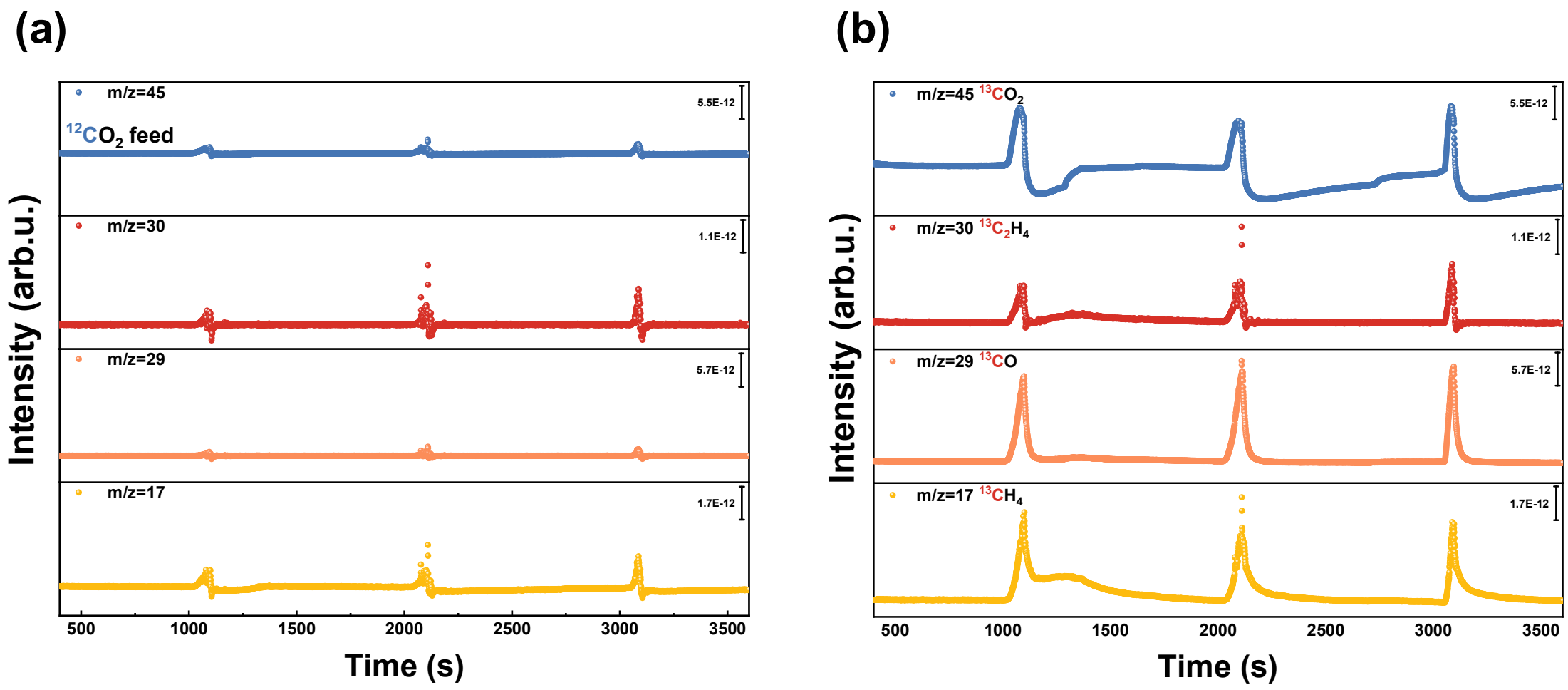

Figure S21. (a) In situ DEMS measurements using $^{12}CO_2$ as the feed gas. (b) In situ DEMS measurements using isotope-labeled $^{13}CO_2$ as the feed gas.

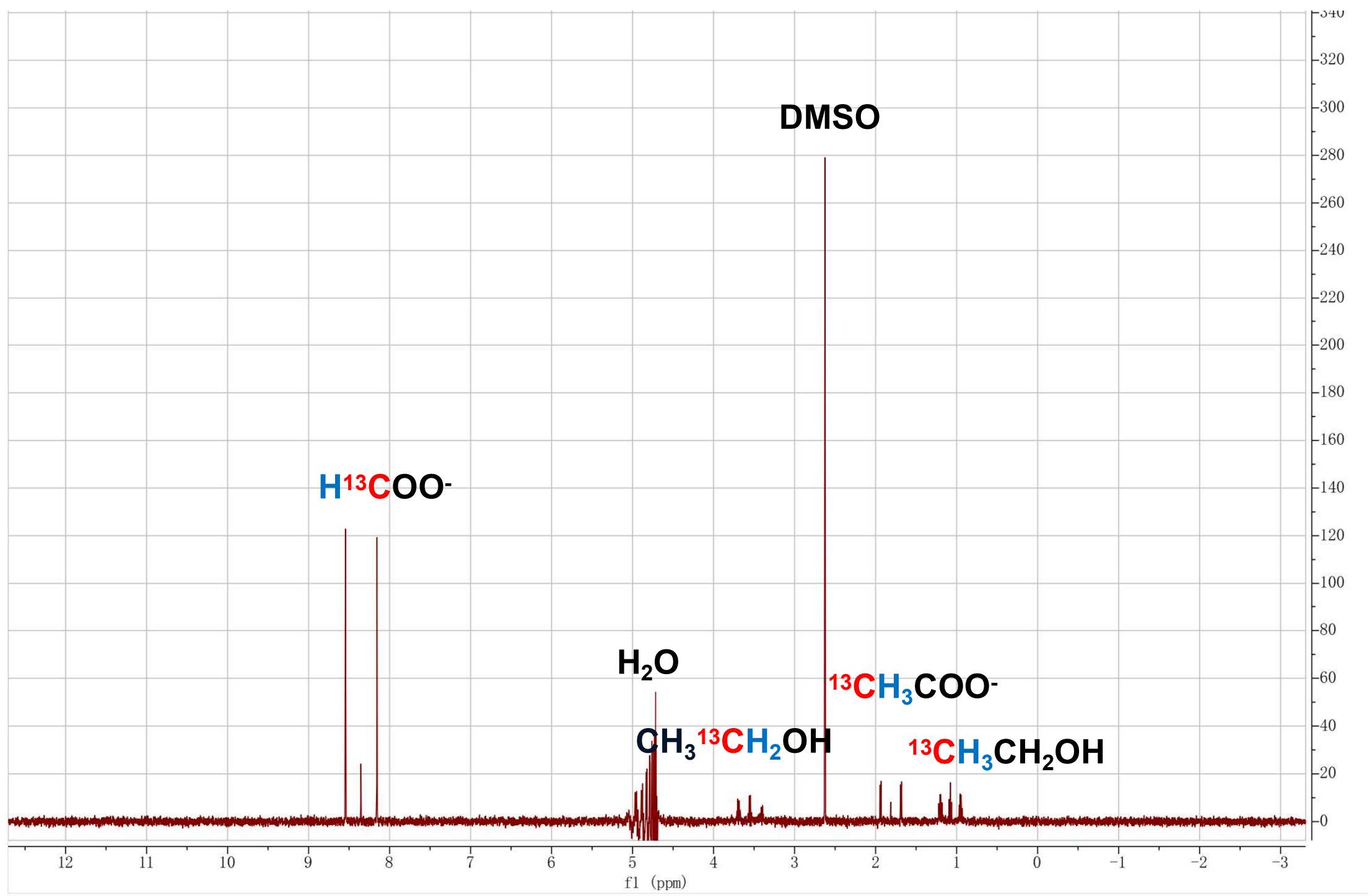

Figure S22. [1]H Nuclear Magnetic Resonance (NMR) spectrum of the liquid electrolyte after $^{13}CO_2$ electroreduction.

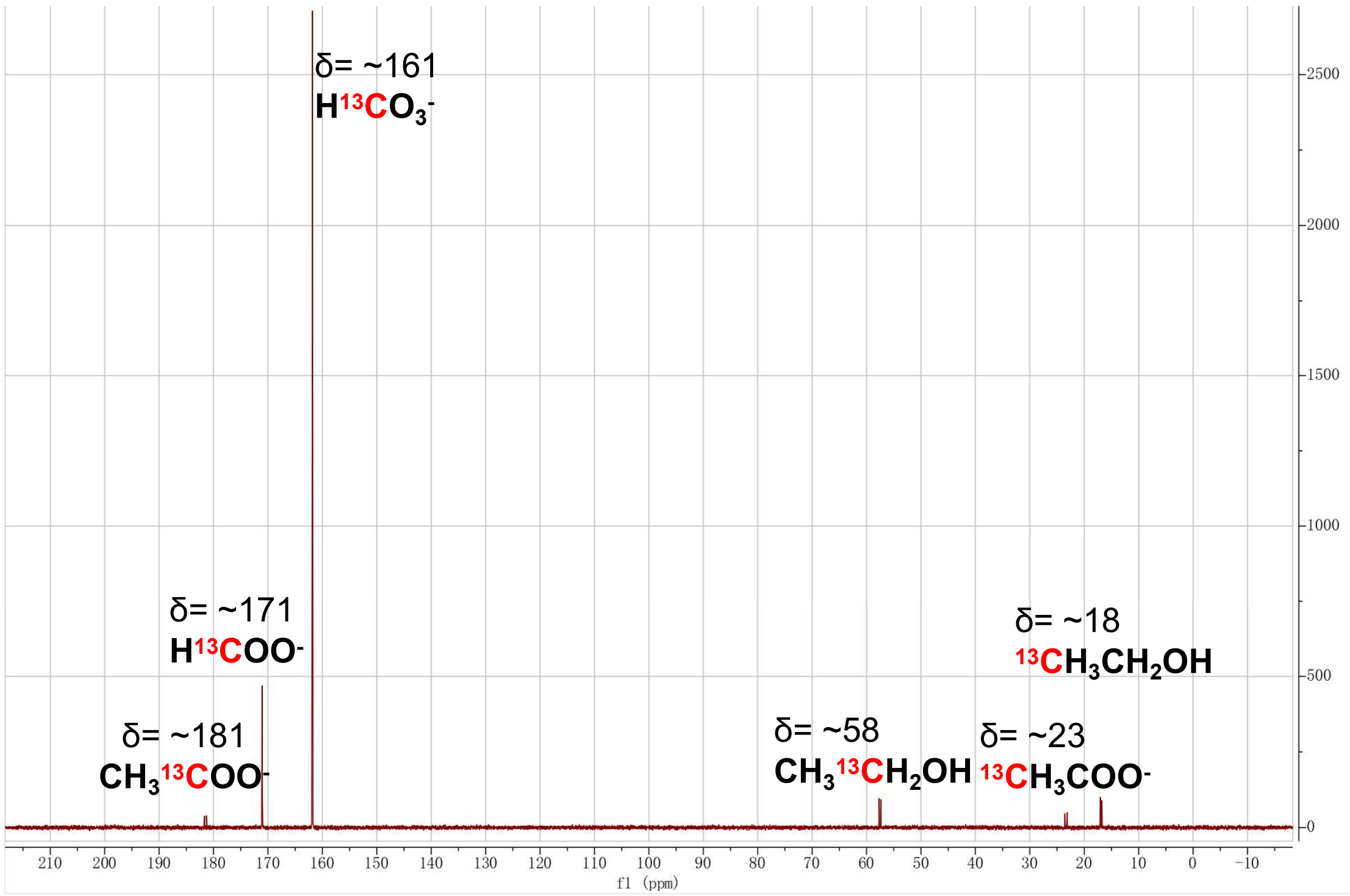

Figure S23. ^{13}C Nuclear Magnetic Resonance (NMR) spectrum of the liquid electrolyte after $^{13}CO_2$ electroreduction.

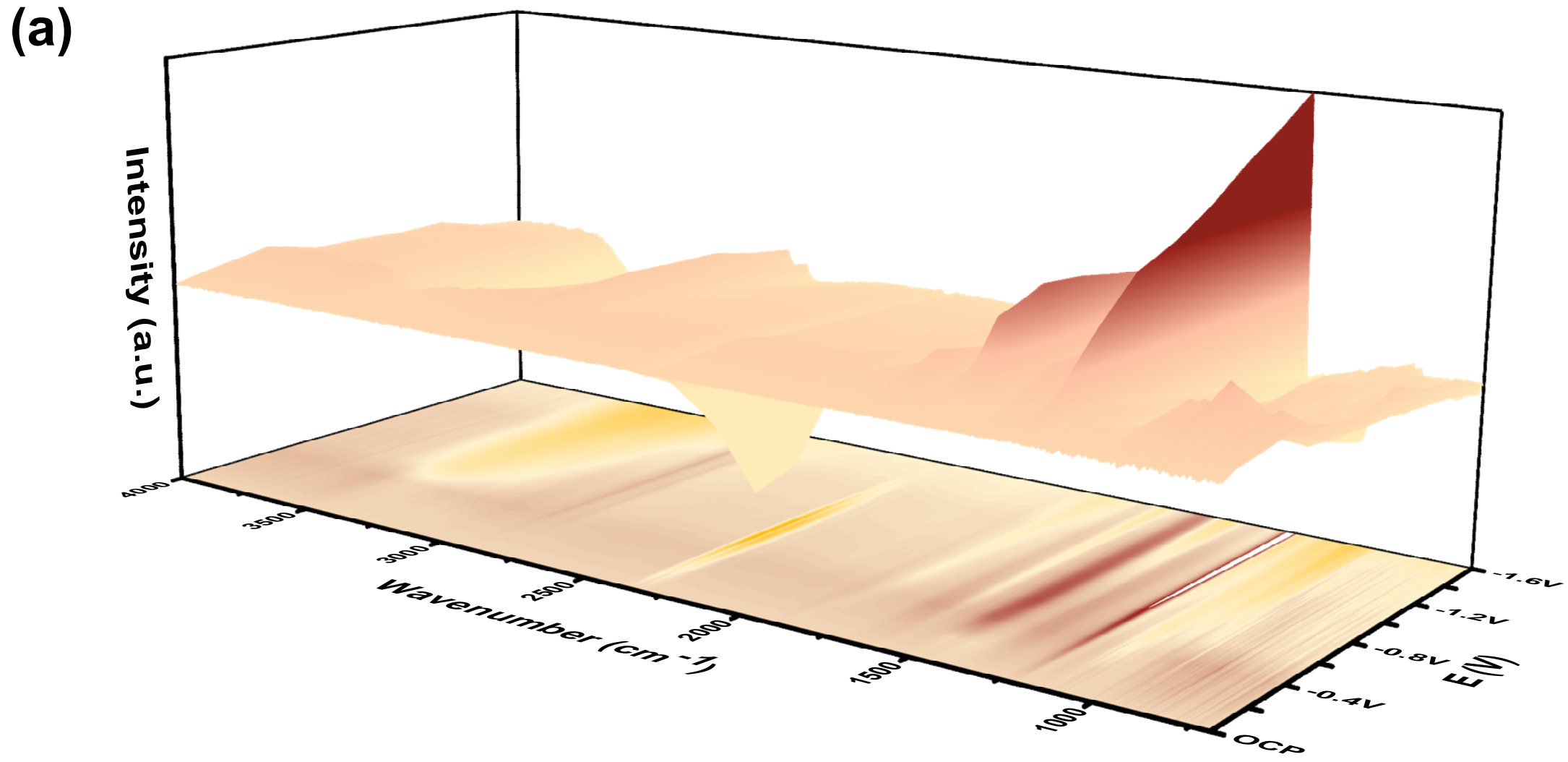

Figure S24. Three dimensional in situ FT-IR spectra of Ag-BTB.

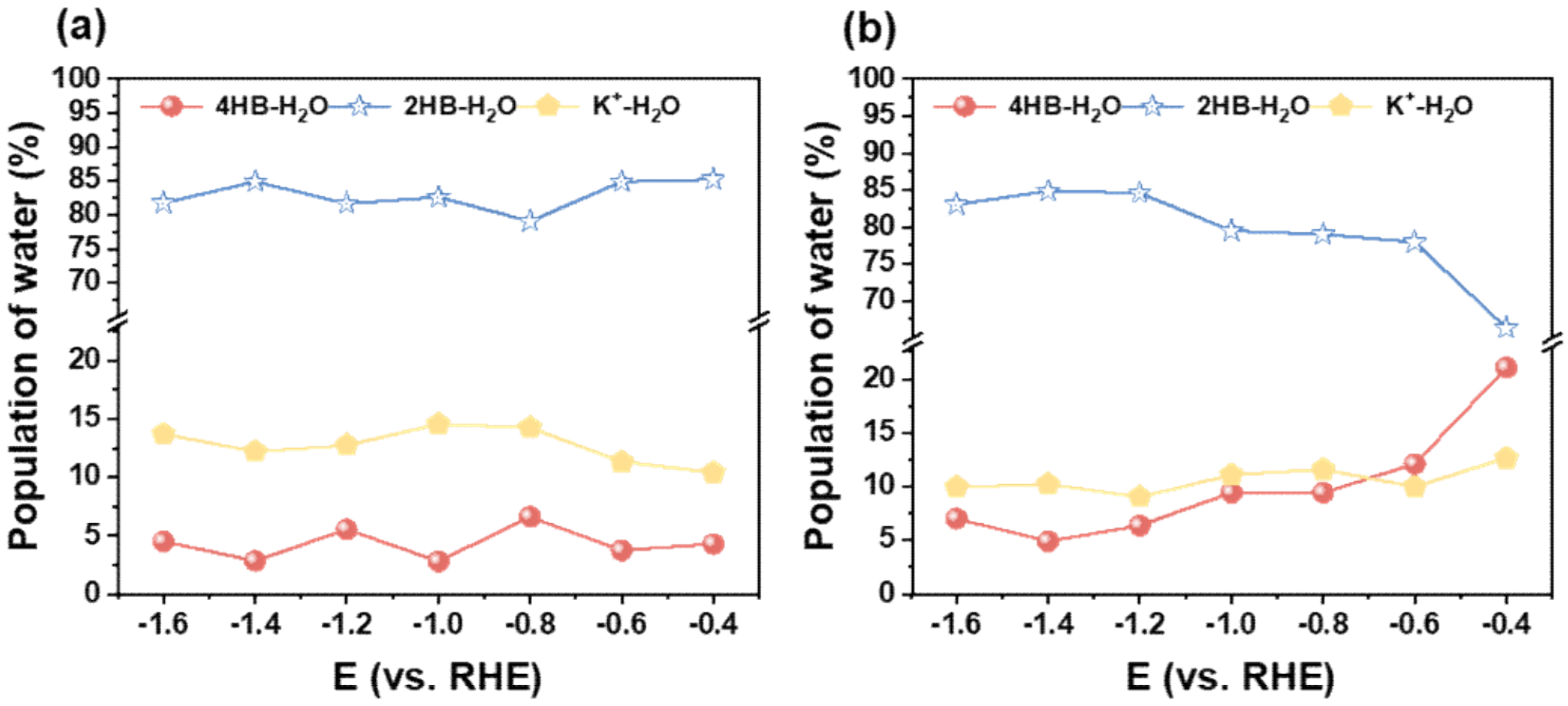

Figure S25. Population of water on Ag (a) and Ag-BTB (b) derived from in situ Raman peak fitting.

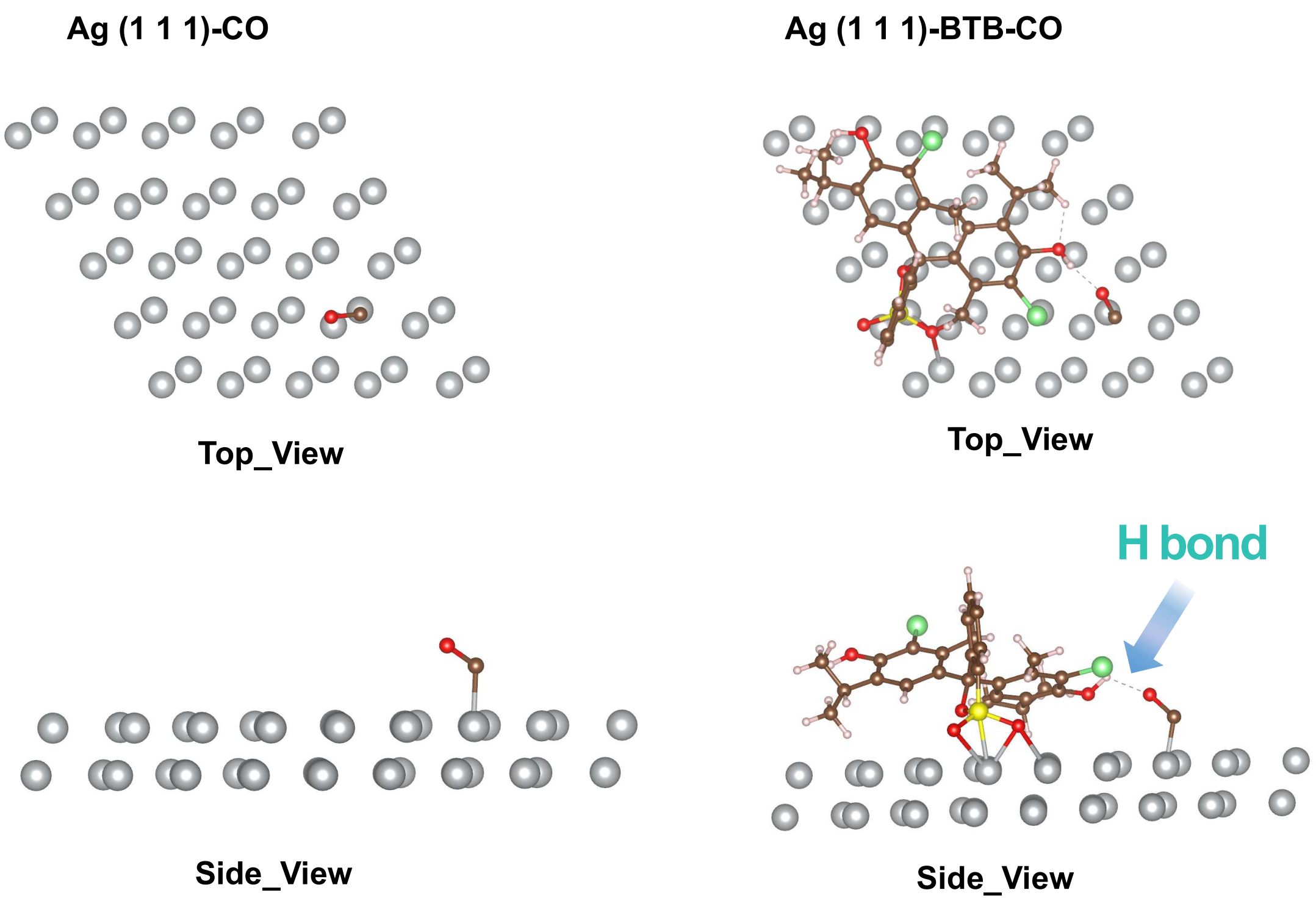

Figure S26. Optimized structure of *CO adsorption configurations on Ag (111) and Ag (111)-BTB from side view and top view. Light gray, brown, red, yellow, green, and light pink balls correspond to Ag, C, O, S, Br, and H atoms, respectively.

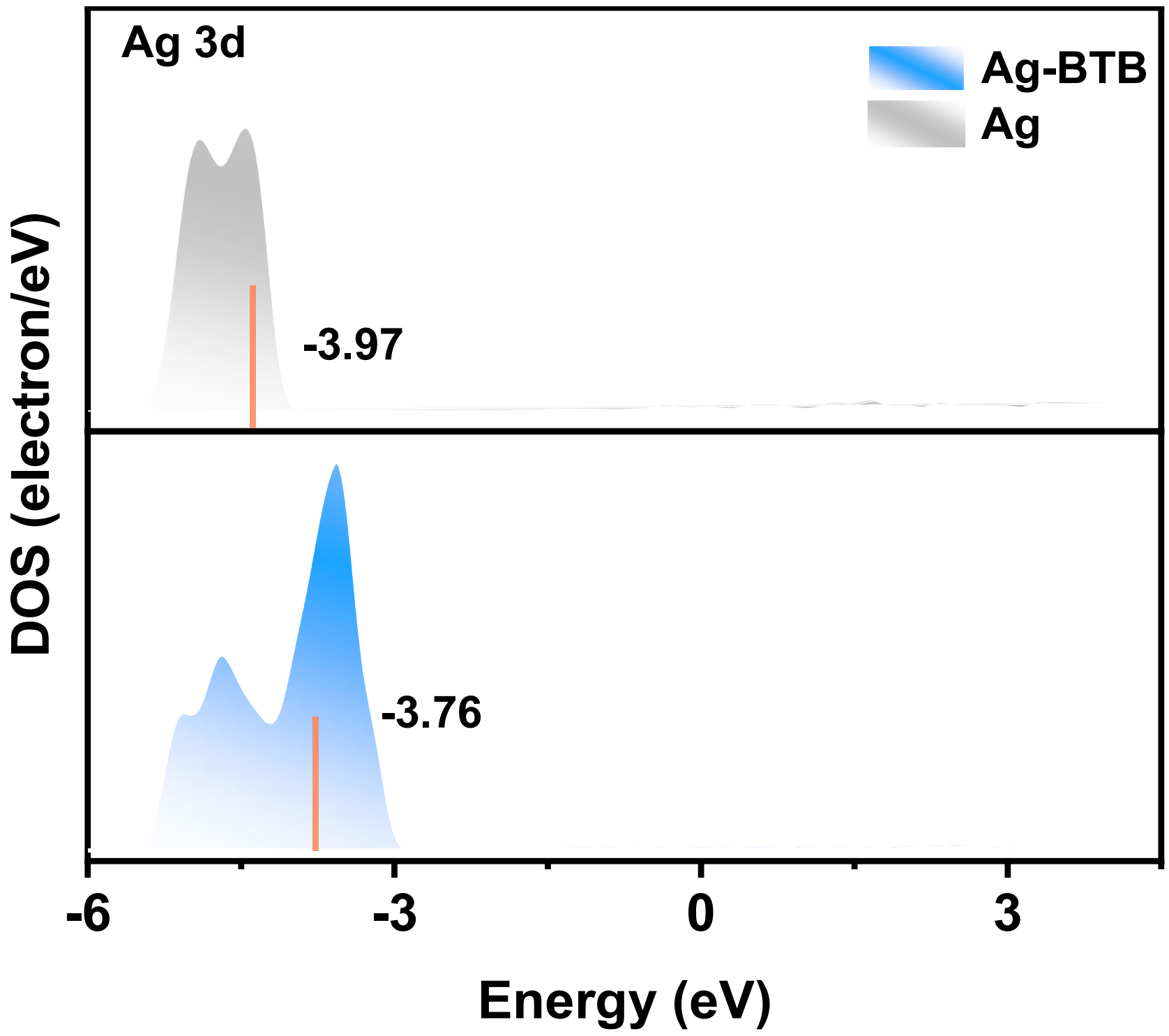

Figure S27. Density of states plots of the Ag 3d orbital of *CO adsorbed on Ag (111) and Ag (111)-BTB.

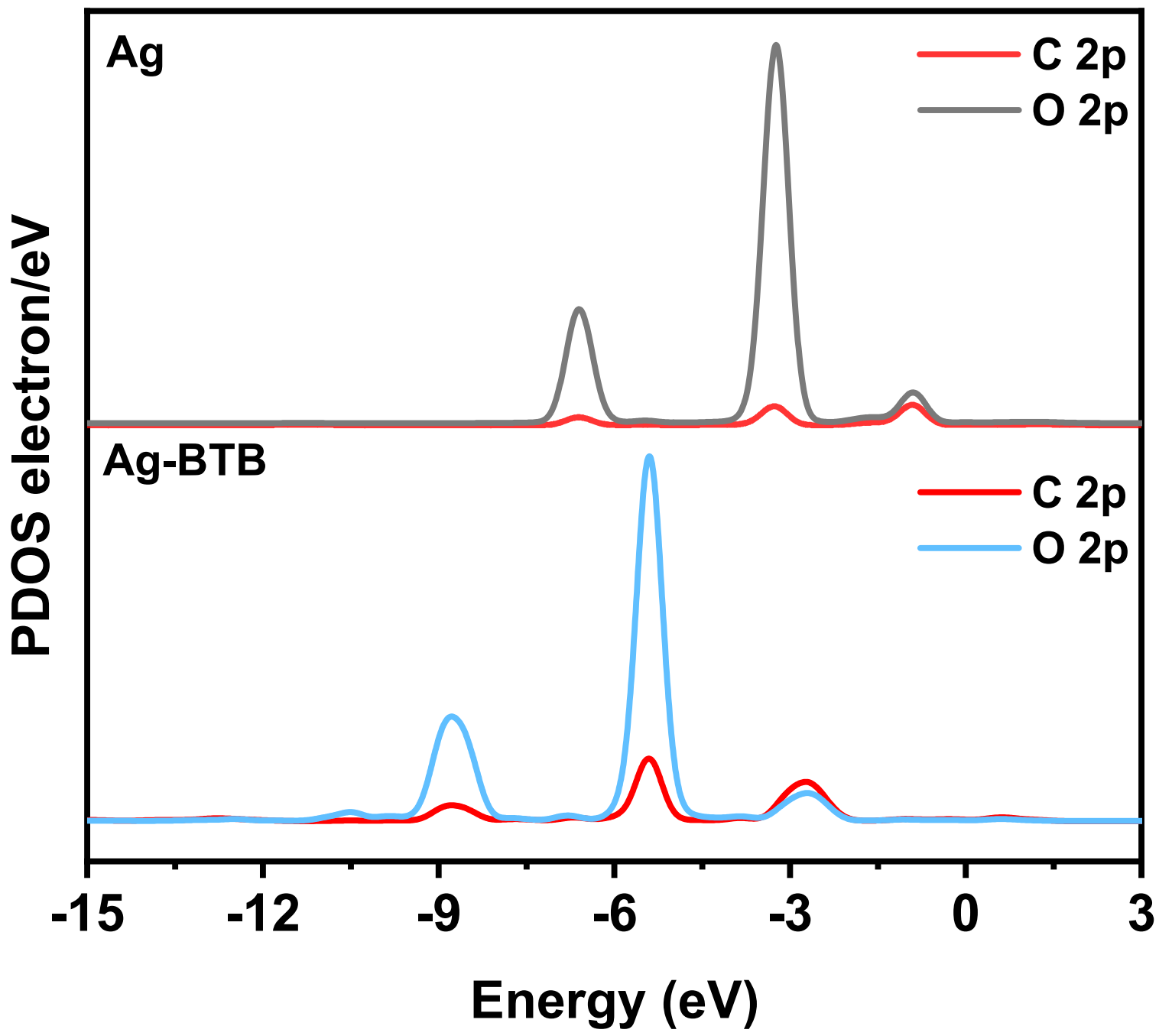

Figure S28. PDOS plots of the O 2p orbital and C 2p orbital of *CO adsorbed on Ag (111) and Ag (111)-BTB

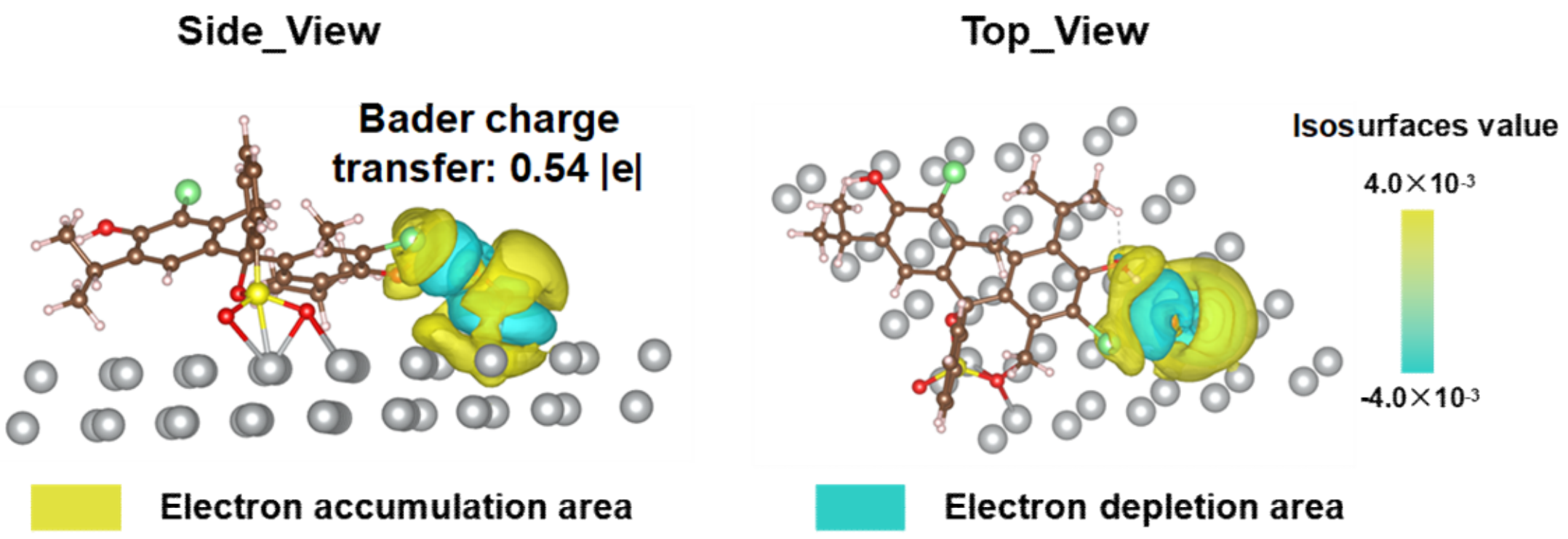

Figure S29. Charge density difference maps and Bader charge analysis for *CO adsorbed on Ag-BTB surfaces. Light gray, brown, red, yellow, green, and light pink balls correspond to Ag, C, O, S, Br, and H atoms, respectively.

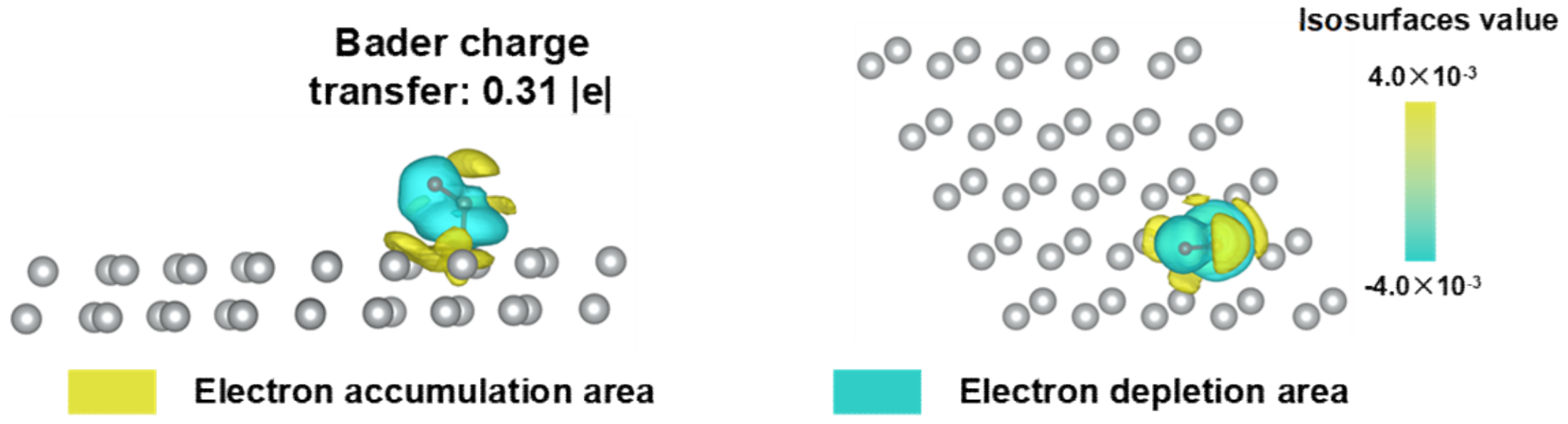

Figure S30. Charge density difference maps and Bader charge analysis for *CO adsorbed on pristine Ag surfaces. Light gray, brown, and red balls correspond to Ag, C, and O atoms, respectively.

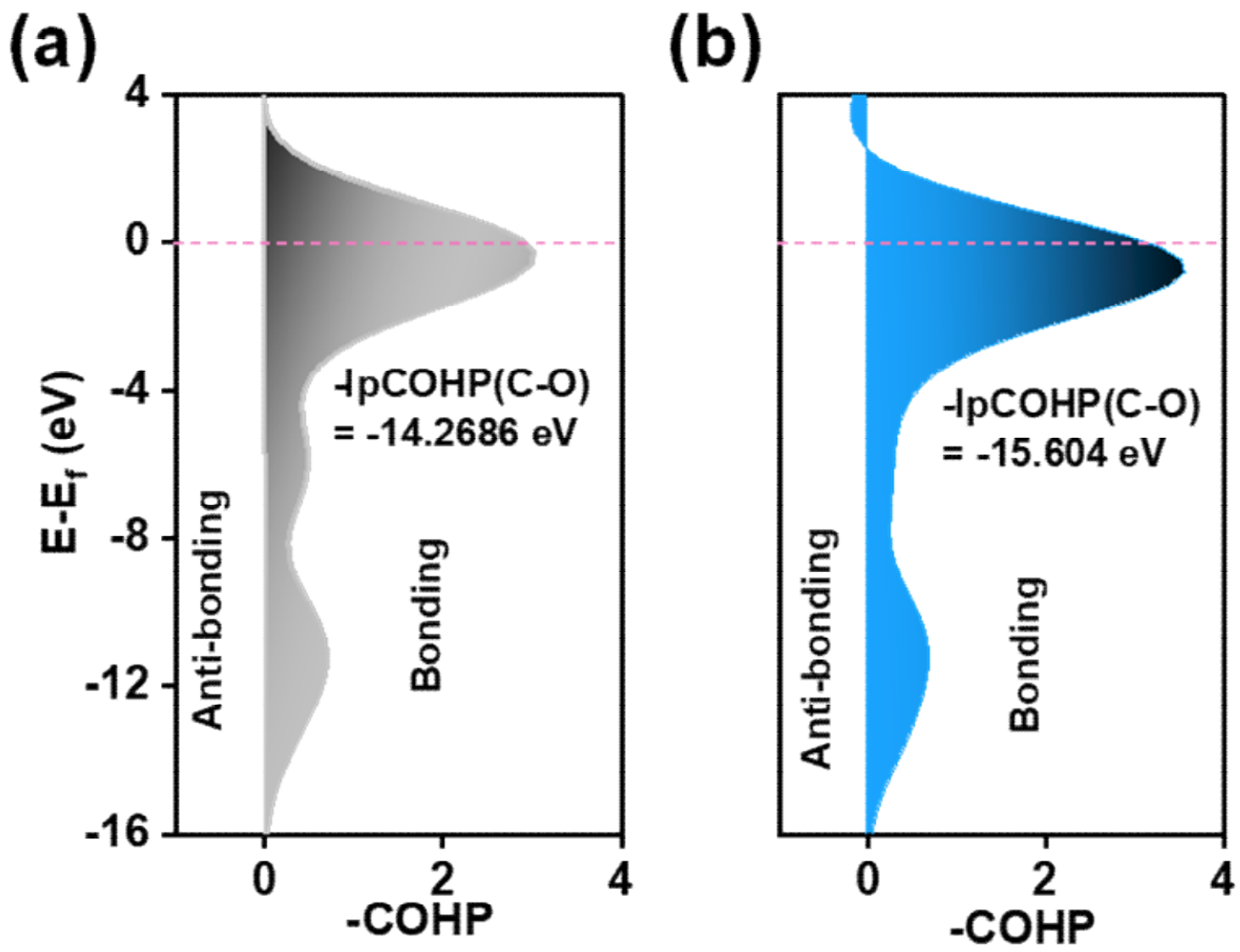

Figure S31. COHP curves for the C-O bonds in *CO on electrode surfaces.

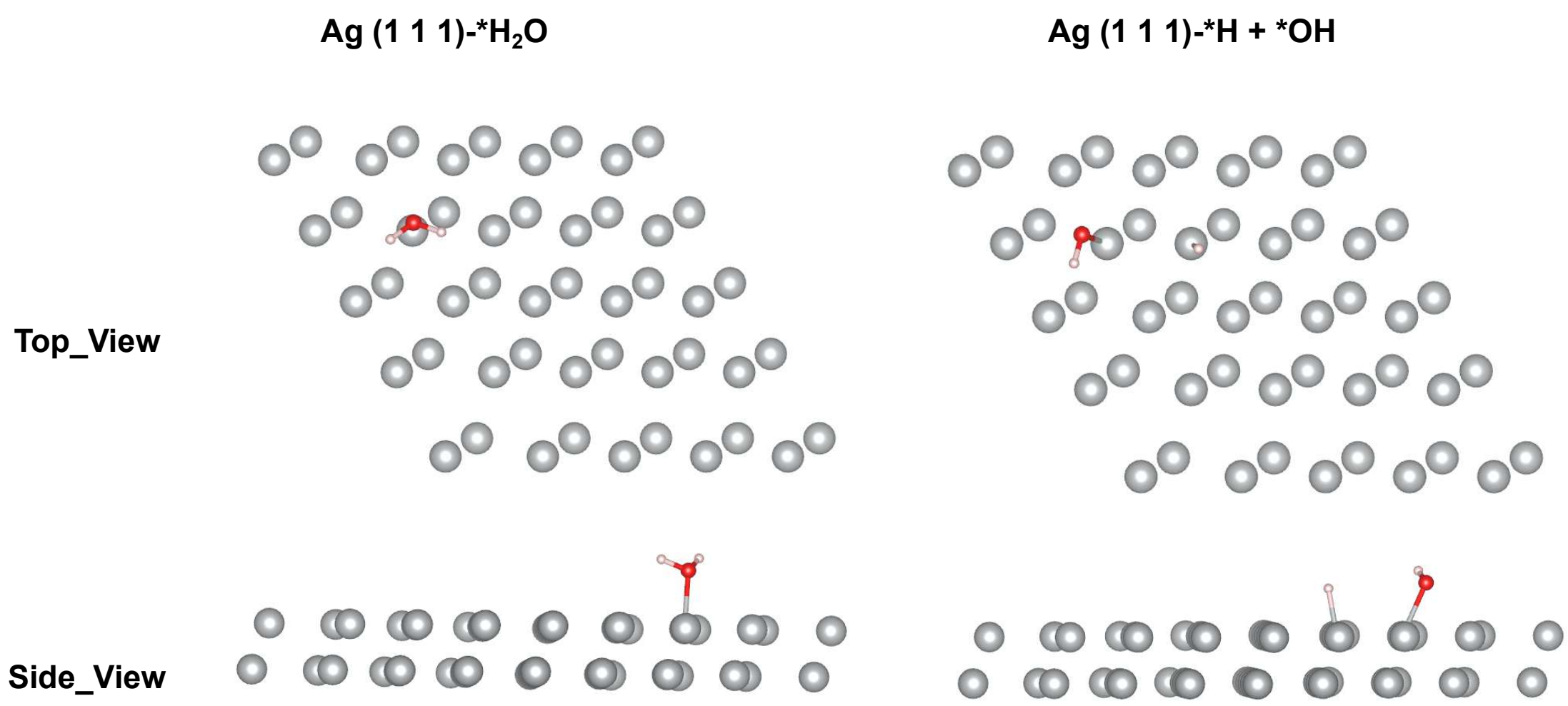

Figure S32. Optimized structure of *H_2O and *H-*OH adsorption configurations on Ag (111) from side view and top view. Light gray, red, and light pink balls correspond to Ag, O, and H atoms, respectively.

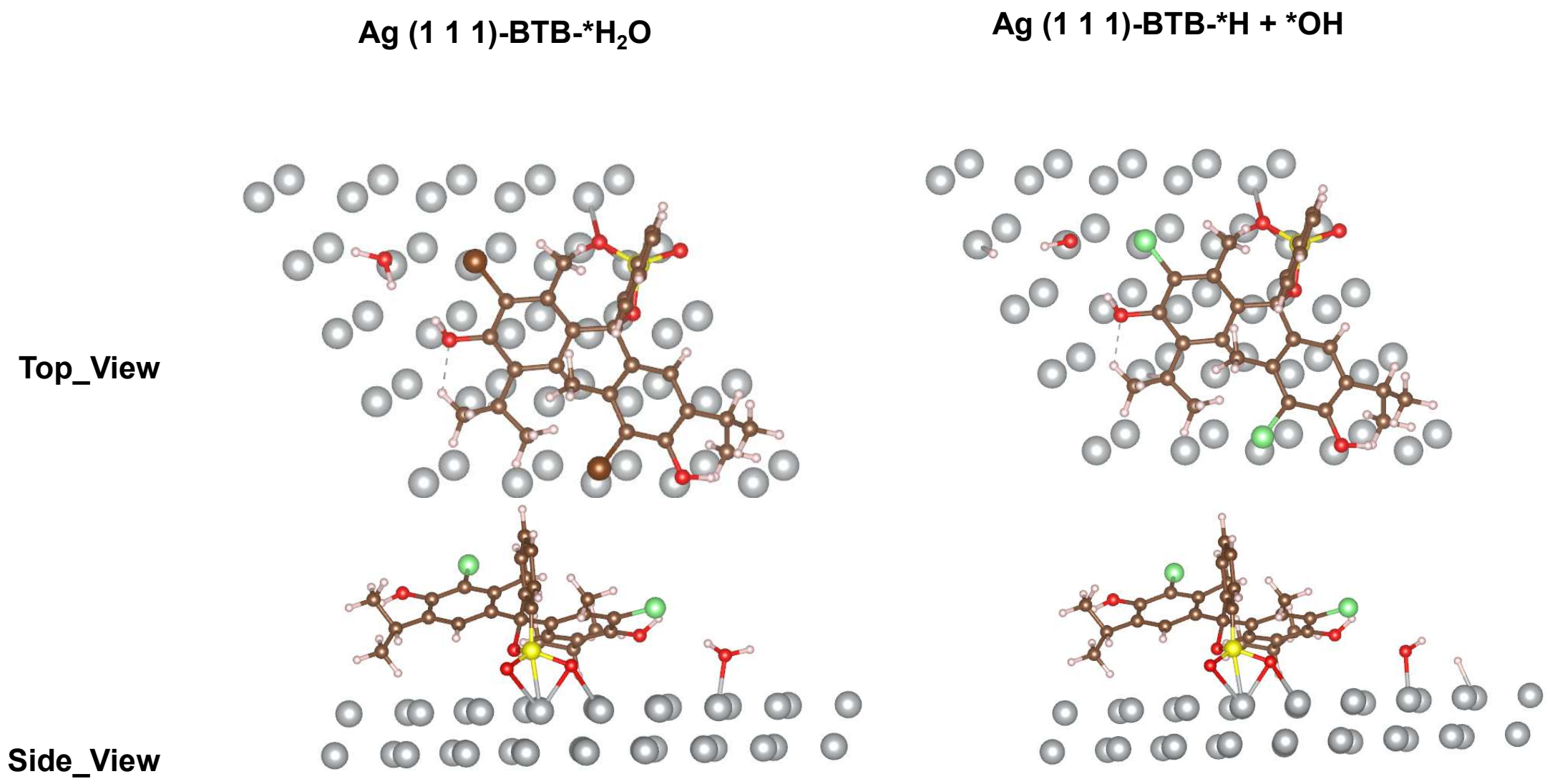

Figure S33. Optimized structure of $*H_2O$ and *H-*OH adsorption configurations on Ag (111)-BTB from side view and top view. Light gray, brown, red, yellow, green, and light pink balls correspond to Ag, C, O, S, Br, and H atoms, respectively.

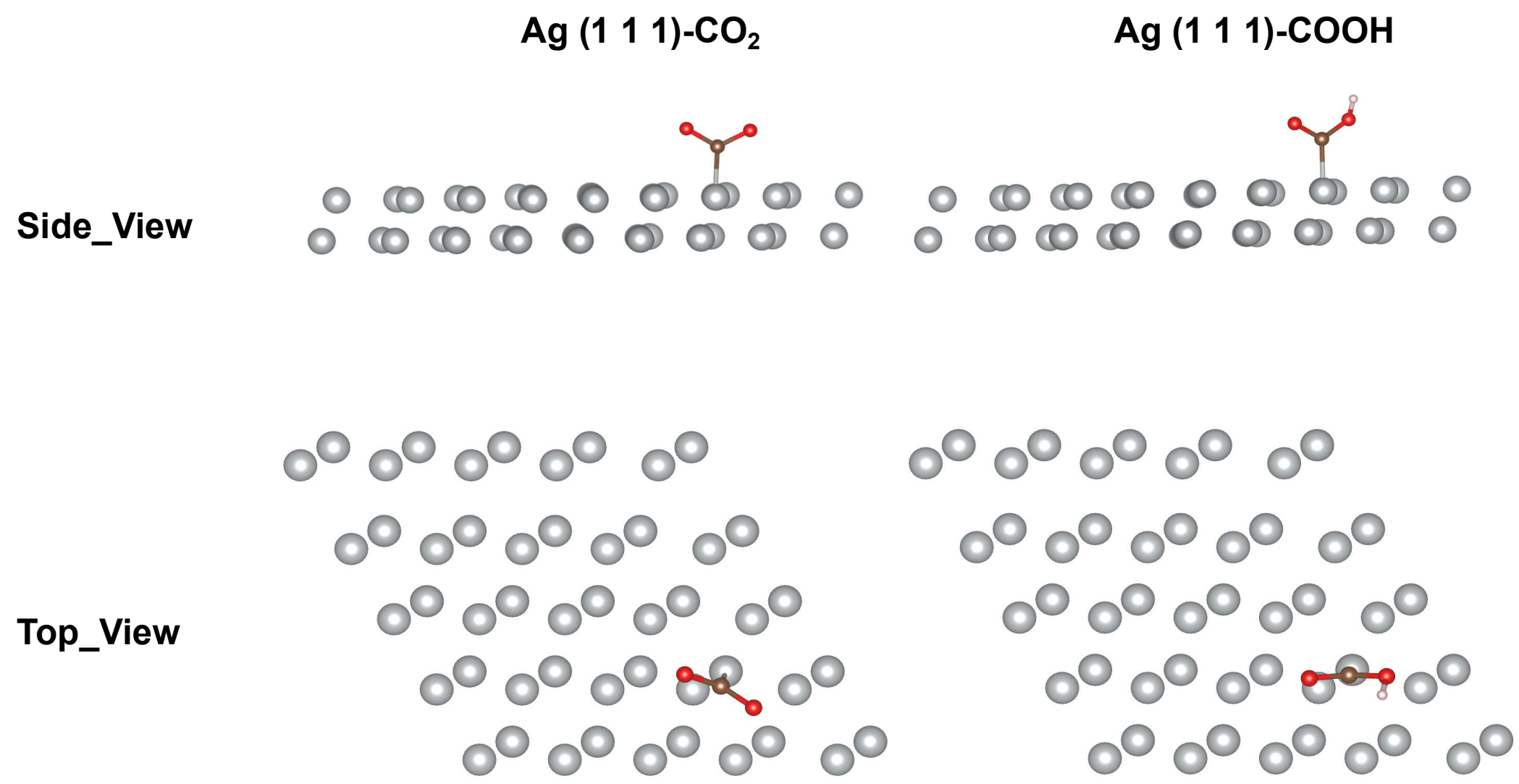

Figure S34. Optimized structure of *CO_2 and *COOH adsorption configurations on Ag (111) from side view and top view. Light gray, brown, red, and light pink balls correspond to Ag, C, O, and H atoms, respectively.

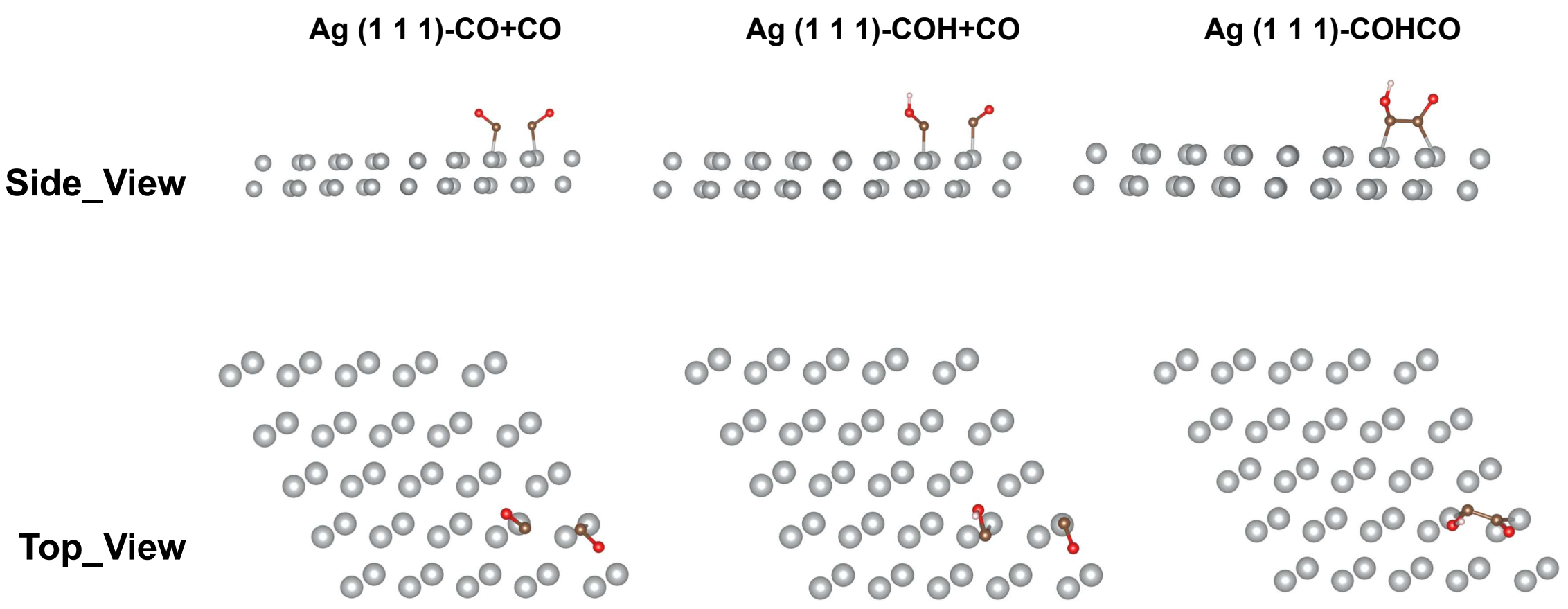

Figure S35. Optimized structure of *CO-*CO, *COH-*CO and *COHCO adsorption configurations on Ag (111) from side view and top view. Light gray, brown, red, and light pink balls correspond to Ag, C, O, and H atoms, respectively.

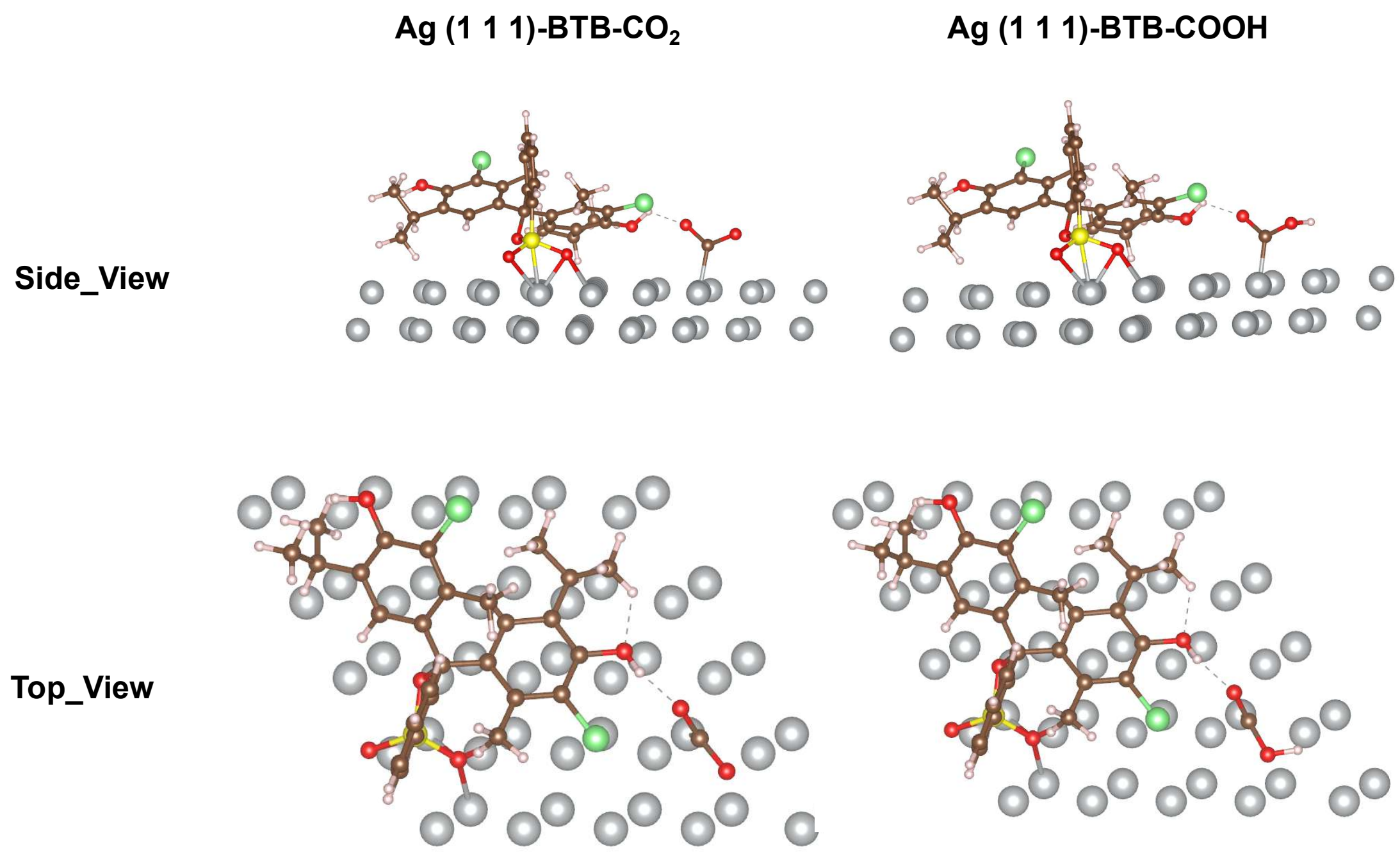

Figure S36. Optimized structure of $*CO_2$ and $*COOH$ adsorption configurations on Ag (111)-BTB from side view and top view. Light gray, brown, red, yellow, green, and light pink balls correspond to Ag, C, O, S, Br, and H atoms, respectively.

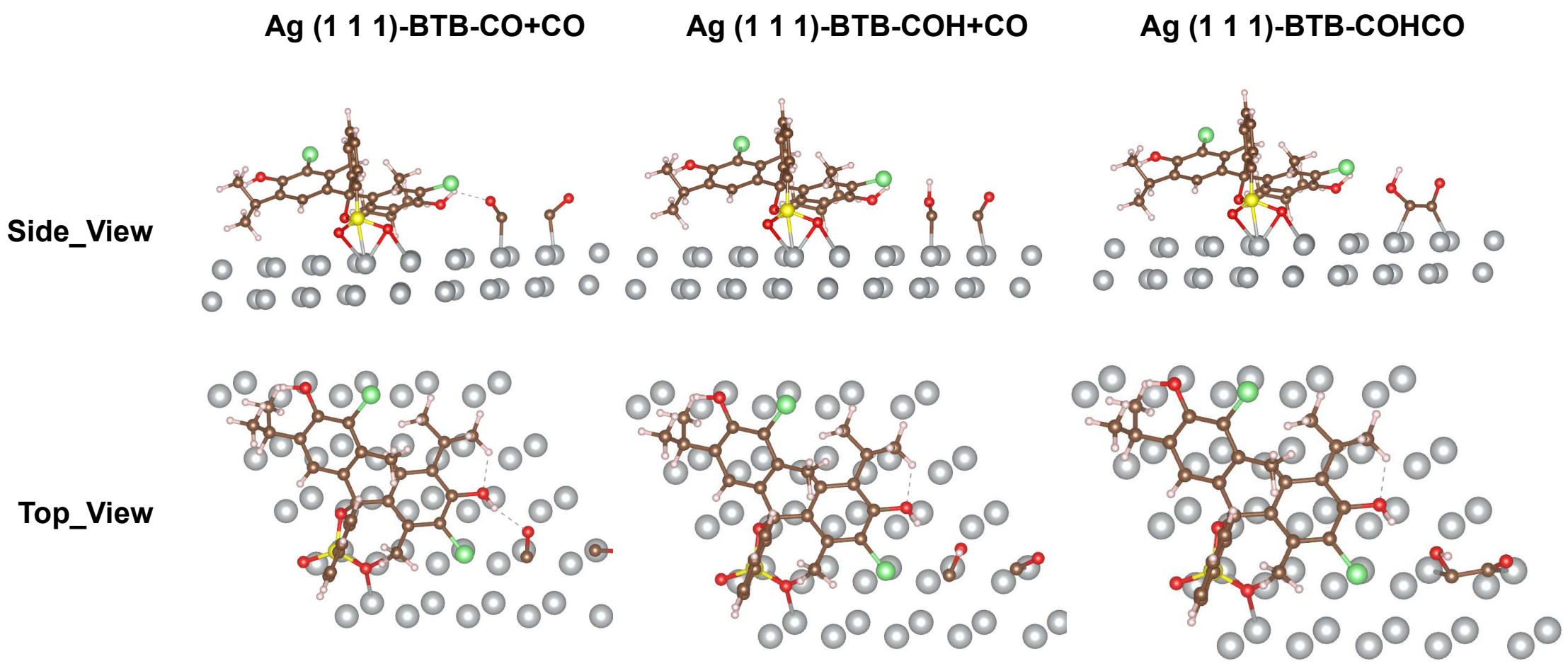

Figure S37. Optimized structure of *CO-*CO, *COH-*CO and *COCHO adsorption configurations on Ag (111)-BTB from side view and top view. Light gray, brown, red, yellow, green, and light pink balls correspond to Ag, C, O, S, Br, and H atoms, respectively.

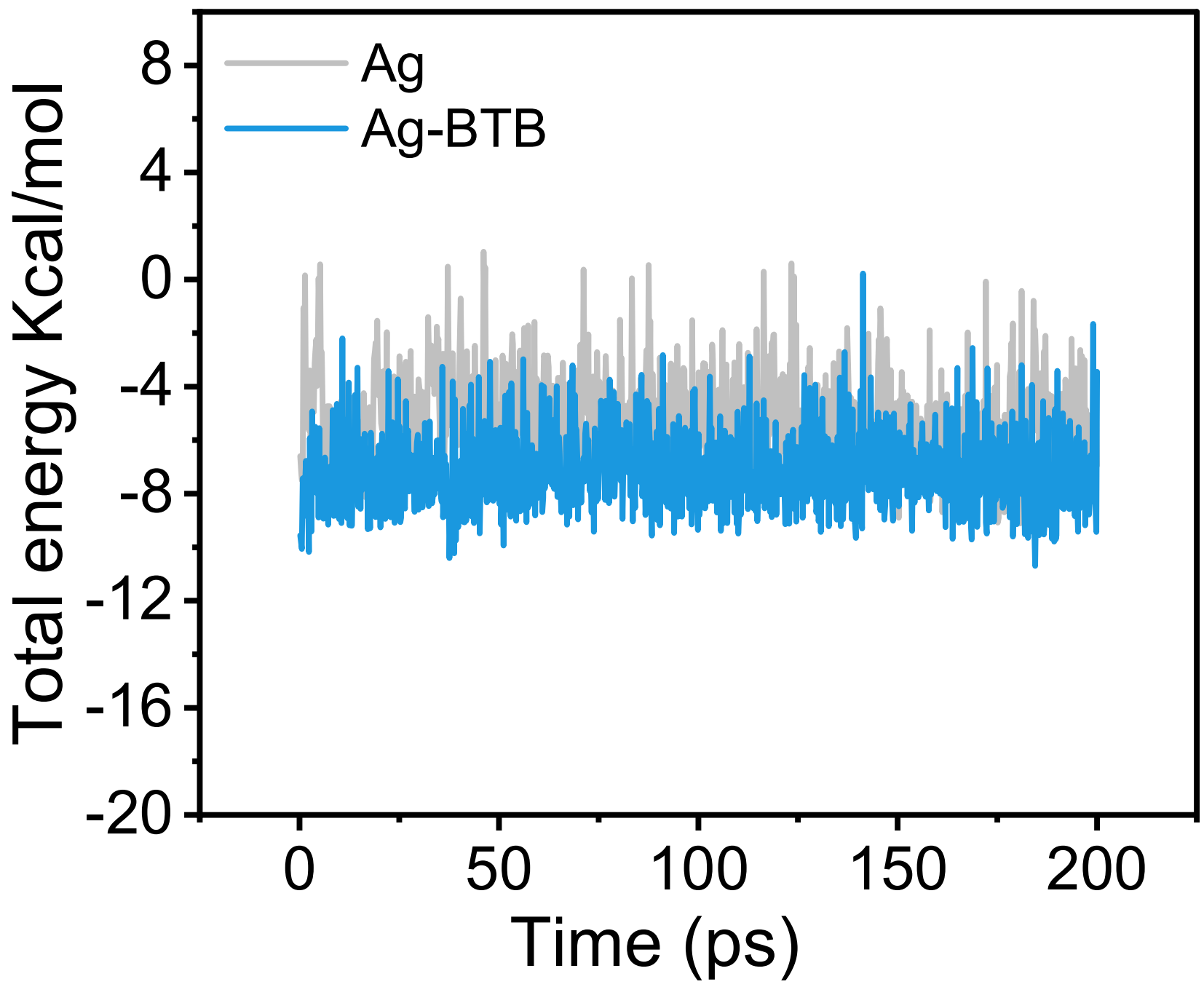

Figure S38. Total energy potential for Ag-*CO and Ag-BTB-*CO system.

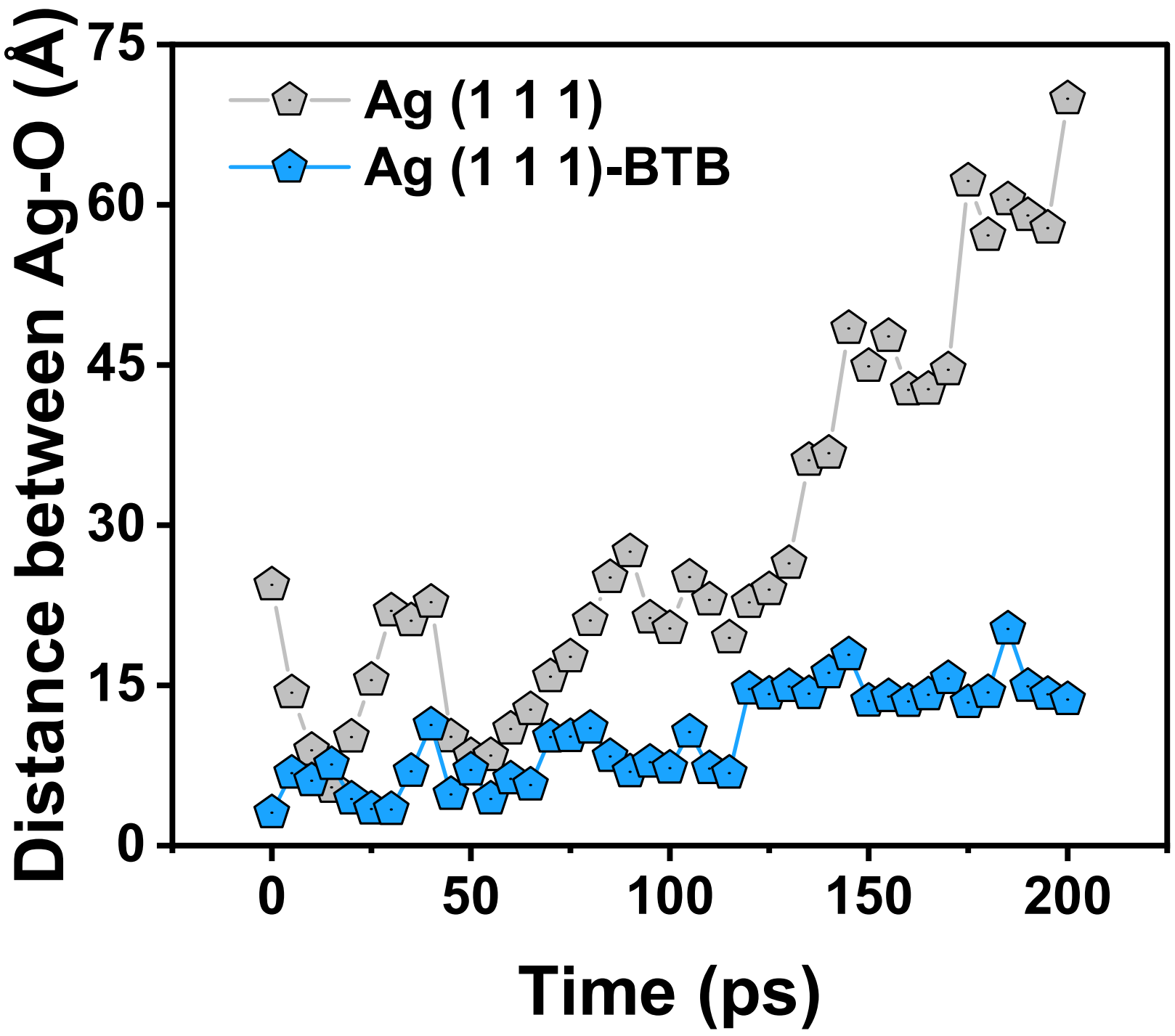

Figure S39. Distance of the O atom in *CO from the Ag surface along the MD trajectory.

Sample	bond type	CN*	R (Å)	σ^2 (10^{-3}Å^2)**	R factor
	Ag-C	0.5±0.1	2.18±0.01	9.6±1.3	
Ag-BTB	Ag-O	2.2±0.2	2.30±0.02	8.8±2.1	0.021
	Ag-Ag	10.6±0.1	2.87±0.01	4.5±3.2	
Ag foil	Ag-Ag	12	2.89±0.02	6.4±3.4	0.007

* CN: coordination number; S_0^2 was fixed to be 0.82 from Ag foil.

** σ^2: Debye−Waller factors

Table S1. EXAFS fitting parameters at the Ag K-edge of Ag-BTB.

References

[1] P. Giannozzi, O. Andreussi, T. Brumme, O. Bunau, M. B. Nardelli, M. Calandra, R. Car, C. Cavazzoni, D. Ceresoli, M. Cococcioni, Advanced capabilities for materials modelling with Quantum ESPRESSO *J. Phys.: Condens. Matter* **29**, 465901 (2017).

[2] P. Giannozzi, S. Baroni, N. Bonini, M. Calandra, R. Car, C. Cavazzoni, D. Ceresoli, G. L. Chiarotti, M. Cococcioni, I. Dabo, QUANTUM ESPRESSO: a modular and open-source software project for quantumsimulations of materials *J. Phys.: Condens. Matter* **21**, 395502 (2009).

[3] J. Paier, R. Hirschl, M. Marsman, G. Kresse, The Perdew–Burke–Ernzerhof exchange-correlation functional applied to the G2-1 test set using a plane-wave basis set *J. Chem. Phys.* **122**, 234102 (2005).

[4] J. P. Perdew, K. Burke, M. Ernzerhof, Generalized gradient approximation made simple *Phys. Rev. Lett.* **77**, 3865–3868 (1996).

[5] S. Grimme, S. Ehrlich, L. Goerigk, Effect of the damping function in dispersion corrected density functional theory *J. Comput. Chem.* **32**, 1456–1465 (2011).

[6] S. Grimme, J. Antony, S. Ehrlich, H. Krieg, A consistent and accurate ab initio parametrization of density functional dispersion correction (DFT-D) for the 94 elements H-Pu *J. Chem. Phys.* **132**, 154104 (2010).
