# Supplementary material for: Hydrogen bond stabilized *CO intermediate enables CO2 electroreduction to multi-electron products on silver catalysts
Source: Nat Commun. 2026 Jun 25;17:8700. doi: 10.1038/s41467-026-73970-9 (PMC13490397; doi:10.1038/s41467-026-73970-9)
Supplement: Supplementary file 2 [file 41467_2026_73970_MOESM2_ESM.pdf]

Description of Additional Supplementary Files

File Name: Supplementary Data 1

Description: This is a Zip file containing the atomic coordinates of DFT and MD structures for Ag (111) and Ag (111)-BTB.
