# Supplementary material for: Hydrogen bond stabilized *CO intermediate enables CO2 electroreduction to multi-electron products on silver catalysts
Source: Nat Commun. 2026 Jun 25;17:8700. doi: 10.1038/s41467-026-73970-9 (PMC13490397; doi:10.1038/s41467-026-73970-9)
Supplement: Supplementary file 3 [file 41467_2026_73970_MOESM4_ESM.pdf]

nature portfolio

Peer Review File

Hydrogen Bond Stabilized *CO Intermediate Enables CO2 Electroreduction to Multi-Electron Products on Silver Catalysts

Corresponding Author: Professor Chuanxin He

This file contains all reviewer reports in order by version, followed by all author rebuttals in order by version.

Version 0:

Reviewer comments:

Reviewer #1

(Remarks to the Author)
Zhang et al. develops a molecular modification strategy to enhance CO2 electroreduction on silver catalysts. By anchoring bromothymol blue (BTB) on Ag surfaces, a hydrogen-bonding network stabilizes the *CO intermediate, prolonging its residence time and enabling further hydrogenation and C C coupling. This shifts product selectivity from primarily CO to multi-electron products like CH4 and C2H4, achieving a 24.2% Faradaic efficiency at 400 mA cm 2. The work highlights a paradigm shift from metal-centric tuning to local microenvironment engineering, offering a general approach to activate non Cu catalysts for deep CO2 reduction. This manuscript can be accepted after solving the below comments:
1. The authors propose a novel mechanism to stabilize CO molecule via the interaction of H-bond. However, the stability analysis is mainly through the results of C-O bond strength, such as in Figure 5b and Figure S25, indicating enhanced C-O interactions. Mostly, the activation of C-O bond is beneficial for the following hydrogenation of CO to multi-electron species. How about the Ag-C bond strength? You should further evaluate the Ag-C interaction via COHP or other electronic structure methods.
2. In order to quantity the interfacial interaction of CO and BTB, you can also add the Bader charge analysis, combining with the differential charge density result, compared with the pristine Ag surface.
3. To clarify the stability of CO in the Ag (111)-BTB via MD results, you can count the height of C and O atom of CO on the Ag(111) surface in the entire trajectory of 200 ps. Additionally, the potential energy statistics results can not illustrate the stability because the CO-Ag (111)-BTB system owns more atoms than CO-Ag (111). Please carefully think over it.
4. In the computational details, do you consider the effect of solvation and Van der Waals correlation on the adsorption of reactant and intermediates, specifically for involving of large molecule BTB. Additionally, the computational methods were very simple, please supplement it, such as, the computational hydrogen electrode method, adsorption energy formula and MD details.
5. In recent years, there are a series of latest work about multi-carbon products of CO2RR in the Cu-based and non-Cu based electrocatalysts that you can refer. Such as, The Journal of Physical Chemistry Letters, 2025, 16 (42), 11016-11024; Nature Communications, 2025, 16 (1), 2217; Nature Communications, 2025, 16 (1), 1203.
6. All the atoms in the diagram of atomic models should added the atomic representatives in order to distinguish the identity of atom in the Figures 6 and S22, S23, S26-S30.
7. For the differential charge density, the isosurface values of the contour should be denoted in Figure S23.

Reviewer #2

(Remarks to the Author)
The manuscript presents an innovative molecular modification strategy using bromothymol blue (BTB) to enhance CO2 electroreduction on Ag electrodes, addressing the long-standing challenge of weak *CO intermediate adsorption. The reported 24.2% Faradaic efficiency (FE) for multi-electron products at high current density is noteworthy, and the shift from metal-centric to microenvironment engineering is conceptually appealing. The integration of in situ spectroscopy and theoretical simulations strengthens the mechanistic insights. Thus, I recommend accept after major revision.
1. The authors chose to modify the Ag surface with BTB molecules, attributing this to the role of OH. Besides BTB, there are other molecules with OH functional groups. Why was BTB selected? Have other molecules been tried? Please explain the

specific reasons for choosing the BTB molecule.
2. How will loading different amounts of BTB affect the catalytic results? Please provide experimental evidence.
3. One of the advantages of using non-Cu-based catalysts is the catalyst stability. The advantage of the Ag material also lies in its stability. Therefore, the authors should verify the stability of Ag-BTB, especially the stability of BTB during the catalytic reaction. The infrared spectra after the reaction should be provided to explore whether there are any changes in BTB during the reaction. Please provide experimental data to show the changes in BTB during the reaction.
4. In the stability experiment in Fig. 2f, only the change in Faraday efficiency of the total multi - electron products were tracked. However, at least the changes in Faraday efficiency of ethanol and acetic acid should also be given.
5. There is a lack of 13CO2 experiments. (i) Please provide in situ differential electrochemical mass spectrometry during the experimental process, and (ii) conduct high-resolution mass spectrometry analysis of the all labeled products collected after the reaction.
6. Please provide the GC spectra and NMR spectra of the catalytic products.
7. here are numerous possibilities for C-C coupling, commonly including intermediates such as OCCO, OCCHO, and OCCOH. Can in situ infrared spectroscopy detect the aforementioned three intermediates? It is recommended to compare these three intermediates through computational analysis to elucidate their differences in the specific context discussed in this article.
8. What is the peak around 55° in the XRD? What substance does it belong to?
9. In Fig. 1g, is the peak of the Ag-BTB EXAFS at approximately R = 2 Å generated due to the bonding between Ag and BTB?

Reviewer #3

(Remarks to the Author)
This manuscript investigates a molecular modification strategy using Bromothymol Blue (BTB) to facilitate multi-electron CO_2 reduction on silver catalysts. The authors propose that an interfacial hydrogen-bonding network stabilizes the *CO intermediate, enabling the formation of multi-electron products. The reported formation of multi-electron products on silver—a metal traditionally limited to C1 pathways—is a noteworthy observation. However, significant mechanistic and experimental deficiencies undermine the primary conclusions. Specifically, the proposed hydroxyl-mediated mechanism is chemically inconsistent with the strongly alkaline conditions. Therefore, the study does not meet the standards required for Nature Communications. Specific comments are listed below:

1. The authors hypothesize that phenolic hydroxyl (-OH) groups stabilize the *CO intermediate via hydrogen bond. However, at the experimental pH of ≈ 14 (1 M KOH), BTB (pKa ≈ 7.1) exists predominantly in its deprotonated phenolate form (-O-). This deprotonation precludes the molecule's function as a conventional hydrogen bond donor, rendering the proposed mechanism chemically inconsistent with the alkaline operating conditions.

2. The comparative study between BTB and triphenylmethane (TPM) is insufficient to isolate the specific catalytic role of the hydroxyl groups. BTB possesses multiple functional moieties, including bromine atoms and a sulfonic acid group, which may significantly modulate the interfacial electronic properties and local hydrophilicity. The current experimental design fails to decouple these confounding factors. Furthermore, the absence of a broader evaluation involving other hydroxyl-containing molecules (e.g., phenolphthalein, phenol or other triarylmethane derivatives) limits the understanding of the structure-activity relationship and the generalizability of the proposed mechanism.

3. In the in-situ Raman spectroscopy measurements, the application of the water-structure deconvolution model from the Nature 2021 study (Ref. 41, https://doi.org/10.1038/s41586-021-04068-z) requires rigorous calibration for the specific interfacial environment. Unlike the SHINERS technique used in Ref. 41 which provides extreme surface sensitivity, the standard in-situ Raman used here may suffer from significant interference from bulk water, potentially skewing the calculated population of 2-HB and 4-HB water. The peak positions (e.g., ~3540 cm-1 for hydrated water) are sensitive to the cation type (K^+ vs. Na^+) and ionic strength. The authors must justify the peak assignments in 1 M KOH instead of simply adopting values from a $NaClO_4$ system in the reference. Furthermore, the manuscript lacks a vibrational Stark slope analysis, which was a cornerstone in Ref. 41 to prove the ordered orientation of interfacial water under bias. Without this analysis, the claim that BTB promotes an "ordered water network" remains speculative.

4. The DFT analysis is restricted to thermodynamic descriptors such as adsorption energies and electronic structure, without evaluating any kinetic barriers for key elementary steps (e.g., C–C coupling or CO hydrogenation). Therefore, the calculations do not provide sufficient mechanistic insight into how BTB promotes the formation of multi-electron products. While the paper emphasizes the role of 4-coordinated hydrogen-bonded (4-HB) water, this species is completely absent from the DFT models. Most critically, the simulations employ a BTB molecule in neutral environment, failing to account for its deprotonated state in the strongly alkaline electrolyte.

5. The successful functionalization of BTB is only demonstrated in a dry state. Given that BTB undergoes deprotonation in 1 M KOH, the molecule's affinity for the Ag surface and its structural integrity may change significantly. The authors should provide spectroscopic evidence (e.g., FT-IR or XPS) of the electrode after rinsing with the 1 M KOH electrolyte to confirm that the BTB layer remains intact in the reaction environment.

6. The synthesis section provides only a qualitative description of the functionalization process. Essential parameters, such as the concentration of the BTB solution, immersion time, temperature, and the resulting molecular loading are entirely

missing. Without these quantitative metrics, the experiments are not reproducible.

7. The 23-hour stability duration is inadequate to demonstrate long-term durability. Beyond the risk of chemical decomposition (e.g., reductive debromination) under cathodic polarization, the organic layer may also undergo physical detachment or leaching from the electrode surface due to vigorous operational conditions. Consequently, extended stability tests and more robust evidence are required to substantiate the persistence and structural integrity of the functionalized layer throughout the electrolysis process.

8. The origin of the detected multi-carbon products requires rigorous validation. There is a potential concern that these products could arise from the degradation of the BTB molecule rather than CO_2 reduction. To address this, 13C isotopic labeling experiments are necessary to confirm that the carbon originates exclusively from the CO_2 feed.

Version 1:

Reviewer comments:

Reviewer #1

(Remarks to the Author)
the authors have completely addressed the above comments and I recommended this manuscript to be published.

Reviewer #2

(Remarks to the Author)
I have reviewed the revised manuscript and the accompanying response letter. I am satisfied to see that the authors have substantially addressed the majority of my previous concerns. The revisions have notably improved the quality and clarity of the paper. While there might be a few minor points to polish, the manuscript is now scientifically sound and I recommend it for publication.

Reviewer #3

(Remarks to the Author)
This reviewer has no further questions.

Open Access This Peer Review File is licensed under a Creative Commons Attribution 4.0 International License, which permits use, sharing, adaptation, distribution and reproduction in any medium or format, as long as you give appropriate credit to the original author(s) and the source, provide a link to the Creative Commons license, and indicate if changes were made.
In cases where reviewers are anonymous, credit should be given to 'Anonymous Referee' and the source.
The images or other third party material in this Peer Review File are included in the article's Creative Commons license, unless indicated otherwise in a credit line to the material. If material is not included in the article's Creative Commons license and your intended use is not permitted by statutory regulation or exceeds the permitted use, you will need to obtain

permission directly from the copyright holder.
To view a copy of this license, visit https://creativecommons.org/licenses/by/4.0/

Responses to the Comments of the Reviewers

We thank the reviewers for the constructive comments and suggestions to help us further improve the manuscript titled "***Hydrogen Bond Stabilized *CO Intermediate Enables CO_2 Electroreduction to Multi-Electron Products on Silver Catalysts***". We have addressed all the comments point-by-point and revised the manuscript accordingly. In this response letter, comments from the referees are summarized in blue typeface and our responses are in black typeface. All changes have been highlighted in yellow in the main text. We hope that the detailed responses and the changes we have made to the manuscript now make it suitable for publication in ***Nature Communications***.

A point-to-point response to the comments

Responses to Reviewer 1:

Zhang et al. develops a molecular modification strategy to enhance CO2 electroreduction on silver catalysts. By anchoring bromothymol blue (BTB) on Ag surfaces, a hydrogen-bonding network stabilizes the *CO intermediate, prolonging its residence time and enabling further hydrogenation and C-C coupling. This shifts product selectivity from primarily CO to multi-electron products like CH4 and C2H4, achieving a 24.2% Faradaic efficiency at 400 mA cm-2. The work highlights a paradigm shift from metal-centric tuning to local microenvironment engineering, offering a general approach to activate non-Cu catalysts for deep CO2 reduction. This manuscript can be accepted after solving the below comments:

Response: We sincerely thank you for your thoughtful evaluation. We have carefully considered all the comments and have revised the manuscript accordingly.

Q1. The authors propose a novel mechanism to stabilize CO molecule via the interaction of H-bond. However, the stability analysis is mainly through the results of C-O bond strength, such as in Figure 5b and Figure S25, indicating enhanced C-O interactions. Mostly, the activation of C-O bond is beneficial for the following hydrogenation of CO to multi-electron species. How about the Ag-C bond strength? You should further evaluate the Ag-C interaction via COHP or other electronic structure methods.

Response: We deeply appreciate the reviewer's highly professional and perceptive comment. The reviewer has acutely identified the kinetic prerequisite for generating multi-electron products on a silver catalyst: the intrinsic Ag-C bond must be sufficiently strengthened to prevent the premature desorption of the *CO intermediate, thereby allowing subsequent hydrogenation steps to occur. We completely agree that while C-O activation is crucial, robust Ag-C anchorage is equally indispensable.

To rigorously address this, we followed the reviewer's excellent suggestion and evaluated the quantum mechanical nature of the Ag–C interaction using Crystal Orbital Hamilton Population (COHP) analysis (Figure 5b). By integrating the COHP up to the Fermi level (ICOHP), we quantified the covalent strength of the Ag–C bond. Our newly added calculations reveal that the ICOHP value for the Ag–C bond in the Ag-BTB system is significantly more negative than that on the bare Ag surface (-1.639 eV vs. -0.398 eV). In solid-state quantum chemistry, a more negative ICOHP value explicitly indicates an increased accumulation of bonding states and a stronger covalent interaction. This proves that the BTB modifier thoroughly anchors the *CO intermediate, effectively preventing its premature desorption and establishing the necessary prerequisite for subsequent multi-electron reduction. Classically, injecting extra electrons into the adsorbate populates the 2π* antibonding orbitals and weakens the C–O bond, which is typically considered beneficial for activating CO toward multi-electron species. However, in our system, the C–O interaction is actually strengthened (as indicated by PDOS overlap Supplementary Figure S28 and a more negative C–O ICOHP Supplementary Figure S31). We attribute this apparent anomaly to a unique “push-pull” electronic effect. Specifically, the interfacial hydrogen-bonding network (O···H–O) from the BTB modifier exerts a strong pull on the adsorbate's electron density. This localized polarization creates a thermodynamic driving force that extracts a greater extent of electron transfer from the Ag substrate to the *CO adsorbate, as confirmed by the increased Bader charge (Supplementary Figure S29-30). Consequently, the substrate “push” is enhanced, populating Ag–C bonding states and significantly strengthening the Ag–C anchoring bond. Crucially, because of this concurrent Ag–C strengthening and H-bond pulling, the transferred electrons do not populate the C–O antibonding orbitals to cause bond cleavage. Instead, the C–O bond undergoes severe electronic polarization. The excess electron density is highly localized on the oxygen terminus, transforming the *CO intermediate into an extremely electron-rich nucleophilic center while

maintaining a robust C–O structural framework. This structural stabilization does not imply kinetic inertness; rather, it dictates the regioselectivity of the subsequent hydrogenation. Our DFT calculations (Figure 5f-h) perfectly corroborate this hypothesis, revealing that the first proton-coupled electron transfer (PCET) optimally proceeds via protonation at the oxygen atom to form *COH, effectively bypassing the conventional *CHO intermediate. Subsequently, it undergoes a highly favorable asymmetric C–C coupling with an adjacent *CO monomer (*CO + *COH → *OCCOH). These calculations and discussions have been added into the revised Manuscript and the Supplementary Information.

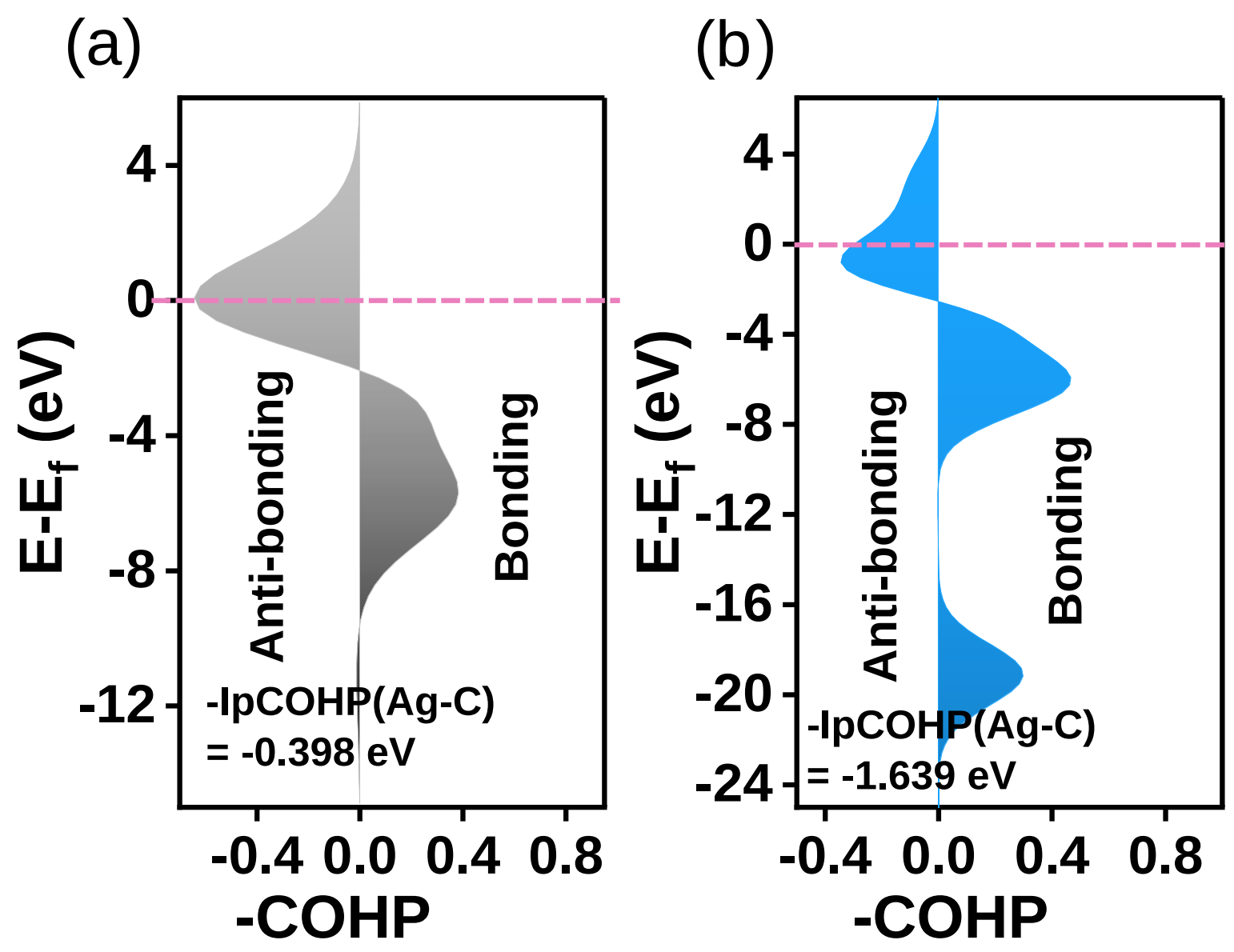

Figure 5b. COHP curves for the Ag-C bonds in *CO on electrode surfaces.

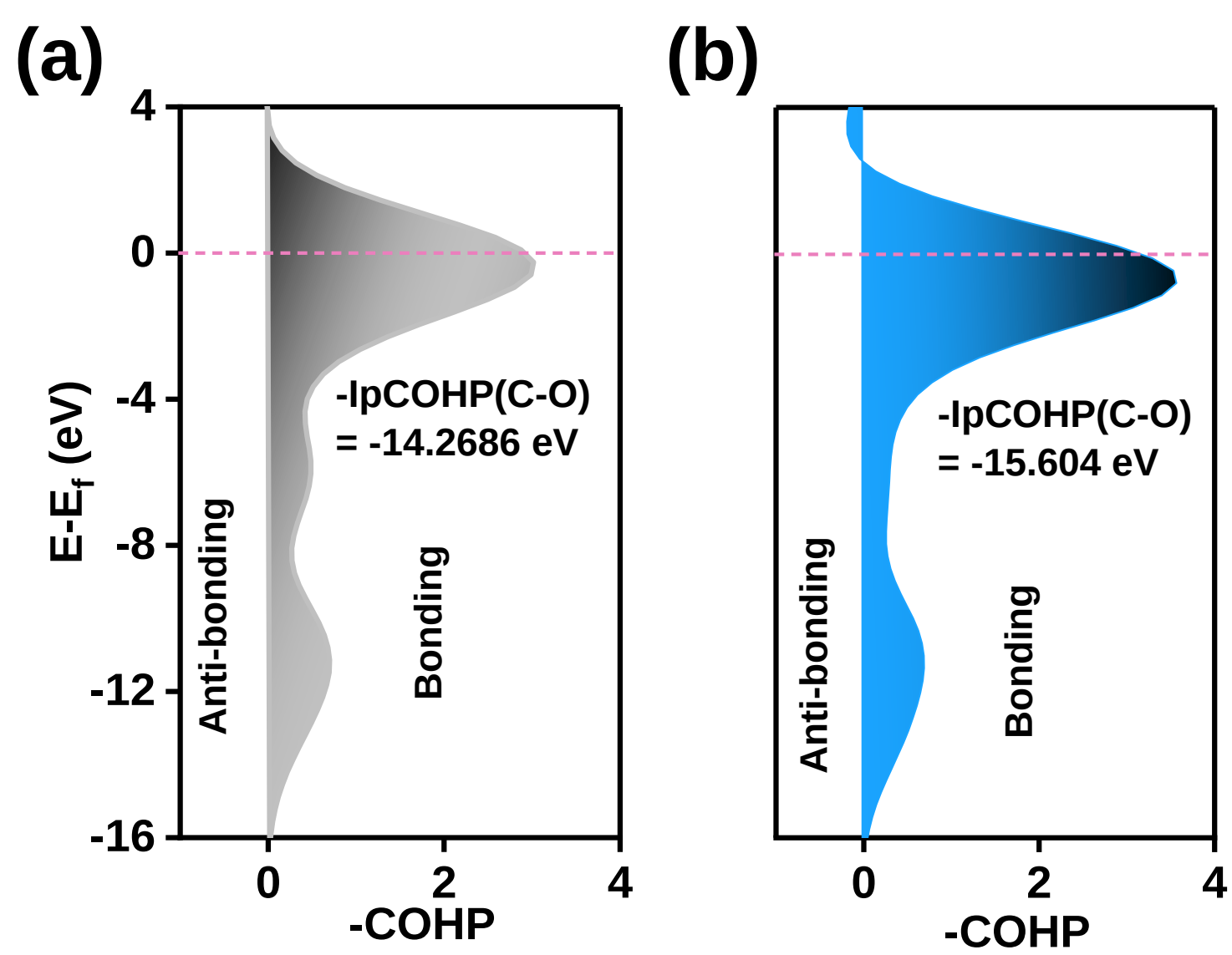

Figure S31. COHP curves for the C-O bonds in *CO on electrode surfaces.

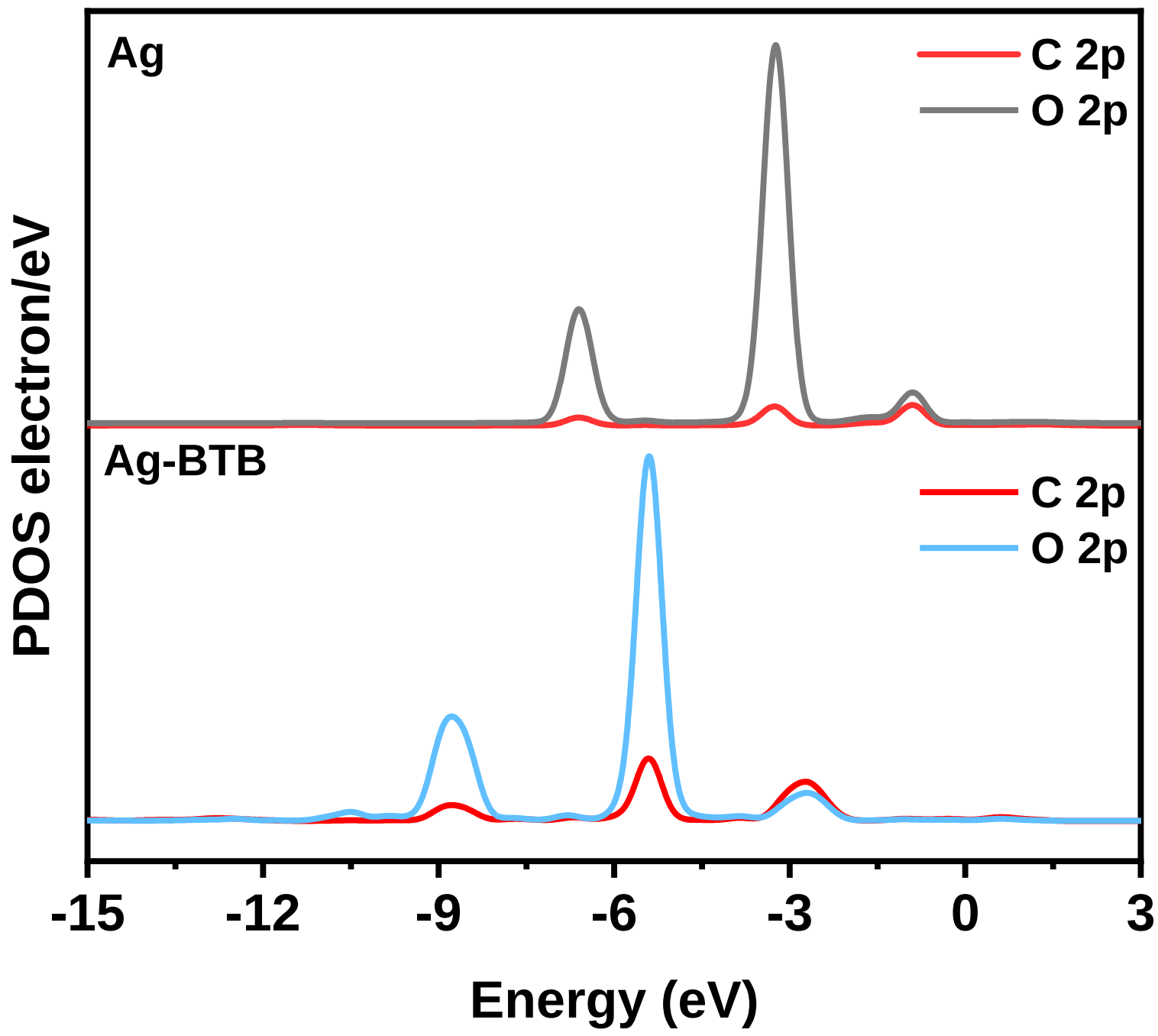

Figure S28. PDOS plots of the O 2p orbital and C 2p orbital of *CO adsorbed on Ag (111) and Ag (111)-BTB.

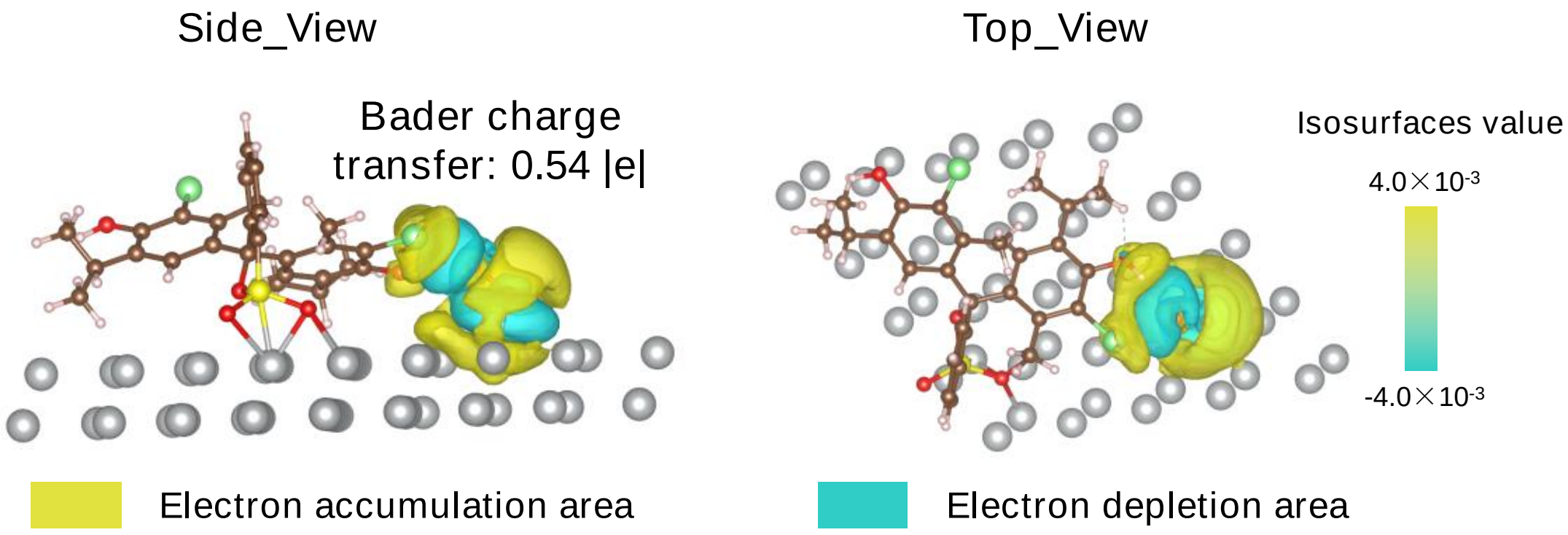

Figure S29. Charge density difference maps and Bader charge analysis for *CO adsorbed on Ag-BTB surfaces. Light gray, brown, red, yellow, green, and light pink balls correspond to Ag, C, O, S, Br, and H atoms, respectively.

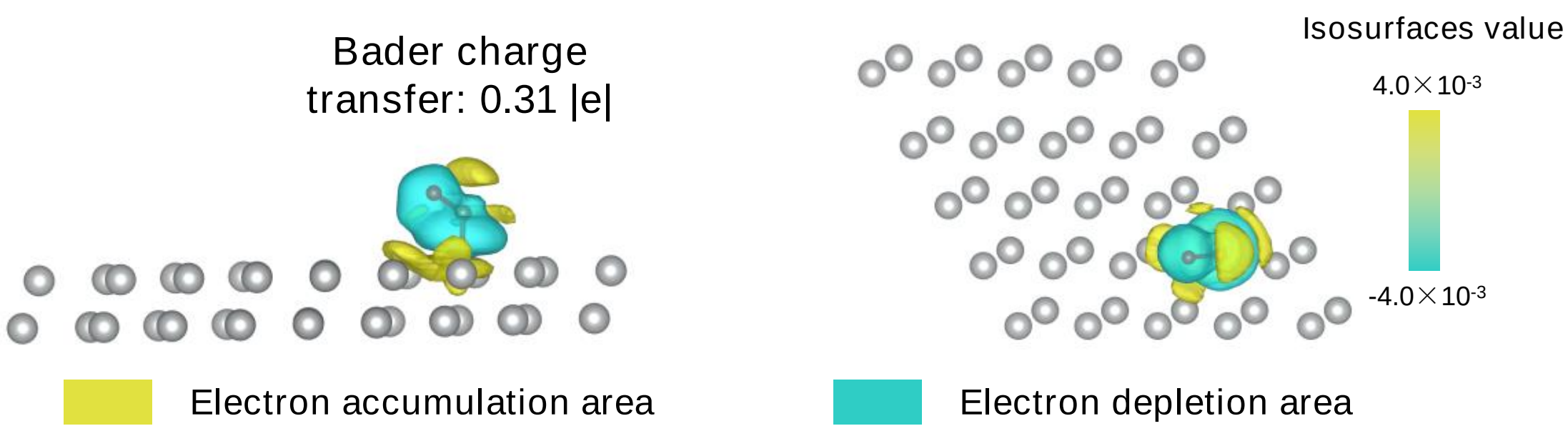

Figure S30. Charge density difference maps and Bader charge analysis for *CO adsorbed on pristine Ag surfaces. Light gray, brown, and red balls correspond to Ag, C, and O atoms, respectively.

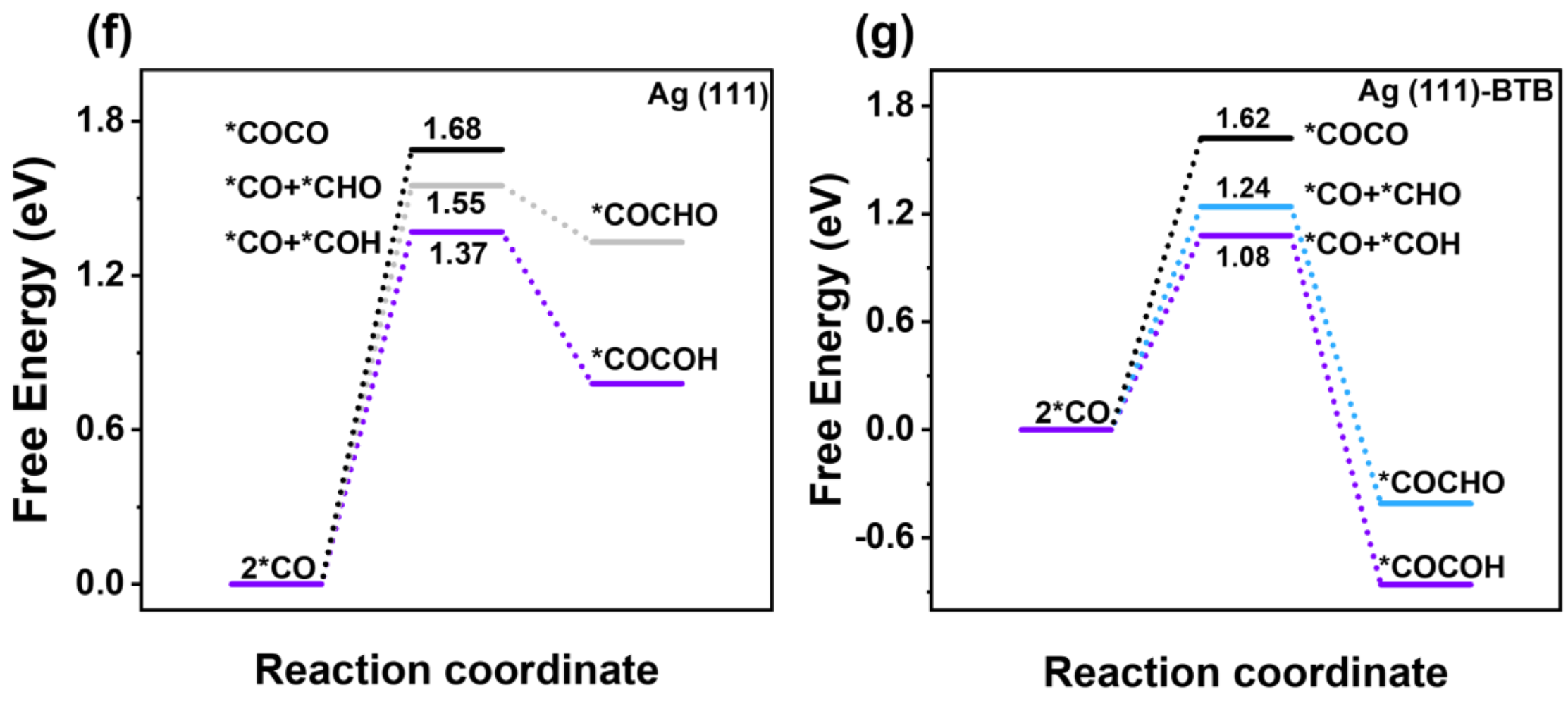

Figure 5f-g. Free energy profiles of the *CO hydrogenation and coupling steps on (f) Ag (111) and (g) Ag (111)-BTB.

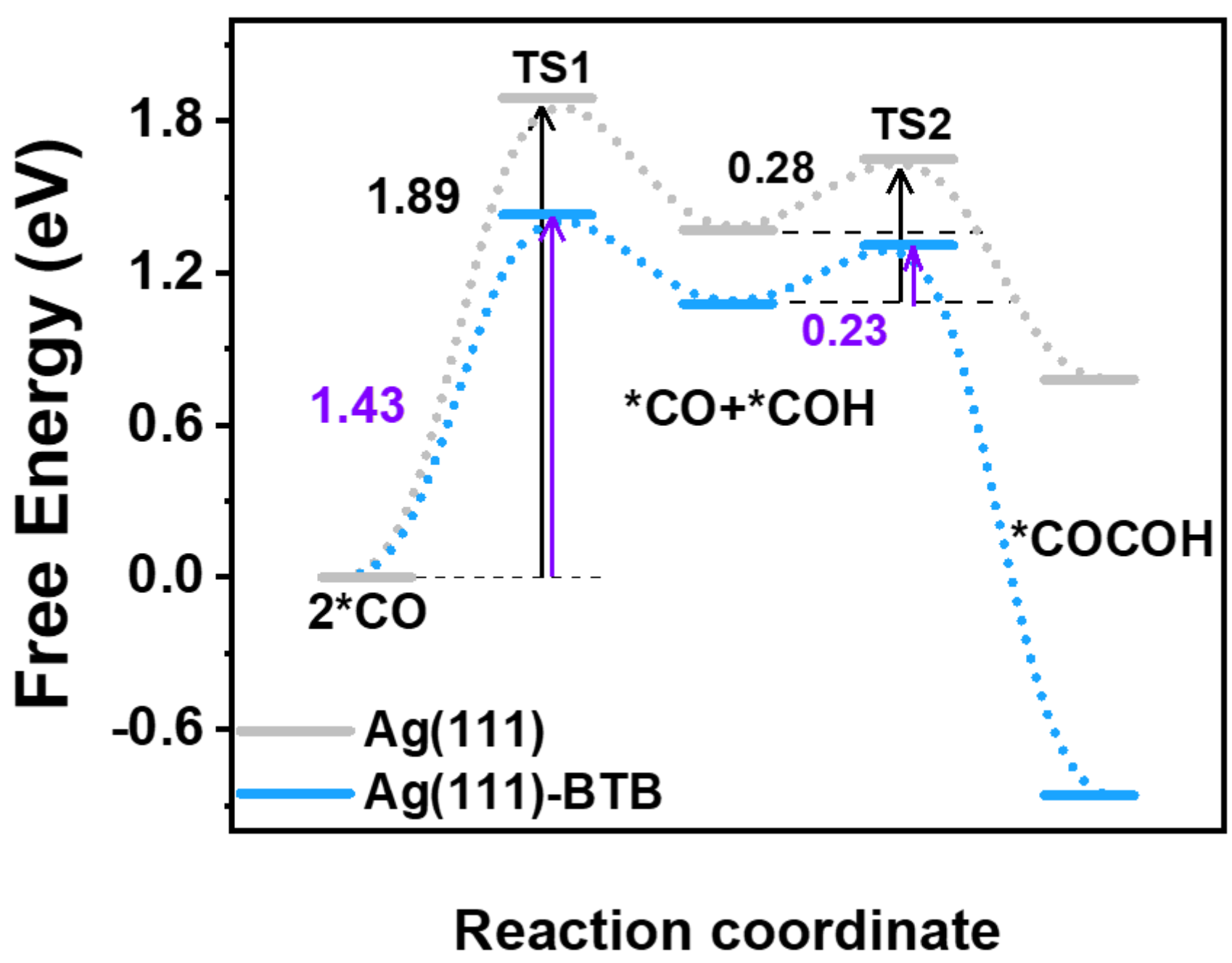

Figure 5h. Kinetic analysis of the transition states associated with *CO hydrogenation and C-C coupling.

Q2. In order to quantity the interfacial interaction of CO and BTB, you can also add the Bader charge analysis, combining with the differential charge density result, compared with the pristine Ag surface.

Response: We sincerely thank the reviewer for this insightful suggestion. We totally agree that combining Bader charge analysis with differential charge density results provides a more comprehensive and quantitative understanding of the interfacial interaction.

We have systematically calculated and compared the differential charge density and Bader charge transfer for CO adsorption on both the pristine Ag surface and the Ag-BTB surface. The results have been added to the revised manuscript. As shown in the new Supplementary Figure S29-30, the differential charge density plots clearly illustrate a much more pronounced spatial electron redistribution (larger electron accumulation and depletion regions) when *CO is adsorbed on the Ag-BTB surface than on the pristine Ag surface. Quantitatively, the Bader charge analysis reveals that the electron transfer from the substrate to the *CO molecule significantly increases from 0.31 |e| (on pristine Ag) to 0.54|e| (on Ag-BTB). These combined visual and quantitative results strongly confirm that the introduction of the BTB molecule effectively modulates the local electronic structure, thereby significantly enhancing the interfacial interaction of *CO. We have added the detailed discussions in the revised Manuscript.

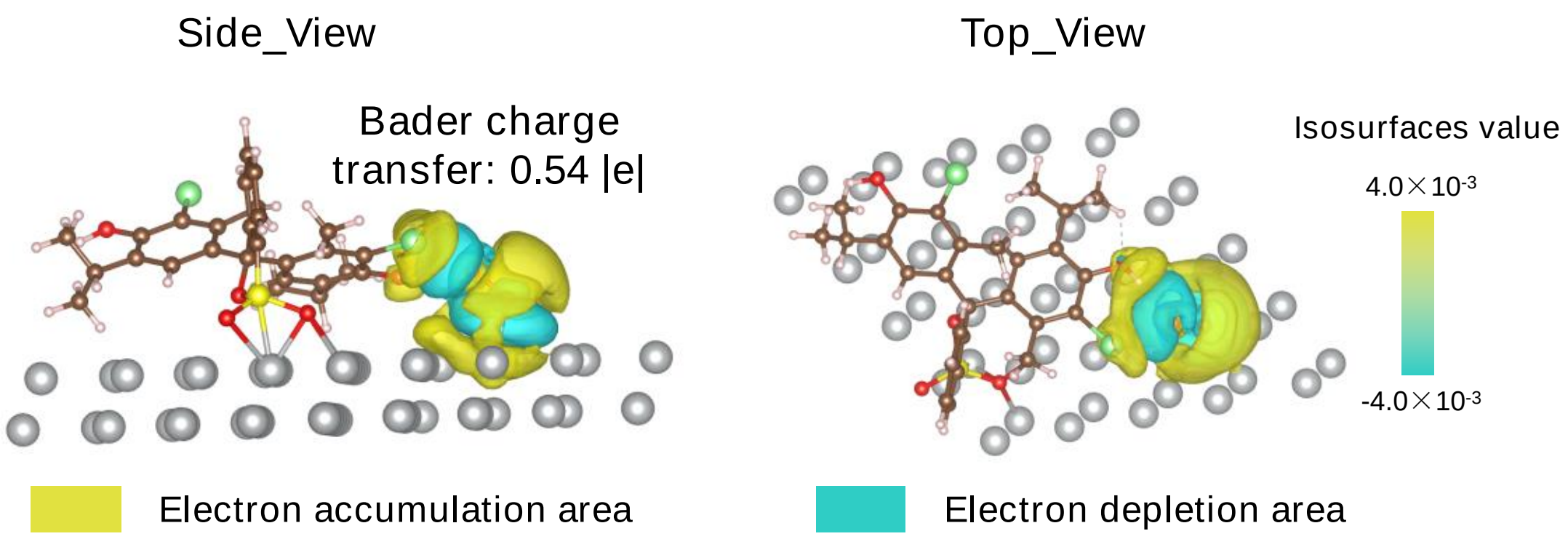

Figure S29. Charge density difference maps and Bader charge analysis for *CO adsorbed on Ag-BTB surfaces. Light gray, brown, red, yellow, green, and light pink balls correspond to Ag, C, O, S, Br, and H atoms, respectively.

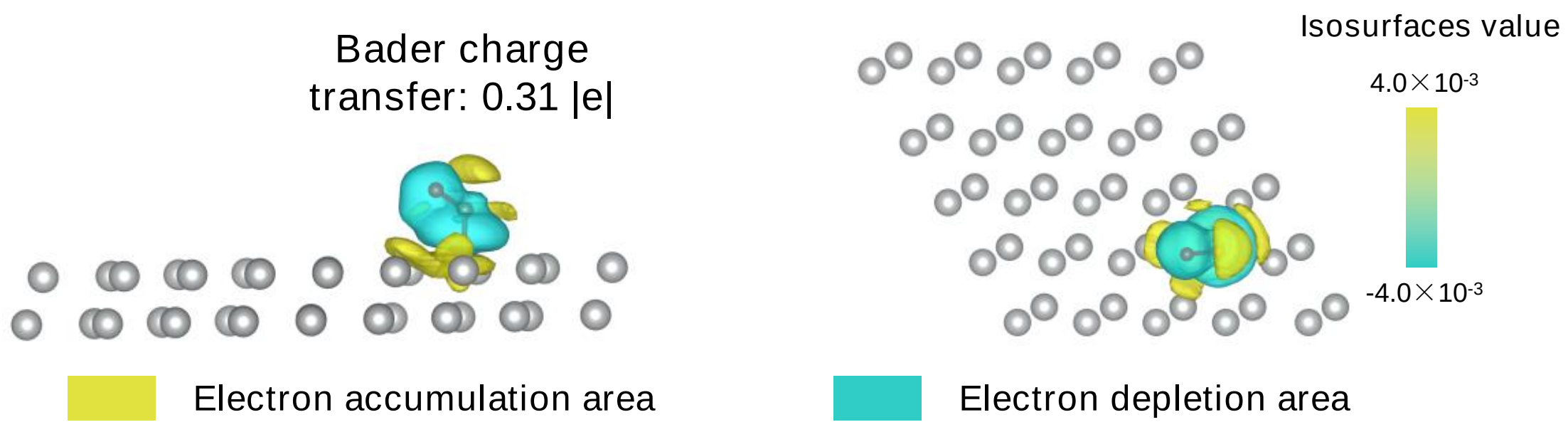

Figure S30. Charge density difference maps and Bader charge analysis for *CO adsorbed on pristine Ag surfaces. Light gray, brown, and red balls correspond to Ag, C, and O atoms, respectively.

Q3. To clarify the stability of CO in the Ag (111)-BTB via MD results, you can count the height of C and O atom of CO on the Ag (111) surface in the entire trajectory of 200 ps. Additionally, the

potential energy statistics results cannot illustrate the stability because the CO-Ag (111)-BTB system owns more atoms than CO-Ag (111). Please carefully think over it.

Response: We sincerely thank the reviewer for pointing out this critical methodological flaw and for providing such a constructive and standard alternative. We fully agree with your rigorous assessment: comparing the statistical potential energies of two systems with different numbers of atoms (CO-Ag (111)-BTB vs. CO-Ag (111)) is physically inappropriate and cannot serve as a valid metric for thermodynamic stability. We apologize for this oversight and have entirely removed the flawed potential energy comparison from the revised manuscript. We have extracted the distance (height) evolution of both the C and O atoms of the adsorbed *CO relative to the Ag (111) surface over the entire 200 ps Molecular Dynamics (MD) trajectory (Figure 6c and Supplementary Figure S38). The distance evolutions for both atoms exhibit highly consistent trends. Notably, the trajectories reveal that the *CO intermediate at the Ag-BTB interface is firmly anchored, remaining significantly closer to the electrode surface than on pristine Ag. This geometric evidence unambiguously demonstrates that the BTB-modification strategy successfully suppresses *CO desorption. The corresponding data and discussions have been added to the revised Manuscript and the Supplementary Information.

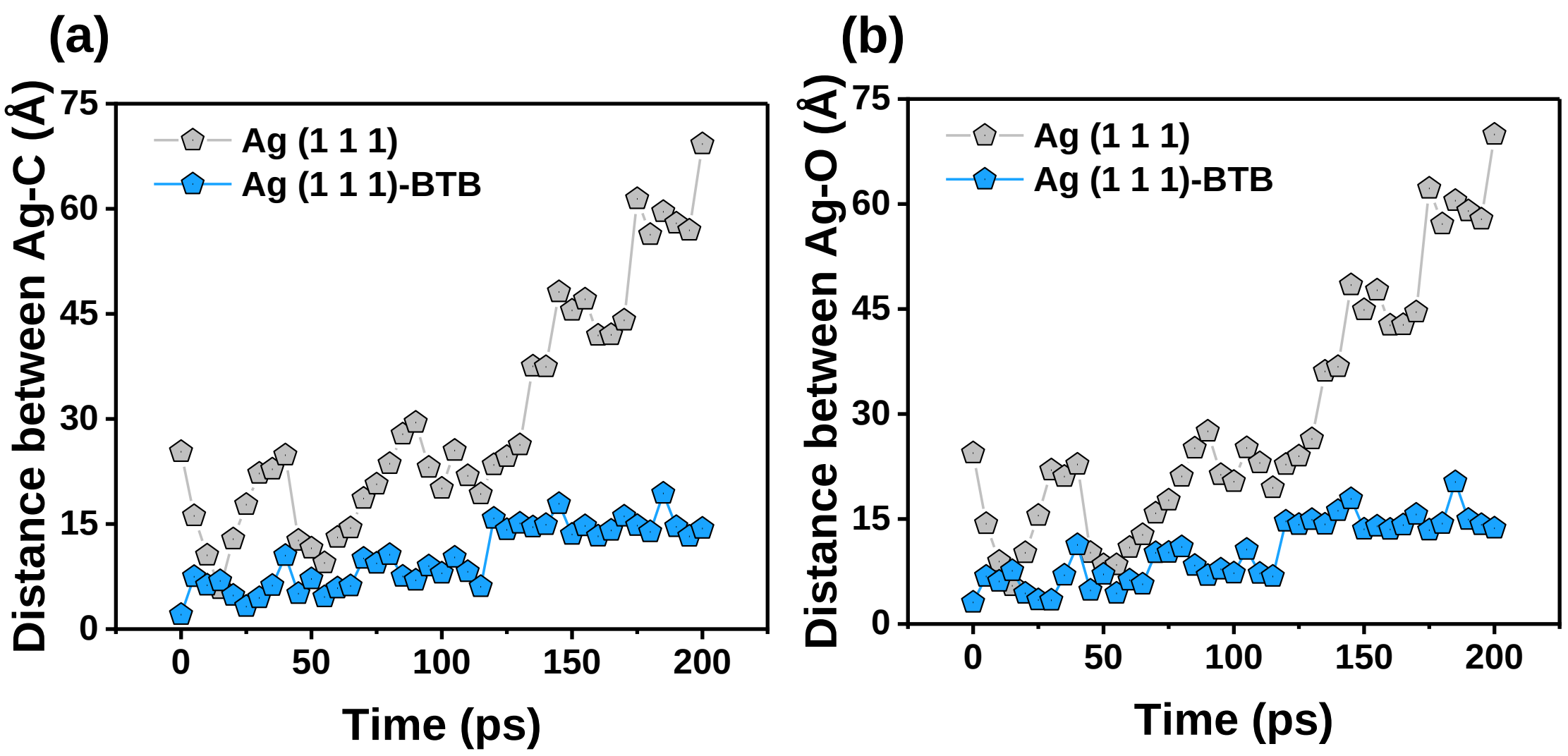

Figure 6c and **S38.** Distance of the C and O atom in *CO from the Ag surface along the AIMD trajectory.

Q4. In the computational details, do you consider the effect of solvation and Van der Waals correlation on the adsorption of reactant and intermediates, specifically for involving of large

molecule BTB. Additionally, the computational methods were very simple, please supplement it, such as, the computational hydrogen electrode method, adsorption energy formula and MD details.

Response: We sincerely thank the reviewer for pointing out these critical methodological details. We completely agree that clarifying the treatments of Van der Waals (vdW) correlation and solvation effects, as well as providing explicit formulas, is essential for reproducibility, especially when handling a large molecule like BTB. We have substantially expanded our computational methods section in the revised manuscript to address all these points based on your constructive suggestions. As the reviewer rightly pointed out, standard PBE functionals are insufficient for describing long-range vdW forces. For the adsorption of large molecules such as BTB, dispersion interactions often contribute a substantial proportion to the overall adsorption energy. As detailed in our revised manuscript, the electron exchange-correlation interactions in our calculations were treated using the Perdew-Burke-Ernzerhof functional (GGA-PBE) with Grimme's D3 dispersion correction. This correction effectively captures the vdW interactions in the DFT calculations. Additionally, in our MD simulations, the atom-based cutoff method was explicitly applied to treat vdW interactions. In our primary Gibbs free energy calculations, a vacuum approximation was employed to evaluate the intrinsic thermodynamic trends. However, we fully agree with the reviewer that the solvent environment can influence the adsorption behavior. To rigorously address this, we supplemented our study by calculating the adsorption energies (Figure 5a) and performing an independent gradient model based on Hirshfeld partition (IGMH) analysis (Figure 5d) using an implicit solvation model. The validation results demonstrated that the variation trends of the adsorption energies under the solvation model were highly consistent with those obtained under vacuum conditions. Moreover, the IGMH analysis under the implicit solvation model visually confirmed the presence of intermolecular hydrogen bonding interactions between the Ag-BTB interface and the *CO intermediate. We have explicitly stated this in the revised manuscript. We have fully supplemented the methodology section, integrating the specific formulas and parameters. We are very grateful to the reviewer for helping us improve the completeness and rigor of our methodology.

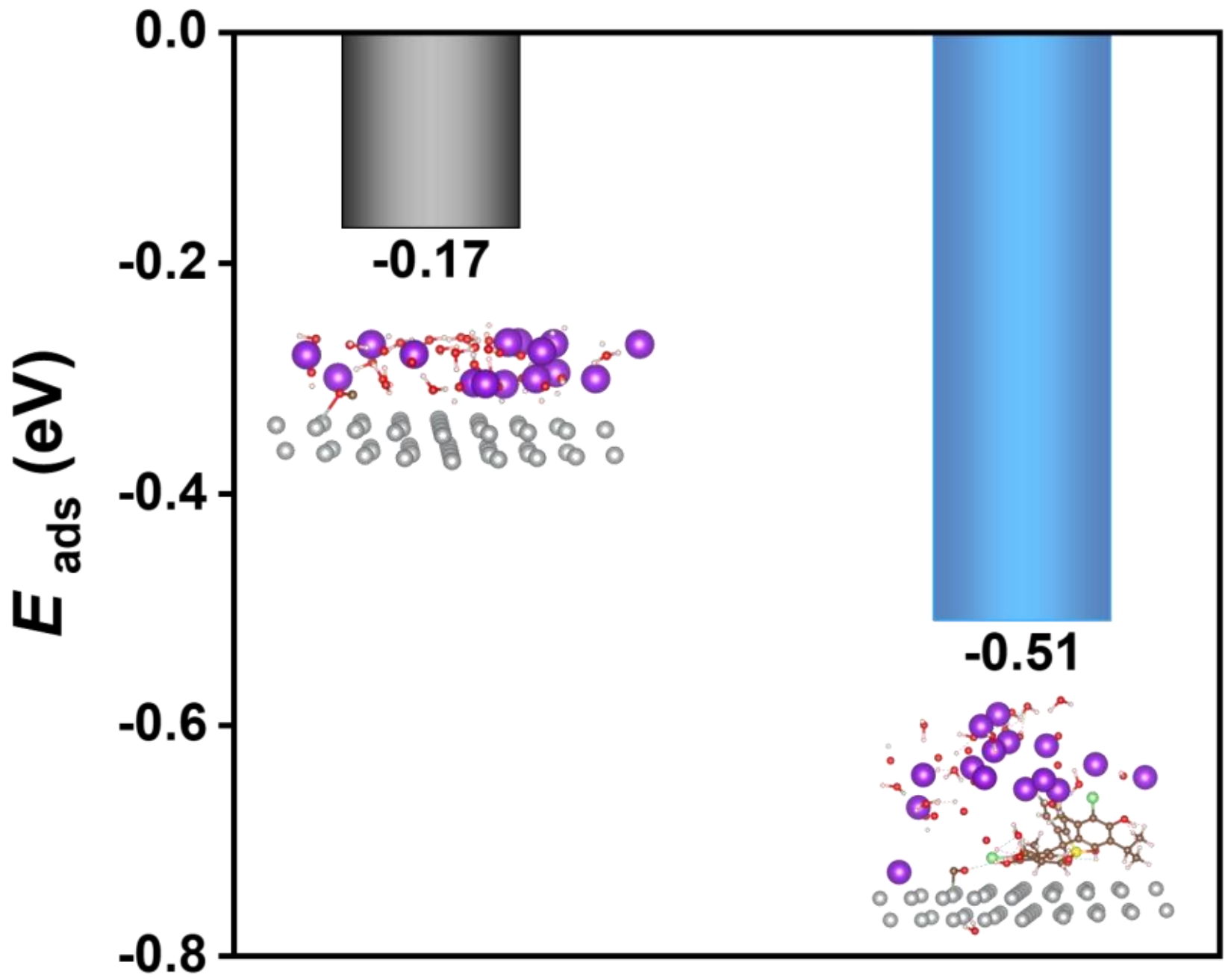

Figure 5a. The *E*ads of *CO on the electrode surfaces. Light gray, brown, red, yellow, green, light pink, and purple balls correspond to Ag, C, O, S, Br, H, and K atoms, respectively.

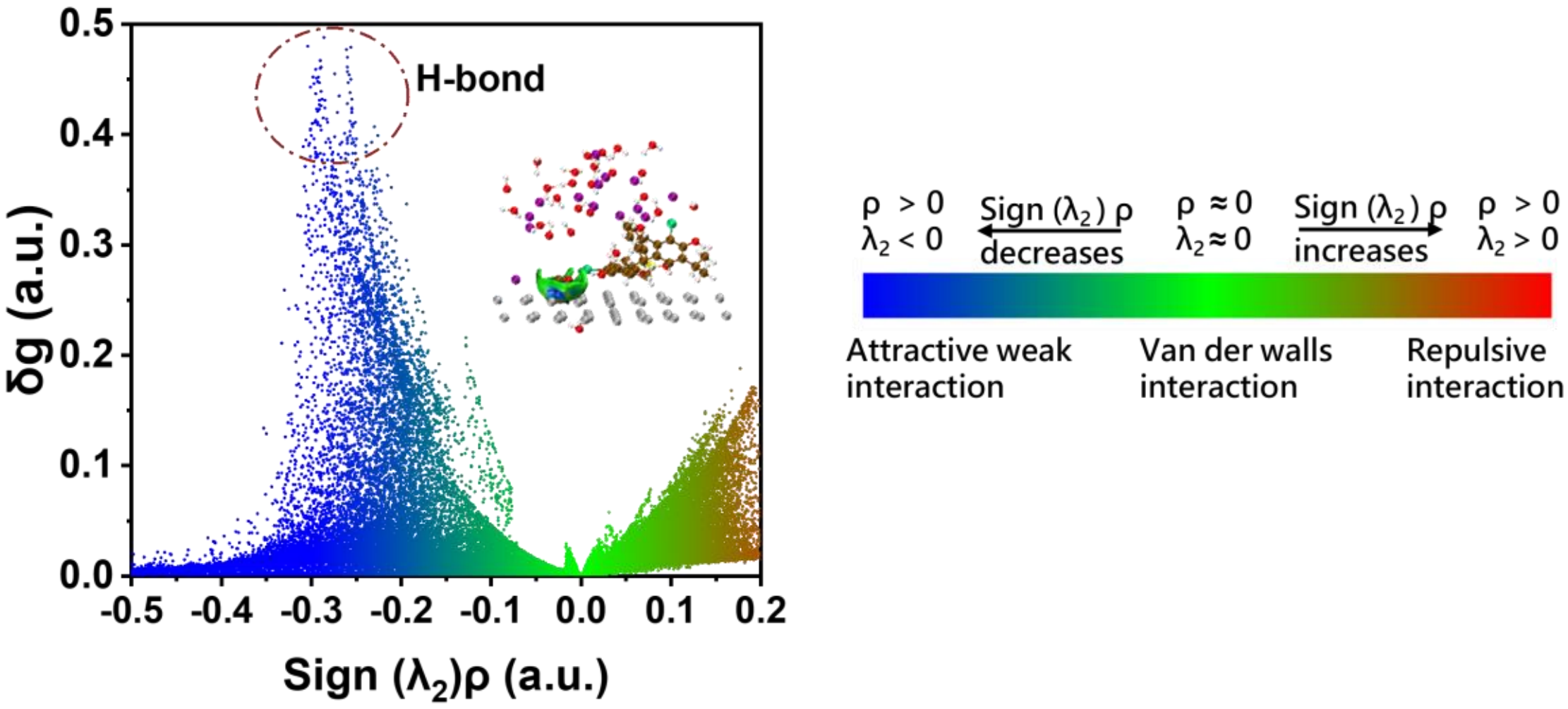

Figure 5d. Independent gradient model based on Hirshfeld partition (IGMH) analysis of the interaction between Ag-*CO and BTB, blue represents strong attraction, green represents van der Waals interaction, and red represents strong repulsion. Light gray, brown, red, yellow, green, light pink, and purple balls correspond to Ag, C, O, S, Br, H, and K atoms, respectively.

(The following content has been added to the revised Supporting Information)

DFT calculation

All first-principles density functional theory (DFT) calculations were performed using the Vienna Ab initio Simulation Package (VASP). The electron exchange-correlation interactions were treated within the generalized gradient approximation using the Perdew-Burke-Ernzerhof functional

(GGA-PBE) with Grimme's D3 dispersion correction, while ion-electron interactions were described by the projector augmented wave (PAW) method. We employed a plane-wave basis set with a kinetic energy cutoff of 450 eV. For Brillouin zone integration, a 3×3×1 Monkhorst-Pack k-point mesh was used during structural optimization. Structural optimizations were performed until the energy change between two electronic self-consistent steps was less than 10^{-5} eV, and the Hellmann-Feynman forces on all atoms were converged to below 0.02 eV/Å. The adsorption energy (E_{ads})was defined according to the equation:

$$E_{\mathrm{ads}} = E_{\mathrm{substrate+adsorbate}} - E_{\mathrm{substrate}} - E_{\mathrm{adsorbate}}$$

Where $E_{\mathrm{substrate+adsorbate}}$ is the total energy of the slab with the adsorbate, $E_{\mathrm{substrate}}$ is the energy of the clean optimized Ag or Ag-BTB slab, and $E_{\mathrm{adsorbate}}$ is the energy of the isolated adsorbate molecule in the gas phase. By this definition, a more negative E_{ads} value indicates a stronger binding interaction. The transition state (TS) structures and reaction pathways were determined using the climbing image nudged elastic band (CI-NEB) method.

To investigate the electrochemical reaction pathways, the free energy change (ΔG) for each elementary step involving coupled proton-electron transfer was calculated within the framework of the computational hydrogen electrode (CHE) model. In this model, the chemical potential of the ($H^+ + e^-$) pair is referenced to half of the chemical potential of gas-phase H_2 at standard conditions (pH = 0, U = 0 V, T = 298.15 K). Accordingly, the free energy was calculated as:

$$\Delta G = \Delta E + \Delta E_{ZPE} - T\Delta S + \Delta G_{pH} + \Delta G_U$$

Where ΔE denotes the electronic energy difference obtained from DFT calculations. $\Delta G_{\mathrm{pH}} = -k_B T \ln_{10} \times \mathrm{pH}$ accounts for the pH dependence and $\Delta G_U = -neU$ accounts for the effect of the electrode potential U. In this work, ΔE_{ZPE} and ΔS were derived from vibrational frequency calculations and standard thermodynamic data, respectively. The temperature T was set to 298.15 K for all reaction systems.

The charge density differences were analyzed by VASPKIT code. In order to quantitatively analyze the bonding properties of CO adsorbed on Ag-BTB and characterization of charge transfer, the charge densities of CO in the corresponding compounds were extracted from the corresponding charge densities in the Ag-BTB substrates to assess the differences in the charge densities of CO adsorbed on the constructed Ag-BTB models. The level of charge transfer between CO and Ag-BTB was calculated using a Bader charge analysis program.

$$\Delta\rho = \rho(\text{CO/Ag-BTB}) - \rho(\text{Ag-BTB}) - \rho(\text{CO})$$

Independent gradient model based on Hirshfeld partition (IGMH) simulations were performed with the Multiwfn program to investigate the type of interaction force. Obviously, H-bonding interactions between CO and Ag-BTB is revealed when the value of sign(λ_2)ρ approaches zero.

Molecular dynamic (MD) simulations

Molecular dynamics (MD) simulations were performed using the Forcite module with the COMPASS force field. The simulation systems comprised a periodic Ag(111) slab model, a layer of explicitly modeled BTB molecules (for the modified system), and a CO molecule. After geometric optimization, the systems were equilibrated under the NPT ensemble for 200 ps. Subsequently, the production runs were performed under the NVT ensemble for 200 ps to collect the dynamic trajectory data. Temperature was regulated using a Nosé-Hoover thermostat. Electrostatic and van der Waals (vdW) interactions were treated using the Ewald summation and an atom-based cutoff method (12.5 Å radius), respectively. The vertical trajectory distance between the carbon atom of CO and the uppermost layer of the Ag (111) surface was systematically extracted to evaluate dynamic spatial confinement.

Q5. In recent years, there are a series of latest work about multi-carbon products of CO_2RR in the Cu-based and non-Cu based electrocatalysts that you can refer. Such as, The Journal of Physical Chemistry Letters, 2025, 16 (42), 11016-11024; Nature Communications, 2025, 16 (1), 2217; Nature Communications, 2025, 16 (1), 1203.

Response: We sincerely thank the reviewer for recommending these highly relevant and outstanding recent publications. We completely agree that including the latest advancements in both Cu-based and non-Cu-based electrocatalysts for CO_2 reduction to multi-carbon (C_{2+}) products is crucial for providing a comprehensive and up-to-date background for our study.

We have carefully studied these excellent pioneering works. They provide profound insights into the regulation of microenvironments, structural design, and C-C coupling mechanisms, which highly resonate with the motivation of our current work. We have incorporated these three important references into the Introduction and Mechanism section of our revised manuscript to discuss the state-of-the-art strategies for multi-carbon ($C_{2+)}$ product generation. Specifically, they have been

added as References [10], [11], and [47]. We greatly appreciate your help in broadening the scope and improving the literature comprehensiveness of our paper.

Q6. All the atoms in the diagram of atomic models should added the atomic representatives in order to distinguish the identity of atom in the Figures 6 and S22, S23, S26-S30.

Response: We sincerely thank the reviewer for this meticulous and constructive suggestion. We fully agree that providing clear atomic representatives is essential for readers to accurately distinguish and understand the structural models and reaction intermediates. We have now added explicit text descriptions of the atomic color codes directly into the captions of Figure 5-6 and Supplementary Figure S26,S29,S32-36. Specifically, we have clarified that light blue, grey, red, yellow, reddish-brown, and white balls represent Ag, C, O, S, Br, and H atoms, respectively, while light gray, brown, red, yellow, green, light pink, and purple balls correspond to Ag, C, O, S, Br, H, and K atoms, respectively (Figure R1). We greatly appreciate the reviewer's careful and thorough review.

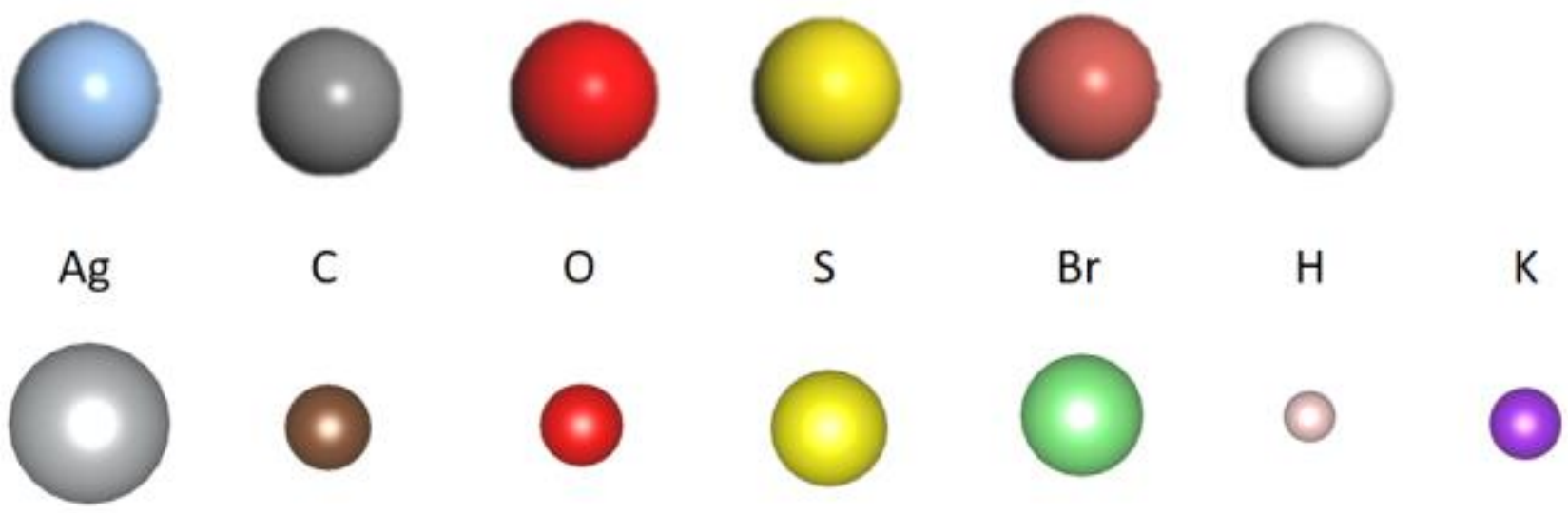

Figure R1. Atomic representation of differently colored balls.

Q7. For the differential charge density, the isosurface values of the contour should be denoted in Figure S23.

Response: We sincerely thank the reviewer for this careful observation. We completely agree that specifying the exact isosurface value is essential for an accurate and quantitative representation of the differential charge density. We have updated the differential charge density plot. As shown in the newly revised Figure S29-30, we have explicitly added the isosurface value label directly into the figure to ensure complete clarity for the readers. We appreciate your rigorous review, and this corrected figure has now been incorporated into the revised Supplementary Information.

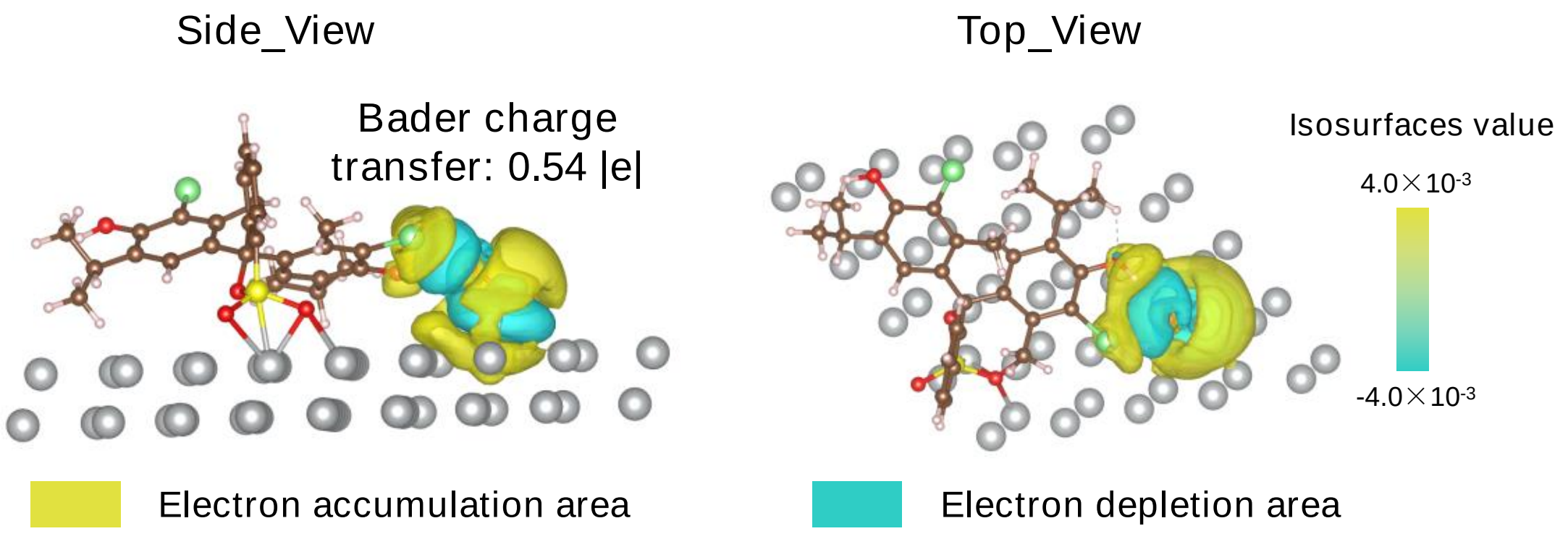

Figure S29. Charge density difference maps and Bader charge analysis for *CO adsorbed on Ag-BTB surfaces. Light gray, brown, red, and light pink balls correspond to Ag, C, O, and H atoms, respectively.

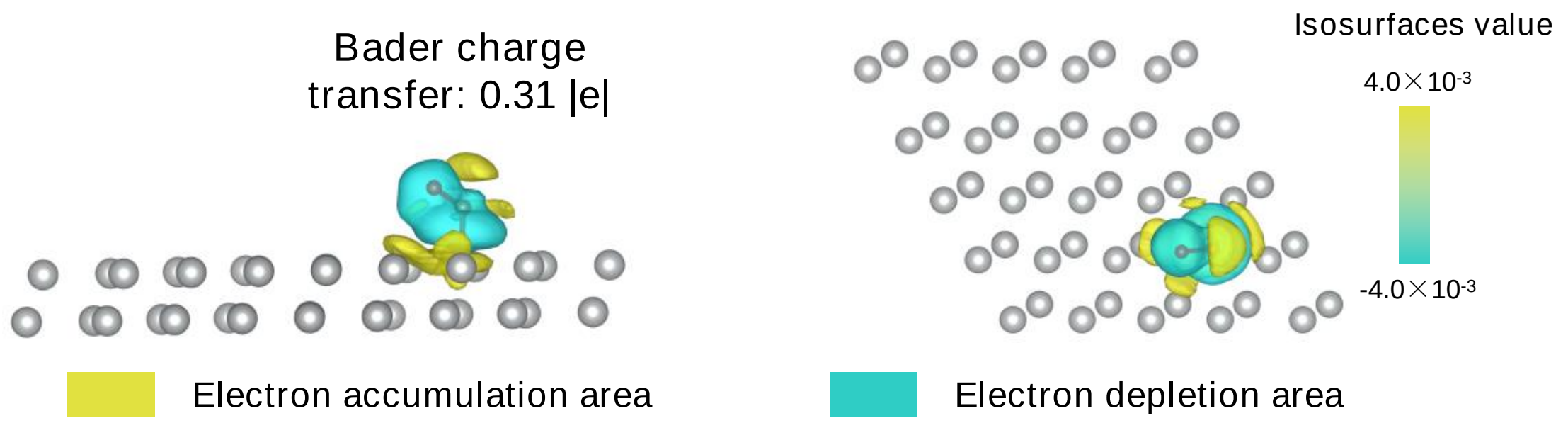

Figure S30. Charge density difference maps and Bader charge analysis for *CO adsorbed on pristine Ag surfaces. Light gray, brown, and red balls correspond to Ag, C, and O atoms, respectively.

Thank you again for reviewing this manuscript. These constructive suggestions have made a real improvement of our work. We truly appreciate your help.

Responses to Reviewer 2:

The manuscript presents an innovative molecular modification strategy using bromothymol blue (BTB) to enhance CO_2 electroreduction on Ag electrodes, addressing the long-standing challenge of weak *CO intermediate adsorption. The reported 24.2% Faradaic efficiency (FE) for multi-electron products at high current density is noteworthy, and the shift from metal-centric to microenvironment engineering is conceptually appealing. The integration of in situ spectroscopy and theoretical simulations strengthens the mechanistic insights. Thus, I recommend accept after major revision.

Response: We sincerely thank you for your thoughtful evaluation. We have carefully considered all the comments and have revised the manuscript accordingly.

Q1. The authors chose to modify the Ag surface with BTB molecules, attributing this to the role of OH. Besides BTB, there are other molecules with OH functional groups. Why was BTB selected? Have other molecules been tried? Please explain the specific reasons for choosing the BTB molecule.

Response: We sincerely thank the reviewer for raising this highly practical and insightful question. It is entirely valid to ask why bromothymol blue (BTB) was specifically selected over simpler molecules containing phenolic or aliphatic -OH groups. To be completely transparent, our selection of BTB did not stem from an immediate stroke of rational microenvironment design. Instead, it evolved from an initial diagnostic ambition, an honest experimental setback, and a subsequent systematic screening. We detail the chronological and physicochemical rationale below: Our primary scientific motivation for introducing BTB was explicitly to exploit its classic pH-dependent colorimetric transitions as an *in situ* optical probe to visualize local pH fluctuations at the working interface. We must candidly admit that this specific *operando* diagnostic attempt was ultimately unsuccessful. The inherent complexities of the gas diffusion electrode (GDE), specifically the severe light scattering caused by vigorous gas evolution at high current densities and the signal-to-noise limitations of our flow cell window, prevented us from capturing reliable, quantitative spectroscopic signals. Nevertheless, this ambitious goal dictated our initial molecular selection. Although the *operando* pH probing failed, the dye nature of BTB provided an unexpected but crucial practical benefit. BTB possesses a remarkably high molar extinction coefficient, which offered a

straightforward visual method to rigorously confirm the successful and uniform functionalization of the Ag catalyst surface.

(a)

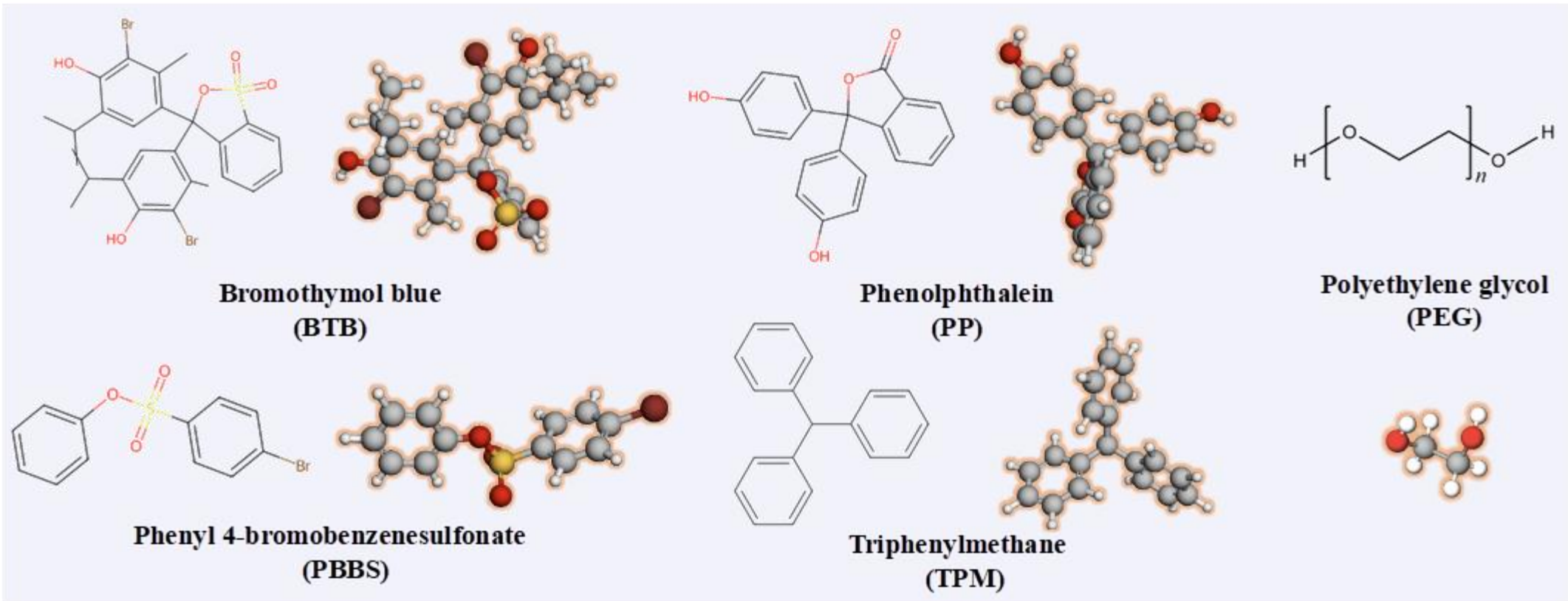

(b)

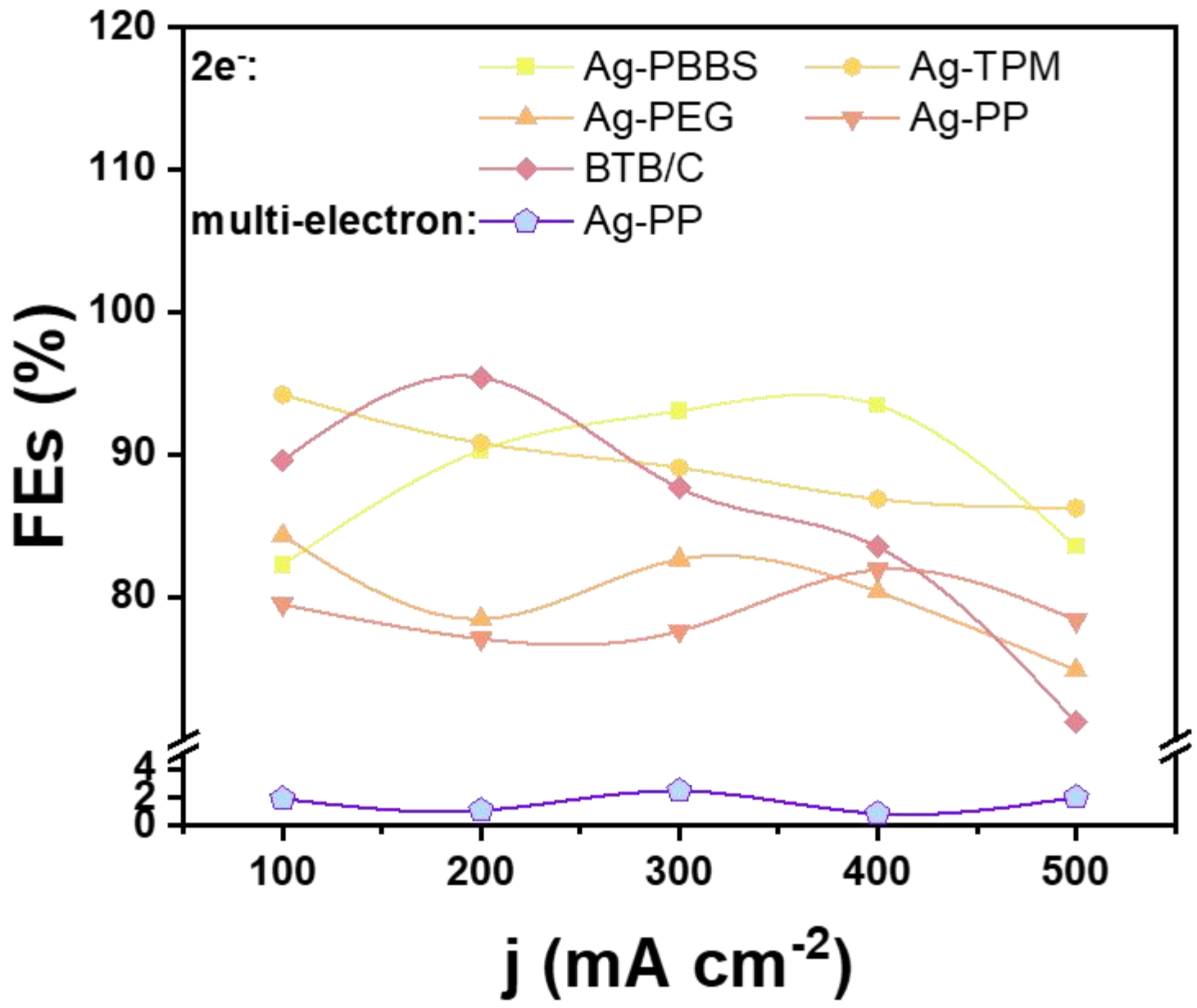

Figure S17. (a) Schematic illustration of different molecular structures. (b) Faradaic efficiencies of the corresponding molecules at current densities ranging from 100 to 500 mA cm^{-2} in a flow cell.

Upon discovering that BTB-modified Ag exhibited unexpectedly high CO_2RR performance, we systematically investigated whether the -OH group is solely responsible and whether simpler molecules can work, using a comprehensive set of control experiments (Supplementary Figure S17) with structurally related or simpler molecules. The results clearly demonstrated the specific necessity of the intact BTB structure: We tested phenyl 4-bromobenzenesulfonate and triphenylmethane (TPM), which share structural similarities with the BTB backbone but completely lack the critical -OH groups. As anticipated, Ag electrodes modified with these molecules yielded

almost zero multi-electron products, unambiguously confirming that the -OH group is the indispensable driver for C–C coupling. To test if any -OH containing molecule would suffice, we evaluated polyethylene glycol (PEG, an aliphatic polymer with terminal -OH) and simple phenolphthalein. PEG failed to produce any multi-electron products, while phenolphthalein generated only trace amounts. This indicates that a simple hydroxyl group is insufficient, and it may be situated within an appropriate structural framework to be effective. The corresponding data and discussion have been added into the revised Manuscript and the Supplementary Information.

Q2. How will loading different amounts of BTB affect the catalytic results? Please provide experimental evidence.

Response: We sincerely thank the reviewer for pointing out this crucial aspect. We completely agree that the loading amount of the molecular modifier is a key parameter that dictates the overall electrocatalytic performance. To address this, we have systematically investigated the effect of different BTB loading amounts on the CO_2RR activity and selectivity, and we have included these experimental data in the revised manuscript. As shown in the newly added Supplementary Figure S16 (the CO_2RR performance with different BTB loading amounts), the catalytic performance exhibits a typical volcano-shaped trend.

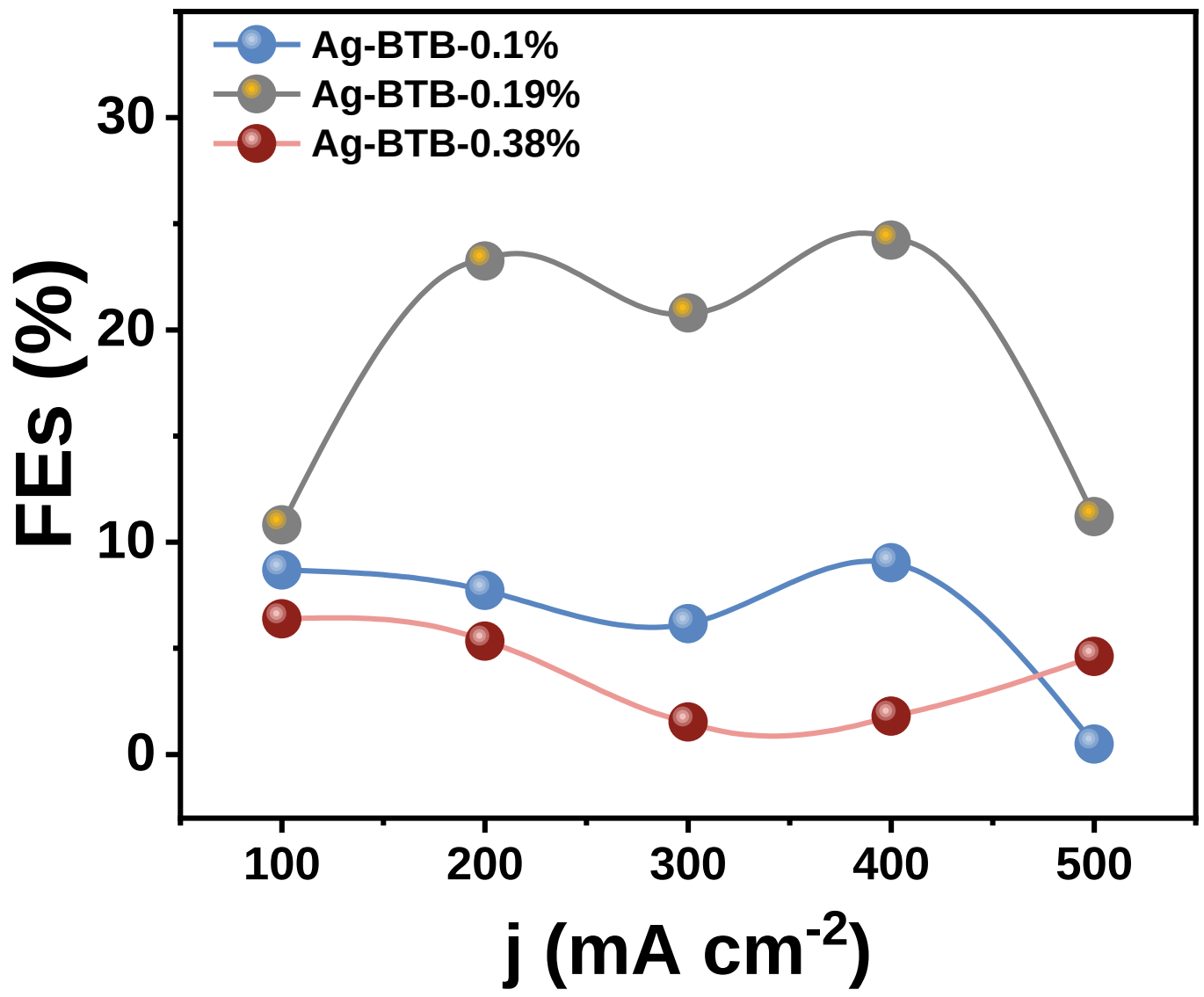

Figure S16. Comparison of Faradaic efficiencies for Ag modified with different contents of BTB.

Q3. One of the advantages of using non-Cu-based catalysts is the catalyst stability. The advantage of the Ag material also lies in its stability. Therefore, the authors should verify the stability of Ag-

BTB, especially the stability of BTB during the catalytic reaction. The infrared spectra after the reaction should be provided to explore whether there are any changes in BTB during the reaction. Please provide experimental data to show the changes in BTB during the reaction.

Response: We sincerely thank the reviewer for this insightful comment. We completely agree that verifying the stability of the Ag-BTB catalyst, especially the presence of the BTB (bromothymol blue) molecules at the interface during the CO_2 reduction reaction (CO_2RR), is crucial. To address this, we have performed post-electrolysis FT-IR and XPS characterizations to track the changes in the catalyst. As shown in the newly added Supplementary Figure S18 (FTIR spectra), the spectrum of the Ag-BTB catalyst after the stability test is almost identical to that of the pristine Ag-BTB. In the O 1s spectra, we observed that the peak intensity weakened after the durability test, demonstrating that the chemical environment of the oxygen atoms in the BTB molecules remains stable. For the Ag 3d spectra (Supplementary Figure S19-20), the peak intensity also weakened after the reaction, accompanied by a noticeable shift towards higher binding energy. This shift suggests a decrease in the electron density of Ag, which is highly reasonable in the context of CO_2RR. It reveals an electronic interaction and charge transfer between the Ag active sites, the BTB molecules, and the CO_2RR intermediates (e.g., *COOH or *CO) during the catalytic process. In summary, the combined FT-IR and XPS results prove that the BTB molecules maintain their structural integrity and stably exist at the Ag interface after long-term electrocatalysis. The corresponding data and discussion have been added to the revised Manuscript and the Supplementary Information.

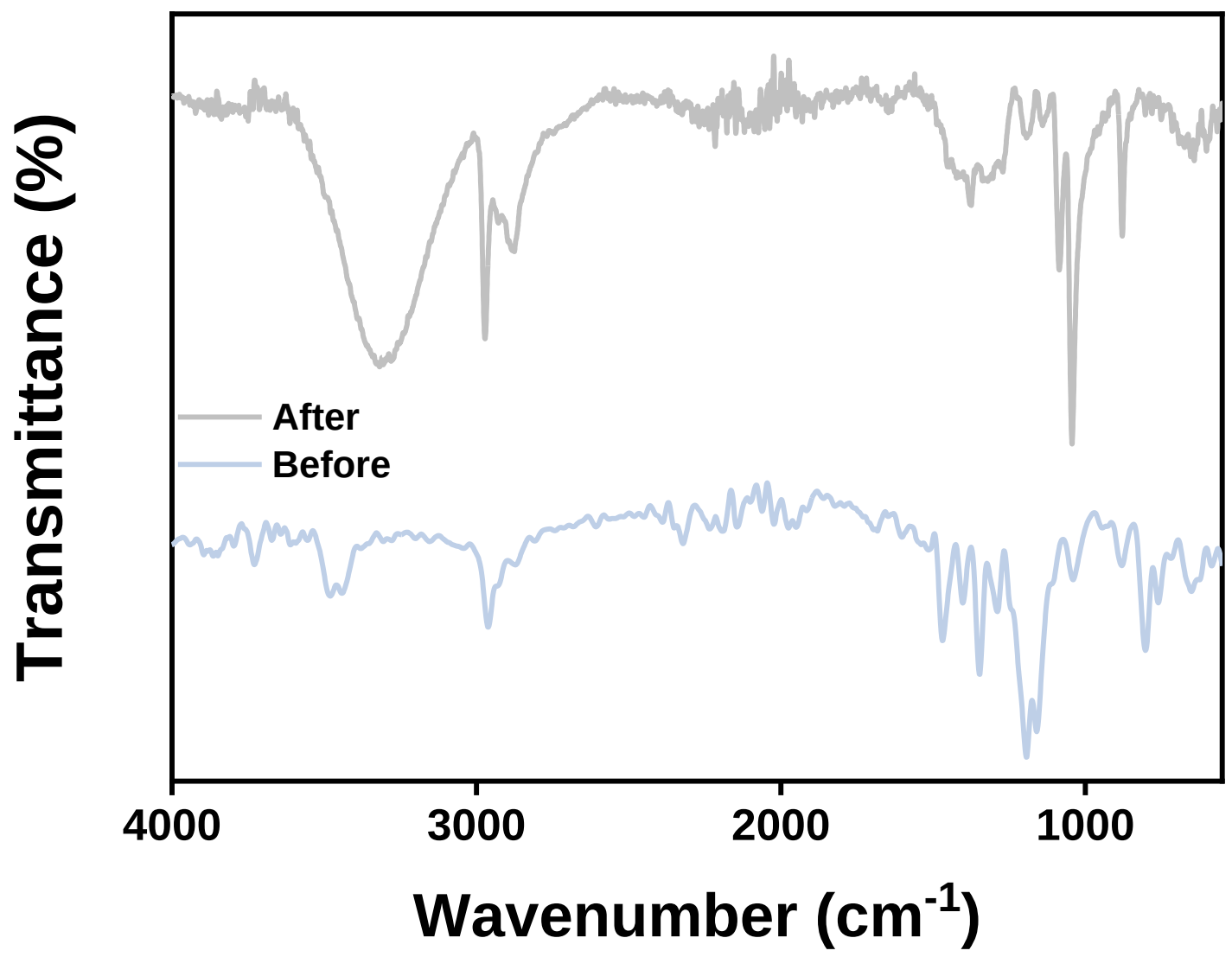

Figure S18. Post-stability FTIR characterization of Ag-BTB.

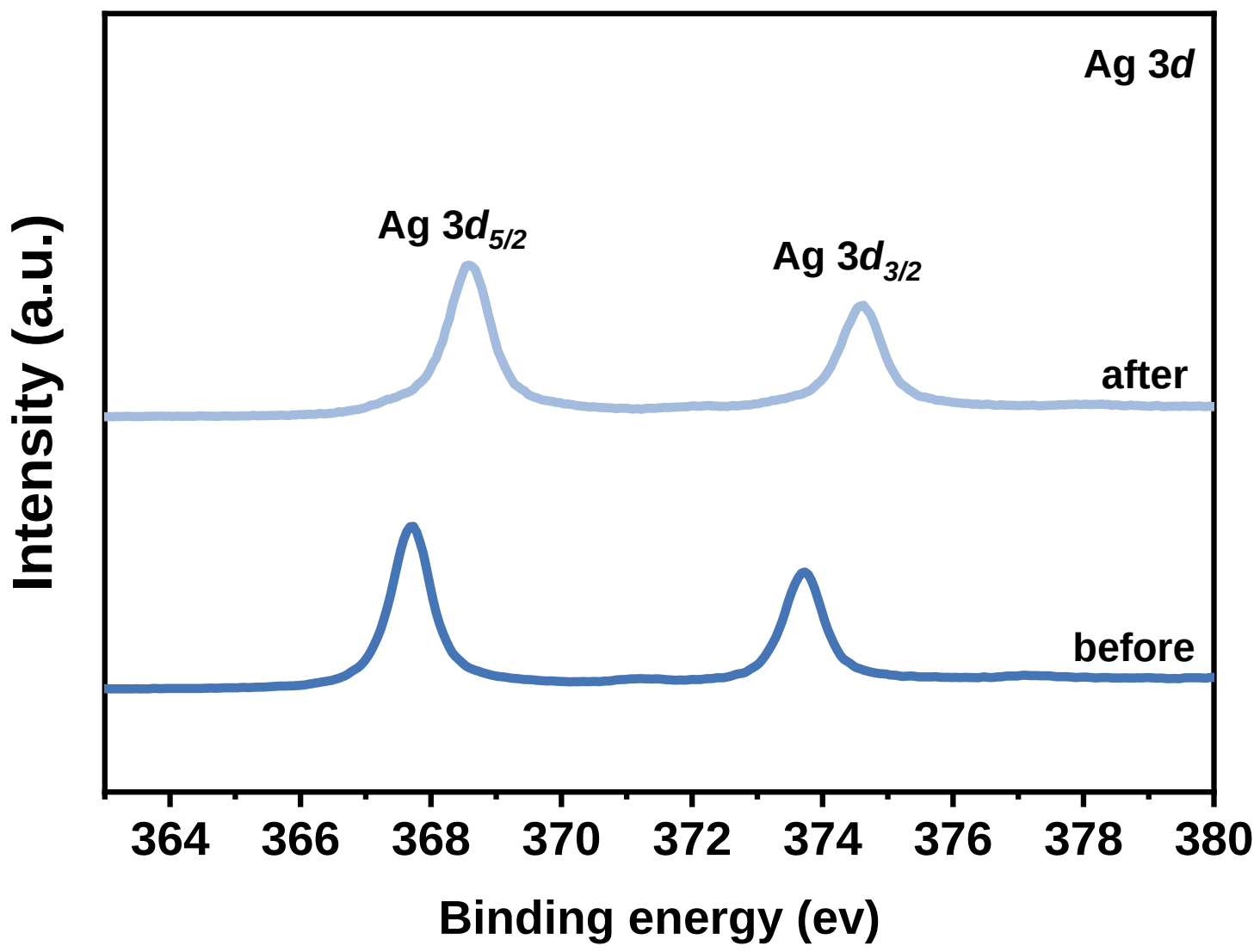

Figure 19. Post-stability XPS characterization of Ag 3d for Ag-BTB.

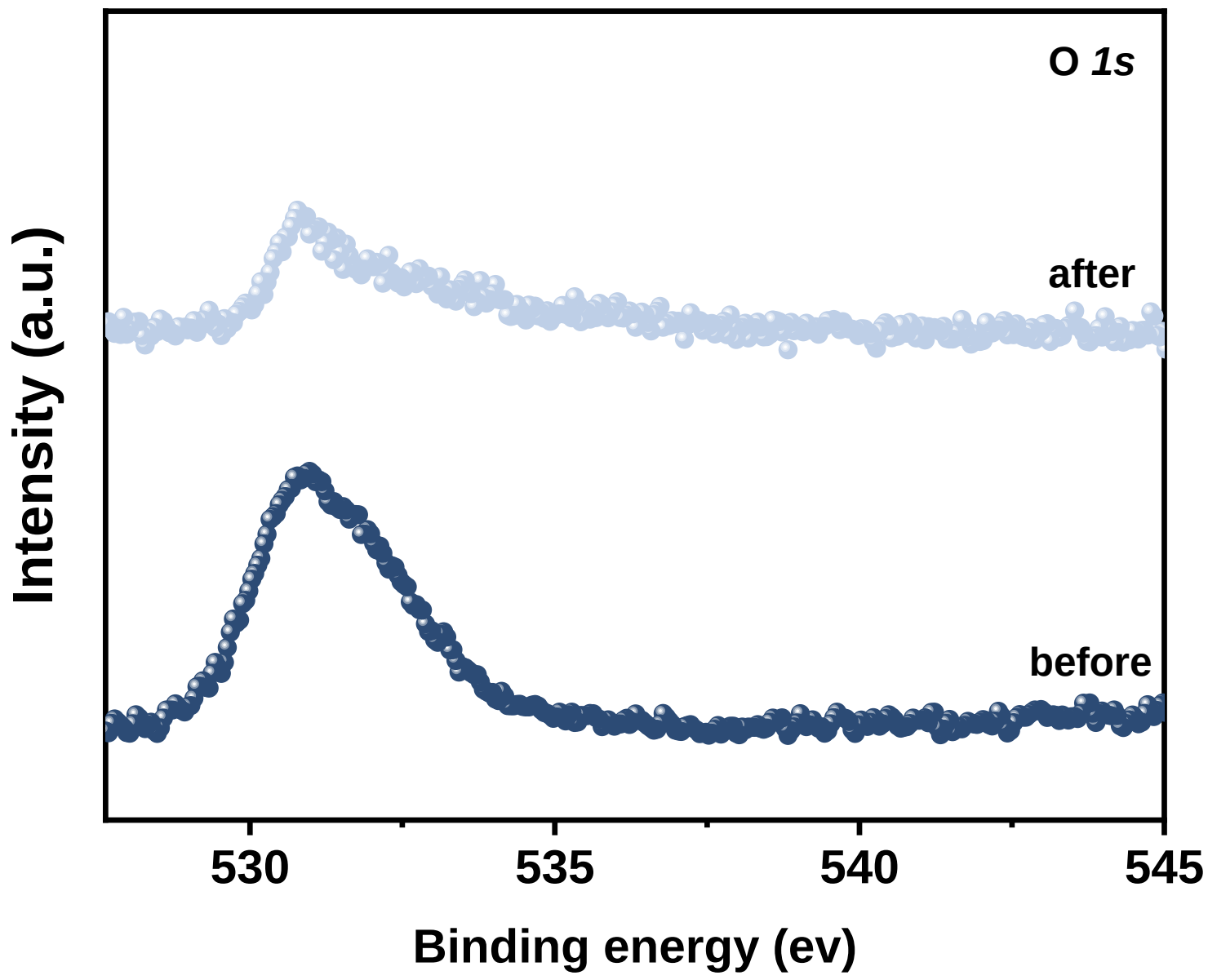

Figure S20. Post-stability XPS characterization of O 1s for Ag-BTB.

Q4. In the stability experiment in Fig. 2f, only the change in Faraday efficiency of the total multi - electron products were tracked. However, at least the changes in Faraday efficiency of ethanol and acetic acid should also be given.

Response: We sincerely thank the reviewer for this constructive and insightful suggestion. We highly agree that tracking the individual Faradaic efficiencies of specific multi-electron products is crucial for a comprehensive and rigorous evaluation of the catalytic stability. Following your valuable advice, we have updated the stability test results. In the revised Figure 2f, we have now explicitly included the continuous changes in the Faradaic efficiencies of both ethanol and acetic

acid throughout the entire stability experiment, in addition to the total multi-electron products. This content has been added into the revised Manuscript.

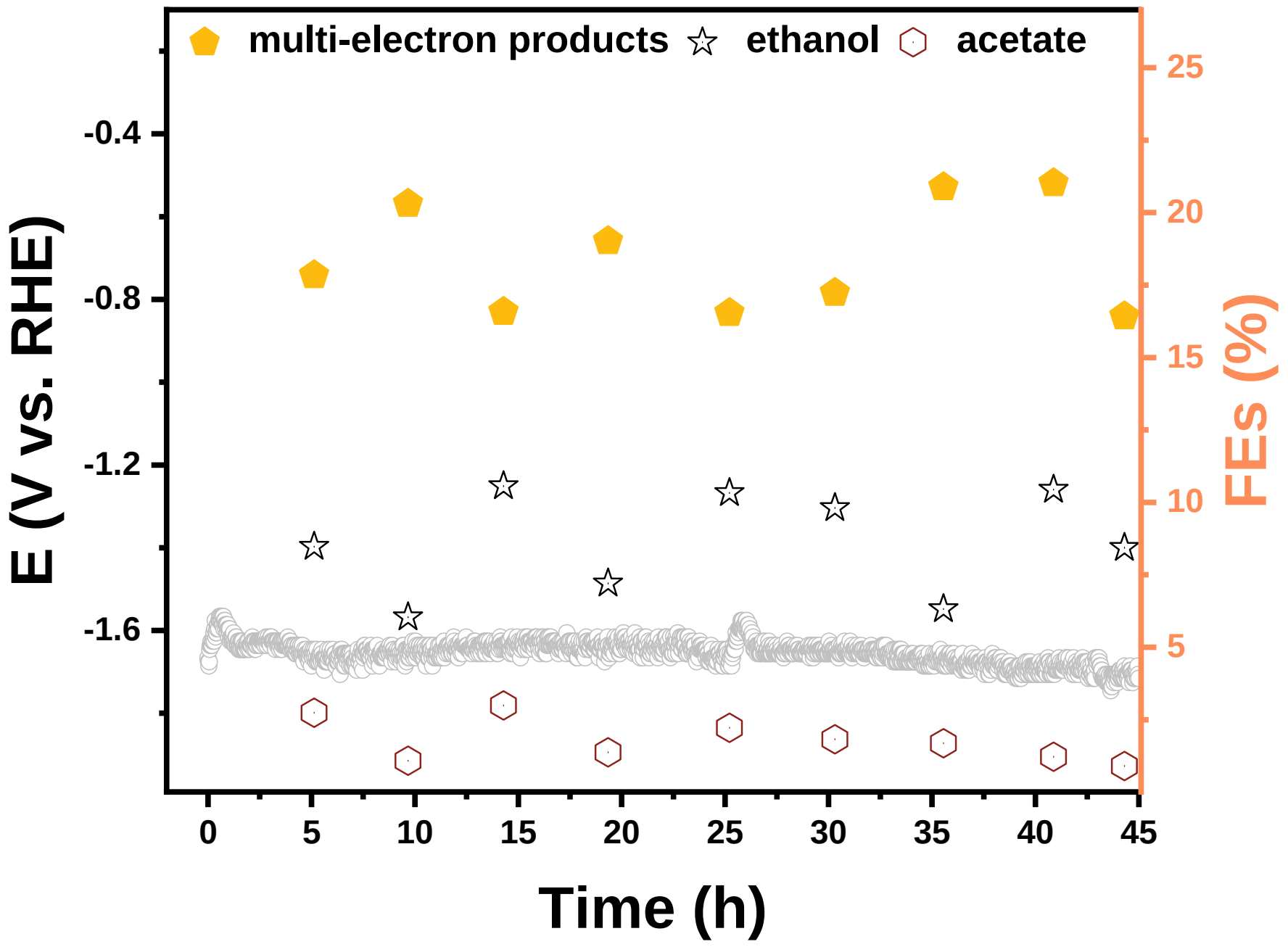

Figure 2f. Long-term CO_2RR performance on Ag-BTB at 300 mA cm^{-2} in a flow cell.

Q5. There is a lack of $^{13}CO_2$ experiments. (i) Please provide in situ differential electrochemical mass spectrometry during the experimental process, and (ii) conduct high-resolution mass spectrometry analysis of the all labeled products collected after the reaction.

Response: We sincerely thank the reviewer for this constructive and professional suggestion. We fully agree that ^{13}C isotopic labeling experiments are essential to provide unambiguous evidence that the detected products originate exclusively from the CO_2 feed. Following your suggestion, we have conducted rigorous ^{13}C labeling experiments. We have supplemented the manuscript with *in situ* differential electrochemical mass spectrometry (DEMS), ^{1}H and ^{13}C nuclear magnetic resonance (NMR) analysis to verify the carbon source for both gaseous and liquid products. Our detailed responses are as follows:(i) Regarding the *in situ* DEMS during the experimental process: We have successfully performed *in situ* DEMS using $^{13}CO_2$ as the reactant. As shown in the revised Supplementary Figure S21, the mass-to-charge ratio (m/z) signals clearly shifted to higher values, corresponding to the ^{13}C-labeled species: $^{13}CH_4$ was detected at m/z = 17 (compared to m/z = 15 for $^{12}CH_3^+$); ^{13}CO was detected at m/z = 29 (compared to m/z = 28 for ^{12}CO); $^{13}C_2H_4$ was detected at m/z = 30 (compared to m/z = 26 for $^{12}C_2H_2^+$);The reactant $^{13}CO_2$ was confirmed at m/z = 45. The

precise detection of these ^{13}C -labeled signals provides direct and real-time evidence that the gaseous products are indeed derived from the reduction of the CO_2 feed. (ii) Regarding the high-resolution mass spectrometry (HRMS) and liquid product validation: We acknowledge the reviewer's suggestion to use HRMS for higher precision. However, due to the current equipment limitations of our laboratory, we regret that we are unable to provide HRMS analysis for all labeled products at this time.

To ensure the scientific rigor of our conclusions, we instead employed ^{1}H and ^{13}C NMR spectroscopy (Supplementary Figure S22-23) to analyze the liquid products collected after the reaction. Notably, in the ^{1}H NMR spectra, the characteristic peaks of the liquid products evolved from standard singlets into distinct multiplets. This peak splitting is a direct consequence of the robust heteronuclear spin-spin coupling (*J*-coupling) between the $^{13}CO_2$ nuclei (spin $I = 1/2$) and adjacent protons (^{1}H), providing unequivocal evidence of ^{13}C incorporation into the molecular skeletons. This was definitively corroborated by the ^{13}C NMR spectra, which clearly resolved the characteristic chemical shifts of the ^{13}C-labeled oxygenates: bicarbonate at ~ 161 ppm; formate at ~171 ppm; acetate at ~181 ppm (carboxyl carbon) and ~23 ppm (methyl carbon); and ethanol at ~58 ppm (methylene carbon) and ~18 ppm (methyl carbon). Mechanistically, these isotopic labeling results provide rigorous analytical closure. They explicitly confirm that the highly valuable multi-electron products are synthesized de novo via the C–C coupling of CO_2-derived intermediates.

We believe that the combination of in situ DEMS (for gaseous products), ^{1}H and ^{13}C NMR (for liquid products) provides comprehensive and sufficient proof that all multi-electron products originate exclusively from CO_2 reduction.

The corresponding data and discussions have been added into the revised Manuscript and the Supplementary Information. We hope these additional results can address your concerns.

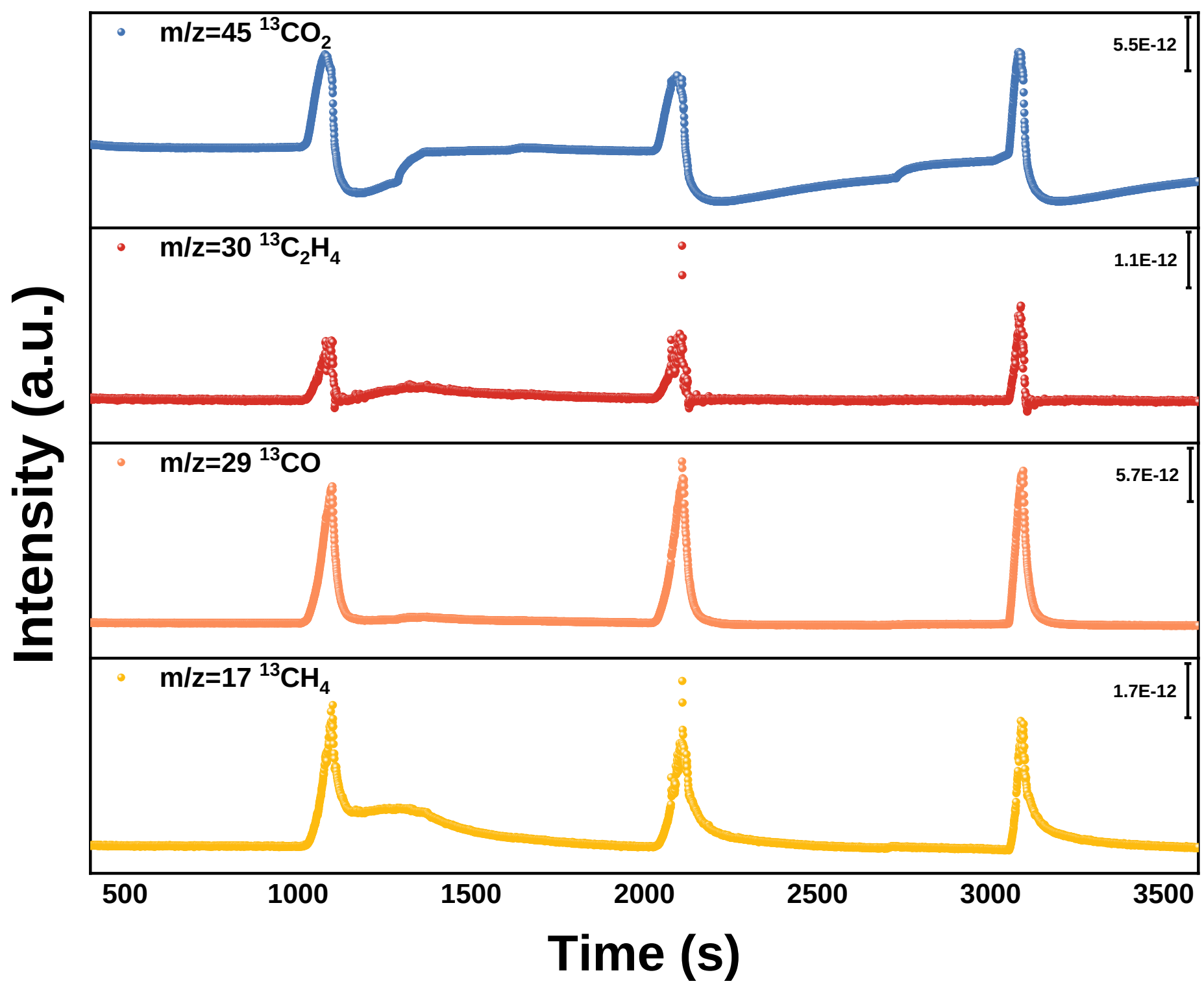

Figure S21. *In situ* Differential Electrochemical Mass Spectrometry (DEMS) measurements using $^{13}CO_2$ as the feed gas.

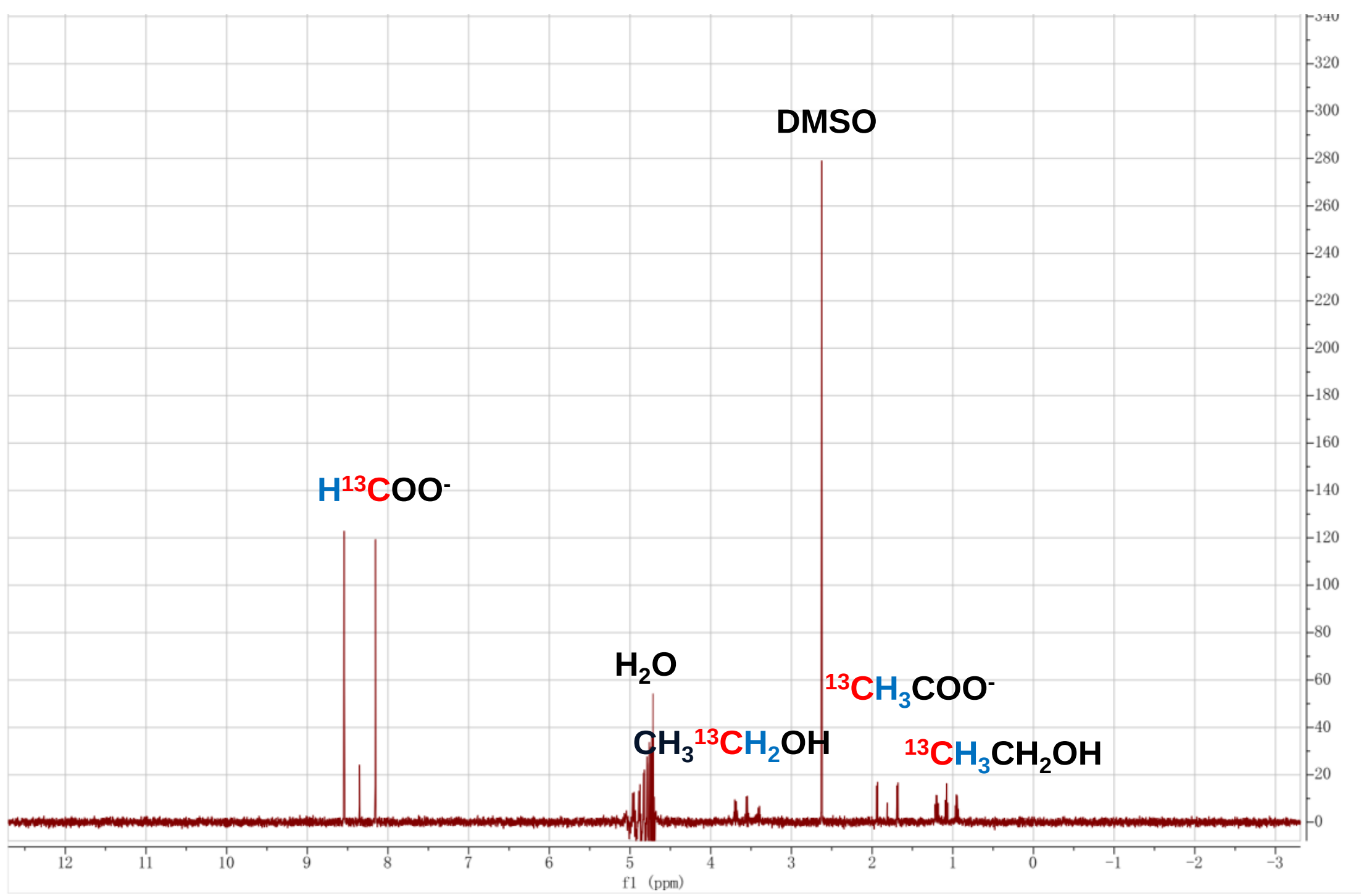

Figure S22. ^{1}H Nuclear Magnetic Resonance (NMR) spectrum of the liquid electrolyte after $^{13}CO_2$ electroreduction.

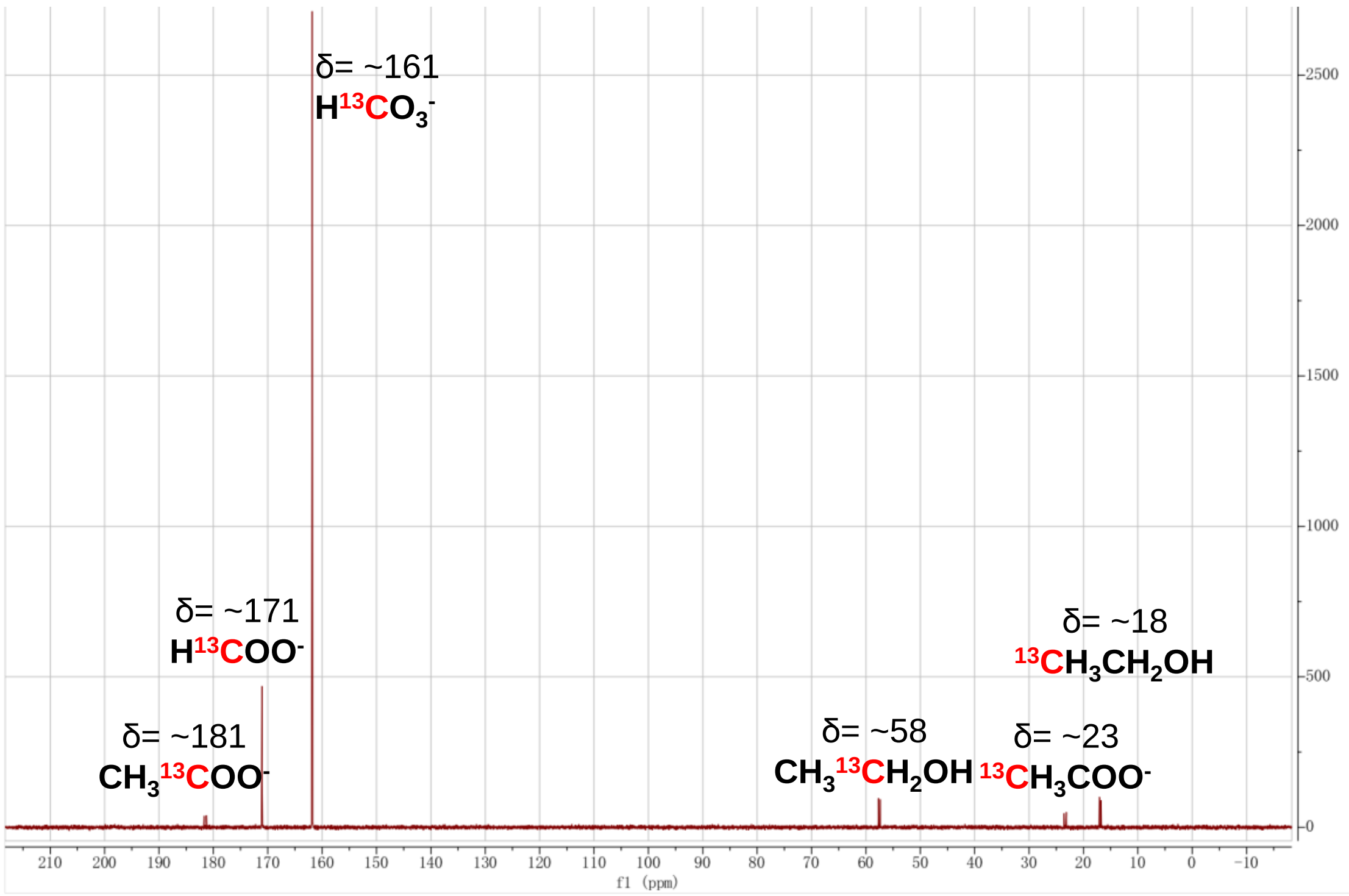

Figure S23. ^{13}C Nuclear Magnetic Resonance (NMR) spectrum of the liquid electrolyte after $^{13}CO_2$ electroreduction.

Q6. Please provide the GC spectra and NMR spectra of the catalytic products.

Response: We sincerely thank the reviewer for this important and rigorous suggestion. We highly agree that providing the gas chromatography (GC) and nuclear magnetic resonance (NMR) spectra is essential to demonstrate the reliability, and accuracy of our product analysis. Following your valuable advice, we have now provided the detailed GC and ^1H NMR spectra for both the pristine Ag and Ag-BTB catalysts (Figure R2-3). These spectra were systematically recorded during the electrocatalytic tests under various applied current densities ranging from 100 to 500 mA cm^{-2}. Furthermore, we have uploaded all the raw data as a separate file in this revised version.

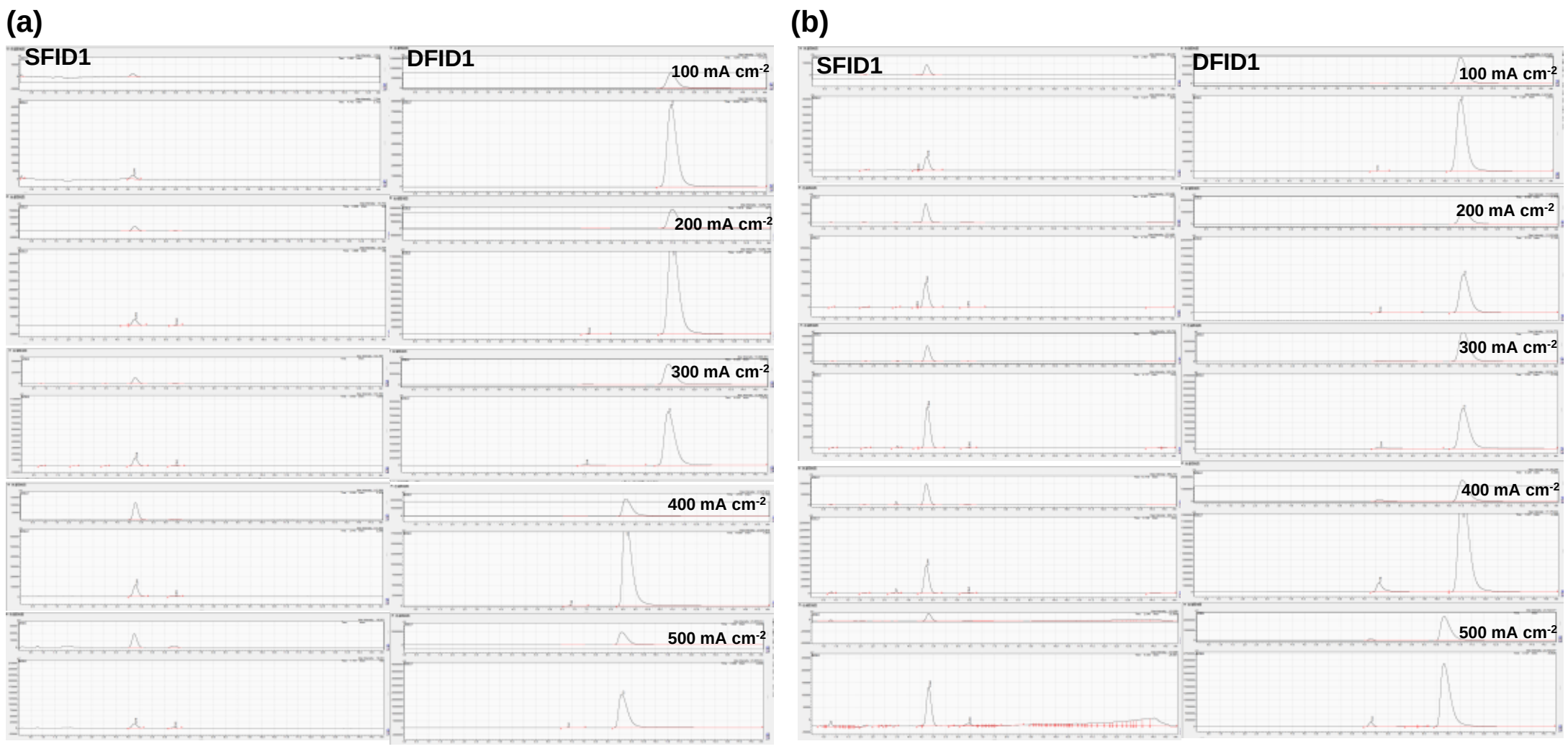

Figure R2. GC spectra of (a) Ag and (b) Ag-BTB

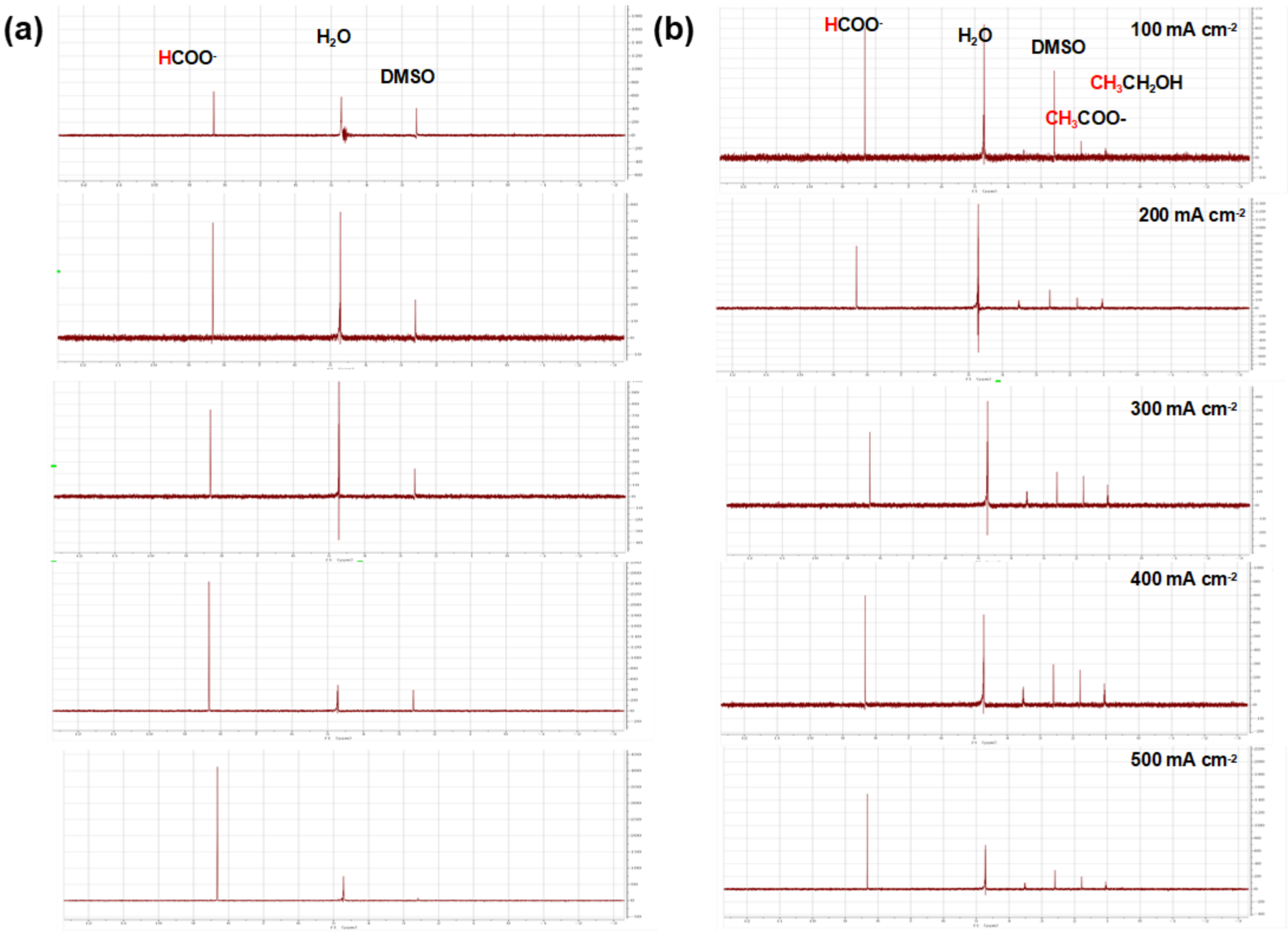

Figure R3. ^{1}HNMR spectra of (a) Ag and (b) Ag-BTB

Q7. here are numerous possibilities for C-C coupling, commonly including intermediates such as OCCO, OCCHO, and OCCOH. Can in situ infrared spectroscopy detect the aforementioned three intermediates? It is recommended to compare these three intermediates through computational analysis to elucidate their differences in the specific context discussed in this article.

Response: We sincerely thank the reviewer for this insightful and constructive comment. We completely agree that identifying the exact C-C coupling pathway is crucial, and comparing the

different possible intermediates computationally provides a rigorous mechanistic explanation.

Regarding experimental detection, *in situ* FTIR measurements successfully captured the characteristic vibrational absorption bands corresponding to the *COCOH intermediate. This provides direct and critical experimental evidence that the reaction in our Ag-BTB system preferentially proceeds via the *COCOH-mediated asymmetric coupling pathway, rather than the alternative *OCCO or *OCCHO pathways.

To elucidate why the *COCOH intermediate is experimentally favored in our specific context, we performed a comprehensive computational analysis comparing the thermodynamic free energies for forming the precursors of *OCCO, *OCCHO, and *OCCOH (Figure 5f-g). On the pristine Ag (111) surface, the free energy required to form the precursors for *COCOH (*CO + *COH) is 1.37 eV, which is already lower than that for *OCCHO (*CO + *CHO, 1.55 eV) and direct *OCCO formation (1.68 eV). Remarkably, upon introducing the BTB modifier (Ag-BTB), the thermodynamic energy barrier for the *CO + *COH pathway is further substantially reduced to 1.08 eV. This remains significantly more favorable than the competing *CO + *CHO pathway (1.24 eV) and the direct *COCO pathway (1.62 eV). This rigorous thermodynamic screening perfectly explains why the *COCOH intermediate is the dominant species observed in our FTIR spectra. Based on the established thermodynamic preference, we subsequently conducted a detailed transition state (TS) kinetic analysis specifically for this optimal *COCOH coupling pathway (Figure 5h).

For pure Ag (111), the kinetic activation barrier for the first critical proton-coupled electron transfer (PCET) step (TS1, forming *CO + *COH) is remarkably high at 1.89 eV. However, on the Ag-BTB surface drastically lowers this TS1 kinetic barrier to 1.43 eV. Furthermore, the subsequent C-C coupling step (TS2, *CO + *COH → *COCOH) features an extremely low barrier of only 0.23 eV (compared to 0.28 eV on pure Ag). In summary, the combination of in situ FTIR detection and our systematic DFT thermodynamic/kinetic comparisons provides a mutually verifying, fully closed-loop validation of the *COCOH pathway.

We have incorporated this comprehensive comparison into the revised Manuscript to address your valuable suggestion.

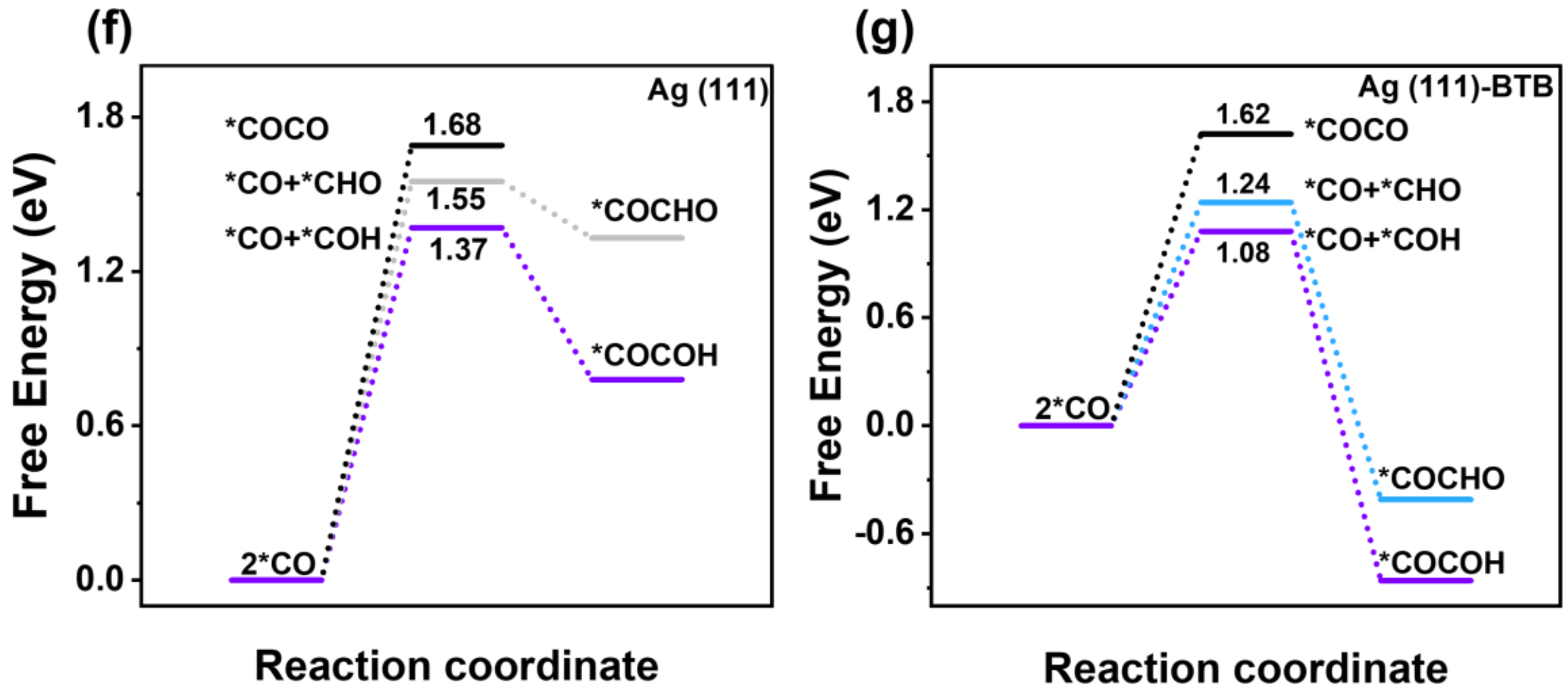

Figure 5f-g. Free energy profiles of the *CO hydrogenation and coupling steps on (f) Ag (111) and (g) Ag (111)-BTB.

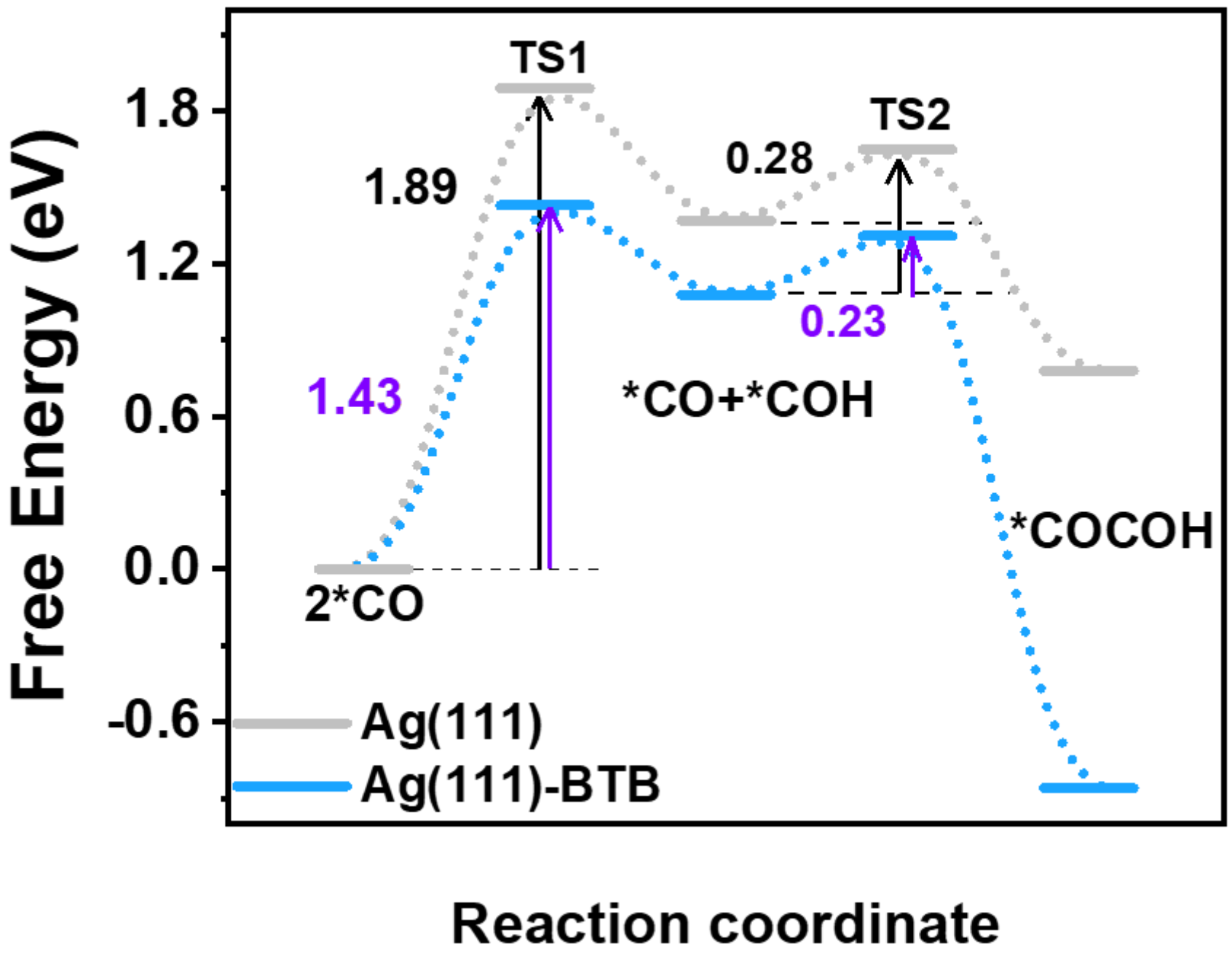

Figure 5h. Kinetic analysis of the transition states associated with *CO hydrogenation and C-C coupling.

Q8. What is the peak around 55°in the XRD? What substance does it belong to?

Response: We sincerely thank the reviewer for the careful observation and detailed question.

The diffraction peak located at around 55° in the X-ray diffraction (XRD) pattern originates from the carbon paper substrate that was used to support our electrocatalysts (Figure 1e). To avoid any confusion for future readers, we have explicitly marked this peak as the carbon paper in the revised XRD pattern. We have also added a clarifying statement in the corresponding main text to explain

the origin of this peak.

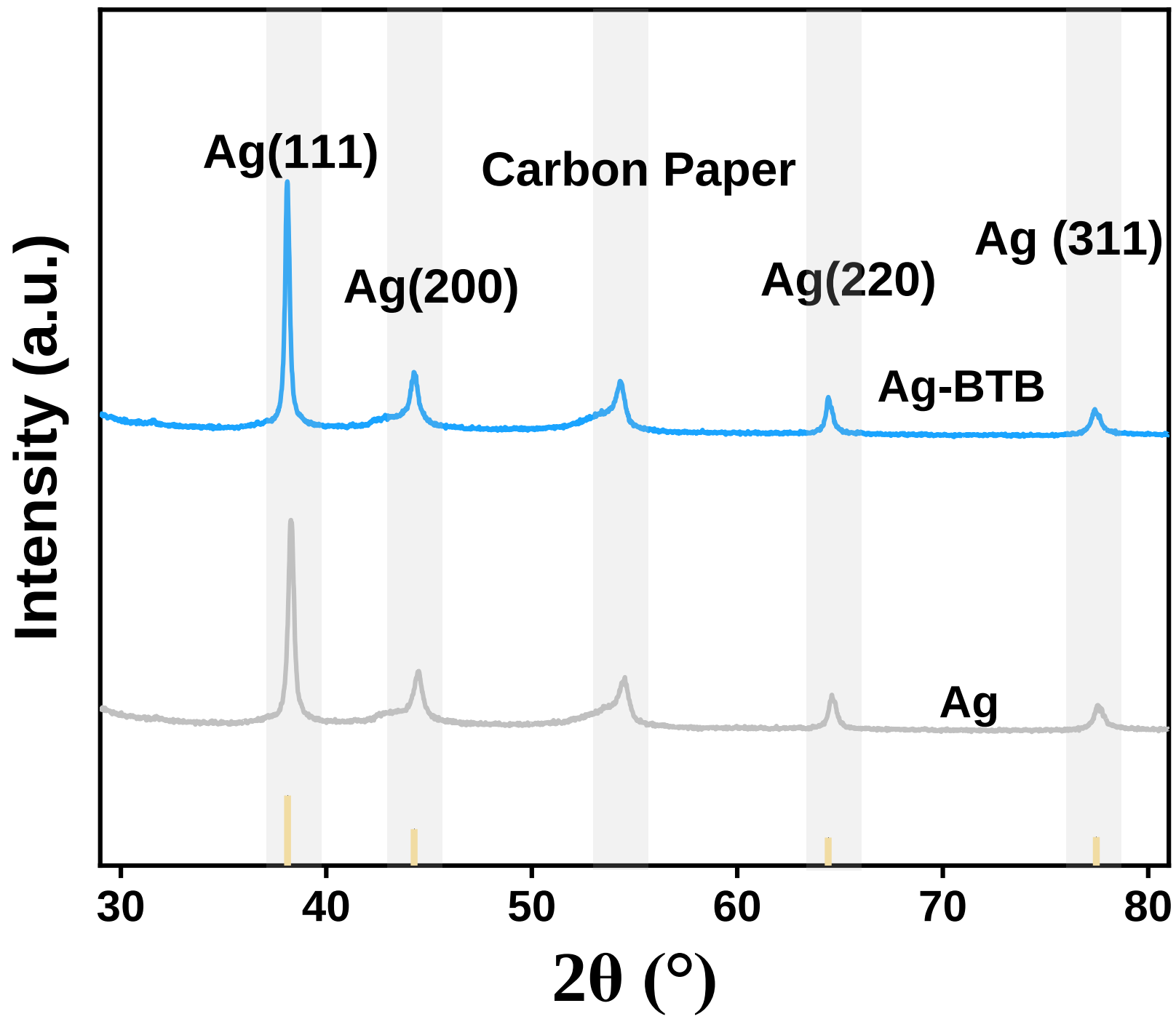

Figure 1e. XRD spectra of catalysts.

Q9. In Fig. 1g, is the peak of the Ag-BTB EXAFS at approximately R = 2 Å generated due to the bonding between Ag and BTB?

Response: We sincerely thank the reviewer for the meticulous observation and highly professional question. We apologize for not explicitly discussing this key feature in our previous manuscript, and we are grateful for the opportunity to clarify it. Yes, your understanding is entirely correct. The prominent peak located at approximately R = 2 Å (uncorrected distance) in the Fourier-transformed EXAFS spectra of Ag-BTB (Fig. 1g) is indeed attributed to the bonding between the Ag atoms and the BTB. To precisely identify this coordination, we conducted quantitative EXAFS fitting (summarized in Table 1). The fitting results reveal that this specific scattering path possesses a true bond length (R) of 2.18 Å with a coordination number (CN) of 0.5 ± 0.1. (Note: The difference between the apparent peak position at ~2.0 Å in the R-space plot and the fitted true bond length of 2.18 Å is caused by the typical photoelectron phase shift without phase-correction). This result exactly corresponds to the Ag-C coordination path, providing direct atomic-level evidence for the bonding between the Ag catalyst and the carbon atoms from the BTB molecules. We have explicitly labeled this peak as Ag-C in the updated Figure 1g and Table S1 to prevent any ambiguity. We

deeply appreciate your rigorous review, which has significantly enhanced the clarity and completeness of our structural analysis.

These results and discussions have been added to revised Manuscript and Supporting Information.

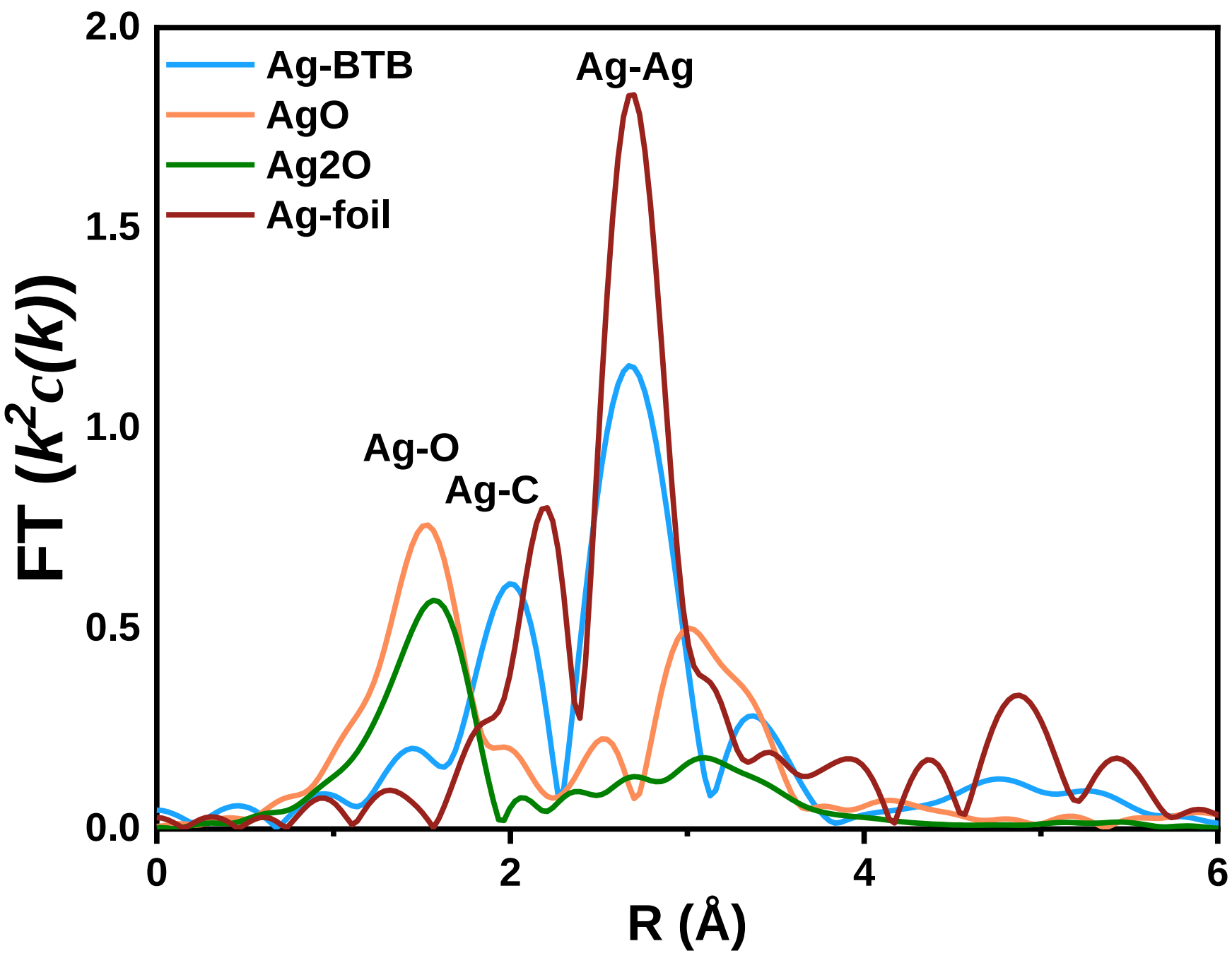

Figure 1g. FT-EXAFS spectra of Ag-BTB and the references.

Sample	bond type	CN*	R (Å)	σ² (10^{-3}Å²)**	R factor
	Ag-C	0.5±0.1	2.18±0.01	9.6±1.3	
Ag-BTB	Ag-O	2.2±0.2	2.30±0.02	8.8±2.1	0.021
	Ag-Ag	10.6±0.1	2.87±0.01	4.5±3.2	
Ag foil	Ag-Ag	12	2.89±0.02	6.4±3.4	0.007

* CN: coordination number; S_0^2 was fixed to be 0.82 from Ag foil.

** σ²: Debye−Waller factors

Table S1. EXAFS fitting parameters at the Ag K-edge of Ag-BTB.

Thank you again for reviewing this manuscript. These constructive suggestions have made a real improvement of our work. We truly appreciate your help.

Responses to Reviewer 3:

This manuscript investigates a molecular modification strategy using Bromothymol Blue (BTB) to facilitate multi-electron CO_2 reduction on silver catalysts. The authors propose that an interfacial hydrogen-bonding network stabilizes the *CO intermediate, enabling the formation of multi-electron products. The reported formation of multi-electron products on silver—a metal traditionally limited to C1 pathways—is a noteworthy observation. However, significant mechanistic and experimental deficiencies undermine the primary conclusions. Specifically, the proposed hydroxyl-mediated mechanism is chemically inconsistent with the strongly alkaline conditions. Therefore, the study does not meet the standards required for Nature Communications. Specific comments are listed below:

Response: We sincerely thank you for your thoughtful evaluation. We have carefully considered all the comments and have revised the manuscript accordingly.

Q1. The authors hypothesize that phenolic hydroxyl (-OH) groups stabilize the *CO intermediate via hydrogen bond. However, at the experimental pH of ≈ 14 (1 M KOH), BTB (pKa ≈ 7.1) exists predominantly in its deprotonated phenolate form ($-O^-$). This deprotonation precludes the molecule's function as a conventional hydrogen bond donor, rendering the proposed mechanism chemically inconsistent with the alkaline operating conditions.

Response: We sincerely thank the reviewer for this highly insightful and critical comment. From the perspective of macroscopic bulk-solution thermodynamics, the reviewer is absolutely correct that an ideal 1 M KOH solution (pH ≈ 14) would theoretically dictate the complete deprotonation of Bromothymol Blue (intrinsic bulk pKa ≈ 7.1).

However, we respectfully emphasize that the true chemical state of molecular modifiers at the highly heterogeneous solid-liquid interface under *operando* gas diffusion electrode (GDE) conditions deviates fundamentally from bulk behaviors. Our proposed mechanism, which stabilizes the *CO intermediate via the protonated -OH group, is chemically and thermodynamically valid. This survival of the -OH group is strictly guaranteed by the convergence of two interconnected physical chemistry phenomena: (1) severe *operando* local pH suppression and (2) a massive interfacial pKa shift driven by intense interfacial electrostatic fields. We clarify this non-ideal boundary condition

as follows:

To directly address the assumption of a pH 14 microenvironment, we must consider the classical Henderson-Hasselbalch equation (pH = pKa +log([CO_3^{2-}]/[HCO_3^-])). The pKa2 of carbonic acid is approximately 10.3. If the active interfacial pH remained at 14 as assumed for bulk 1 M KOH, the theoretical ratio of CO_3^{2-}/HCO_3^- would be roughly $10^{3.7}$ (over 4500:1). From a purely thermodynamic standpoint, this renders the existence of HCO_3^- practically impossible. However, it is a widely recognized consensus in the CO_2 reduction community that the generation and accumulation of HCO_3^- is ubiquitous in alkaline electrolytes. The very presence of this bicarbonate buffer network indicates that the actual catalytic interface is an extremely complex, non-uniform, and dynamic micro-domain that is heavily decoupled from the bulk alkalinity.

Operating a GDE flow cell at high current densities fundamentally reshapes the local mass-transport and acid-base equilibria compared to traditional H-cells (***Energy Environ. Sci.*** 2019, 12, 1442). Although the macroscopic electrolyte is 1 M KOH, the continuous, high-flux supply of CO_2 through the porous electrode triggers a rapid and intense neutralization reaction (CO_2+OH^-→HCO_3^-), which drastically depletes interfacial OH^-.

To quantitatively prove this, we conducted ^{13}C isotope-labeling NMR experiments (Supplementary Figure S23) to probe the *operando* interfacial speciation. In the rapid-exchange equilibrium between bicarbonate ($H^{13}CO_3^-$, ~160 ppm) and carbonate ($^{13}CO_3^{2-}$, ~168.5 ppm), the exact chemical shift dictates their relative ratio. Crucially, our *operando* extract revealed a prominent peak at exactly 161 ppm. Quantitatively, this proves that the majority of the labeled species exist as $H^{13}CO_3^-$. Based on the Henderson-Hasselbalch equation, the overwhelming dominance of bicarbonate provides ironclad proof that the massive CO_2 flux has completely buffered the local micro-domain pH down to a near-neutral/mildly alkaline range. As elucidated by Dunwell and Yan (***J. Am. Chem. Soc.*** 2017, 139, 3774), the abundant HCO_3^- at the interface acts not only as a pH buffer but also as a highly active, dynamic proton donor for interfacial reactions.

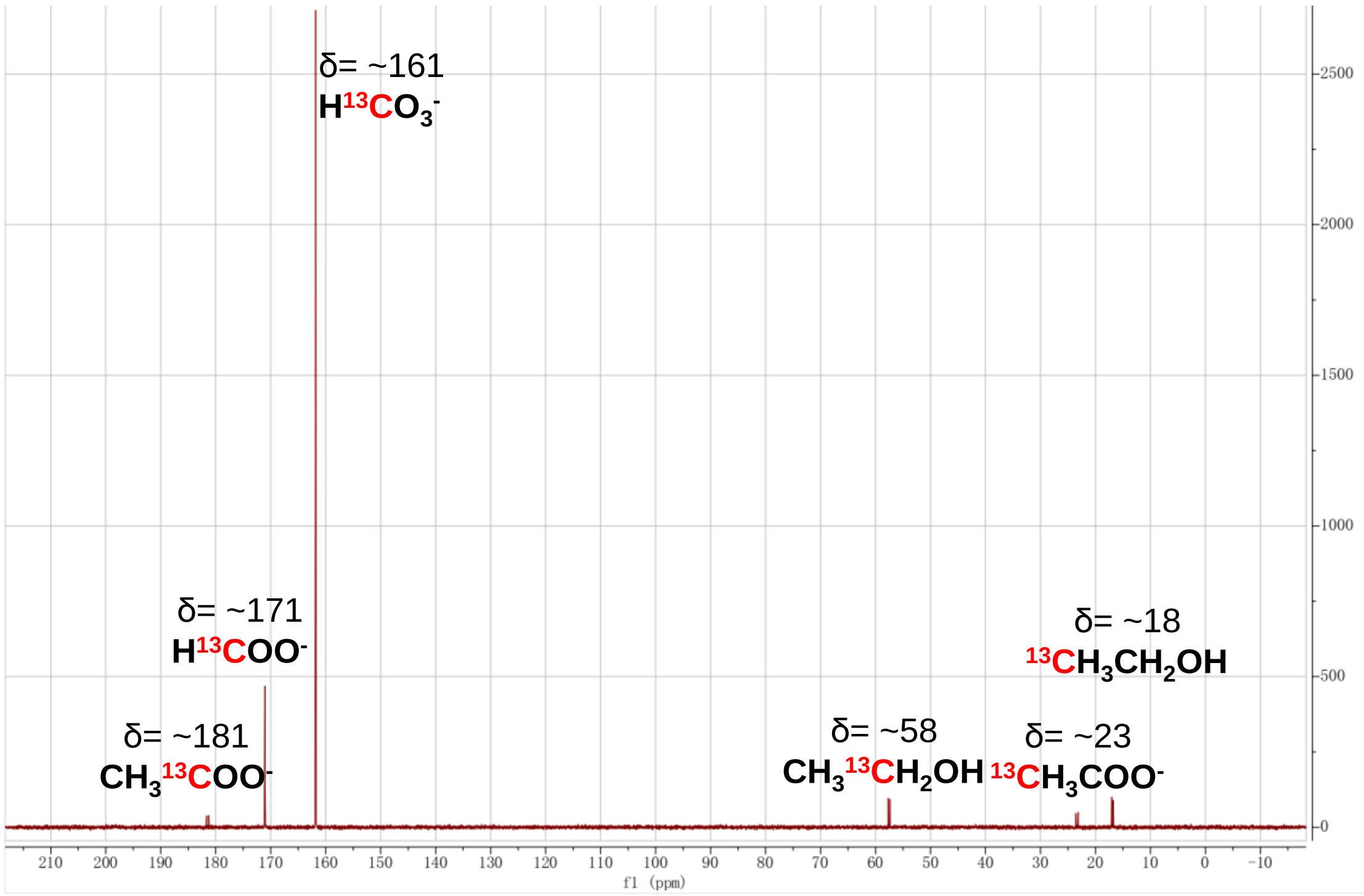

Figure S23. ^{13}C Nuclear Magnetic Resonance (NMR) spectrum of the liquid electrolyte after $^{13}CO_2$ electroreduction.

This profound acidification effect is rigorously supported by authoritative continuum modeling of porous electrodes by Bell and Weber (***Chem. Rev.*** 2022, 122, 11022). Furthermore, multiple independent *in situ* experiments confirm this exact phenomenon in 1 M KOH: direct Raman measurements by Zhuang et al. observed the local pH plummeting to ~7.2 (***J. Am. Chem. Soc.*** 2020, 142, 15438); a very recent *in situ* pH-probe study revealed an interfacial pH drop to ~8.8 at -1.2 V vs. RHE (***Natl. Sci. Rev.*** 2025, 12, nwaf173); and 2D direct imaging has visualized these low-pH micro-domains at the gas-liquid-solid boundary (***ACS Sustainable Chem. Eng.*** 2023, 11, 10300). While the local pH drops to the mildly alkaline range, preserving the -OH group requires the pKa of BTB to rise above its intrinsic bulk value (7.1). This is exactly what happens under the extreme physics of the *operando* electric double layer (EDL) and interfacial dielectric confinement. At operating cathodic current densities, the electrode surface is highly negatively polarized. If the BTB molecule were to exist as the deprotonated phenolate anion (-O$^-$), placing this negative charge near a highly negatively charged cathode would incur an overwhelmingly massive electrostatic repulsion penalty. To minimize the thermodynamic free energy of the interface, the extreme electric field forcibly shifts the apparent pKa upward. This exact physical mechanism was definitively proven *in*

situ via surface-enhanced infrared absorption spectroscopy (SEIRAS) by Delley, Mayer, et al. (***J. Am. Chem. Soc.*** 2021, 143, 10778). In this mechanism, applied negative potentials force the protonation of surface-bound organic species by drastically shifting their apparent pKa upward. Simultaneously driven by the locally suppressed pH (pillar 1) and the elevated apparent pKa (pillar 2), thermodynamics strongly favor the preservation of the BTB molecule predominantly in its neutral, protonated (-OH) state. The corresponding results and discussions have been added into the revised Supporting Information.

Q2. The comparative study between BTB and triphenylmethane (TPM) is insufficient to isolate the specific catalytic role of the hydroxyl groups. BTB possesses multiple functional moieties, including bromine atoms and a sulfonic acid group, which may significantly modulate the interfacial electronic properties and local hydrophilicity. The current experimental design fails to decouple these confounding factors. Furthermore, the absence of a broader evaluation involving other hydroxyl-containing molecules (e.g., phenolphthalein, phenol or other triarylmethane derivatives) limits the understanding of the structure-activity relationship and the generalizability of the proposed mechanism.

Response: We sincerely thank the reviewer for pointing this out. We agree that the initial comparison between BTB and TPM was insufficient to fully decouple the specific roles of the different functional groups (-OH, -Br, sulfonic acid) in the BTB molecule. To address your constructive comment, we conducted additional control experiments (Supplementary Figure S17) using several representative molecules to verify the necessity of BTB's specific structure. The results are summarized as follows: Confirming the necessity of the -OH group: We tested phenyl 4-bromobenzenesulfonate (PBBS) and triphenylmethane (TPM). Both molecules have similar aromatic backbones to BTB but lack the -OH group. As a result, both modified electrodes yielded almost zero multi-electron products. This confirms that the -OH group is essential for promoting the C-C coupling process. Evaluating simpler -OH containing molecules: We then tested if other common molecules with -OH groups could work. We chose polyethylene glycol (PEG) (aliphatic -OH) and phenolphthalein. The PEG-modified electrode produced no multi-electron products, while the phenolphthalein -modified electrode yielded only trace amounts. This indicates that simply having an -OH group is not enough; the larger structural framework of BTB is necessary to maintain

the effective catalytic environment.

These results and discussions have been added to revised Manuscript and Supporting Information.

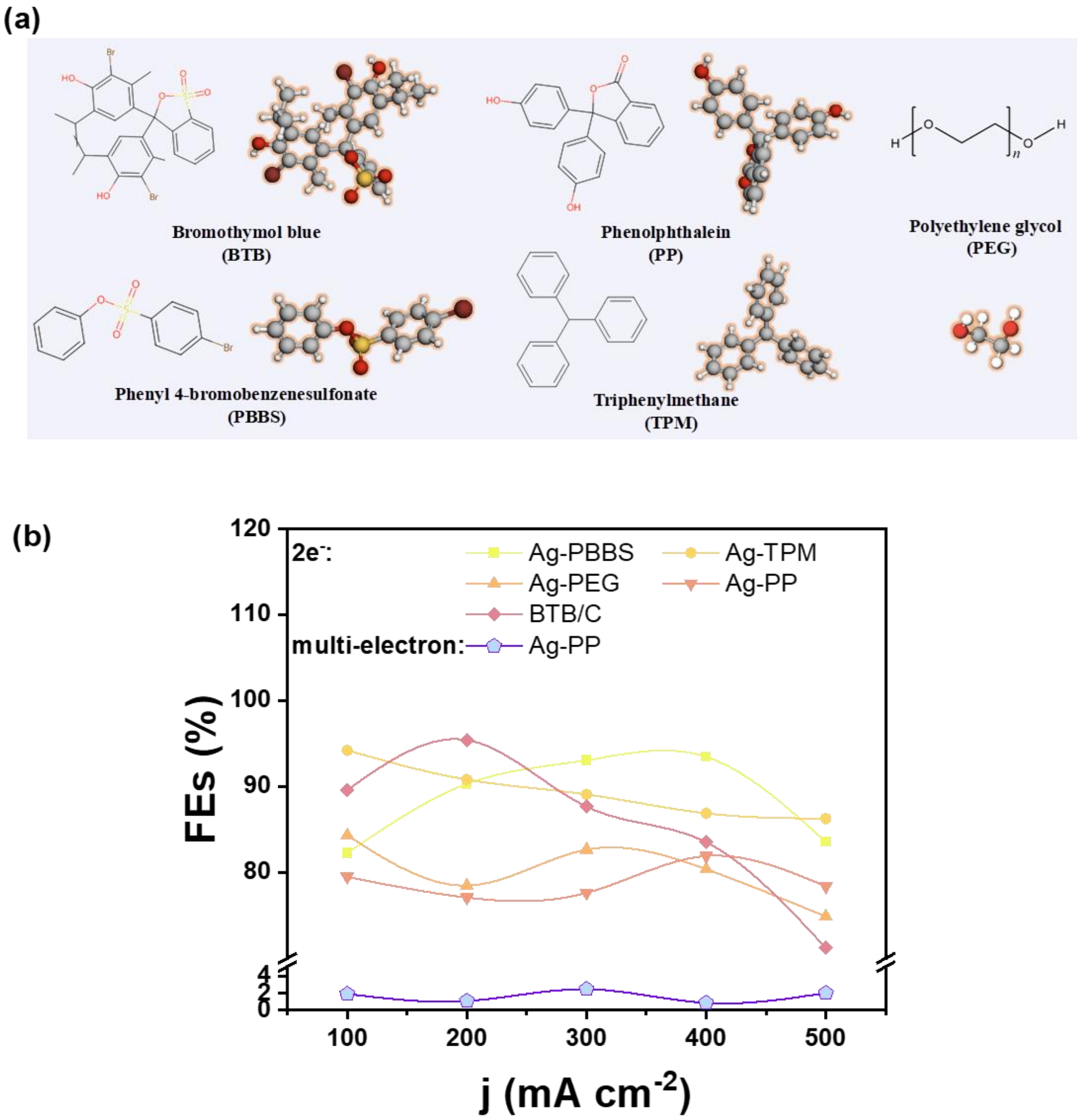

Figure S17. (a) Schematic illustration of different molecular structures. (b) Faradaic efficiencies of the corresponding molecules at current densities ranging from 100 to 500 mA cm^{-2} in a flow cell.

Q3. In the in-situ Raman spectroscopy measurements, the application of the water-structure deconvolution model from the Nature 2021 study (Ref. 41, https://doi.org/10.1038/s41586-021-04068-z) requires rigorous calibration for the specific interfacial environment. Unlike the SHINERS technique used in Ref. 41 which provides extreme surface sensitivity, the standard in-situ Raman used here may suffer from significant interference from bulk water, potentially skewing the calculated population of 2-HB and 4-HB water. The peak positions (e.g., ~3540 cm^{-1} for hydrated water) are sensitive to the cation type (K^+ vs. Na^+) and ionic strength. The authors must justify the peak assignments in 1 M KOH instead of simply adopting values from a $NaClO_4$ system in the

reference. Furthermore, the manuscript lacks a vibrational Stark slope analysis, which was a cornerstone in Ref. 41 to prove the ordered orientation of interfacial water under bias. Without this analysis, the claim that BTB promotes an "ordered water network" remains speculative.

Response: We sincerely thank the reviewer for this exceptionally insightful, rigorous, and highly professional critique. We fully agree that addressing potential bulk water interference, rigorously calibrating the peak assignments in 1.0 M KOH, and most importantly providing a quantitative vibrational Stark slope analysis are absolute prerequisites for validating our claim of an ordered water network. Following your invaluable guidance, we have recalibrated our model for the highly alkaline 1.0 M KOH environment and conducted a detailed Stark tuning rate and population analysis for both the pristine Ag and Ag-BTB electrodes. The newly extracted data perfectly align with the theoretical framework in Ref. 41 and provide unequivocal, quantitative proof of our hypothesis. Our detailed responses are as follows: We agree that directly adopting assignments from a $NaClO_4$ system is inappropriate due to the high ionic strength and cation hydration in 1.0 M KOH. We have rigorously recalibrated the O-H stretching envelope deconvolution, specifically accounting for the K^+-hydrated network. Gaussian deconvolution of this region reveals three distinct water configurations: four-coordinated hydrogen-bonded water (4-HB·H_2O, ~3200cm^{-1}), two-coordinated hydrogen-bonded water (2-HB·H2O, ~3450 cm^{-1}), and K^+-hydrated water (K^+-H_2O, ~3650 cm^{-1}). The detailed justification for these peak assignments in 1.0 M KOH. To definitively address the concern regarding bulk water interference and to prove the ordered nature of the interfacial water, we plotted the Stark tuning diagrams (Figure 4a-d). The comparison of the Stark slopes between the two electrodes reveals a profound mechanistic contrast.

For pristine Ag electrode, the Stark slopes for the K^+-hydrated water, 2-HB water, and 4-HB water are 17.18, 14.81, and 13.8 cm^{-1} V^{-1}, respectively. These moderate tuning rates suggest that the observed water molecules experience a relatively weak average local electric field, indicating that the signal on pristine Ag may indeed contain contributions from the less-polarized diffuse layer or bulk water. As contrast, the Stark slopes for the corresponding peaks on the Ag-BTB electrode exhibit a massive increase, reaching 34.07, 43.92, and an astonishing 51.11 cm^{-1} V^{-1}, respectively. According to the physical principles established in Ref. 41, the vibrational Stark tuning rate is directly proportional to the local interfacial electric field strength.

We have incorporated these decisive Stark slope diagrams, the population analysis (Supplementary

Figure S25), and this data-driven discussion into the revised main text. We are deeply grateful to the reviewer; this rigorous analysis has transformed a previously qualitative claim into a solid, quantitatively proven mechanism.

The relevant spectra and discussions have been added to the revised Manuscript.

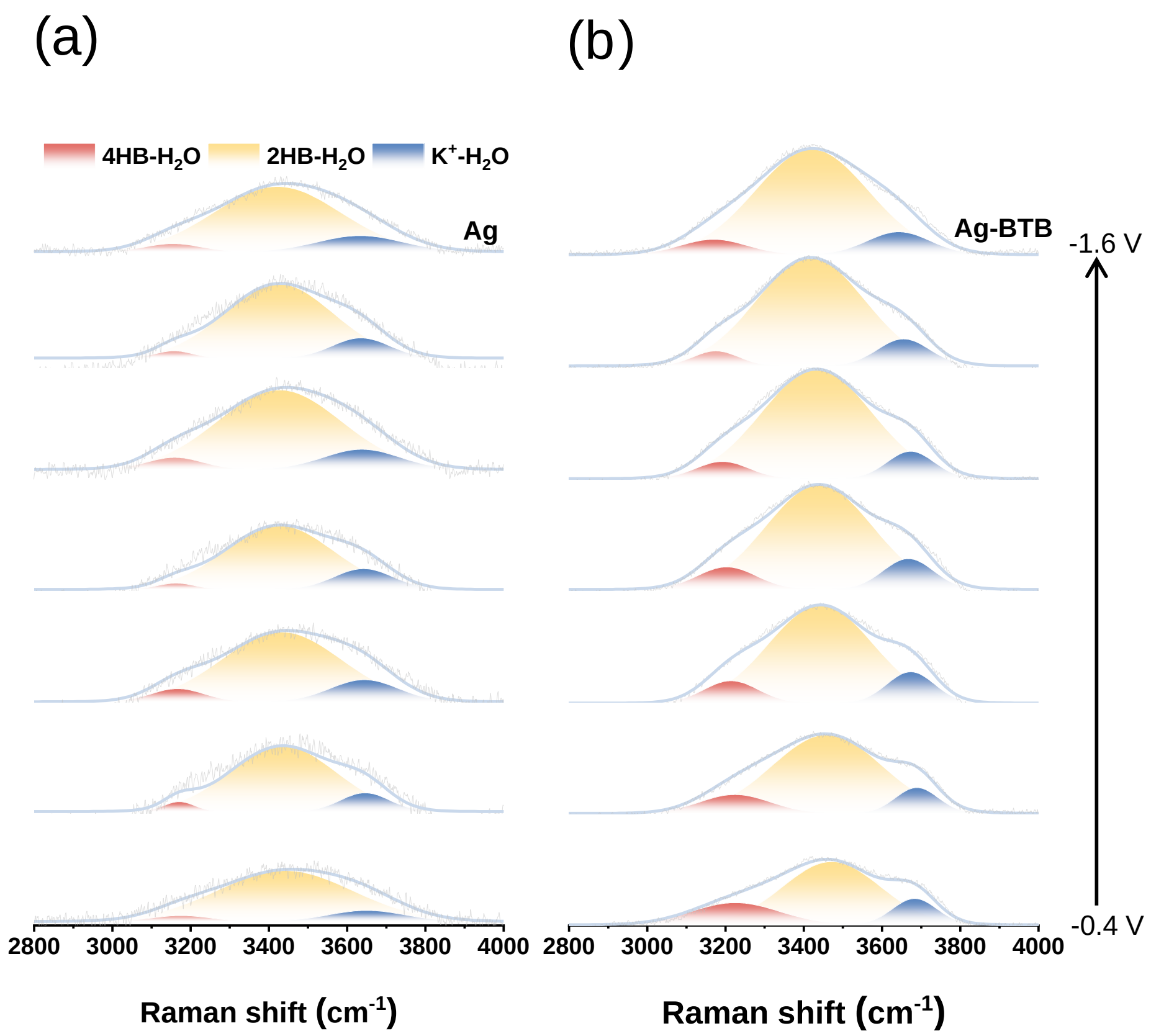

Figure 4a-b. Peak fitting of the water peaks for (a) Ag and (b) Ag-BTB at various potentials.

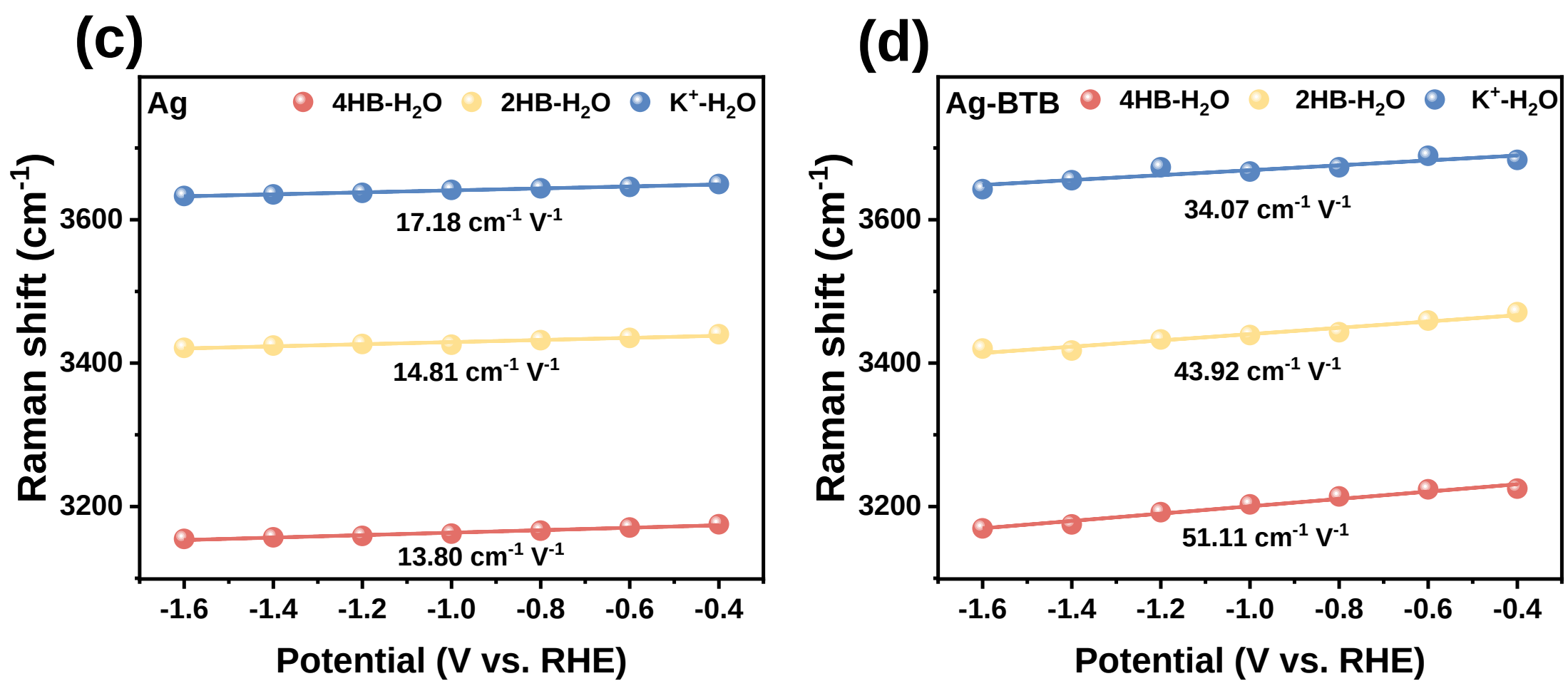

Figure 4c-d. Peak fitting of the water peaks at various potentials, corresponding stark slope analysis for (c) Ag and (d) Ag-BTB.

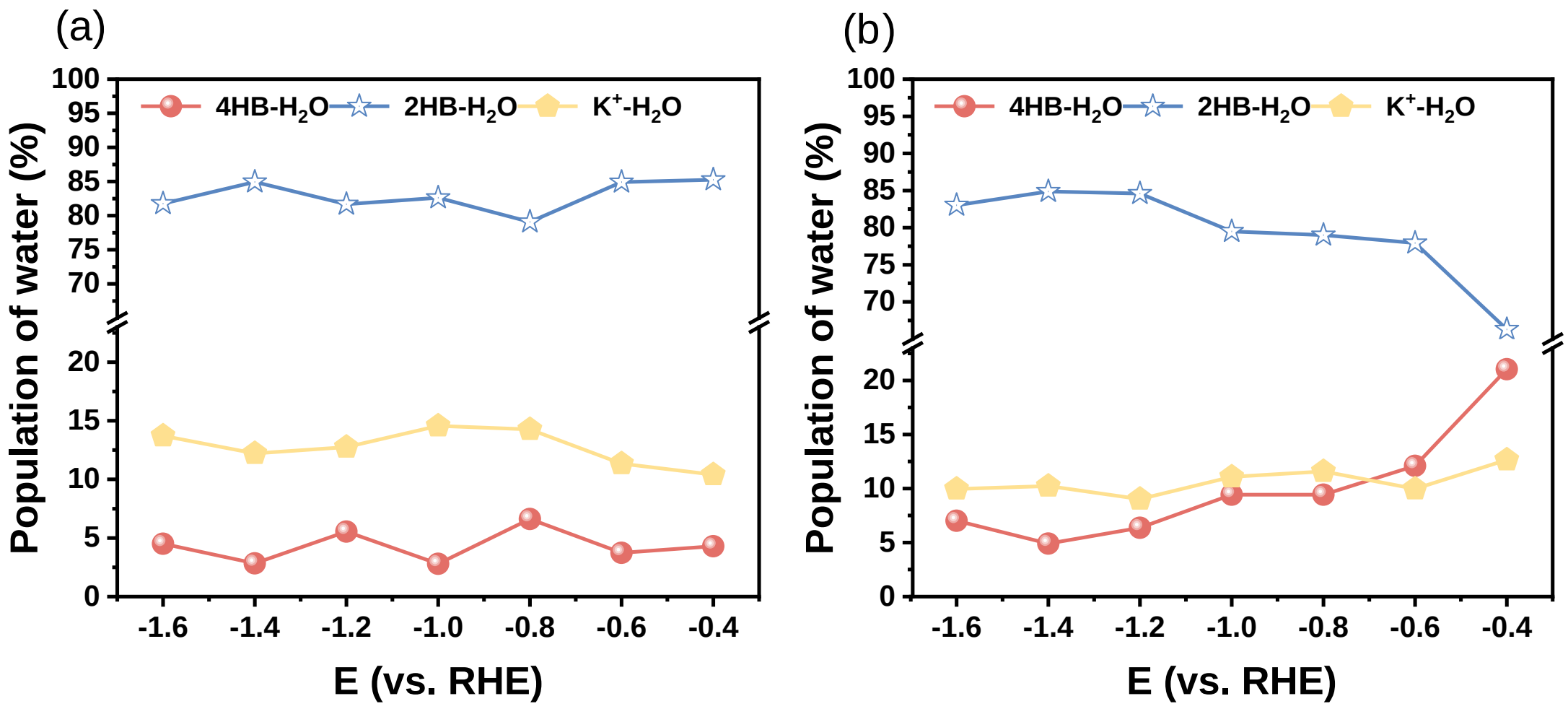

Figure S25. Population of water on Ag (a) and Ag-BTB (b) derived from in situ Raman peak fitting.

Q4. The DFT analysis is restricted to thermodynamic descriptors such as adsorption energies and electronic structure, without evaluating any kinetic barriers for key elementary steps (e.g., C–C coupling or CO hydrogenation). Therefore, the calculations do not provide sufficient mechanistic insight into how BTB promotes the formation of multi-electron products. While the paper emphasizes the role of 4-coordinated hydrogen-bonded (4-HB) water, this species is completely absent from the DFT models. Most critically, the simulations employ a BTB molecule in neutral environment, failing to account for its deprotonated state in the strongly alkaline electrolyte.

Response: We sincerely thank the reviewer for these highly constructive and insightful comments. We completely agree that incorporating kinetic evaluations and considering the interfacial aqueous/electrolyte environment are essential for providing a comprehensive mechanistic understanding. In response to your valuable suggestions, we have performed extensive additional calculations and updated our models. To gain deeper mechanistic insights into the formation of multi-electron products, we have now evaluated the kinetic activation barriers for the key elementary steps along the thermodynamically favored *COCOH pathway (Figure 5f-h). The transition state (TS) analysis indicates that the BTB modification effectively lowers the kinetic barrier for the critical initial CO hydrogenation step (TS1, forming *COH) from 1.89 eV on pristine Ag to 1.43 eV on Ag-BTB. Furthermore, the subsequent C-C coupling step (TS2, *CO + *COH → *COCOH) features a highly accessible barrier of 0.23 eV. These kinetic results provide strong theoretical support for how BTB promotes the multi-electron products pathway. We acknowledge the reviewer's point regarding the absence of water molecules in our previous simplified model. To

better reflect the realistic electrochemical interface and the alkaline electrolyte conditions, we reconstructed our DFT model by explicitly introducing interfacial H_2O molecules and KOH species.

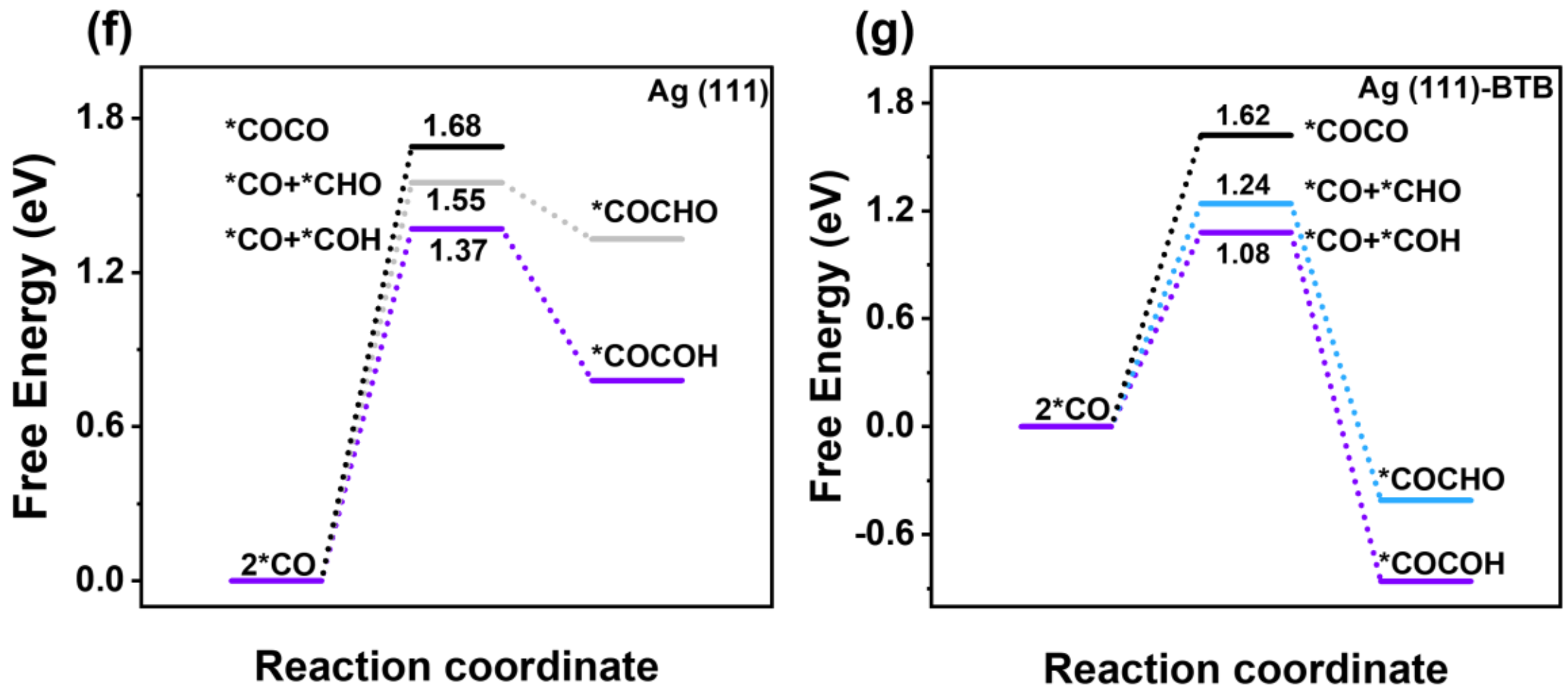

Figure 5f-g. Free energy profiles of the *CO hydrogenation and coupling steps on (f) Ag (111) and (g) Ag (111)-BTB.

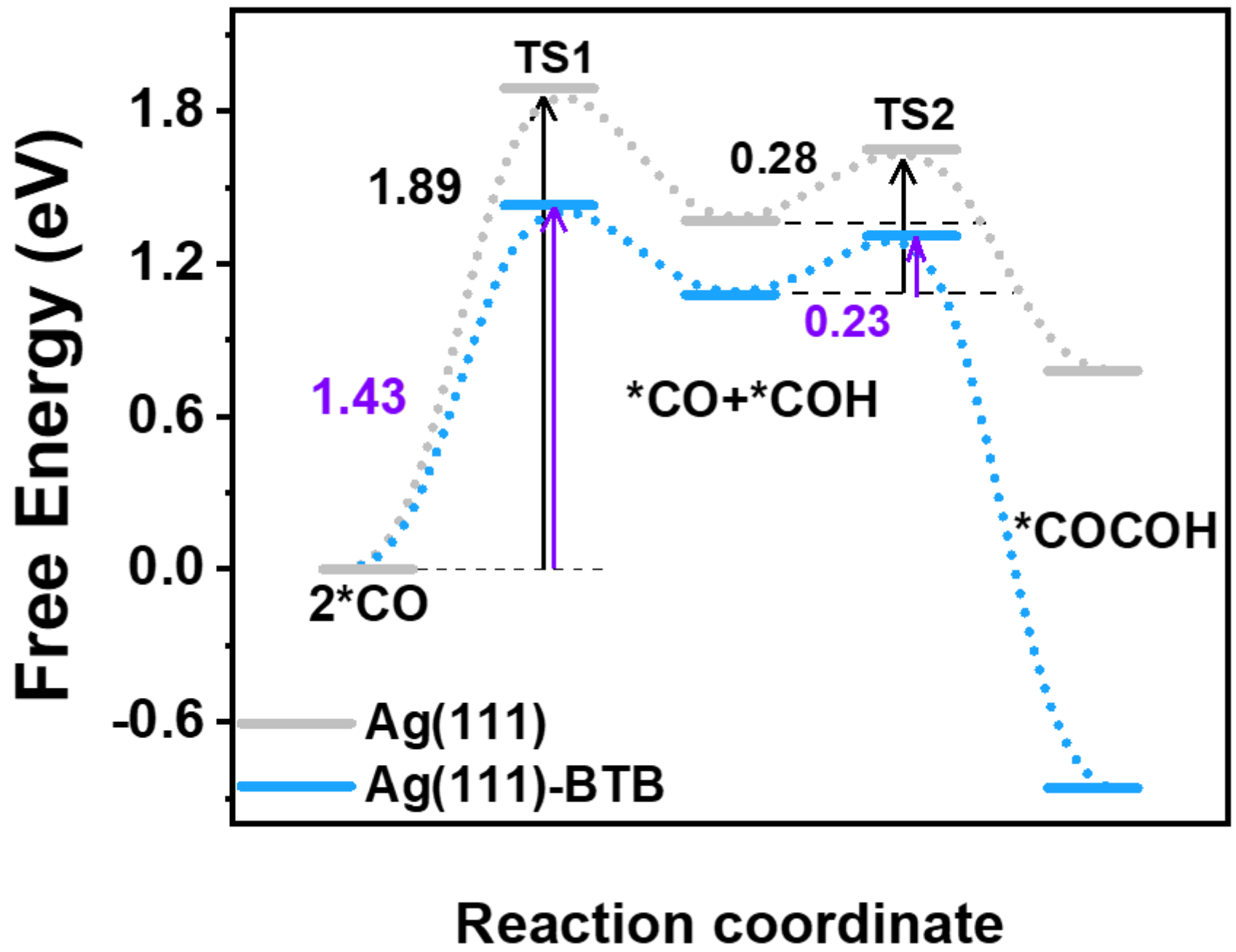

Figure 5h. Kinetic analysis of the transition states associated with *CO hydrogenation and C-C coupling.

Under these explicitly solvated conditions, we recalculated the adsorption energy E_{ads}. As shown in Figure 5a, the E_{ads} of *CO on the solvated Ag-BTB interface is -0.51 eV, which remains significantly more negative than that on the pristine Ag surface (-0.17 eV). This result indicates that the BTB-induced stabilization of the *CO intermediate remains highly effective, even when

accounting for the competitive solvation environment. To further investigate whether the crucial hydrogen-bonding network can survive in this complex aqueous environment, we performed an independent gradient model based on Hirshfeld partition (IGMH) analysis on the solvated model. As depicted in Figure 5d, the scatter plot of $d\delta_g$ versus Sign $(\lambda_2)\rho$ displays a distinct distribution peak in the negative region, which is a characteristic indicator of strong hydrogen-bonding interactions. This suggests that the H-bond network between *CO, the explicit water molecules, and the BTB modifier can be effectively maintained at the interface. We are deeply grateful to the reviewer for prompting us to consider these realistic conditions, which has significantly enhanced the mechanistic depth and reliability of our study.

The relevant calculations and discussions have been added into the revised Manuscript.

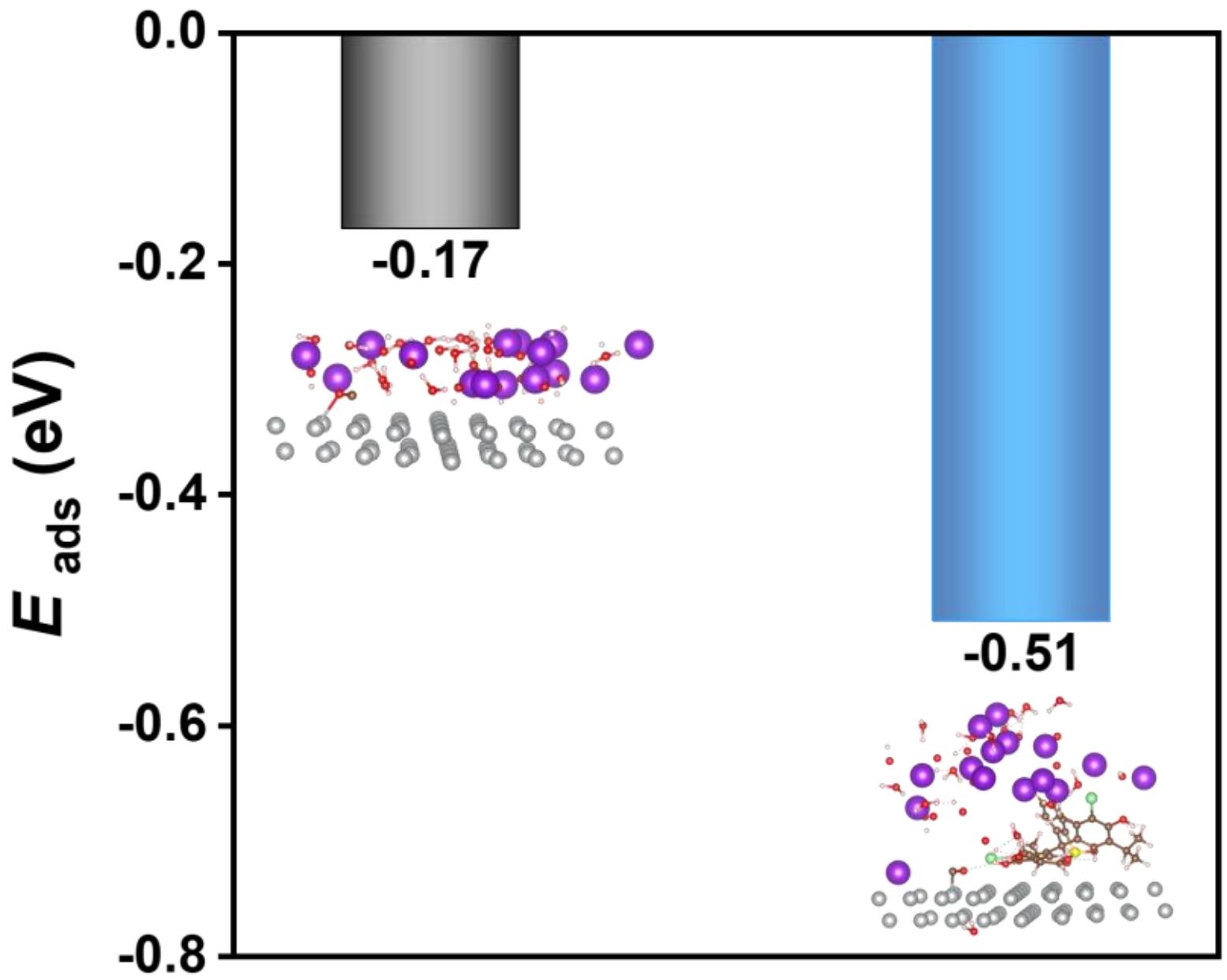

Figure 5a. The *E*ads of *CO on the electrode surfaces; (b) COHP curves for the Ag-C bonds in *CO on electrode surfaces. Light gray, brown, red, yellow, green, light pink, and purple balls correspond to Ag, C, O, S, Br, H, and K atoms, respectively.

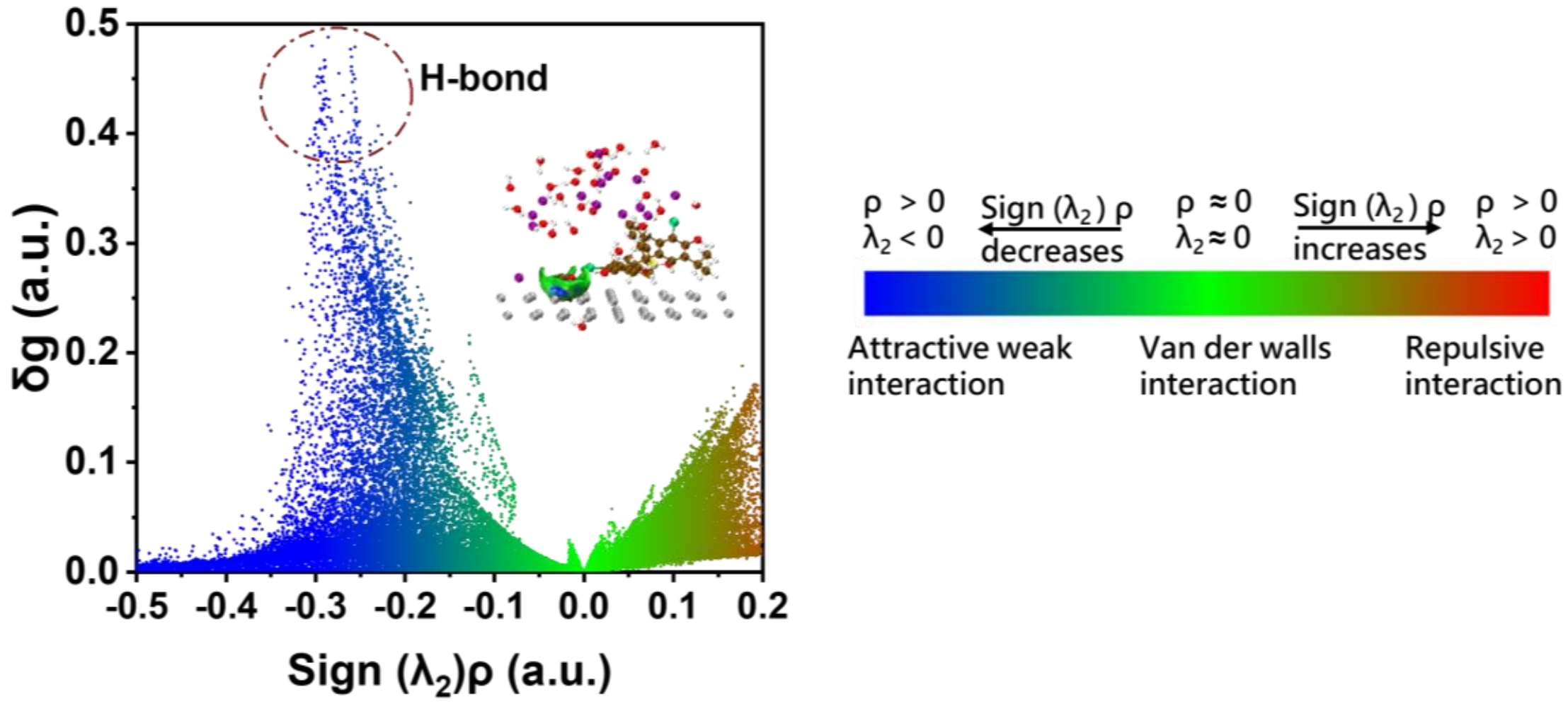

Figure 5d. Independent gradient model based on Hirshfeld partition (IGMH) analysis of the interaction between Ag-*CO and BTB, blue represents strong attraction, green represents van der Waals interaction, and red represents strong repulsion. Light gray, brown, red, yellow, green, light pink, and purple balls correspond to Ag, C, O, S, Br, H, and K atoms, respectively.

Q5. The successful functionalization of BTB is only demonstrated in a dry state. Given that BTB undergoes deprotonation in 1 M KOH, the molecule's affinity for the Ag surface and its structural integrity may change significantly. The authors should provide spectroscopic evidence (e.g., FT-IR or XPS) of the electrode after rinsing with the 1 M KOH electrolyte to confirm that the BTB layer remains intact in the reaction environment.

Response: We sincerely thank the reviewer for this constructive suggestion. We completely agree that confirming the structural integrity and interfacial stability of BTB in the 1.0 M KOH reaction environment is of paramount importance. As we elaborated in our previous response regarding the protonation state of BTB(Q1), the interfacial behavior of molecules at the solid-liquid boundary significantly deviates from bulk solution thermodynamics. The highly hydrophobic interface of Ag-BTB effectively repels bulk water and hydroxide ions, creating a restricted, low-dielectric microenvironment. This confinement prevents bulk-like deprotonation and strongly anchors the molecule to the Ag surface. To go a step further than merely rinsing the electrode, and to provide the most rigorous experimental proof of its stability, we have supplemented the revised manuscript with post-electrolysis FT-IR and XPS characterizations (Supplementary Figure S18-20) of the Ag-BTB catalyst after long-term durability testing in 1.0 M KOH. These results confirm that the BTB layer remains intact.

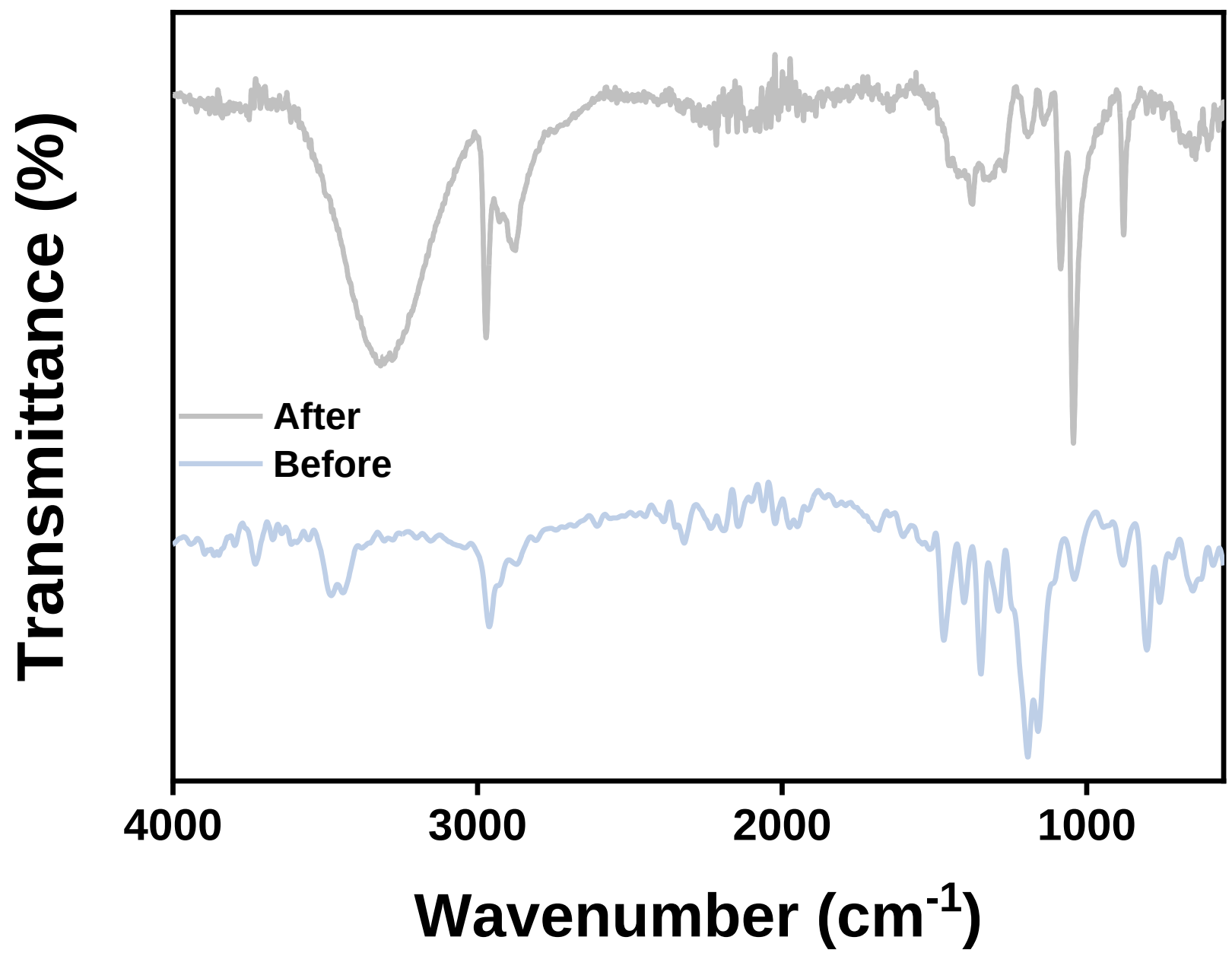

Figure S18. Post-stability FTIR characterization of Ag-BTB.

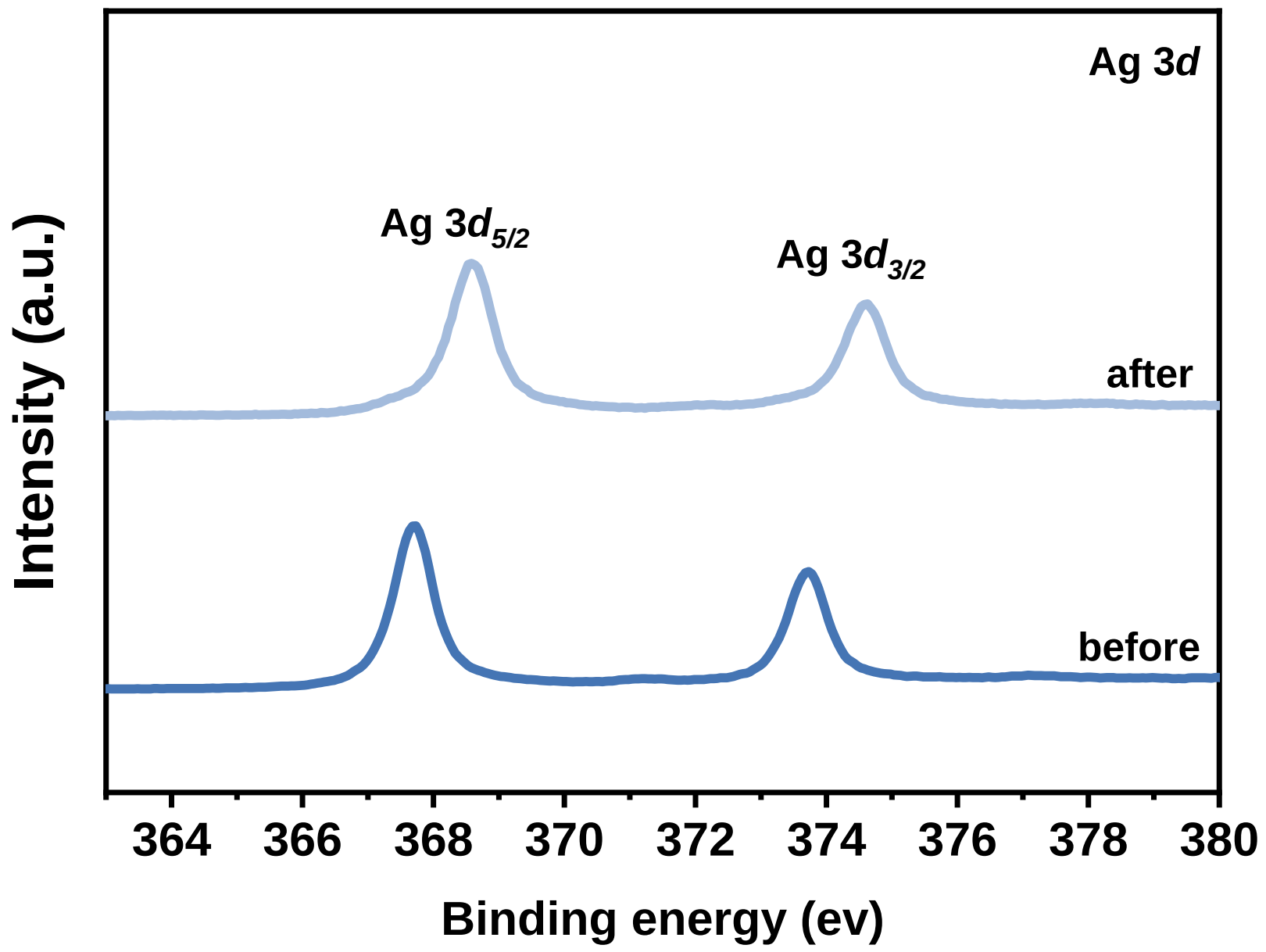

Figure 19. Post-stability XPS characterization of Ag 3d for Ag-BTB.

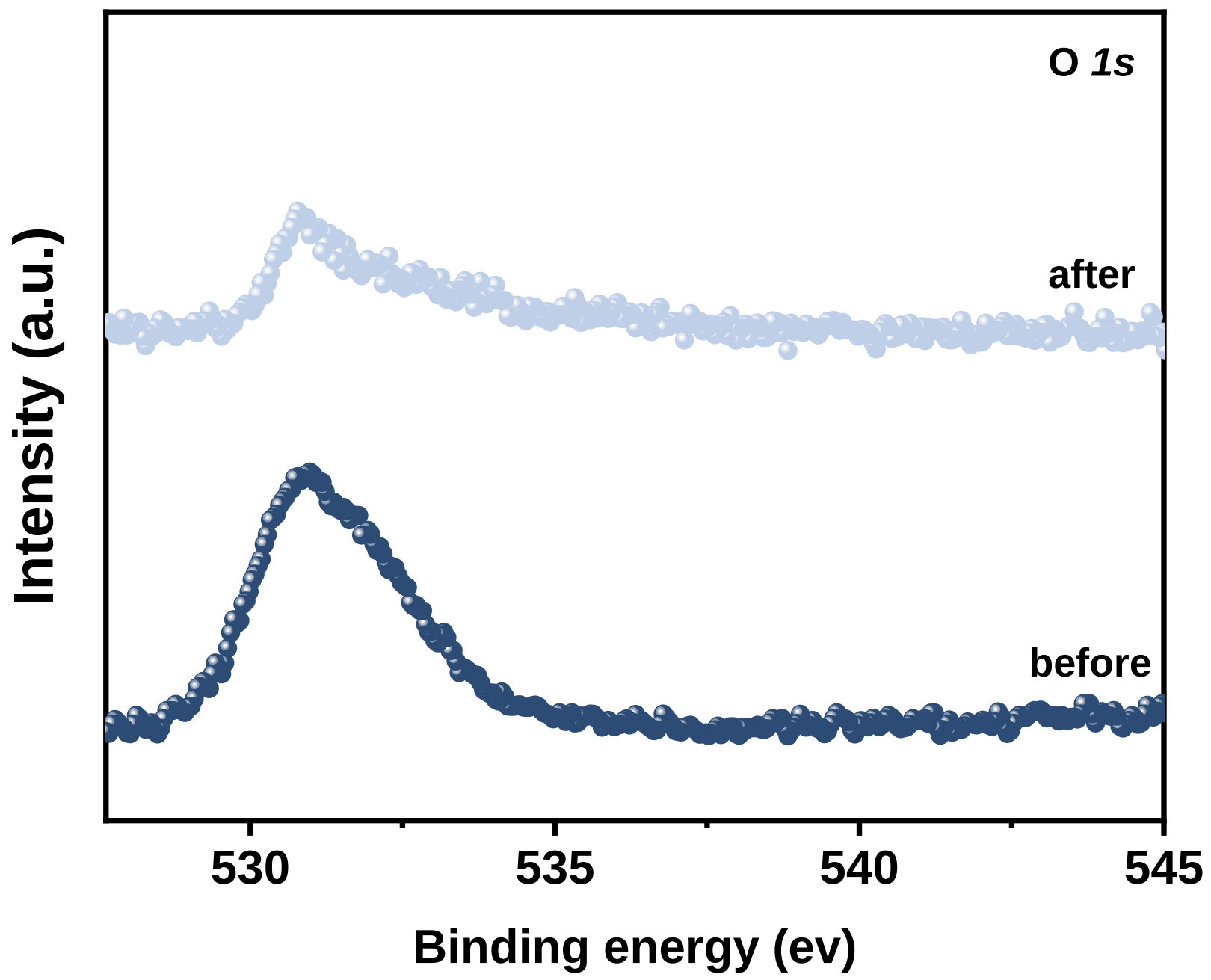

Figure S20. Post-stability XPS characterization of O 1s for Ag-BTB.

Q6. The synthesis section provides only a qualitative description of the functionalization process. Essential parameters, such as the concentration of the BTB solution, immersion time, temperature, and the resulting molecular loading are entirely missing. Without these quantitative metrics, the experiments are not reproducible.

Response: We sincerely appreciate the reviewer for pointing out this critical omission. We completely agree that precise quantitative parameters are absolutely essential to ensure the strict reproducibility of our experimental results. In response to your highly constructive feedback, we have thoroughly revised the "Synthesis of catalysts" section in the Supplementary Information. We have explicitly incorporated all the missing quantitative metrics, including the Ag film thickness (100 nm), the precise concentrations of the modifier solutions (0.1%, 0.19%, and 0.38% w/v), the sonication procedure, the immersion time (10 min), the temperature (room temperature), and the specific drying method (infrared lamp). This standardized immersion-drying protocol ensures a uniform and reproducible molecular loading across all batches. The control samples (Ag-PBBS, Ag-PEG, Ag-TB, and Ag-TPM) were also prepared using the exact same quantitative protocol.

The fully detailed and quantitative synthesis procedure has been updated in Supplementary Information.

The catalyst substrate was prepared using commercial carbon paper (Toray 30 T) as the base

material. Initially, a uniform thin silver (Ag) film was deposited onto the carbon paper via thermal evaporation in a high-vacuum deposition system. Prior to evaporation, the chamber was evacuated to a base pressure below 10 Pa to minimize potential contamination. High-purity silver (99.999%) was thermally evaporated from a tungsten boat at a strictly controlled deposition rate of 1 Å s^{-1} to ensure the formation of a homogeneous Ag film with a targeted thickness of 100 nm.

Following the physical deposition process, the Ag-coated carbon paper was further functionalized with molecular modifiers to enhance its electrocatalytic performance. For the preparation of the BTB-functionalized electrodes (denoted as Ag-BTB), a series of aqueous BTB solutions with varying concentrations of 0.1%, 0.19%, and 0.38% (w/v) were prepared and subjected to ultrasonication to ensure complete dissolution. The pristine Ag substrates were then immersed into these specific BTB solutions and kept undisturbed for 10 min at room temperature. This precisely controlled immersion step ensures sufficient and uniform molecular adsorption onto the catalyst surface. Subsequently, the freshly functionalized electrodes were carefully removed and completely dried under an infrared lamp to obtain the final Ag-BTB catalysts.

For comparative structure-activity studies, a series of control catalysts modified with different organic molecules were synthesized strictly following the identical protocol. These molecules include phenyl 4-bromobenzenesulfonate (Ag-PBBS), polyethylene glycol (Ag-PEG), phenolphthalein (Ag-PP), and triphenylmethane (Ag-TPM). They were prepared using the same standard mass/volume concentration (w/v) under the exact same immersion time, temperature, and drying conditions to ensure a standardized molecular loading across all experimental groups.

Q7. The 23-hour stability duration is inadequate to demonstrate long-term durability. Beyond the risk of chemical decomposition (e.g., reductive debromination) under cathodic polarization, the organic layer may also undergo physical detachment or leaching from the electrode surface due to vigorous operational conditions. Consequently, extended stability tests and more robust evidence are required to substantiate the persistence and structural integrity of the functionalized layer throughout the electrolysis process.

Response: We sincerely thank the reviewer for raising this critical and professional point. We completely agree that demonstrating robust long-term durability, while ruling out potential chemical decomposition (such as debromination) and physical detachment under vigorous cathodic

polarization, is essential for validating the practical potential of our Ag-BTB catalyst.

To address this rigorous requirement, we have significantly extended the continuous electrocatalysis durability test from 23 hours to 45 hours (Figure 2f in the revised manuscript). More importantly, to directly address the reviewer's concerns regarding chemical decomposition and physical leaching, we have performed comprehensive post-electrolysis spectroscopic characterizations (FT-IR and XPS Supplementary Figure S18-20) on the Ag-BTB electrode after this extended durability test. To confirm the persistence and structural integrity of the BTB layer.

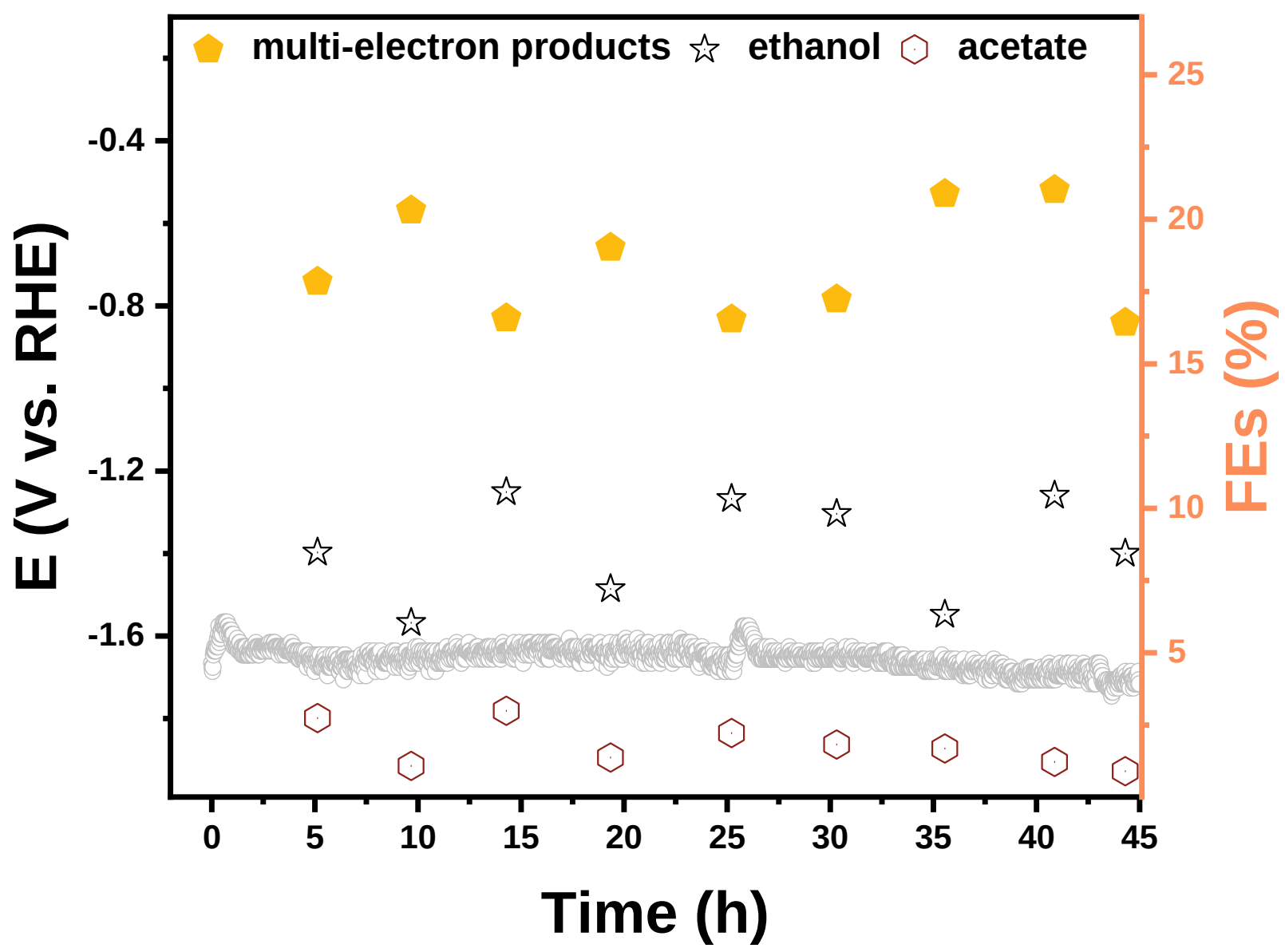

Figure 2f. Long-term CO_2RR performance on Ag-BTB at 300 mA cm^{-2} in a flow cell.

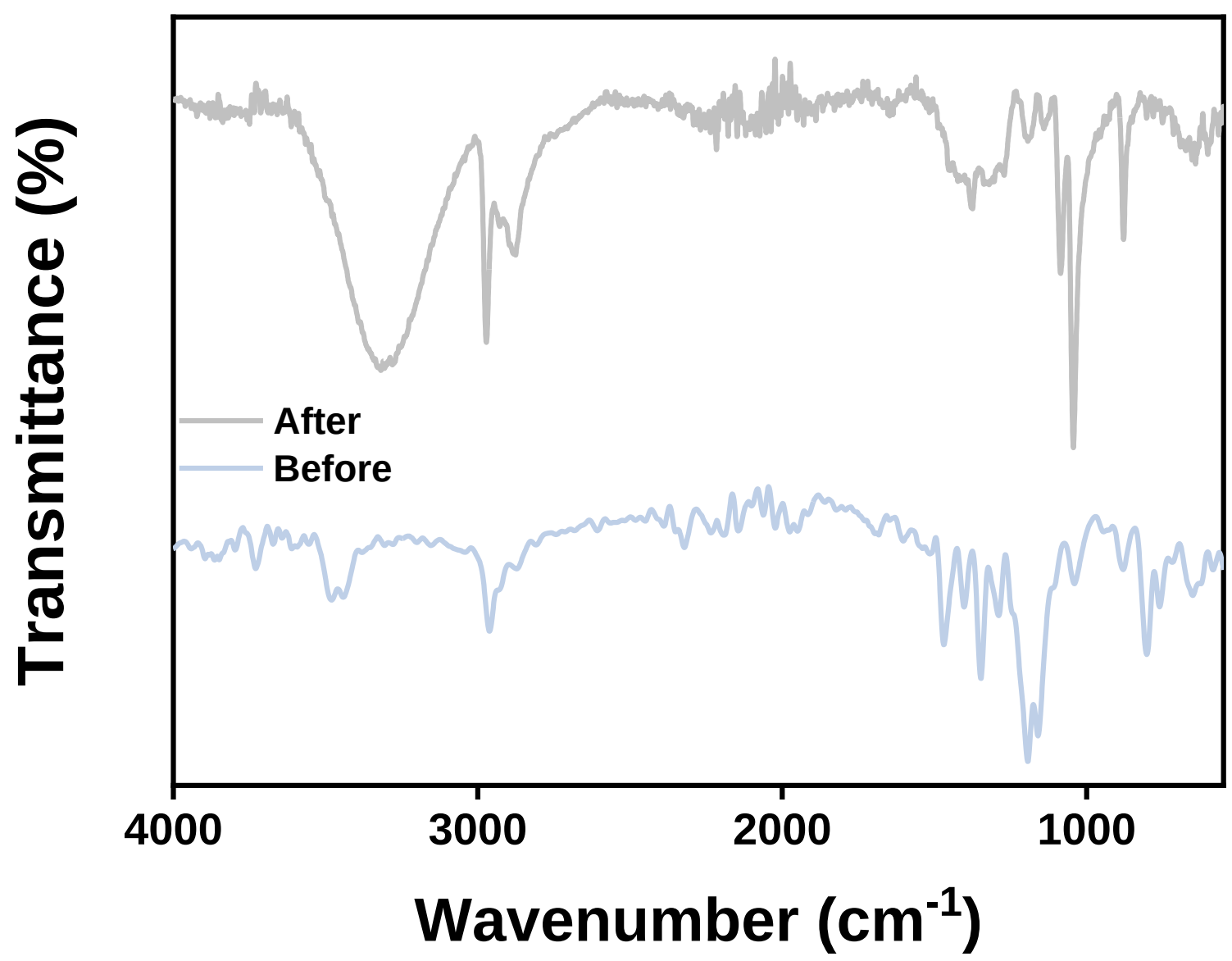

Figure S18. Post-stability FTIR characterization of Ag-BTB.

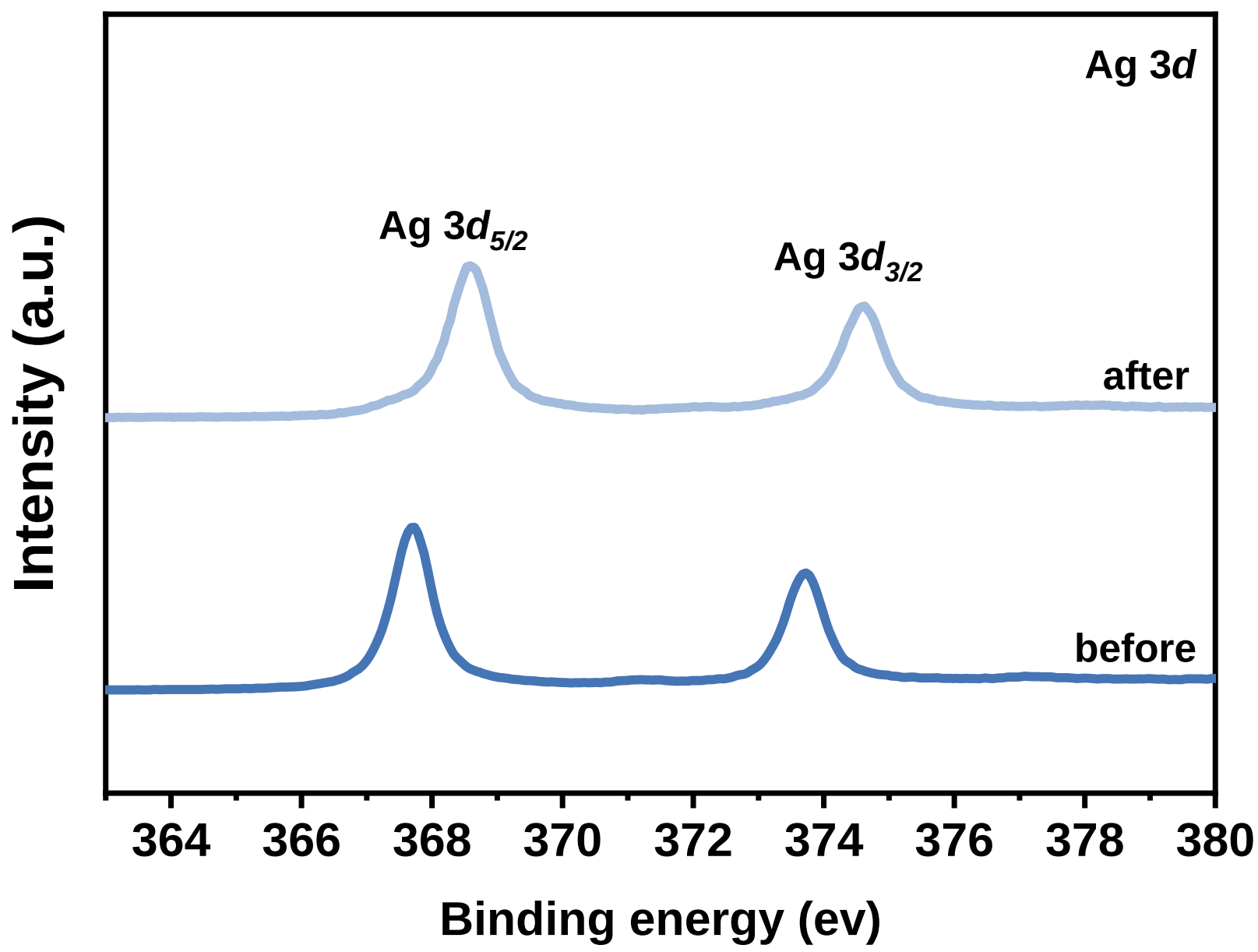

Figure 19. Post-stability XPS characterization of Ag 3d for Ag-BTB.

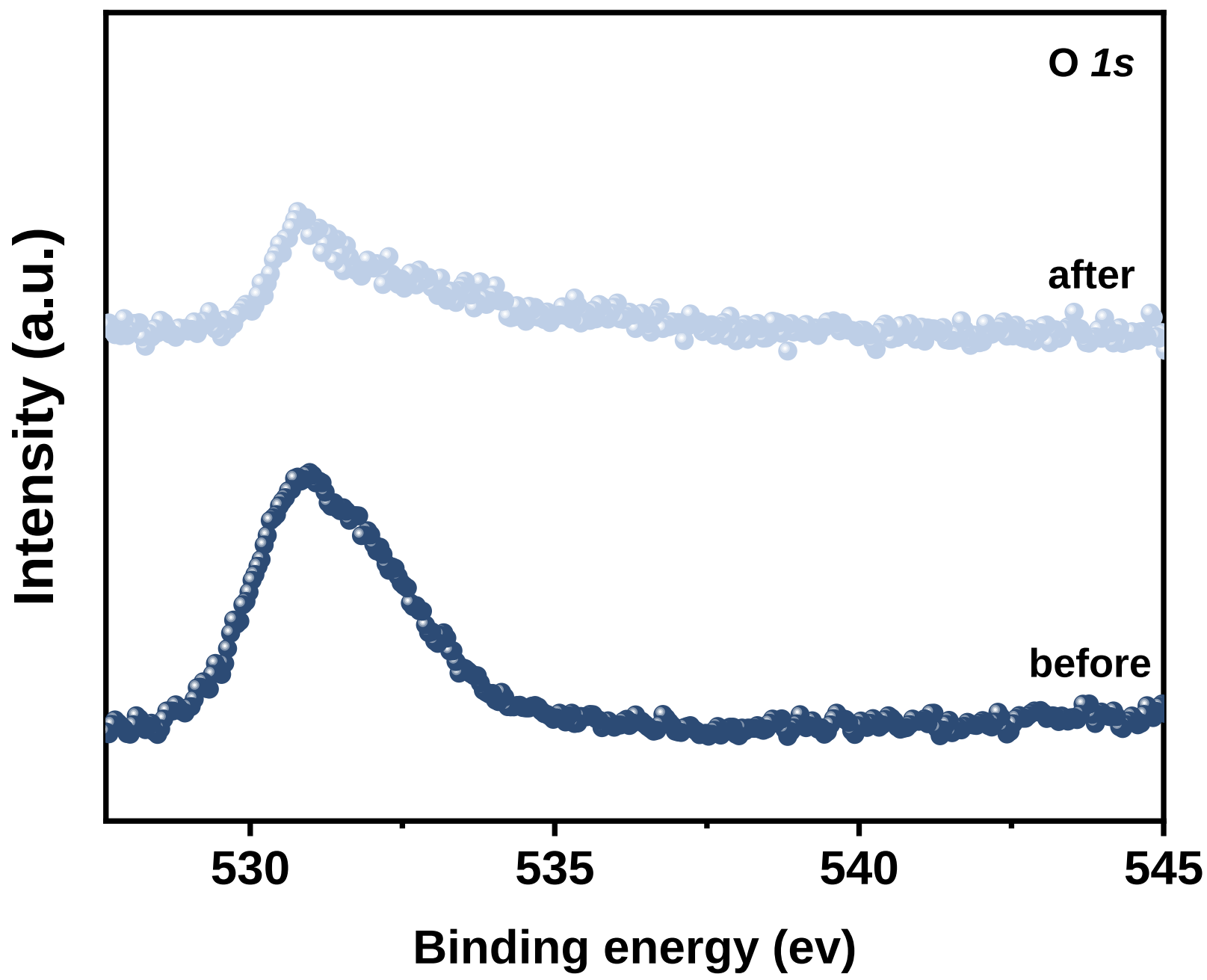

Figure S20. Post-stability XPS characterization of O 1s for Ag-BTB.

Q8. The origin of the detected multi-carbon products requires rigorous validation. There is a potential concern that these products could arise from the degradation of the BTB molecule rather than CO_2 reduction. To address this, ^{13}C isotopic labeling experiments are necessary to confirm that the carbon originates exclusively from the CO_2 feed.

Response: We sincerely thank the reviewer for raising this highly insightful and critical point. We completely agree that rigorously validating the carbon source is of utmost importance, particularly

in catalytic systems containing organic molecules like BTB, to definitively rule out any contribution from BTB degradation. To directly address this concern and provide unequivocal proof, we have performed ^{13}C isotopic labeling experiments using $^{13}CO_2$ as the feed gas under identical electrocatalytic conditions. Specifically, we employed both In-situ Differential Electrochemical Mass Spectrometry (DEMS), ^{1}H and ^{13}C Nuclear Magnetic Resonance (NMR) spectroscopy for comprehensive validation of both gaseous and liquid products. Specifically, we have performed *in situ* DEMS using $^{13}CO_2$ as the reactant. As shown in the revised Figure S21-23, the mass-to-charge ratio (m/z) signals clearly shifted to higher values, corresponding to the ^{13}C-labeled species: $^{13}CH_4$ was detected at m/z = 17 (compared to m/z = 15 for $^{12}CH_3^+$); ^{13}CO was detected at m/z = 29 (compared to m/z = 28 for ^{12}CO); $^{13}C_2H_4$ was detected at m/z = 30 (compared to m/z = 26 for $^{12}C_2H_2^+$);The reactant $^{13}CO_2$ was confirmed at m/z = 45.

Besides, we employed ^{1}H and ^{13}C NMR spectroscopy to analyze the liquid products collected after the reaction. Notably, in the ^{1}H NMR spectra, the characteristic peaks of the liquid products evolved from standard singlets into distinct multiplets. This peak splitting is a direct consequence of the robust heteronuclear spin-spin coupling (*J*-coupling) between the $^{13}CO_2$ nuclei (spin I = 1/2) and adjacent protons (^{1}H), providing unequivocal evidence of ^{13}C incorporation into the molecular skeletons. This was definitively corroborated by the ^{13}C NMR spectra, which clearly resolved the characteristic chemical shifts of the ^{13}C-labeled oxygenates: bicarbonate at ~ 161 ppm; formate at ~171 ppm; acetate at ~181 ppm (carboxyl carbon) and ~23 ppm (methyl carbon); and ethanol at ~58 ppm (methylene carbon) and ~18 ppm (methyl carbon). Mechanistically, these isotopic labeling results provide rigorous analytical closure. They explicitly confirm that the highly valuable multi-electron products are synthesized de novo via the C–C coupling of CO_2-derived intermediates.

The corresponding data and discussions have been added to the revised Manuscript and the Supplementary Information. We hope these additional results can address your concerns. Furthermore, we have uploaded all the raw data as a separate file in this revised version.

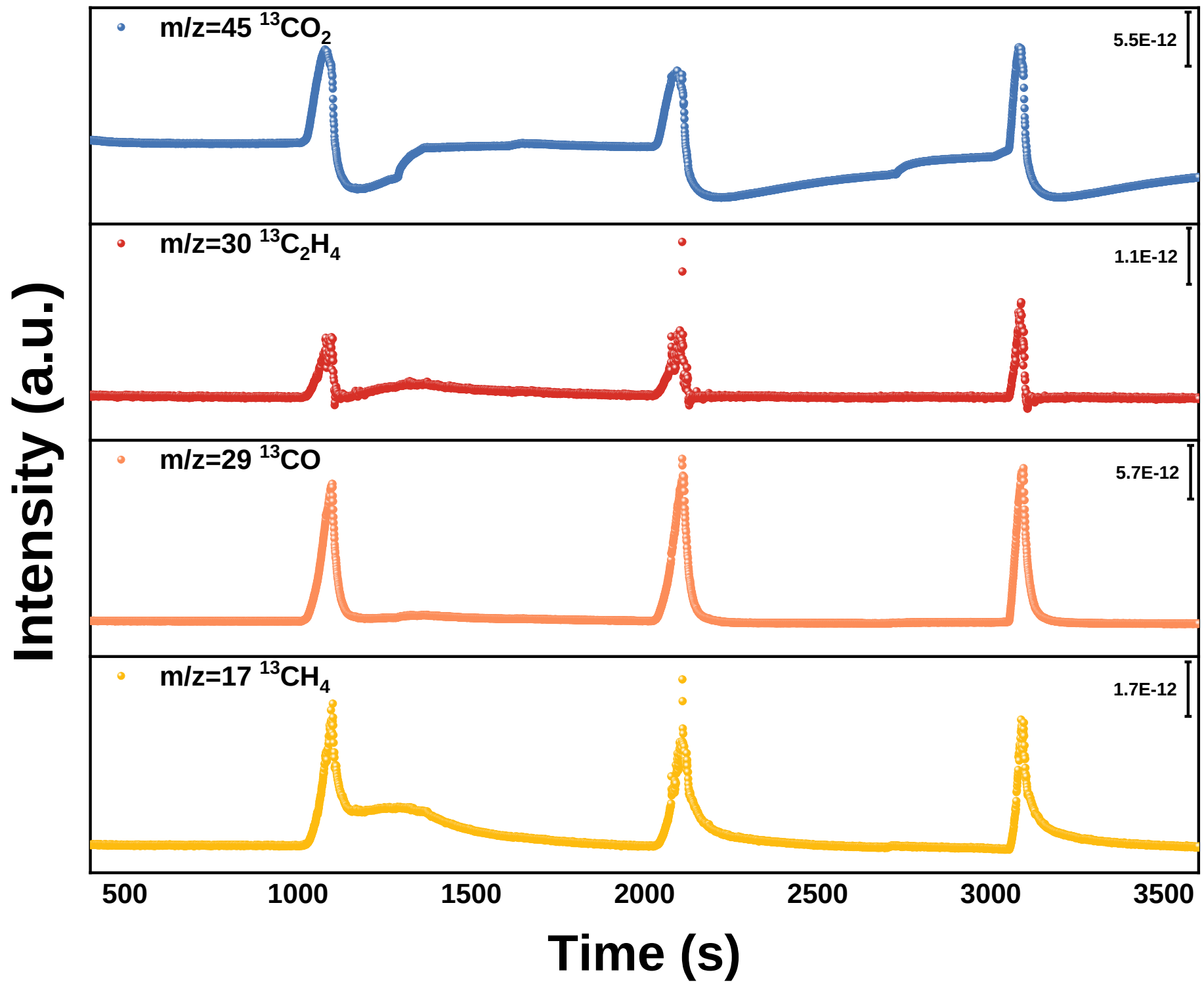

Figure S21. *In situ* Differential Electrochemical Mass Spectrometry (DEMS) measurements using $^{13}CO_2$ as the feed gas.

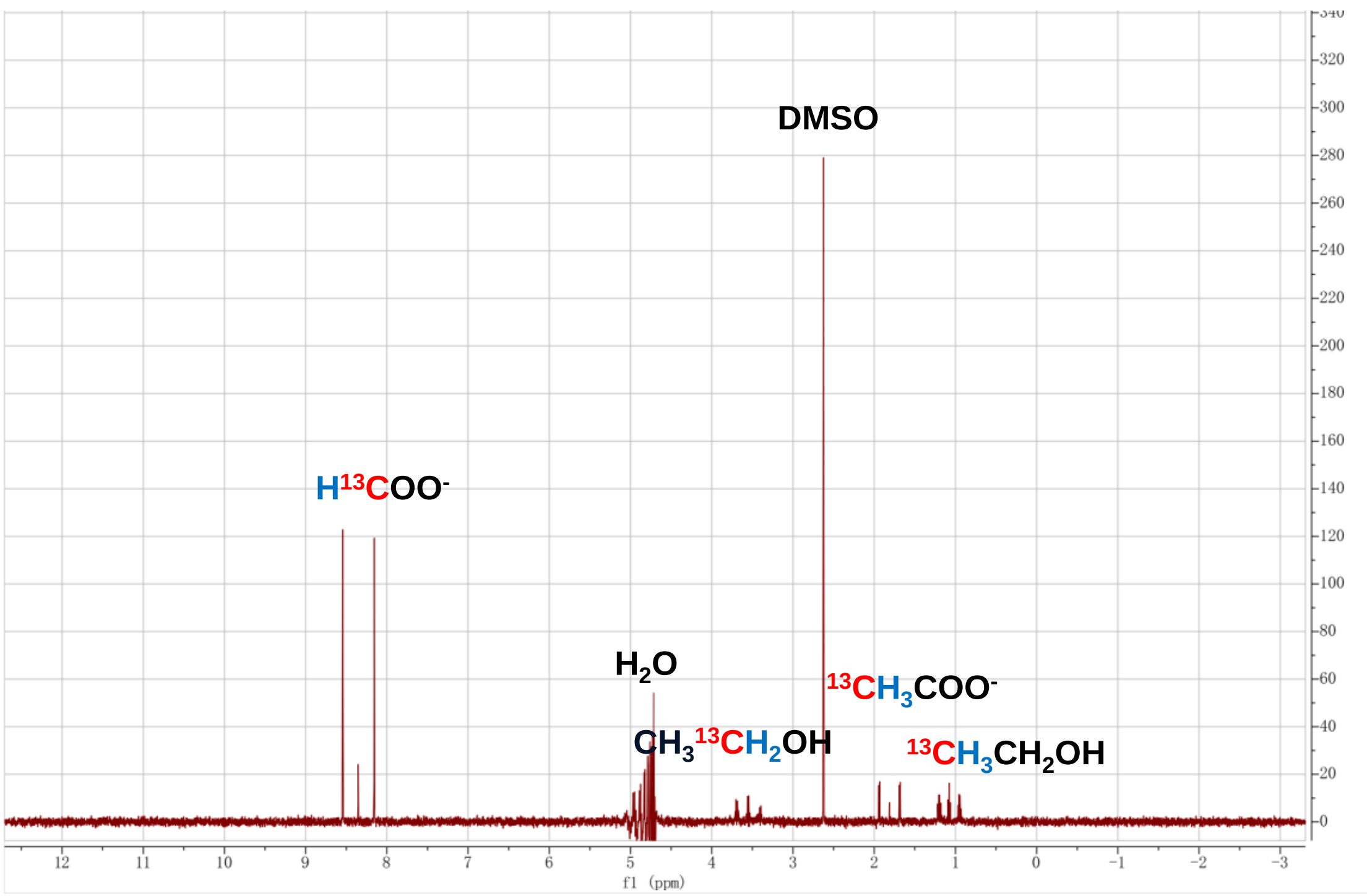

Figure S22. ^{1}H Nuclear Magnetic Resonance (NMR) spectrum of the liquid electrolyte after $^{13}CO_2$ electroreduction.

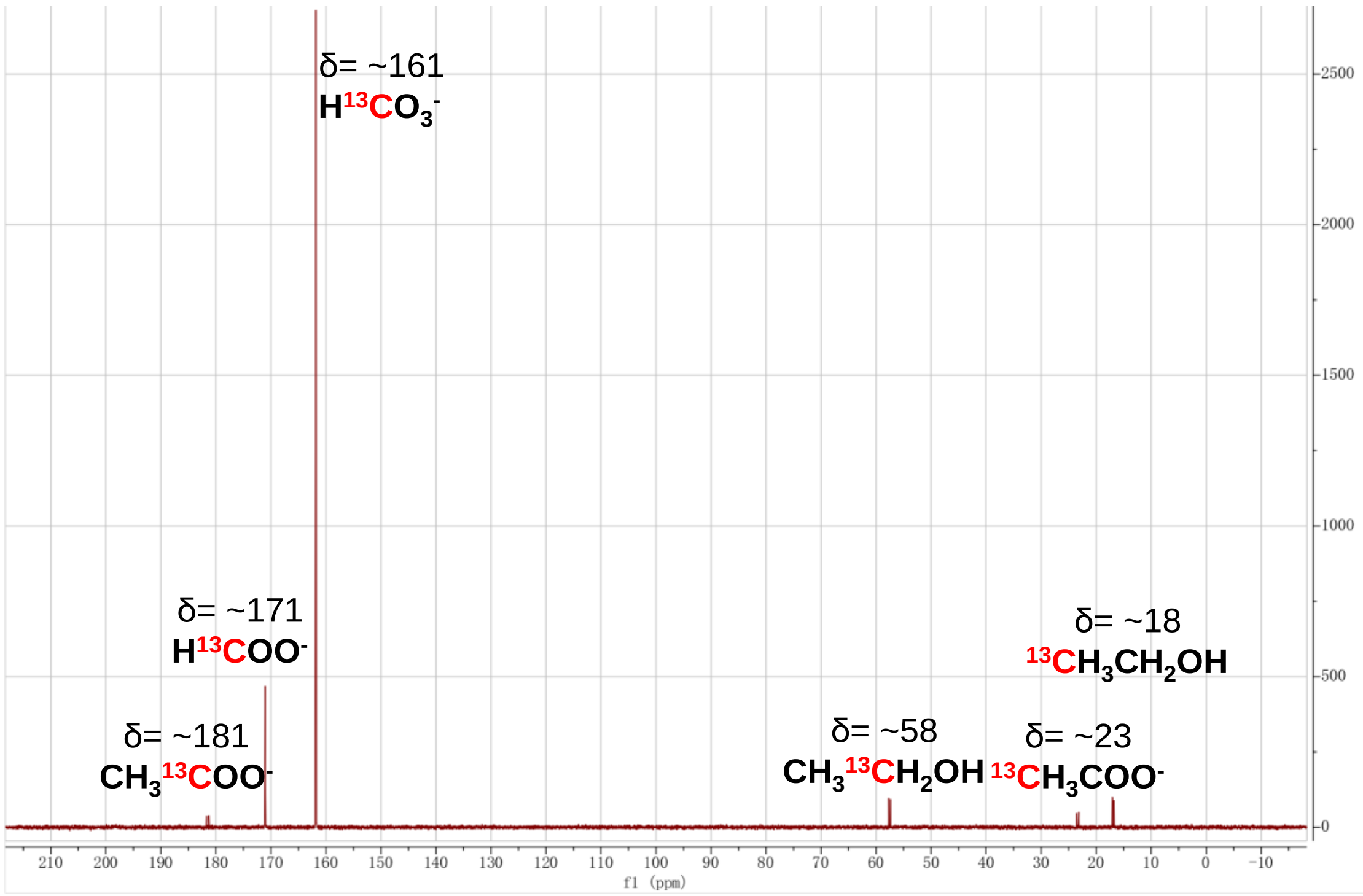

Figure S23. ^{13}C Nuclear Magnetic Resonance (NMR) spectrum of the liquid electrolyte after $^{13}CO_2$ electroreduction.

Thank you again for reviewing this manuscript. These constructive suggestions have made a real improvement of our work. We truly appreciate your help.
